# UpTCR: a unified progressive knowledge transfer foundation model for robust T-cell receptor-antigen binding recognition

**DOI:** 10.1038/s41467-026-78075-x

**Published:** 2026-09-24

**Authors:** Tianxu Lv, Yang Xiao, Li Chen, Bing He, Maiyi Zhong, Zihan Feng, Zheyu Hu, Fei Ye, Jiashu Han, Shouzhi Chen, Zhenchao Tang, Jiale Zhou, Dawei Huang, Xiaoqing Lian, Jiansong Fan, Yixuan Huang, Chenyi Lei, Dandan Meng, Yuan Liu, Lihua Li, Pengjiang Qian, Jianhua Yao, Kai Miao, Xiao Liu, Xiang Pan

**Affiliations:** 1 School of Artificial Intelligence and Computer Science, Jiangnan University, Wuxi, 214122 China; 2 Tsinghua Shenzhen International Graduate School, Tsinghua University, Shenzhen, 518071 China; 3 Cancer Centre and Institute of Translational Medicine, Faculty of Health Sciences, University of Macau, Macau, 999078 China; 4 AI for Life Sciences Lab, Tencent, Shenzhen, 518000 China; 5 Department of general surgery, Peking Union Medical College Hospital, Beijing, China; 6 Institute of Biomedical Engineering and Instrumentation, Hangzhou Dianzi University, Hangzhou, 310018 China; 7 The PRC Ministry of Education Engineering Research Center of Intelligent Technology for Healthcare, Wuxi, Jiangsu 214122 China

**Keywords:** Machine learning, Computational models, Data mining, MHC, T-cell receptor

## Abstract

T-cell receptor (TCR) recognition of antigenic peptides presented by human leukocyte antigen (HLA) molecules underpins adaptive immunity and T cell-based immunotherapy. However, the scarcity of complete interaction data and TCR cross-reactivity challenge robust prediction. Here, we present UpTCR, a unified progressive knowledge-transfer foundation model that learns from incomplete data to predict TCR-antigen-HLA binding. UpTCR progressively transfers knowledge from dimeric and trimeric interactions to tetrameric complexes and uses soft contrastive learning to mitigate false negatives. It outperforms existing methods in predicting TCR binding specificity and antigen-HLA binding affinity, particularly for neoantigens, and reveals pairwise residue-level interactions across the tetramer. UpTCR also transfers effectively to breast cancer cohorts with limited data. Prospective validation against melanoma antigen variants identifies eight immunogenic peptides that elicit T cell responses and one variant associated with immune escape. These findings establish UpTCR as a generalizable and interpretable tool for studying antigen recognition and advancing TCR-based immunotherapies.

## Abstract (web summary)

Peptides are presented to T Cell receptors (TCR) in the context of human leukocyte antigen (HLA) MHC molecules and the prediction of these peptide sequences are important for vaccines and cancer therapeutics. Here the authors present UpTCR a computational method for learning from incomplete data for TCR-antigen-HLA prediction, considering antigen-HLA binding affinity and specificity and using this tool to predict epitopes in breast cancer patient cohorts.

## Introduction

The specificity of T cell-mediated immune responses relies on the precise recognition of antigenic peptides presented by human leukocyte antigen (HLA) molecules through T-cell receptors (TCRs)^1,2^. This TCR-antigen-HLA ternary interaction serves as the linchpin of adaptive immunity, governing critical processes from pathogen clearance to cancer immunosurveillance^3,4^. The ability to computationally decode this molecular handshake would not only illuminate fundamental mechanisms of immune recognition but also facilitate the rational design of vaccines and T cell therapies with tailored specificity^5–7^. However, a comprehensive understanding of this interaction is hindered by structural complexity, experimental limitations, and intrinsic cross-reactivity. First, TCRs are composed of alpha (α) and beta (β) chains, each containing a crucial fragment variable (FV) region responsible for the specific recognition of antigens^8^. The FV region consists of the complementarity-determining regions (CDRs, i.e. CDR1, CDR2, CDR2.5^9^ and CDR3) and framework regions (FRs). Among these, CDRs, particularly CDR3, play a vital role in the recognition of antigens, while FRs provide structural support. A unified modeling approach that integrates both CDRs and FRs could significantly enhance our understanding of TCR specificity and binding mechanisms. Second, current experimental techniques, such as single-cell-based tetramer sequencing^10^, particularly in the simultaneous acquisition of TCRα and TCRβ chain sequences, still face challenges such as high cost, technical complexity and limited epitope coverage^11^. Third, TCR cross-reactivity^12^, where a single TCR can bind to multiple antigen-HLA complexes, presents both opportunities and challenges in T cell-based therapies^13,14^. While this property can be harnessed to target a broader range of tumor antigens, it also raises concerns about potential off-target effects and unintended immune responses to self-antigens^15^. Understanding the mechanisms underlying TCR cross-reactivity is essential for mitigating these risks.

Recent deep learning-based approaches have shown significant potential in addressing these challenging issues. Early methods primarily focus on the interaction between intracellular peptides and HLA-I molecules, including NetMHCpan^16^, MHCflurry^17^, DeepMHCI^18^, ACME^19^, TransPHLA^20^, and PickPocket^21^. However, the binding of peptides to HLA is only the initial phase in tumor-specific immune responses. To achieve a comprehensive understanding of this process, it is essential to consider TCR-antigen binding recognition, as exemplified by tools such as NetTCR^22^, ERGO^23^, DLpTCR^24^, TITAN^25^, PanPep^26^, and TEIM-Res^27^. More recently, a handful of approaches, such as pMTnet^28^ and UnifyImmun^29^, simultaneously consider antigen-HLA binding and TCR-antigen binding, leading to improved results in pairwise binding prediction. Furthermore, studies such as PISTE^30^ and EPACT^31^ have been proposed to directly predict TCR-antigen-HLA interactions, a more challenging task due to the complex interaction landscape and extensive molecular diversities^32^.

Despite this progress in modeling TCR-antigen-HLA interactions, accurate and robust prediction of T cell-related immune responses remains an unsolved challenge. This challenge is largely due to 1) the limited availability of high-throughput complete experimental data, restricting the size and diversity of the training data, and 2) the tendency of current methods to focus on only a subset of available data, limiting their generalization ability. Note that the total amount of pairwise data is 5.6-fold greater than that of the ternary data, and there is no redundancy between the two data categories (see Methods, Supplementary Fig. S1). Thus, a core question is how to effectively leverage the abundant pairwise data to enhance model generalizability for TCR specificity prediction. Second, existing computational approaches for TCR-antigen binding prediction, while increasingly incorporating both TCRα and TCRβ chains (e.g., TCRdist^9^, RACER^5^), often still rely predominantly on the CDR3 loop of the TCRβ chain. While the CDR3β plays a dominant role in antigen recognition, the TCRα chain and several components of the complete fragment variable (FV) region (e.g., CDR1, CDR2, and CDR2.5) also interact with the antigen-HLA complexes and contribute to the overall interaction. Thus, the unified modeling of TCRα and TCRβ, as well as simultaneously considering the supplementary information of FV, should provide a more comprehensive understanding of TCR binding specificity. Third, current methods primarily generate negative samples through random shuffling or various sampling strategies, while training models with hard labels, i.e. 0 and 1. Such an approach may introduce false-negative issues, making it challenging for models to perform in-depth analysis of TCR cross-reactivity.

In this work, we present UpTCR (Unified progressive modeling for TCR binding recognition), a unified progressive pretrained foundation model, which integrates any pairwise interactions, for paired αβ T Cell Receptor-antigen-HLA binding recognition. To comprehensively leverage incomplete data, we introduce a progressive soft contrastive pretraining strategy (see Methods) that learns prior knowledge from dimers (e.g., TCRα-antigen and antigen-HLA) to trimers (e.g., TCRα-TCRβ-antigen, TCRβ-antigen-HLA), and then leverages transfer learning for tetramer (i.e., TCRα-TCRβ-antigen-HLA) binding prediction. The presented soft contrastive learning innovatively incorporates intra-modal similarity to soften hard labels used in conventional methods. This softening not only alleviates potential false negative issues to improve the model’s robustness, but also allows UpTCR to handle TCR cross-reactivity. UpTCR also employs a multi-scale modeling approach (see Methods) for TCRs, leveraging the evolutionary and structural representations from pretrained large language models (LLMs)^33^, the global information from the FV region, and the critical motifs within the CDR3. By effectively integrating the progressive pretraining strategy and multi-scale representations, UpTCR exhibits several advantages over previous methods on understanding T cell-mediated immune responses. (1) UpTCR significantly improves the performance of TCR-antigen-HLA binding prediction compared to state-of-the-art methods, particularly in predicting interactions with unseen antigens. UpTCR also demonstrates superior predictive capability even using incomplete data. (2) UpTCR serves as a powerful tool for investigating TCR cross-reactivity via its innovative soft contrastive pretraining. This capability allows researchers to explore the nuances of TCR interactions with multiple antigens, providing insights into the mechanisms underlying cross-reactive responses that are critical for vaccine design and immunotherapy. (3) UpTCR facilitates the elucidation of any pairwise residue-level contacts within the entire TCRαβ-antigen-HLA interaction process. It not only reveals the interactions between full-length TCR sequences (including CDRs and framework regions) and antigens, but also uncovers pairwise contacts, such as TCRα-TCRβ and antigen-HLA interactions. Notably, UpTCR exhibits strong transferability in predicting TCR-antigen interactions across diverse molecular subtypes within in-house breast cancer patient cohorts, attaining superior performance with limited transfer data. Furthermore, in a prospective study, UpTCR accurately predicts TCR cross-reactivity against melanoma antigen variants, identifying eight immunogenic variant peptides that provoke T cell responses, and one variant linked to immune escape. Our analyses demonstrate the application potential of UpTCR in advancing the understanding of T cell-mediated immune responses and enhancing the design of targeted immunotherapies.

## Results

### The UpTCR algorithm

**Fig. 1:**
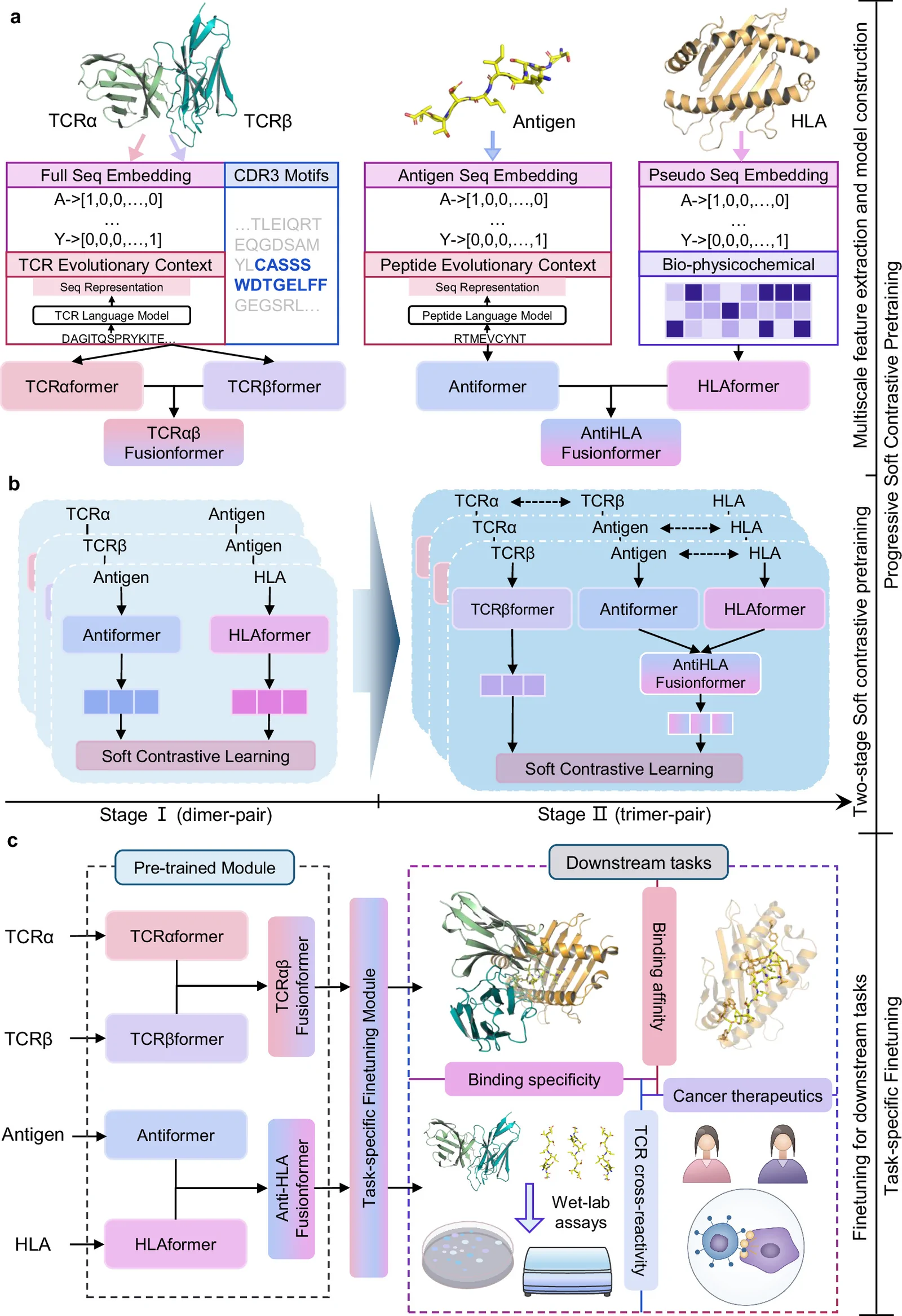
Illustration of the UpTCR foundation model. **a** UpTCR derives a set of features, including the full-length sequence, evolutionary representations and CDR3 motifs of TCRα and TCRβ, the sequence and evolutionary representations of antigens, as well as the pseudo sequences and bio-physicochemical properties of HLA. Based on these features, we developed TCRαformer, TCRβformer, Antiformer, and HLAformer to integrate and learn the high-level representations of each element. These extracted single representations are fed into the developed TCRαβFusionformer and AntiHLAFusionformer to acquire a unified representation of TCRα-TCRβ and antigen-HLA, respectively. **b** To fully utilize data with missing modalities and address the issue of cross-reactivity, we performed progressive soft contrastive pretraining on UpTCR. The pretraining consists of two stages. In the first stage, we used pairwise data, such as antigen-HLA binding, TCRα-antigen binding, and TCRβ-antigen binding, primarily focusing on pretraining the TCRαformer, TCRβformer, Antiformer, and HLAformer. We proposed a soft contrastive learning strategy that not only brings positive samples closer together and pushes negative samples further apart, but also effectively handles false-negative samples through similarity mining. In the second stage, we used triplet data, such as TCRα-TCRβ-antigen binding and TCRα-antigen-HLA binding, primarily to pretrain the TCRαβFusionformer and AntiHLAFusionformer to obtain unified representations for TCRα-TCRβ and antigen-HLA, respectively. **c** After pretraining, these pretrained modules are employed for various downstream tasks, including TCR-antigen-HLA binding specificity prediction, antigen-HLA binding affinity prediction, rapid translation in cancer therapeutics, and TCR cross-reactivity analysis. TCR T-cell receptor, HLA human leukocyte antigen, CDR complementarity-determining region, FV fragment variable, LLM large language model.

UpTCR employs a modular divide-merge architecture to model TCRαβ-antigen-HLA interactions (Fig. 1). Individual encoders first capture the distinct properties of TCRα, TCRβ, antigen, and HLA. Pairwise fusion encoders then integrate TCRαβ and antigen-HLA complexes, and finally, the unified tetramer representation is used for downstream prediction. Specifically, UpTCR comprises monomer modeling encoders (TCRαformer, TCRβformer, Antiformer, and HLAformer), dimer complex fusion encoders (TCRαβFusionformer and AntiHLAFusionformer), and task-specific finetuning modules (Fig. 1a). For TCRα and TCRβ, UpTCR derives multi-scale features including full-length sequence embeddings, CDR3 motifs, and evolutionary and structural information from a pretrained TCR LLM. For the antigen, sequence embeddings and evolutionary information are extracted through a peptide LLM. For HLA, pseudo sequence^34,35^ embeddings and bio-physicochemical properties (e.g., Atchley factor^36^ and BLOSUM^37^) are used. The learned representations of TCRα and TCRβ are fused via TCRαβFusionformer, and the antigen and HLA representations are integrated via AntiHLAFusionformer. The joint TCRαβ and antigen-HLA representations are then combined by task-specific finetuning modules for downstream tasks.

To leverage incomplete data, we developed a progressive soft contrastive pretraining strategy (Fig. 1b) that maps paired data into a co-embedding space while addressing potential false-negative issues. We organized all available data into three categories (see Methods): dimers (TCRα-antigen, TCRβ-antigen, antigen-HLA), trimers (TCRα-TCRβ-antigen, TCRα-antigen-HLA, TCRβ-antigen-HLA), and the complete tetramer (TCRα-TCRβ-antigen-HLA). To prevent data leakage, any pretraining sample was excluded if its antigen or TCR CDR sequence shared >30% identity with any sequence in the downstream test sets. This strict separation ensures that model performance reflects genuine generalization rather than memorization of homologous sequences. In Stage I, we pretrained the four monomer encoders using dimer data (Fig. 1b). For TCRα-antigen and TCRβ-antigen interactions, we introduced SoftCLIP (see Methods, Supplementary Fig. S4), an intra-modal similarity-regularized contrastive learning algorithm. Unlike traditional CLIP^38^, which treats all non-matching pairs as hard negatives—potentially contradicting TCR cross-reactivity where a single TCR may bind multiple homologous antigens—SoftCLIP incorporates sequence similarity between antigens to soften the contrastive targets, allowing the model to learn a gradient of binding likelihood reflective of biophysical reality. For antigen-HLA pretraining, we proposed SoftTri (see Methods, Supplementary Fig. S5), a soft triplet contrastive learning approach that integrates quantitative binding affinity (IC_50_) as a regularization coefficient, rather than relying on a binary 500 nM threshold^16,34^. This affinity-aware strategy reflects the continuous nature of antigen-HLA binding. By pretraining on dimer data, UpTCR aligns the embeddings of all four components into a unified latent space. In Stage II, we pretrained the two fusion encoders using trimer data (Fig. 1b). For TCRα-antigen-HLA and TCRβ-antigen-HLA data, the AntiHLAFusionformer merges antigen and HLA representations, and SoftCLIP aligns these fused embeddings with TCRα/TCRβ. For TCRα-TCRβ-antigen data, the TCRαβFusionformer integrates TCRα and TCRβ representations, aligned with the antigen embedding via SoftCLIP. The resulting pretrained encoders provide representations for multiple downstream tasks (Fig. 1c): (1) TCR binding specificity prediction under diverse generalization settings; (2) antigen-HLA binding affinity prediction; (3) interpretable residue-level interaction analysis; (4) translational validation in cancer cohorts; and (5) TCR cross-reactivity analysis with prospective experimental validation.

### UpTCR achieves state-of-the-art performance in predicting TCR specificity

We evaluated UpTCR’s predictive performance in predicting TCR-antigen-HLA recognition across four distinct settings: majority, few-shot, zero-shot with 50% sequence identity filtering, and zero-shot with 30% sequence identity filtering. For this evaluation, binding specificity is defined as a binary classification task, where a label of 1 indicates a positive binding event and 0 indicates non-binding. We collected a comprehensive TCR-antigen-HLA dataset consisting of 36,061 positive samples. Currently, negative sample construction primarily relies on two methods: shuffling and background data sampling. However, sampling from background data can easily introduce significant biases^39^, causing the model to learn differences related to data collection rather than genuine biological signals. This may lead to high performance on the test set but a sharp decline in real-world scenarios. Therefore, in this work, we employed the shuffling method, and the ratio of positive to negative samples was approximately 1:5. We compared the proposed UpTCR against several state-of-the-art baselines, including DLpTCR^24^, NetTCR2.0^22^, ERGO-AE^23^, ERGO-LSTM^23^, pMTnet^28^, PanPep^26^, and EPACT^31^. To ensure a rigorous and fair comparison, all competing models were implemented and retrained using the same curated and filtered dataset utilized for UpTCR.

**Fig. 2:**
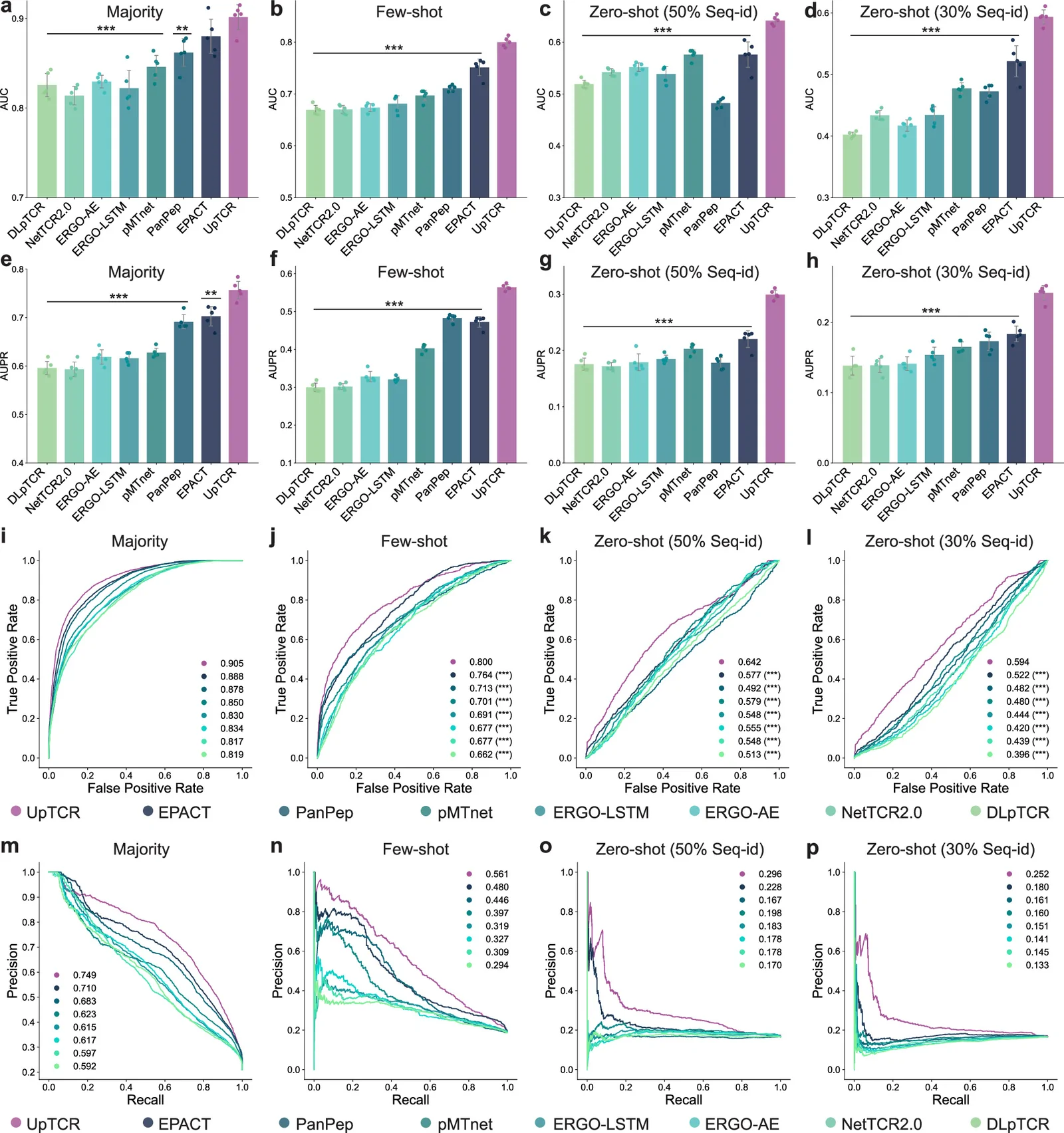
UpTCR achieves superior performance in predicting TCR-antigen-HLA binding specificity across diverse evaluation settings. **a–d** Bar plots of AUC for all benchmark methods under the majority (**a**), few-shot (**b**), zero-shot with 50% sequence identity filtering (**c**), and zero-shot with 30% sequence identity filtering (**d**) settings. The few-shot setting refers to a scenario in which only a limited number of training samples are available for each antigen. The zero-shot setting represents a more challenging scenario in which the model is required to predict TCR-antigen-HLA binding specificity for antigens that are entirely absent from the training data, with sequence identity filtering applied to ensure strict separation between training and test sets. Bars represent the mean AUC and error bars indicate standard deviations. Individual data points from *n* = 5 independent runs are overlaid. Statistical comparisons between UpTCR and each baseline were performed using a two-sided unpaired Student’s t-test. *P* values were not adjusted for multiple comparisons. **e–h** Bar plots of AUPR under the same four settings: majority (**e**), few-shot (**f**), zero-shot with 50% sequence identity filtering (**g**), and zero-shot with 30% sequence identity filtering (**h**). Bars represent the mean AUPR and error bars indicate standard deviations. Individual data points from *n* = 5 independent runs are overlaid. **i–l** Receiver Operating Characteristic (ROC) curves for all benchmark methods under the majority (**i**), few-shot (**j**), zero-shot with 50% sequence identity filtering (**k**), and zero-shot with 30% sequence identity filtering (**l**) settings. Each curve represents one method, with the legend indicating the AUC value. Statistical significance of the improvement in AUC was assessed by a two-sided DeLong’s test. **m–p** Precision-Recall (PR) curves for all benchmark methods under the majority (**m**), few-shot (**n**), zero-shot with 50% sequence identity filtering (**o**), and zero-shot with 30% sequence identity filtering (**p**) settings. AUC, area under the receiver operating characteristic curve; AUPR, area under the precision–recall curve; ROC, receiver operating characteristic; PR, precision-recall. Exact *P* values are provided in the Source Data. *P < 0.05, **P < 0.01, ***P < 0.001.

In the majority setting, where the majority of relevant data for each antigen is present in the training set, UpTCR achieved an AUC (Area Under the Curve) of 0.905 and an AUPR (Area Under the Precision-Recall Curve) of 0.749, outperforming all baselines (Fig. 2a, e). In the few-shot setting, where only a limited number of training samples are available for each antigen, UpTCR attained an AUC of 0.800 and AUPR of 0.561, outperforming the next-best method (EPACT) by 3.6% in AUC and 8.1% in AUPR (Fig. 2b, f). We then assessed UpTCR in two zero-shot settings of increasing stringency, where none of the antigens in the test set appeared in the training data. Under 50% sequence identity filtering, UpTCR achieved an AUC of 0.642 and AUPR of 0.296, exceeding the next-best baseline by 6.5% in AUC (Fig. 2c, g). Under a more stringent 30% threshold, UpTCR maintained the highest AUC of 0.594 and AUPR of 0.252, outperforming all baselines (*P* < 0.001, Fig. 2d, h). The corresponding ROC and PR curves (Fig. 2i–p) further corroborated these findings across all four settings.

To further assess the early retrieval performance of UpTCR, we computed the partial AUC at a false positive rate of 0.1 (AUC0.1) across all four settings (Supplementary Fig. S8). UpTCR consistently achieved the highest AUC0.1 among all benchmark methods (*P* < 0.01) across all settings, demonstrating superior discriminative performance within the high-specificity regime. The result indicates that UpTCR’s most confident predictions are reliable for prioritizing candidates for downstream experimental validation.

To further evaluate UpTCR’s discriminative power in a biologically stringent setting, we constructed a hard-negative benchmark comprising 3,495 TCR-epitope pairs. In each pair, a TCR is matched with one experimentally confirmed binding epitope and one non-binding homologous epitope differing by only a few amino acid substitutions (92.7% with a single substitution) and originating from the same pathogen. This design ensures that the model cannot exploit global sequence dissimilarity and must instead identify binding-critical residue positions. UpTCR achieved a pairwise accuracy of 0.799, consistently outperforming DLpTCR, PanPep, and EPACT across all metrics (Supplementary Fig. S24), demonstrating its ability to discriminate biologically plausible hard negatives from true binders.

### UpTCR achieves superior performance even with missing modalities

In the above evaluations of TCR-antigen-HLA binding predictions, we utilized a complete dataset that included TCRα, TCRβ, antigen, and HLA information. However, due to technical limitations, TCRα and HLA data may not be readily available in practical scenarios. Therefore, UpTCR's ability to achieve robust performance in the absence of these modalities is of significant practical value.

**Fig. 3:**
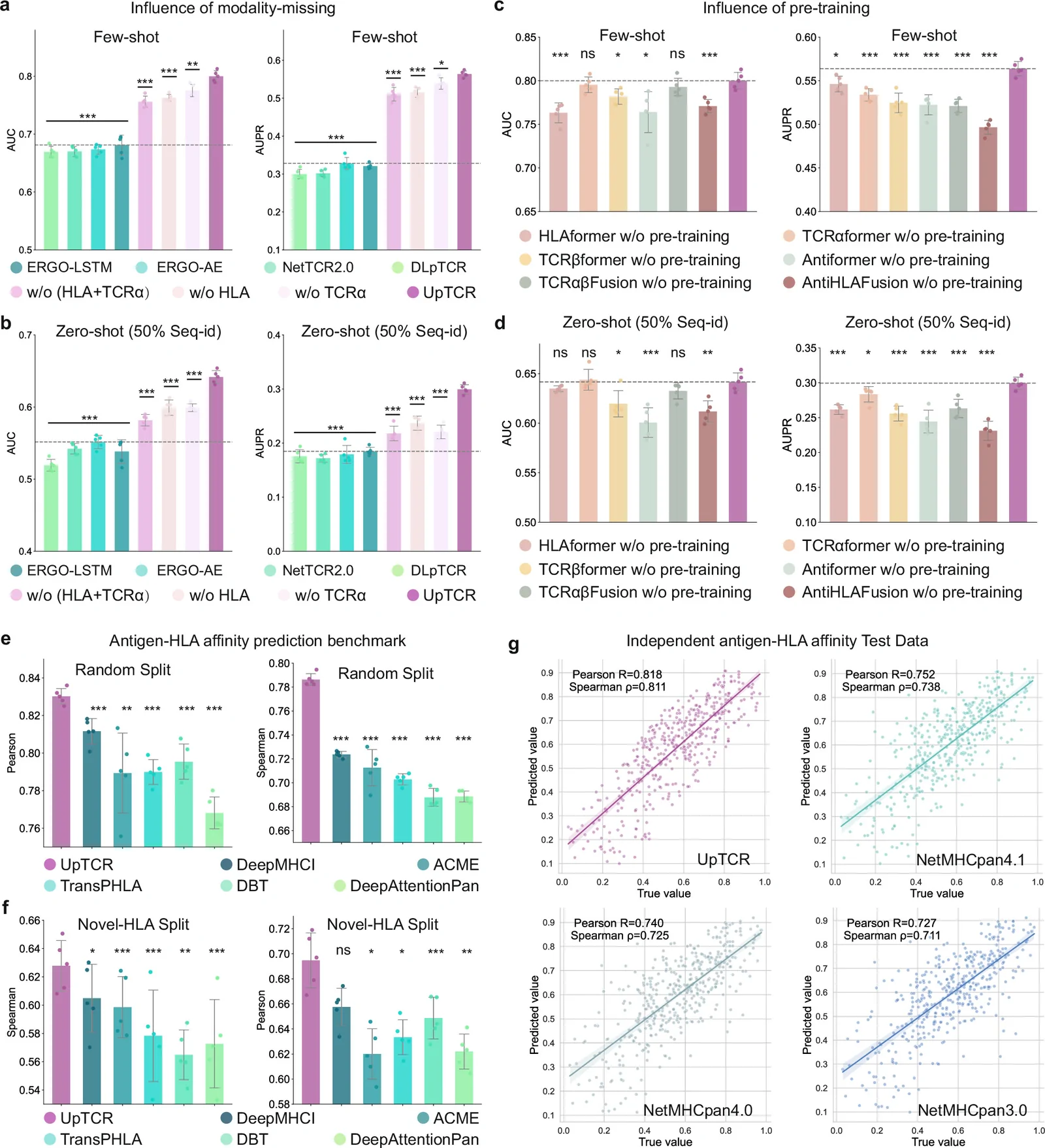
UpTCR achieves superior performance in modality-missing TCR specificity prediction and antigen-HLA affinity prediction. **a, b** Bar plots of AUC (left) and AUPR (right) when several modalities are missing for predicting TCR-antigen-HLA binding specificity under few-shot (**a**) and zero-shot (**b**) settings. Even when both HLA and TCRα are missing, UpTCR still significantly outperforms baselines. Bars represent the mean AUC and error bars indicate standard deviations. Individual data points from *n* = 5 independent runs are overlaid. **c, d** Bar plots of AUC (left) and AUPR (right) when the pretrained modules were replaced with non-pretrained versions for predicting TCR-antigen-HLA binding specificity under few-shot (**c**) and zero-shot (**d**) settings. Bars represent the mean AUC, and error bars indicate standard deviations. Individual data points from *n* = 5 independent runs are overlaid. **e, f** Bar plots of Pearson correlation coefficients (left) and Spearman correlation coefficients (right) of the baseline methods for predicting antigen-HLA binding affinity under random split (**e**) and novel-HLA split (**f**) settings. The bars represent the average metric and the error bars indicate the standard deviations. Individual data points from *n* = 5 cross-validation folds are overlaid. For **a–f**, UpTCR was compared with each variant using a two-sided unpaired Student’s t-test without multiple-comparison correction. **g** Scatter plots of predicted versus true antigen–HLA binding values on the independent test set for UpTCR, NetMHCpan4.1, NetMHCpan4.0, and NetMHCpan3.0 (*n* = 431 antigen-HLA pairs). Each point is one pair. The solid line is the linear regression, and the shaded band is the 95% confidence interval of the fitted line. For UpTCR, Pearson *r* = 0.818 (*P* = 5.8 × 10⁻¹⁰⁵). w/o, without. Other abbreviations as in Fig. 2. Exact *P* values are provided in the Source Data. *P < 0.05, **P < 0.01, ***P < 0.001, ns indicates no significant difference.

To assess this capability, we evaluated three variants of UpTCR: one missing TCRα, another missing HLA, and a third missing both TCRα and HLA. We conducted performance evaluations in both few-shot and zero-shot settings, which are critical for understanding the model’s adaptability to data scarcity. In the few-shot setting, the variants achieved AUCs of 0.784 (w/o TCRα), 0.765 (w/o HLA), and 0.748 (w/o both), representing declines of 2.0%–6.5% relative to the complete model (AUC 0.800, *P* < 0.01). In the zero-shot setting, the variants achieved AUCs of 0.598 (w/o TCRα), 0.603 (w/o HLA), and 0.585 (w/o both), representing declines of 6.1%–8.9% relative to the complete model (AUC 0.642, *P* < 0.01). This finding underscores the importance of incorporating TCRα and HLA molecule information for a comprehensive understanding of TCR-antigen-HLA interactions. Despite this decline, all three variants still outperformed baseline methods in terms of both AUC and AUPR across both settings (Fig. 3a,b). This promising result for these modality-missing variants can be attributed to the progressive pretraining module employed in UpTCR, which enables the four individual encoders and the two feature fusion modules to learn transferable information. This pretraining approach allows the model to capture essential features and relationships even when certain modalities are absent.

We further evaluated the contribution of each pretrained module by replacing it with a non-pretrained version. All variants exhibited decreased performance compared to the complete UpTCR model (Fig. 3c, d). Among these modules, ablation of the Antiformer (few-shot AUC: 0.789, a 3.5% decrease) and the AntiHLAFusionformer (few-shot AUPR: 0.553, a 5.6% decrease) resulted in the largest performance declines, highlighting the critical role of antigen-HLA information integration. Although ablation of certain individual modules (TCRαformer, HLAformer, TCRαβFusionformer) resulted in statistically indistinguishable performance degradation across some metrics and settings, this did not indicate that these modules are dispensable. This may be due to a compensatory mechanism, whereby remaining pretrained modules can partially compensate for the loss of a single component. To verify this, we performed multi-component ablation experiments in which pairs of these individually non-significant modules were simultaneously removed. Ablation of any two of these modules concurrently resulted in statistically significant performance declines compared to the full model (*P* < 0.05, Supplementary Fig. S19). Furthermore, removal of all pretraining modules led to a substantial performance decline (e.g., AUC decreased by 8.8% in the few-shot setting and 11.1% in the zero-shot setting). The above results confirm the critical importance of the pretrained modules.

To clarify the respective contributions of the two pretraining stages, we performed a stage-wise ablation study comparing four configurations: No Pretrain, Only Stage I (dimer-level pretraining), Only Stage II (trimer-level pretraining without Stage I initialization), and the complete UpTCR. In the few-shot setting, transitioning from Only Stage I to Full UpTCR yielded improvements of 4.8% in AUC and 8.0% in AUPR; in the zero-shot setting, the gains were 6.9% in AUC and 8.1% in AUPR. Both Only Stage I and Only Stage II performed significantly worse than Full UpTCR (*P* < 0.001, Supplementary Fig. S20), yet achieved comparable levels to each other (e.g., few-shot AUC: 0.752 versus 0.745), indicating that the two stages contribute complementary rather than redundant knowledge. Stage I establishes foundational molecular representations by aligning the embeddings of individual components into a shared latent space, whereas Stage II builds upon these representations to capture higher-order interaction patterns by integrating fused TCRαβ and antigen-HLA complexes through trimer-level contrastive learning.

### Accurate prediction of antigen-HLA binding affinity with UpTCR

We further evaluated UpTCR’s performance in predicting HLA-antigen binding affinity. Our assessment was conducted in two distinct settings: the random-split setting and the novel-HLA split setting. In the random-split setting, the same HLA molecules could appear in both the training and testing datasets. Conversely, in the novel-HLA split setting, the HLA molecules present in the test set were entirely absent from the training set, providing a stringent test of the model’s generalization abilities. We compared UpTCR against several baseline methods, including DeepMHCI^18^, ACME^19^, TransPHLA^20^, DBT^40^, and DeepAttentionPan^41^. In both settings, UpTCR achieved optimal performance across all five evaluation metrics (Fig. 3e, f, Supplementary Fig. S9), including AUC, AUPR, Pearson correlation, Spearman correlation, and Mean Squared Error (MSE). Note that the Pearson correlation of UpTCR significantly outperforms that of baselines. This outstanding performance can likely be attributed to the pretraining of the TCRαβ-antigen-HLA interactions, which deepened the model’s understanding of the underlying mechanisms governing antigen-HLA binding. We noted that the NetMHCpan series of methods^16,35,42^ are representative works in HLA-antigen affinity prediction. However, they have not made their source code publicly available. To ensure a fair comparison, we constructed an independent test set comprising data acquired from weekly updates between October 2021 and November 2024, which included IC_50_ binding affinity measurements sourced from the IEDB database. The overlapping data from our training data were removed. The results demonstrated that UpTCR achieved improved performance in comparison to the NetMHCpan series (Fig. 3g) while outperforming the other baseline methods (Supplementary Fig. S10). The above results highlight the robustness and effectiveness of UpTCR in predicting antigen-HLA binding affinity.

### UpTCR captures biologically meaningful and discriminative embeddings

To further investigate the effectiveness of UpTCR’s progressive pretraining strategy and its ability to capture complex biological relationships, we conducted a comprehensive visualization of UpTCR’s embeddings. This included various combinations: antigen-HLA, TCRα-antigen, TCRβ-antigen, TCRα-(antigen+HLA), TCRβ-(antigen+HLA), and (TCRα + TCRβ)-antigen. Here, Antigen+HLA and TCRα + TCRβ refer to the fused representations of the respective components.

**Fig. 4:**
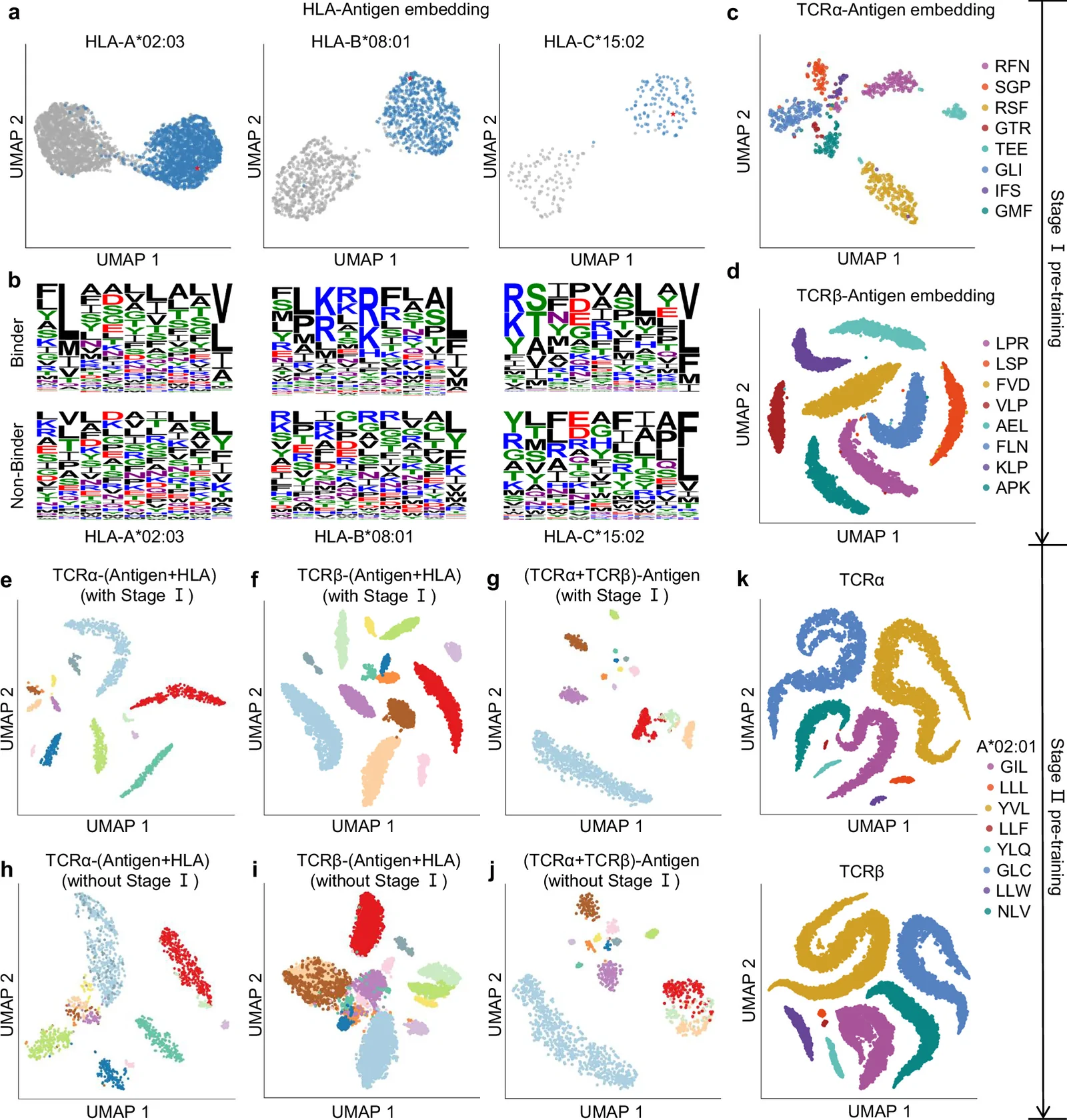
UpTCR effectively captures biologically meaningful and discriminative embeddings for TCR-antigen-HLA interactions. **a** Uniform manifold approximation and projection (UMAP) of the HLA-specific antigen embeddings, including different HLA loci, *i.e*. HLA-A*02:03 (left), HLA-B*08:01 (middle), and HLA-C*15:02 (right). Colors are defined in the key. **b** Sequence motifs of antigen binders and non-binders targeting HLA-A*02:03 (left), HLA-B*08:01 (middle), and HLA-C*15:02 (right). Both binders and non-binders exhibit substantial sequence heterogeneity across all three HLA alleles. **c** UMAP projections of the predicted antigen-specific TCRα clusters. The text labels within the clusters (e.g., RFN, SGP, RSF, GTR) represent the N-terminal trimer (the first three amino acid residues) of the corresponding antigen sequences. **d** UMAP projections of the predicted antigen-specific TCRβ clusters. The text labels within the clusters (e.g., LPR, LSP, FVD, VLP) represent the N-terminal trimer (the first three amino acid residues) of the corresponding antigen sequences. **e–g** UMAP projections of the predicted antigen-HLA-specific TCRα clusters (**e**), the predicted antigen-HLA-specific TCRβ clusters (**f**), and the predicted TCRα-TCRβ-specific antigen clusters (**g**) with the first-stage pretraining. **h–j** UMAP of the predicted antigen-HLA-specific TCRα clusters (**h**), the predicted antigen-HLA-specific TCRβ clusters (**i**), and the predicted TCRα-TCRβ-specific antigen clusters (**j**) without the first stage pretraining. **k** Comparison of antigen-HLA-specific TCRα (top) and TCRβ (bottom) clusters. The text labels within the clusters (e.g., GIL, LLL, YVL, LLF) represent the N-terminal trimer (the first three amino acid residues) of the corresponding antigen sequences.

First, we visualized the antigen-HLA embeddings, focusing on three major HLA gene loci: *HLA-A*, *HLA-B*, and *HLA-C*. We employed Uniform Manifold Approximation and Projection (UMAP)^43^ to project the embeddings of HLA molecules, HLA-binding antigens, and non-binding antigens into a two-dimensional space (Fig. 4a, Supplementary Fig. S6a, c). The results revealed a clear and interpretable clustering pattern, where HLA-binding antigens were situated closer to their corresponding HLA molecules in the embedding space, while non-binding antigens were distinctly farther. This spatial separation indicates that the model has effectively learned the discriminative features that distinguish binding from non-binding interactions. For example, in the visualization of HLA-A*02:03 (Fig. 4a left), HLA-binding antigens (blue) are tightly clustered around the HLA molecule (red), whereas non-binding antigens (gray) are dispersed farther from the HLA cluster. Similar patterns were observed for HLA-B*08:01 (Fig. 4a middle) and HLA-C*15:02 (Fig. 4a right), demonstrating the consistency and generalizability of the model’s learned representations across different HLA alleles.

We further analyzed the sequence motifs of antigen binders and non-binders targeting the different HLA alleles (Fig. 4b, Supplementary Fig. S6b, d). Both binders and non-binders exhibited considerable sequence diversity, with no specific patterns dominating either group. This observation underscores the complexity of antigen-HLA interactions and highlights the model’s ability to capture these intricate relationships without relying on simplistic sequence patterns. Next, we visualized the TCRα-antigen and TCRβ-antigen embeddings to explore the relationships between TCRs and their corresponding antigens. We selected eight distinct antigens for each group, and each antigen-binding TCR was represented using a specific color. The results revealed that TCRα embeddings recognizing the same antigen were more closely grouped together in the embedding space (Fig. 4c), indicating a correlation between TCRαs and their respective antigens. Interestingly, this pattern was more obvious in the TCRβ embeddings (Fig. 4d), emphasizing the role of the TCRβ chain in recognizing and interacting with antigens.

Subsequently, we further analyzed the embeddings of trimers in UpTCR, including TCRα-(antigen+HLA), TCRβ-(antigen+HLA), and (TCRα + TCRβ)-antigen. We selected fourteen distinct antigen+HLA or antigens for each group, and each antigen-binding TCRα, TCRβ, or TCRαβ was represented using a specific color. Overall, the TCRs’ embeddings for each antigen or antigen+HLA clustered closely (Fig. 4e–g, Supplementary Fig. S7a–c), indicating the effectiveness of the second pretraining phase. We also found that the embeddings for TCRβ-(antigen+HLA) exhibited a notably tighter clustering, underscoring the crucial role of the TCRβ chain and HLA in TCR-antigen-HLA interactions. This observation indicates that the model has effectively learned the intricate relationships between TCRβ, antigens, and HLA molecules, reflecting the biological relevance of these interactions.

Moreover, we conducted a comparative analysis to assess the impact of first-phase pretraining on the second-phase pretraining. Notably, we observed that the embeddings for all three trimers were less compact without using the first pretraining weights (Fig. 4h–j, Supplementary Fig. S7d–f). This result not only indicates the first-phase pretraining is crucial for enhancing the model’s ability to capture the complex relationships inherent in TCR-antigen-HLA interactions, but also demonstrates the effectiveness of our proposed progressive pretraining approach. We also visualized the embeddings of TCRα and TCRβ for the same set of HLA-antigen pairs to further elucidate the distinct roles of each TCR chain in antigen recognition (Fig. 4k). While both TCR chains were able to form distinct clusters based on their antigens, the TCRβ embeddings exhibited tighter clustering compared to TCRα. This finding highlights that TCRβ plays a more dominant role in the recognition of antigens presented by HLA molecules, providing further evidence of the biological relevance of the model’s learned embeddings.

### UpTCR helps investigations of TCR cross-reactivity

To investigate UpTCR's ability to predict TCR cross-reactivity, we analyzed its performance in identifying cases in which a single TCR binds to multiple antigens. Accurate prediction of TCR cross-reactivity is critical for informing therapeutic strategies and enhancing our understanding of immune responses. In this study, we collected data on 20 groups of TCR cross-reactivity (see Methods, Supplementary Table S1), with each TCR binding to multiple different antigens. For each TCR group, we additionally sampled the same number of non-binding antigens as negative controls to assess the model’s discriminative power. After performing multiple sequence alignment^44^, we identified conserved positions within the antigen sequences. A position was regarded as conserved if a specific amino acid residue appeared at that position in more than half of the sequences. The conservation ratio (CR) was calculated as the number of conserved positions relative to the total length of the multiple sequence alignment. Based on this, we categorized the TCR cross-reactivity data into two groups: the high-conservation group (CR > 0.5) and the low-conservation group (CR < 0.5).

**Fig. 5:**
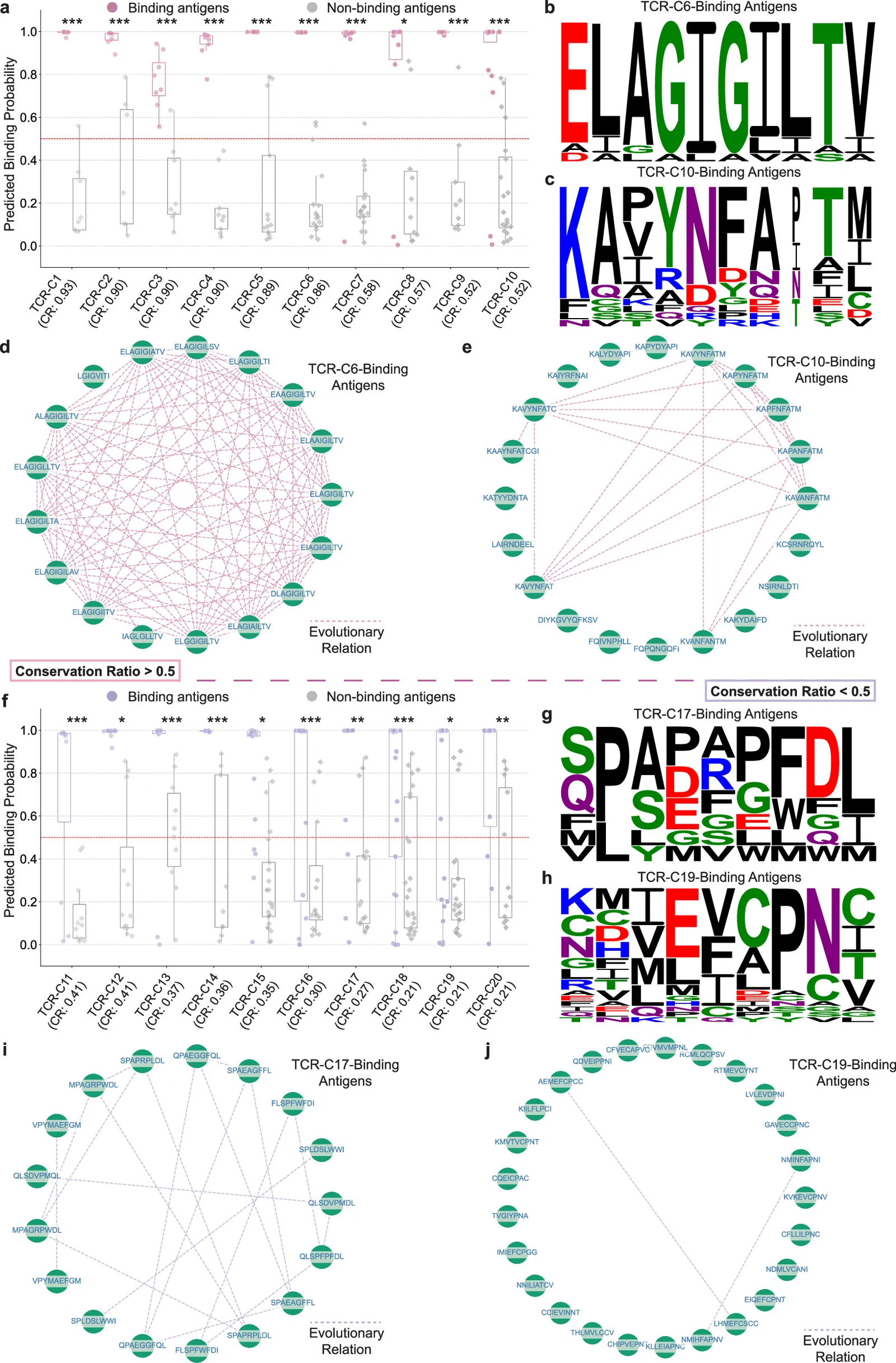
UpTCR is capable of investigating TCR cross-reactivity. **a** Predicted binding probabilities for 10 cross-reactive TCR groups (TCR-C1 to TCR-C10) in the high-conservation group (conservation ratio > 0.5). Binding and non-binding antigens are defined in the key. The conservation ratio for each group is indicated in parentheses. The red dashed line denotes the 0.5 decision threshold. Data are shown as individual points with overlaid box plots. The center line indicates the median, box bounds indicate the 25th and 75th percentiles, and whiskers indicate the minima and maxima. The unit of study is one TCR-antigen pair. Exact sample sizes (binding = non-binding) are TCR-C1 *n* = 8, C2 *n* = 7, C3 *n* = 8, C4 *n* = 9, C5 *n* = 13, C6 *n* = 17, C7 *n* = 19, C8 *n* = 10, C9 *n* = 9 and C10 *n* = 20. Bi*n*ding versus non-binding probabilities in a were compared with a two-sided Mann-Whitney U test (unadjusted). **b, c** Sequence motifs of the antigens targeting TCR-C6 (**b**) and TCR-C10 (**c**). **d, e** Sequence homology networks for antigens targeting TCR-C6 (d) and TCR-C10 (**e**). Nodes represent individual antigen sequences and edges connect sequences with a Levenshtein distance <3, indicating high sequence similarity. **f** Predicted binding probabilities for 10 cross-reactive TCR groups in the low-conservation group. Data are shown as individual points with overlaid box plots. The center line indicates the median, box bounds indicate the 25th and 75th percentiles, and whiskers indicate the minima and maxima. Exact sample sizes are TCR-C11 *n* = 12, C12 *n* = 10, C13 *n* = 9, C14 *n* = 25, C15 *n* = 16, C16 *n* = 17, C17 *n* = 25, C18 *n* = 25, C19 *n* = 12 and C20 *n* = 20. Binding versus *n*on-binding probabilities were compared with the two-sided Mann-Whitney U test (unadjusted). The unit of study is one TCR-antigen pair. Binding and non-binding antigens are defined in the key. **g, h** Sequence motifs of the cross-reactive antigens targeting TCR-C17 (**g**) and TCR-C19 (**h**). **i, j** Sequence homology networks for antigens targeting TCR-C17 (i) and TCR-C19 (j). The sparse connectivity in the low-conservation group reflects high sequence diversity among cross-reactive antigens. Exact *P* values are provided in the Source Data. *P < 0.05, **P < 0.01, ***P < 0.001.

In the high-conservation group, UpTCR significantly discriminated against cross-reactive binding antigens from non-binding antigens across all 10 TCR groups (*P* < 0.05 for all comparisons, Fig. 5a). The mean predicted binding probability for confirmed cross-reactive antigens was 0.927, compared with 0.245 for non-binding antigens (a 3.79-fold difference), yielding an average classification accuracy of 90.4% at the 0.5 decision threshold. This high success rate may be attributed to the presence of highly conserved residues among the antigens (Fig. 5b, c), which often exhibit significant sequence homology (Fig. 5d, e). The sequence homology^45^ is defined based on the Levenshtein Distance, which quantifies the minimum number of single-residue changes required to transform one sequence into another. Antigens with a Levenshtein Distance of less than 3 were considered to be sequence-similar peptides. This ability can be attributed to the soft contrastive learning during pretraining, which enables the model to discern intra-modal sequence relationships and identify binding potential among sequence-similar antigens.

In the low-conservation group, UpTCR also achieved significant discrimination for all 10 TCR groups (*P* < 0.05, Fig. 5f), with a mean predicted probability of 0.790 for binding antigens versus 0.316 for non-binding antigens (2.5-fold difference) and an average accuracy of 76.9%. As expected, the discriminative margins were smaller than those of the high-conservation group (*P* < 0.01, Supplementary Fig. S11), consistent with the higher sequence diversity in motifs (Fig. 5g, h) and more complex evolutionary relationships (Fig. 5i, j). Despite these challenges, UpTCR maintained statistically significant discrimination across all groups, indicating its effectiveness in handling variable antigen sequences. This ability may be attributed to the progressive pretraining on dimer and trimer data, which allows UpTCR to fully leverage all available interaction information and enhances its generalization ability.

### UpTCR uncovers meaningful patterns of any pairwise residue interaction

Understanding the residue-level interactions within the TCR-antigen-HLA complex is paramount for elucidating the structural determinants of T cell-mediated immune responses^46^. While previous methods have attempted to reveal certain pairwise contacts^30,31^, such as CDR3β-antigen and antigen-HLA, these analyses were often limited to the CDR3β region alone, thereby failing to provide comprehensive structural insights into the full-length TCRβ and TCRα. Moreover, a complete view encompassing all six pairwise contact relationships among the four components (TCRα, TCRβ, antigen, HLA) has remained elusive.

Due to UpTCR’s unique architecture, which models full-length TCRα and TCRβ and integrates them into the complete TCRα-TCRβ-antigen-HLA tetramer, it has the potential to comprehensively predict these complex interactions. To achieve this, we introduced the interaction conformation prediction module, which forces the model to predict the residue-level contact maps between all four components. This module works by first calculating the outer product of the learned residue-level embeddings from corresponding encoders to form a pairwise feature matrix. This matrix is then processed by a convolutional neural network to predict the continuous spatial distance between residue pairs. We finetuned and cross-validated UpTCR using 164 experimentally determined TCR-antigen-HLA complex structures as ground truth. To further expand the structural training data, we constructed an augmented variant, UpTCR+AF, by incorporating 1,037 high-confidence AlphaFold-predicted TCR-antigen-HLA complex structures filtered by stringent predicted Template Modeling (pTM) and interface predicted Template Modeling (ipTM) thresholds (pTM > 0.8 and ipTM > 0.8, Supplementary Fig. S21). For visualization and interpretation, the predicted continuous distance is mapped to a normalized interaction score (ranging from 0 to 1) using a sigmoid function, where a higher score indicates a closer distance and thus a higher probability of physical contact.

**Fig. 6:**
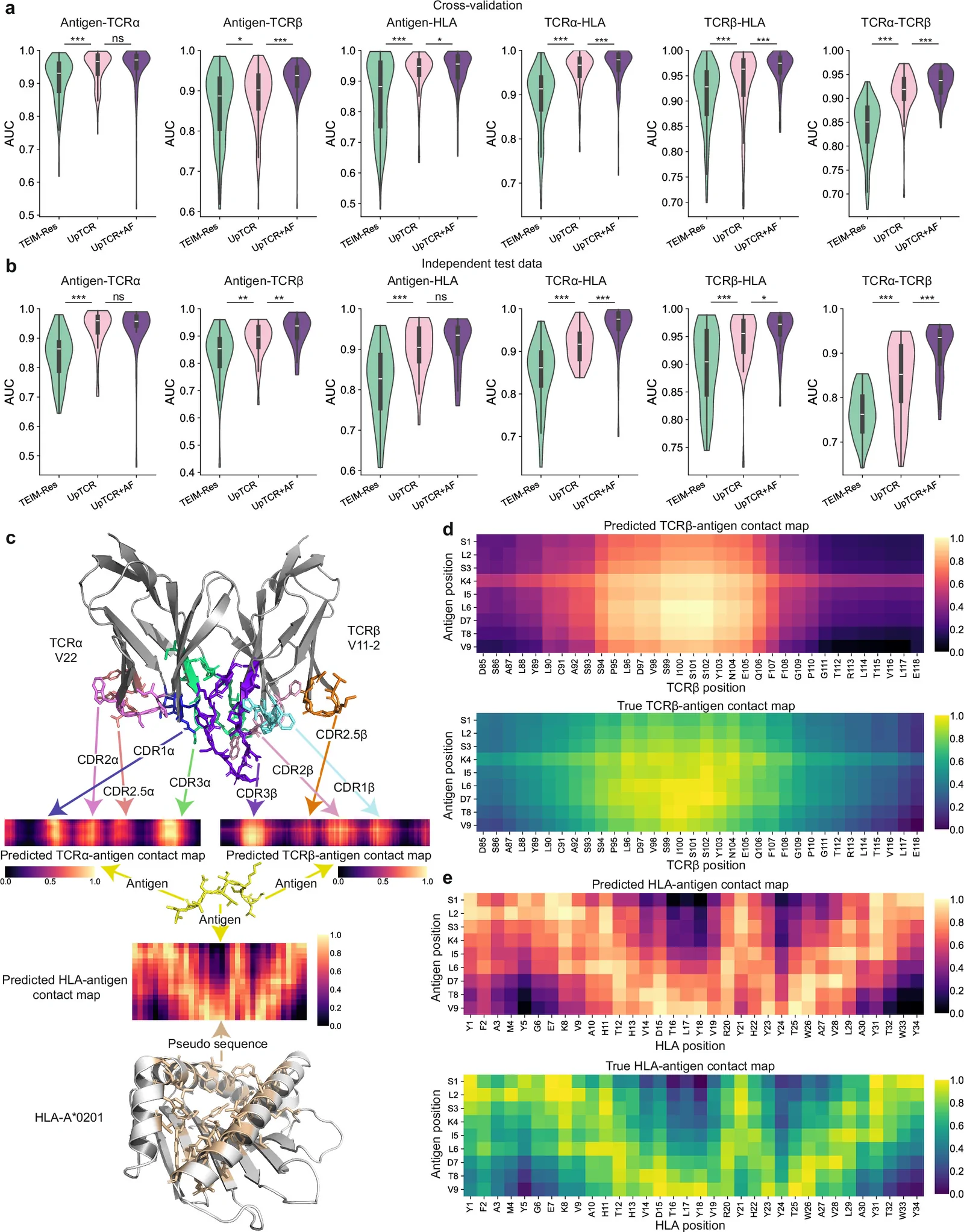
UpTCR facilitates investigations of any pairwise residue contact relationship among full-length TCRα, full-length TCRβ, Antigen, and HLA. **a, b** Violin plots displaying the cross-validation (**a**) and independent test (**b**) AUC for predicting residue-level contact maps between any pairwise components by TEIM-Res, UpTCR, and UpTCR+AF. The unit of study is one TCR–antigen–HLA complex. *n* = 164 crystal structures in (**a**) and *n* = 39 held-out structures in (**b**). Pairwise comparisons among TEIM-Res, UpTCR, and UpTCR+AF were performed using a two-sided Mann-Whitney U test (unadjusted). The violin shows the data distribution; the center line indicates the median, the box bounds the 25th and 75th percentiles, and the whiskers indicate the minima and maxima. From left to right: antigen-TCRα, antigen-TCRβ, antigen-HLA, TCRα-HLA, TCRβ-HLA, and TCRα-TCRβ. UpTCR+AF denotes UpTCR further trained with additional high-confidence AlphaFold-predicted complex structures. pTM, predicted template modeling score; ipTM, interface predicted template modeling score. The independent test set comprises 39 TCR-antigen-HLA complex structures that were excluded from all stages of pretraining and fine-tuning. **c** Representative TCR-antigen-HLA complex structure (PDB ID: 6R2L) with full-length TCRα-antigen and TCRβ-antigen predicted contact maps. The antigen peptide (yellow, stick representation) is shown in the binding groove of the HLA molecule (gray). The color scale represents the normalized interaction score (0 to 1), where higher values indicate closer predicted spatial distances and greater contact probability. **d** Zoomed-in residue-level contact maps with amino acid identities and residue indices for CDR3β-antigen interactions, comparing UpTCR predictions (top) with experimentally determined ground truth (bottom). **e** Zoomed-in residue-level contact maps with amino acid identities and residue indices for HLA-antigen interactions, comparing UpTCR predictions (top) with experimentally determined ground truth (bottom). Exact *P* values are provided in the Source Data. *P < 0.05, **P < 0.01, ***P < 0.001, ns indicates no significant difference.

We evaluated contact prediction performance using both binary classification (AUC) and continuous distance regression (Pearson correlation). In cross-validation on the 164 crystal structures, UpTCR significantly outperformed TEIM-Res^27^ across all six pairwise interaction types in both AUC (*P* < 0.05, Fig. 6a) and Pearson correlation (*P* < 0.05, Supplementary Fig. S22). UpTCR+AF further improved upon UpTCR in most interaction types, demonstrating the benefit of augmenting the limited crystal structure data with high-confidence computational models. Notably, the improvement was most pronounced for TCRα-TCRβ contact map predictions (Pearson: TEIM-Res 0.705 versus UpTCR 0.860 versus UpTCR+AF 0.873), reflecting the capacity of additional structural data to capture the complex chain-pairing interface.

To rigorously assess the model’s generalizability, we curated an independent test set of 39 TCR-antigen-HLA complex structures, which were not used in any stage of pretraining and finetuning. On this held-out set, UpTCR consistently outperformed TEIM-Res across all six interaction types (Fig. 6b), confirming the model’s ability to generalize to unseen structural data. UpTCR+AF achieved the highest performance on the independent test set, with a mean AUC improvement of 3.1% over UpTCR across all tasks.

We further presented a representative case (PDB ID: 6R2L) to exemplify UpTCR’s ability in elucidating interactions within the TCR-antigen-HLA complex (Fig. 6c). The antigen peptide first binds to the HLA molecule to form an antigen-HLA complex and is then presented on the cell surface. Subsequently, T cells recognize the HLA-antigen complex through their surface TCRs, with CDR3 playing a dominant role in the specific recognition of the antigen peptide, while CDR1, CDR2, and CDR2.5 primarily interact with the α-helical domains of the HLA molecule. Our results demonstrated that UpTCR can effectively predict the contact maps across all six pairwise interactions, including TCRα-TCRβ, TCRα-HLA, TCRβ-HLA, TCRα-antigen, TCRβ-antigen, and antigen-HLA (Supplementary Figs. S12–S17). To illustrate the residue-level specificity captured by UpTCR, we generated zoomed-in contact maps for CDR3β-antigen and HLA-antigen interactions with amino acid identities and residue indices (Fig. 6d, e). In the CDR3β-antigen contact map, residues S99, I100, S101, and S102 of TCRβ exhibited strong predicted contacts with the antigen peptide, consistent with their spatial proximity to the bound peptide in the crystal structure. In the HLA-antigen contact map, each antigen residue displayed a distinct contact profile with specific HLA residues lining the binding groove, reflecting the position-dependent anchoring of the peptide within the HLA binding cleft. Notably, the predicted contact patterns aligned with the experimentally determined ground truth, correctly identifying key interacting residue pairs across both interfaces.

### Translation validation of UpTCR in breast cancer across different subtypes

**Fig. 7:**
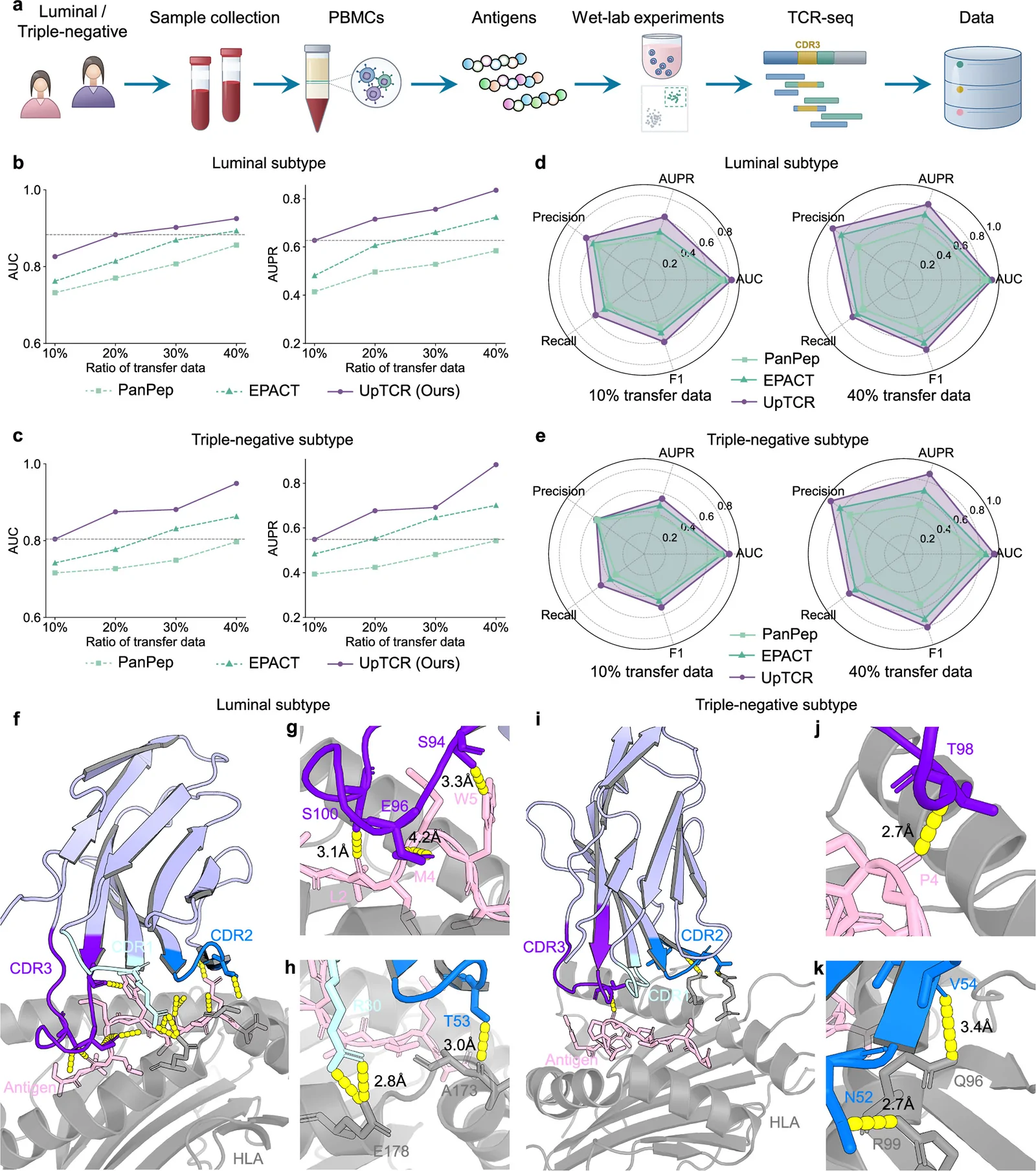
Translational validation of UpTCR for antigen-stimulated T cell responses in breast cancer patients across different molecular subtypes. **a** Schematic of the validation experiment. Samples were obtained from peripheral blood mononuclear cells (PBMCs) of Luminal and triple-negative breast cancer (TNBC) patients. Thirty-two tumor-associated antigen-derived peptides (e.g., New York esophageal squamous cell carcinoma 1 (NY-ESO-1), mammaglobin-A (Mam-A)), highly expressed in breast cancer, were used to stimulate T cells. This identified the direct correspondence between antigen identity and TCR sequence, creating a breast cancer-specific TCR database. **b, c** AUC (left) and AUPR (right) performance of UpTCR, EPACT, and PanPep in predicting TCR binding specificity across the Luminal (**b**) and TNBC (**c**) subtypes with increasing ratios of transfer data (10%-40%). UpTCR consistently outperforms both EPACT and PanPep across all transfer data ratios. **d, e** Radar plots quantitatively comparing UpTCR, EPACT, and PanPep across five key metrics (AUC, AUPR, Precision, Recall, and F1-score) for the Luminal (**d**) and TNBC (**e**) subtypes at 10% (left) and 40% (right) transfer data ratios. UpTCR dominates across all metrics in both subtypes, with the advantage being most pronounced in the low-data regime (10%). **f–h** Representative AlphaFold3-predicted TCR–antigen–HLA complex structures for the Luminal subtype. The left panel (**f**) displayed the overall architecture with annotated CDR regions (CDR1, CDR2, CDR3) of the TCRβ chain (ipTM: 0.8, pTM: 0.82). The HLA molecule is shown in gray, the antigen peptide in pink (stick), and the TCR in purple/blue (cartoon). Right panels (**g, h**) present zoomed-in views of key molecular interfaces: CDR3β–antigen contacts (**g**) and CDR1/CDR2β–HLA contacts (**h**), with specific interacting residues labeled by amino acid identity, residue number, and distance. Hydrogen bonds are highlighted by yellow dashed lines. **i–k** Representative AlphaFold3-predicted TCR-antigen-HLA complex structure for the TNBC subtype, presented in the same format as **f–h** (ipTM: 0.89, pTM: 0.87). The overall complex architecture (**i**) is shown with CDR annotations, accompanied by zoomed-in views of CDR3β-antigen contacts (**j**) and CDR2β–HLA contacts (**k**), with interacting residues and distances annotated as described above.

To assess UpTCR’s real-world applicability, we evaluated UpTCR’s transfer ability on experimentally derived novel TCR-antigen pairs obtained from breast cancer patients with distinct subtypes, i.e., Luminal and triple-negative breast cancer (TNBC). We performed antigen-stimulated T cell response assays using peripheral blood mononuclear cells (PBMCs) from breast cancer patients (Fig. 7a). Thirty-two tumor-associated antigen-derived peptides, such as NY-ESO-1 and Mam-A, known to be highly expressed and immunogenic in breast cancers, were used to stimulate T cells. After experimental validation, a breast cancer-specific TCR database was created for downstream validation. The final curated dataset comprises the following interactions: 1) Luminal Subtype (709 positive pairs and 3500 negative control pairs) and 2) TNBC subtype (489 positive TCR-antigen pairs and 2500 negative control pairs).

We benchmarked UpTCR against PanPep and EPACT, two competitive methods known for their transferability with limited training data, under varying amounts of transfer data (10% to 40%). In both Luminal (Fig. 7b) and TNBC (Fig. 7c) cohorts, UpTCR consistently achieved superior performance in both AUC and AUPR under various transfer data. Notably, UpTCR reached high AUPR using only 10% of transfer data, whereas PanPep required 40% to achieve comparable performance (Fig. 7b, c, right). Further comprehensive quantitative evaluation (Fig. 7d, e) demonstrated UpTCR’s superiority across multiple key metrics, including Precision, Recall, AUC, AUPR, and F1-score in both breast cancer subtypes at different transfer data ratios. This highlights UpTCR’s robustness and efficiency in few-shot samples, which is critical for personalized immunotherapy applications where patient-specific data are often scarce.

To provide structural insights into the predicted TCR-antigen-HLA interactions, we employed AlphaFold3 to model representative high-confidence TCR-antigen-HLA complex structures. For the Luminal subtype (Fig. 7f), the predicted structure revealed a canonical TCR binding orientation, with CDR3β positioned centrally over the antigen peptide and CDR1β/CDR2β engaging the HLA α-helices. Zoomed-in views of the CDR3β-antigen interface (Fig. 7g) identified specific interacting residue pairs, including contacts between S94, E96, and S100 of TCRβ and residues L2, M4, and W5 of the antigen peptide, with inter-atomic distances ranging from 3.1 Å to 4.2 Å. At the CDR1β/CDR2β-HLA interface (Fig. 7h), residues R30 and T53 of TCRβ formed hydrogen bonds with A173 and E178 of the HLA. For the TNBC subtype (Fig. 7i–k), a similar pattern was observed, with CDR3β residue T98 contacting antigen residue P4 at a distance of 2.7 Å (Fig. 7j), and CDR2β residues N52 and V54 forming hydrogen bonds with HLA residue R99 and Q96 (Fig. 7k). These observations demonstrate that UpTCR’s high-confidence predictions correspond to biologically plausible binding conformations with molecular contacts.

### Prospective validation of UpTCR in predicting cross-reactivity

**Fig. 8:**
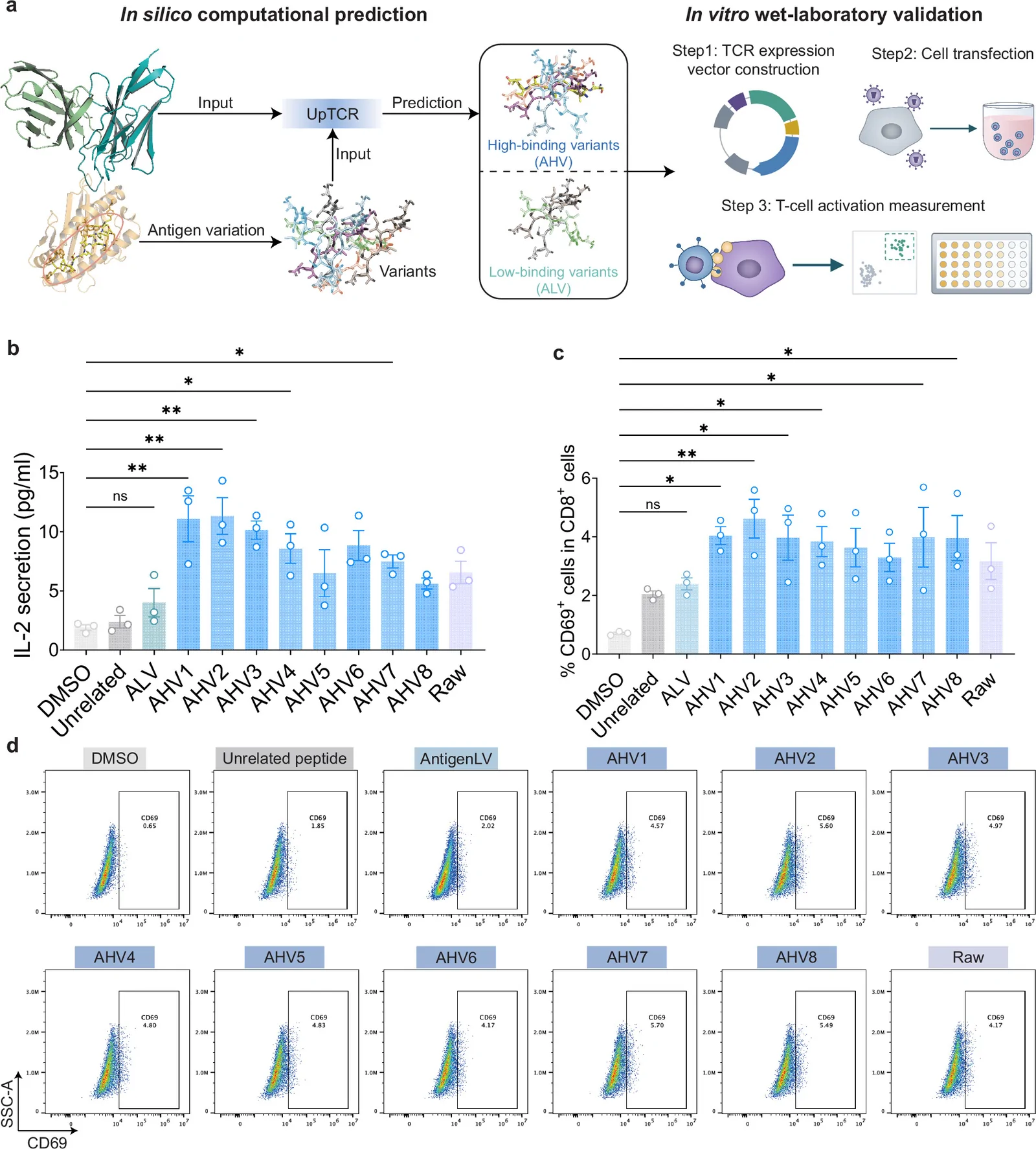
Prospective validation of TCR engagement with antigen variants using UpTCR. **a** Schematic overview of the computational prediction and experimental validation workflow. Starting from the native Melan-A/MART-1 epitope, antigen variants were generated and computationally screened for binding to the cognate TCR using UpTCR. Predicted high-binding variants (AHV1–AHV8) and a predicted low-binding variant (AntigenLV), together with the native peptide (Raw), DMSO, and an unrelated peptide control, were selected for peptide synthesis and functional validation in TCR-expressing Jurkat cells. **b** IL-2 secretion measured by ELISA after 24 h stimulation with DMSO, unrelated peptide, AntigenLV, AHV1-AHV8, or the native peptide (Raw) (*n* = 3 independent batches). Overall difference was assessed by a Kruskal-Wallis test, followed by uncorrected two-sided Dunn’s tests versus DMSO. **c** Frequency of CD69⁺ cells measured by flow cytometry after 24 h stimulation with the indicated peptides or controls (*n* = 3 independent batches). Overall difference was assessed by ordinary one-way ANOVA, followed by Tukey’s post hoc tests versus DMSO. **d** Representative flow cytometry plots showing CD69 expression following stimulation with DMSO, unrelated peptide, AntigenLV, AHV1–AHV8, or the native peptide (Raw). Tick labels are shown on all plots, with decade labeling on fluorescence axes. AHV predicted high-binding antigen variant, AntigenLV / ALV predicted low-binding variant, Raw native Melan-A peptide, DMSO dimethyl sulfoxide, IL-2 interleukin-2, CD69 early T-cell activation marker. Data are presented as mean ± SD. Exact *P* values are provided in the Source Data. *P < 0.05, **P < 0.01, ***P < 0.001, ns indicates no significant difference.

Prospective validation is essential for establishing the real-world applicability of computational models in immunology, particularly for predicting T cell receptor (TCR) cross-reactivity. While retrospective analyses based on curated cross-reactivity datasets provide valuable evidence, such datasets often aggregate data from heterogeneous experimental systems and may contain assay-dependent definitions of reactivity. To evaluate UpTCR’s ability to predict TCR recognition of antigenic variants in a unified and controlled experimental setting, we designed a combined in silico and experimental workflow focusing on variants derived from the melanoma-associated antigen Melan-A/MART-1 (Fig. 8a).

Starting from the native Melan-A decamer (EAAGIGILTV), we constructed a comprehensive in silico variant library by systematically introducing amino acid substitutions, insertions, and deletions across peptide positions, thereby generating variants spanning a range of peptide lengths and sequence identities. For edit distances 1-2, where the combinatorial space is tractable, all possible variants were exhaustively enumerated. For edit distances 3-5, we uniformly sampled 20,000 variants per level to ensure broad coverage while maintaining computational tractability, yielding a total library of approximately 110,000 variants. For each candidate, we predicted the binding probability using UpTCR and ranked the variants by predicted score. From the ranked list, we selected eight predicted high-binding variants (AHV1-AHV8) spanning a wide range of sequence identity to the native peptide (40%-80%) and one predicted low-binding variant (AntigenLV, 60% identity) for experimental validation (Table S2). Notably, several predicted high-binding variants (AHV4-AHV8) share substantially lower sequence identity (40%-54.5%) with the native epitope, providing a stringent evaluation of the model’s generalization capability.

To establish the experimental system, Jurkat T cells were engineered to express CD8, as parental Jurkat cells are CD4^+^CD8^-^. Flow cytometry confirmed robust CD8 expression in transduced cells compared with parental Jurkat cells (Supplementary Fig. S26). The cognate TCR was then introduced into these CD8-expressing Jurkat cells for functional validation. To further benchmark assay specificity, we included a literature-reported Melan-A-specific MEL8 TCR^47^ as a positive control. MEL8 TCR-expressing cells responded significantly to the native Melan-A peptide (Supplementary Fig. S27), confirming the reliability and specificity of the experimental system.

We synthesized the corresponding peptides and co-cultured them at a 1:1 ratio with overnight-pulsed HLA-A*02:01 A375 cells and the TCR-expressing CD8^+^ Jurkat cells. Functional T cell activation was assessed by measuring IL-2 secretion and CD69 expression, an early activation marker, after 24 h of stimulation. DMSO and unrelated peptide controls were included in each assay. Consistent with the computational predictions, the predicted high-binding variants AHV1-AHV8 induced higher IL-2 secretion than the predicted low-binding variant AntigenLV and unrelated peptide controls (Fig. 8b). Similarly, flow cytometry analysis showed an increased proportion of CD69^+^ cells upon stimulation with AHV1-AHV8, whereas AntigenLV, unrelated peptide, and DMSO controls induced minimal activation (Fig. 8c, d). Importantly, the low-identity variants also elicited T cell activation, supporting the ability of UpTCR to identify TCR-recognized peptide variants beyond near-canonical sequence similarity. These findings collectively validate UpTCR’s proficiency in not only predicting TCR-antigen binding specificity but also in identifying antigenic variants capable of modulating T cell recognition. By integrating computational prediction with targeted experimental validation, UpTCR provides a powerful and extensible platform for the rational design of antigen variants and personalized immunotherapies. This capability is valuable for dissecting TCR cross-reactivity landscapes, optimizing vaccine epitopes, and mitigating potential off-target effects in T cell-based therapies.

## Discussion

In this work, we have introduced UpTCR, a unified progressive pretrained foundation model for T-cell receptor (TCR)-antigen-HLA binding recognition, bringing forth multiple technical advancements that collectively address critical challenges in computational immunology. Compared to existing methods, UpTCR offers several key advantages that enhance both its predictive performance and practical applicability in understanding adaptive immune responses. 1) Comprehensive and multi-scale modeling of full-length TCRs. UpTCR employs holistic modeling of the full-length TCR, incorporating not only the dominant CDR3 loops but also the complementary variable regions (CDR1, CDR2, and CDR2.5) and the framework regions. This comprehensive representation captures the multifaceted nature of TCR binding, reflecting the biological reality where multiple segments contribute synergistically to antigen-HLA recognition. The multi-component and stage-wise ablations confirm that these modules function synergistically and are collectively indispensable. Furthermore, UpTCR integrates multi-scale features, including primary sequence embeddings, evolutionary profiles, structural insights extracted via pretrained large language models, and CDR3-specific motif representations. This rich feature integration allows the model to grasp both global and fine-grained patterns within TCR sequences, effectively bridging sequence-level information with structural-functional understanding. 2) Leveraging incomplete data through progressive pretraining. A major limitation in modeling TCR-antigen-HLA interactions is the scarcity of complete tetrameric datasets, as experimental acquisition of paired TCRα, TCRβ, antigen, and HLA data remains technically and financially challenging. To overcome this, UpTCR adopts a progressive soft contrastive pretraining strategy that capitalizes on abundant dimeric (pairwise) and trimeric (three-component) interaction data. This incremental training approach enables UpTCR to learn transferable knowledge from simpler interaction pairs, progressively integrating them into a robust tetrameric representation. The ability to maintain competitiveness even when crucial modalities such as TCRα or HLA are missing demonstrates UpTCR’s practicality for real-world datasets, which often suffer from such incompleteness. 3) Soft contrastive learning to address biological complexity and cross-reactivity. Traditional contrastive learning approaches tend to treat all non-interacting pairs as hard negatives, which can introduce false negatives and hinder model robustness—particularly detrimental when modeling TCR cross-reactivity, where a single TCR can bind multiple related or even distinct antigens. UpTCR innovatively incorporates soft contrastive learning, leveraging intra-modal similarity to soften labels on negative samples based on sequence homology or binding affinity information. This nuanced learning paradigm mitigates over-penalization of biologically plausible interactions and enriches the model’s capacity to reflect the nuanced spectrum of TCR specificity and cross-reactivity. To further probe generalizability, we evaluated UpTCR on an independent HLA-B*27:05-restricted autoantigen dataset reported by Wang et al.^48^. In a zero-shot setting, UpTCR achieved the highest performance among competing methods (Supplementary Fig. S28), indicating promising generalization ability. Experimental validations on cross-reactive TCRs further confirm UpTCR’s ability to capture these complex immunological relationships, which are instrumental for applications in vaccine design and cancer immunotherapy.

Beyond TCR specificity prediction, UpTCR excels in predicting antigen-HLA binding affinities with competitive or superior performance compared to state-of-the-art affinity predictors. This dual functionality underscores the model’s deep understanding of the fundamental components in T cell immunity and places it as a versatile tool for epitope discovery and immunogenicity prediction. Crucially, UpTCR provides residue-level contact maps across all pairwise interactions within the TCRα-TCRβ-antigen-HLA tetramer. Unlike prior methods limited to CDR3β or to partial contacts, UpTCR elucidates the complete network of molecular contacts, including interactions between CDR1 and CDR2 and HLA molecules, and the interplay between TCRα and TCRβ chains. Such detailed structural insights afford mechanistic interpretations of TCR recognition and cross-reactivity, enabling rational design of modified TCRs or peptide vaccines with fine-tuned specificity and reduced off-target effects.

Importantly, UpTCR’s utility was demonstrated through prospective validation across melanoma antigen variants and breast cancer patient cohorts. By accurately screening variant peptides for TCR engagement and corroborating predictions with functional assays of IL-2 secretion and CD69 upregulation, UpTCR proves to be a potent platform for personalized immunotherapy design. Moreover, its ability to achieve robust performance with limited transfer-learning data makes it especially valuable where patient-specific TCR data are sparse, a common bottleneck in clinical translation. We acknowledge that certain limitations remain to be addressed. First, the curated cross-reactivity dataset used in the retrospective analysis aggregates reactivity annotations derived from heterogeneous experimental systems, including T-cell lines and primary T cells, which may introduce assay-dependent biases. Although UpTCR achieved high prediction accuracy in this setting and its cross-reactivity predictions were prospectively validated using a unified experimental assay, extending such controlled validation to a broader panel of TCRs across diverse HLA restrictions and assay platforms remains an important direction for future work. Second, although the generalizability of UpTCR across diverse HLA restrictions has been supported by our computational evaluations, future wet-lab validation using additional non-HLA-A2-restricted TCR systems would further strengthen the experimental evidence for its pan-specific applicability. To foster transparency, reproducibility, and community-driven improvements, we have made the UpTCR framework publicly available as an open-source resource. This includes the pretrained models, training pipelines, and relevant datasets, enabling researchers to extend and adapt the platform to diverse immunological questions. We anticipate that this collaborative effort will accelerate methodological advancements and facilitate the translation of computational insights into clinical immunotherapy applications.

In summary, UpTCR represents a unified breakthrough in decoding the complex interaction grammar of adaptive immunity. By integrating multi-scale molecular representations, progressive training on incomplete datasets, and soft contrastive learning, UpTCR transcends previous limitations and enables more accurate, generalizable, and interpretable modeling of TCR-antigen-HLA interactions. This foundation model not only accelerates fundamental immunological research but also paves the way toward the development of safe, effective, and precision-targeted T cell-based therapeutics.

## Methods

### Ethics

This study complies with all relevant ethical regulations. All experiments were approved by the ethics committee and institutional review board at Jiangnan University. Written informed consent was obtained from all participants. Five adults with clinically diagnosed breast cancer were enrolled (three Luminal subtype and two TNBC). Consecutive eligible patients at the participating clinical center were enrolled after informed consent, and peripheral blood was collected; enrollment was not based on predicted TCR repertoire. Sex and/or gender were not used as an inclusion criterion, were not stratified, and were not analyzed as a covariate because the cohort is too small for a sex-disaggregated analysis. How sex was determined was not recorded. Age was not collected. No additional compensation was provided.

### Data and preprocessing

#### TCRαβ-antigen-HLA pretraining and finetuning data

We first collected all related data from VDJdb^45^, PIRD^49^, McPAS-TCR^50^, and IEDB^51,52^, and 10X Genomics^53^ databases. To ensure data quality and biological relevance, we applied the following inclusion and curation criteria: 1) Species and MHC Class: We constructed a pan-specific dataset focusing on MHC Class I interactions. The data primarily consists of Human (HLA) entries, supplemented with Mouse (H-2) data. This multi-species strategy leverages the structural conservation of the MHC binding groove to enhance the model’s ability to learn generalized residue interaction patterns. 2) Confidence Filtering: For entries derived from VDJdb, we filtered out low-confidence records, retaining only those with a confidence score ≥ 1. 3) De-duplication and Bias Correction: We removed duplicate entries where the TCRα, TCRβ, peptide, and HLA alleles were identical. To address the known overrepresentation of specific peptides in high-throughput single-cell datasets (e.g., the CMV peptide KLGGALQAK in 10x Genomics data), we removed redundant clonotypes to prevent model bias toward these specific epitopes. 4) Collection Timeframe: The data collection and curation were completed in November 2024, incorporating all available entries up to that date. Then, we categorized the data into different combinations based on the status of the missing modality (Supplementary Fig. S1). This classification included various groups, i.e., TCRα-antigen (857), TCRβ-antigen (61,524), antigen-HLA (41,903), TCRα-TCRβ-antigen (2710), TCRβ-antigen-HLA (70,971), TCRα-antigen-HLA (25,323), and TCRα-TCRβ-antigen-HLA (36,061).

To rigorously prevent data leakage and ensure an unbiased assessment of our model’s generalization capabilities, we performed a stringent de-redundancy step on the incomplete data utilized during the pretraining phase. We employed the MMseqs2^54^ tool to quantify the sequence similarity between the entire pretraining corpus and the final test datasets. A pretraining entry was excluded if any component shared greater than 30% sequence identity with the downstream test data (antigen sequence identity > 30%, TCRα CDR identity > 30%, or TCRβ CDR identity > 30%). This uniform threshold was empirically justified: the median pairwise identities among functionally related sequences (same-HLA binders: 0.444; same-antigen-HLA TCRα: 0.389; TCRβ: 0.471; Supplementary Fig. S23) all fall above 30%, confirming that this threshold effectively eliminates the majority of potentially confounding homologous pairs. Following this stringent sequence-based filtration, the refined, non-redundant pretraining dataset comprised the following entries: TCRα-antigen (291), TCRβ-antigen (39,768), antigen-HLA (26,083), TCRα-TCRβ-antigen (326), TCRβ-antigen-HLA (43,382), and TCRα-antigen-HLA (12,124).

The six groups of paired data for pretraining were divided into training and validation sets in a 9:1 ratio to maximize use of the available data. For the TCRα-TCRβ-antigen-HLA binding prediction, we implemented four different splitting strategies: 1) Majority learning, where the majority of relevant data for each antigen is present in the training set. 2) Few-shot learning, where only a limited amount of relevant data for each antigen is available in the training set. 3) Zero-shot learning with 50% sequence identity filtering, where none of the antigens in the test set appeared in the training set, and all sequences in the training data maintained a sequence identity of less than 50% with the sequences in the test set. 4) Zero-shot learning with 30% sequence identity filtering, applying a more stringent threshold to further eliminate potential information leakage through distantly homologous sequences. Negative samples were generated by a commonly used shuffling strategy, i.e., positive TCRs were randomly shuffled and paired with non-cognate antigen-HLA complexes to generate a large number of presumed negative interactions. The ratio of positive to negative samples in the evaluation sets was approximately 1:5. In addition, to assess the model’s ability to discriminate biologically plausible non-binders from true binders, we constructed a hard negative evaluation benchmark from the VDJdb database. For each TCR in the dataset, we identified pairs of epitopes where one peptide is a confirmed binder and the other is a homologous non-binder from the same pathogen species, differing by only 1-4 amino acid substitutions. Contradictory entries were excluded. The final benchmark comprised 3,495 stratified pairs covering 74 unique epitopes, 19 HLA alleles, and 2,853 unique TCRs. The majority of pairs (92.7%) differed by a single amino acid substitution. For evaluation, both the binding and non-binding epitopes within each pair were independently scored by each model, and pairwise accuracy (the proportion of pairs where the binding epitope received a higher predicted score), mean score margin, and AUC were computed. Details of data preprocessing can be found in Supplementary Notes 1,2. To further assess the generalizability of UpTCR, we evaluated it on an independent dataset from Wang et al.^48^, which characterized HLA-B*27:05-restricted TCRs from patients with ankylosing spondylitis and acute anterior uveitis. We used their experimentally measured T-cell activation data as ground truth, yielding 336 TCR-peptide pairs (21 TCRs, each tested against 16 candidate peptides; 65 activating and 271 non-activating pairs). All models were evaluated in a strict zero-shot setting.

#### Antigen-HLA binding affinity data

The antigen-HLA binding affinity data were collected from the IEDB^51,52^ database, including 168,711 antigen-HLA pairs. We implemented two splitting strategies: 1) Random splitting, where HLA molecules and antigens were randomly split into training, validation, and testing sets. 2) Novel-HLA splitting, where none of the HLA molecules in the test set appeared in the training set. We collected independent test data from weekly data^55^ between Oct. 2021 and Nov. 2024 with binding affinity measurements in the IEDB database. The duplicated data with our training data were removed, and the final independent test data includes 431 antigen-HLA pairs.

#### TCR cross-reactivity data

We collected cross-reactivity data, i.e., one TCRαβ is able to bind multiple antigens, from the curated TCR-antigen-HLA dataset. Finally, we obtained 20 groups of TCRαβs with diverse conservation ratios. To ensure the integrity of the cross-reactivity evaluation, when constructing the training set for this specific experiment, we rigorously removed related data that could lead to data leakage. An Antigen-HLA-TCR complex was defined as related and consequently excluded from the training set if it met one of the following criteria: 1) Antigen Sequence Similarity: The antigen peptide showed a sequence identity of > 50% with any training peptide. 2) TCR Sequence Similarity: The TCRα CDR and TCRβ CDR sequences shared a sequence identity of > 50% with any training TCR. This stringent exclusion guarantees that the model is tested on complexes with genuinely novel interaction surfaces and not on near-homologs present in the training data. To assess whether the low-conservation cross-reactive peptides retain HLA presentation capacity, we evaluated their predicted pMHC binding affinity using NetMHCpan 4.1. Of the 88 peptides in the low-conservation groups, 72 (81.8%) were predicted as HLA binders (percentile rank ≤ 10%; Supplementary Fig. S25). Additionally, the remaining 16 peptides have been independently confirmed as positive T-cell epitopes via IEDB T-cell assay records.

#### TCRαβ-antigen-HLA complex structure data

The crystal structures of the TCRαβ-antigen-HLA complex were derived from the STCRDab^56^ database, which provides highly curated annotations of TCR complexes. It offers standardized definitions for CDR loops and V/J gene usage, which streamlines data processing. To ensure high data quality for training the interaction prediction module, we applied the following strict filters to the retrieved complexes: 1) Completeness: Only structures containing all four components—TCRα, TCRβ, Antigen, and HLA—were retained. Partial structures (e.g., HLA-Antigen dimers) were excluded as they lack the necessary ground-truth quaternary interface contacts required for our model’s objective. 2) Verification: Given the known functionality issues of the STCRDab search engine, we manually verified the PDB IDs, confirming the presence of all required chains and performing a visual inspection of calculated contact maps for accuracy. 3) De-duplication: Duplicate structures were defined as those with identical sequences for the CDR3α, CDR3β, Antigen, and HLA allele. 4) Inclusion of Mutants: Structures differing by single amino acid substitutions (single mutants) were intentionally included. This was critical for enriching the model’s ability to learn fine-grained structural determinants of binding and specificity, enhancing the generalization capabilities required for cross-reactivity prediction. Finally, we constructed a structural dataset consisting of 164 crystal structures. For each retrieved quaternary structure, we computed the residue-level closest distances between any pair of residues across all four chains (TCRα, TCRβ, Antigen, and HLA) to serve as ground truth for the Interaction Conformation Prediction Module. This distance was calculated as the minimum Euclidean distance between all heavy (non-hydrogen) atoms of the two residues. The output of this calculation is a continuous distance matrix in Angströms (Å). For the purpose of visualization and interpretation of the contact maps in the results, a contact was defined as a residue pair with a closest heavy atom distance of <= 5.0 Å.

We also constructed a held-out independent test set. This test set was curated from TCR-antigen-HLA complex structures^57^. From this resource, we identified 39 available quaternary complex structures (containing complete TCRα, TCRβ, antigen, and HLA chains) that were deposited in the Protein Data Bank. To ensure complete independence, these structures were not used at any stage of pretraining or fine-tuning to provide an unbiased evaluation.

AlphaFold-augmented structural data. To expand the structural training data beyond the 164 experimentally determined crystal structures, we employed AlphaFold3 to predict TCR-antigen-HLA complex structures for all available tetrameric sequences in our curated dataset. A total of 2,652 structures were generated. To ensure structural reliability, we applied stringent quality filters based on the predicted Template Modeling (pTM) and interface predicted Template Modeling (ipTM) scores, retaining 1,037 high-confidence structures (Supplementary Fig. S21). The augmented variant (UpTCR+AF) was trained using the combined dataset of 164 crystal structures and 1,037 AlphaFold-predicted structures.

#### In-house breast cancer TCR data

The antigen-specific TCR data used in this study were derived from an in-house T-cell receptor screening project conducted by our research group, focusing on breast cancer patients. Samples were obtained from peripheral blood mononuclear cells (PBMCs) of clinically diagnosed Luminal and triple-negative breast cancer (TNBC) patients, all of whom provided informed consent and were enrolled under approved ethical protocols. A total of five breast cancer patients were recruited, including three with the Luminal subtype and two with the TNBC subtype. For each patient, 8 mL of peripheral blood was collected, and PBMCs were isolated using Ficoll-Paque PLUS density-gradient medium (Cytiva, 17144002) and washed with PBS (Gibco, C10010500BT). Following the Multiplex Identification of T-cell Receptor Antigen Specificity (MIRA) approach^58^, a unique address peptide matrix was constructed, in which 32 peptides (95% purity, Sangon Biotech, Shanghai, China) derived from highly expressed tumor-associated antigens in breast cancer (such as NY-ESO-1, WT1, Mam-A, MUC1, PIK3CA, etc.) were grouped into eight peptide pools. In this combinatorial encoding scheme, the eight pools were labeled A–H, and each of the 32 peptides was assigned a unique combination of four of the eight pools as its address (e.g., P1 → pools A, C, D, F; P2 → pools B, E, G, H), such that a T cell specific to a given peptide is expected to be enriched exclusively in the four pools comprising that peptide’s address. Each PBMC sample was divided into ten equal parts, with eight parts used for antigen stimulation with the respective peptide pools, and the remaining two serving as controls: one negative (no peptide stimulation) and one positive (stimulated with 1 μg/mL staphylococcal enterotoxin B, SEB; Toxin Technology, BT202). Cells were cultured in RPMI-1640 (ATCC modification; Gibco, A10491-01) supplemented with 10% FBS (Gibco, 10270106), MEM non-essential amino acids (Gibco, 11140050) and recombinant human IL-2, IL-4, IL-15 and GM-CSF (PeproTech, 200-02, 200-04, 200-15 and 300-03) for seven days, with peptide stimulation administered on days 3 and 7. This two-round stimulation regimen was adopted to expand the rare antigen-specific clones present at very low frequency in peripheral blood, while the four-day interval between stimulations allowed T-cell recovery and proliferation, minimizing the risk of activation-induced cell death (AICD). Consistent with this, cell viability was measured with trypan blue (Sigma-Aldrich, T8154); all stimulated groups remained highly viable on day 7 (81.2%–93.8%, comparable to the 88.8% of the unstimulated control) and exhibited net cell expansion (51.6%–242.1% increase in cell number; Supplementary Table S4), arguing against substantial AICD. On day 8, cells were stained with FITC anti-human CD3 (clone OKT3, BioLegend, 317306), APC anti-human CD8a (clone HIT8a, BioLegend, 300912), PE anti-human CD137 (clone 4B4-1, BD Biosciences, 555956), and propidium iodide (BioLegend, 421301), each at 1:50, and CD3⁺CD8⁺CD137⁺ activated T cells were sorted by flow cytometry. Because AICD-affected and anergic/dysfunctional cells are excluded by forward/side-scatter and viability gating and fail to upregulate CD137, this CD3⁺CD8⁺CD137⁺ gating strategy inherently enriches for functionally competent, antigen-responsive T cells. TCR libraries were prepared with a Human TRB VJ multiplex Kit (FAIR-SEQ, R0021I) and sequenced on a NovaSeq X Plus (Illumina) at Novogene (Beijing, China) to identify TCR clonotypes enriched in each peptide pool. By applying the unique address design in conjunction with the sequencing data, a direct correspondence between antigen identity and TCR sequence was established, enabling accurate identification of antigen-specific TCR clones.

The in-house TCR screening project yielded a robust dataset for analyzing antigen-specific T-cell responses in breast cancer. To ensure statistical rigor and minimize false positives, we established a high-confidence dataset by applying stringent thresholds for TCR clonotype enrichment relative to controls. Specifically, TCRs whose enrichment matched a single four-pool address (allowing at most one pool dropout, i.e., enrichment in three to four pools) were retained as high-confidence (strict-match) assignments, whereas TCRs enriched in five or more pools—potentially arising from cross-reactivity or technical noise—were resolved by Mann–Whitney U testing and clustering analysis to determine their most likely cognate peptide, thereby filtering out ambiguous signals. The subtype-specific response patterns observed (e.g., stronger Mam-A responses in the Luminal subtype and stronger survivin and KK-LC-1 responses in the TNBC subtype) further support the high specificity of this screening strategy and argue against substantial false-positive contamination. The final curated dataset comprises the following antigen-specific TCR-peptide interactions: 1) Luminal Subtype: The dataset includes 709 high-confidence positive TCR-antigen pairs, supported by 3,500 negative control pairs. 2) TNBC Subtype: The dataset includes 489 high-confidence positive TCR-antigen pairs, supported by 2,500 negative control pairs. This data composition provides a substantial foundation for training and evaluating TCR specificity prediction models in real-world applications.

### Model architecture

#### Representation of TCR

To fully utilize the data from TCRα and TCRβ, we performed independent modeling for TCRα and TCRβ. When both are available, they will be fused through the subsequent TCRαβFusionformer. Taking TCRα as an example, we derived a series of features to characterize it, including 1) full-length sequence features encoding the complete FV region, capturing both CDRs and FRs; 2) CDR3-specific motif embeddings, enabling targeted emphasis on this hypervariable loop known to play a critical role in antigen binding specificity; 3) structural and evolutionary features derived from advanced protein language models; and 4) Atchley factor and Blocks Substitution Matrix (BLOSUM) information. Unlike many prior studies that limited characterization to the CDR3 sequence alone—thereby potentially neglecting informative contextual residues—we incorporated the entire FV sequence and employed a one-hot encoding scheme to represent amino acid identities at each residue position, thus preserving sequence-level nuance and enabling downstream models to learn relevant spatial and sequential patterns. In addition, recognizing the pivotal role of the CDR3 region in determining antigen specificity, we specifically augmented the model’s input with dedicated CDR3 sequence embeddings. This dual representation strategy ensures that both global contextual and localized motif information are effectively captured. To further enhance the biological realism and predictive power of our model, we integrated structural and evolutionary information extracted from the recently developed protein language model ESM-ppi-TCR^33^, which represents an advancement over conventional models such as ESM-2^59^. ESM-ppi-TCR has been pretrained specifically on extensive TCR sequence datasets, thereby learning intricate residue-residue dependencies and evolutionary constraints unique to TCRs. This pretrained model facilitates the extraction of high-fidelity embeddings that encode both the three-dimensional structural propensities and evolutionary conservation patterns inherent in TCR sequences, providing rich, context-aware representations crucial for accurate binding prediction. For the TCRβ chain, an analogous feature-extraction and modeling framework was employed to maintain consistency and ensure a symmetrical representation of both chains.

#### Representation of antigen

To represent antigens, we built two types of features, including 1) sequence features and 2) evolutionary representations. The sequence feature is a one-hot representation to indicate the amino acid type of the residue. For evolutionary features, we utilized ESM-pep, a protein language model based on ESM-2^59^ further pretrained on peptide sequences, to generate the embedding for each residue.

#### Representation of HLA

For HLA modeling, we utilized pseudo-sequences representing residues of the HLA molecule, with a distance of 4.0 Å between any pair of atoms from the HLA and the bound peptide, taken from known antigen-HLA crystal structures. In addition to one-hot representation, we incorporated Atchley factor^36^ and Blocks Substitution Matrix (BLOSUM)^37^ information to enhance the representation of HLA.

#### Structures of TCRαformer, TCRβformer, TCRαβFusionformer

Supplementary Fig. S2 illustrates the detailed structure of TCRαformer (a), TCRβformer (b), TCRαβFusionformer (c). For the TCRα, TCRαformer employs two residual convolutional neural networks to extract features from the FR and CDR3 regions of the TCRα sequence, effectively capturing both the global characteristics of the TCRα and the local motifs of the CDR3 region. The extracted features are then combined to form a unified representation of TCRα sequences. In addition, we utilized a pretrained TCR large language model, which has been trained on large-scale TCR data, to extract evolutionary and structural features from the TCRα sequences. By incorporating these features, the representation of TCRα is further enhanced by the self-attention mechanism. Similarly, TCRβformer employs the same strategy to encode TCRβ. After obtaining the individual representations of TCRα and TCRβ, the TCRαβFusionformer first concatenates them to create a combined representation. Then, TCRαβFusionformer utilizes residual convolutional neural networks and self-attention mechanisms to further enhance the expressive power of the fused representation.

#### Structures of Antiformer, HLAformer, AntiHLAFusionformer

Supplementary Fig. S3 displays the detailed structure of Antiformer (a), HLAformer (b), AntiHLAFusionformer (c). For the antigen, Antiformer uses residual convolutional neural networks to capture its motif representation. Meanwhile, Antiformer leverages a pretrained peptide large language model, which has been trained on large-scale peptide data, to extract evolutionary features from the antigen sequences. The two representations are fused into a single representation, followed by a self-attention mechanism to improve its expressiveness. For the HLA molecule, HLAformer utilizes residual convolutional neural networks to extract crucial residue embedding from the pseudo sequence. Following the acquisition of the individual representations for the antigen and HLA, the AntiHLAFusionformer concatenates them to form a unified feature representation. Subsequently, it employs residual convolutional neural networks alongside self-attention mechanisms to further augment the expressive capacity of the fused representation.

#### SoftCLIP

To learn generalizable binding patterns and pretrain paired embeddings in stage 1 and stage 2, including TCRα-antigen, TCRβ-antigen, (TCRα + TCRβ)-antigen, TCRα-(antigen+HLA), and TCRβ-(antigen+HLA), we proposed a SoftCLIP algorithm. For example, for antigen and TCRβ, our goal aims to align both representations into a shared feature space. For a batch antigen and TCRβ data, they were first transformed into features ${P\_}1,{P\_}2,\,\ldots,{P\_N}$ and ${B\_}1,{B\_}2,\,\ldots,{B\_N}$ via Antiformer and TCRβformer, respectively. Then a cross-modal similarity matrix is computed with elements ${B\_i\; P\_i}$. Traditional CLIP^38^ algorithm often employs the Information Noise Contrastive Estimation (InfoNCE) loss to maximize the similarity of matching pairs and minimize that of non-matching pairs (Supplementary Fig. S4a):

1 $${L}_{{\mbox{CLIP}}}=-\log \left(\frac{\exp \left(\frac{{B}_{i}{P}_{i}}{\tau }\right)}{{\sum }_{j=1}^{N}\exp \left(\frac{{B}_{i}{P}_{j}}{\tau }\right)}\right)$$

where τ is the temperature parameter. However, we noted that such hard labels could lead to false-negative problems because negative samples are often randomly paired. To address this problem, we introduced the intra-modal similarity to soften the training labels (Supplementary Fig. S4b). Initially, SoftCLIP also calculates the cross-modal similarity matrix ${S\_cross}$ with elements ${B\_i\; P\_i}$ and defines a label matrix H where matching positions are 1 and others are 0. Subsequently, SoftCLIP calculates the intra-modal similarities, including the TCRβ similarity matrix ${S\_B}$ with elements ${B\_i\; B\_j}$ and antigen similarity matrix ${S\_P}$ with elements ${P\_i\; P\_j}$. The softened labels are created by combining hard labels with intra-modal similarities: ${P\_B}=(1-\beta )H+\beta {S\_B}$ and ${P\_P}=(1-\beta )H+\beta {S\_P}$, where β is a balanced coefficient that controls the contribution of intra-modal similarities. Then SoftCLIP uses KL-Divergence to measure the differences between the predicted cross-modal similarities and the softened labels:

2 $${L}_{{sy}{m}_{{KL}}}=\frac{1}{2}\left[{KL}\left(\log {softmax}\left({S}_{{cross}}\right) | {softmax}\left({P}_{B}\right)\right)+{KL}\left(\log {softmax}\left({S}_{{cross}}\right) | {softmax}\left({P}_{P}\right)\right)\right]$$

The overall SoftCLIP loss function combines the cross-modal alignment loss with the intra-modal guidance loss, defined as follows:

3 $${L}_{{SoftCLIP}}={L}_{{CLIP}}+\lambda \,{L}_{{sy}{m}_{{KL}}}+\mu \,{L}_{r{e}_{{sy}{m}_{{KL}}}}$$

where ${L\_re\_sym\_KL}$ is the reversed KL divergence loss (ignoring diagonal losses), λ and μ are weighting parameters. This SoftCLIP strategy is applied to multiple interaction pairs, including TCRα-antigen, TCRβ-antigen, (TCRα + TCRβ)-antigen, TCRα-(antigen+HLA), and TCRβ-(antigen+HLA).

#### SoftTri

For paired embeddings of antigen-HLA complexes, we developed a specialized SoftTri algorithm to pretrain this specific interaction pair. The decision to employ a distinct approach from SoftCLIP was driven by two key considerations specific to antigen-HLA binding characteristics.

First, antigen-HLA interactions benefit from extensive quantitative binding affinity data unavailable for other component pairs. Second, while binary classification thresholds (e.g. 500 nM) are commonly used to define binding events, the biological reality is more nuanced - interactions below this threshold may still trigger T-cell responses. These considerations necessitated an affinity-aware learning strategy that could capture continuous binding relationships rather than discrete categories.

Triplets are formed, consisting of an anchor (HLA), a binder (positive antigen), and a decoy (negative antigen). The traditional triplet contrastive learning^60^ is to minimize the distance between the anchor and binder while maximizing the distance between the anchor and decoy (Supplementary Fig. S5a). In contrast, SoftTri introduces affinity-aware weighting mechanisms that modulate the learning objective based on quantitative binding measurements (Supplementary Fig. S5b). This innovation allows the model to learn a more biologically realistic embedding space that reflects the continuous nature of molecular interactions. Formally, the weighting terms ${w\_pos}$ and ${w\_neg}$ are derived directly from quantitative binding affinity measurements (IC_50_ values) through the following transformations:

4 $${w}_{{pos}}=\exp \left(-\frac{I{C}_{50,{pos}}}{k}\right)$$

5 $${w}_{{neg}}=1-\exp \left(-\frac{I{C}_{50,{neg}}}{k}\right)$$

where k is a scaling factor that normalizes the affinity values (set to 500 nM in our experiments). Then the complete SoftTri loss function with these elements is expressed as:

6 $${L}_{{SoftTri}}=\frac{1}{N}{\sum}_{i=1}^{N}\left[{w}_{{pos},i}\cdot d\left({a}_{i},{b}_{i}\right)+{w}_{{neg},i}\cdot \max \left(m-d\left({a}_{i},{d}_{i}\right),0\right)\right]$$

where N is the number of triplets, ${w}_{{pos},i}$ and ${w}_{{neg},i}$ are weights for the positive and negative pairs, $d\left({a}_{i},{b}_{i}\right)$ is the distance between anchor and binder, $d\left({a}_{i},{d}_{i}\right)$ is the distance between anchor and decoy, m is the margin parameter.

#### TCR-antigen-HLA binding specificity prediction module

The TCR-antigen-HLA-finetuner module is designed for predicting TCR-antigen-HLA interactions. It takes the representations of TCRαβ and antigen-HLA generated by TCRαβFusionformer and AntiHLAFusionformer, along with their masks, as input. These inputs are concatenated and fed into convolutional layers to extract local features, followed by batch normalization. The acquired features are then fed into a self-attention layer to capture global dependencies, followed by multiple fully connected layers to output the binding probability.

#### Modality-missing prediction module

The representations of TCRα, TCRβ, TCRαβ, antigen, hla, antigen-HLA can be acquired through TCRαformer, TCRβformer, TCRαβFusionformer, Antiformer, HLAformer, AntiHLAFusionformer, respectively. Therefore, when certain modalities are missing, we can extract the representations from the available modalities and concatenate them. These concatenated features are then processed through residual convolutional layers, batch normalization, self-attention mechanisms, and finally fed into fully connected layers for the final prediction.

#### Antigen-HLA binding affinity prediction module

For the antigen-HLA binding affinity prediction task, we utilized the combined antigen-HLA representation produced by AntiHLAFusionformer. The combined representation is fed into multiple fully connected layers to predict the binding affinity between antigen-HLA.

#### Interaction conformation prediction module

The interaction conformation prediction module is designed to predict the residue-level spatial distances between any pair of components, including TCRα-antigen, TCRβ-antigen, antigen-HLA, TCRα-TCRβ, TCRα-HLA, and TCRβ-HLA (Supplementary Fig. S18). For example, for antigen-HLA, the interaction conformation prediction module takes representations of antigen and HLA from Antiformer and HLAformer as input. Each representation is first fed into convolutional layers and layer normalization to enhance its expressive ability. Then the two representations are integrated by outer product and fed into a convolutional layer, followed by a ReLU activation function to predict the pairwise distance matrices between antigens and HLA molecules. For the other five interaction pairs, UpTCR employs similar structures to obtain the predicted distance map.

### Model training

For the training of UpTCR, we initially employed a two-stage pretraining approach, beginning with training on dimers before transitioning to trimers. Subsequently, we finetuned the model for various downstream tasks, including TCR-antigen-HLA binding specificity prediction, antigen-HLA affinity prediction, and binding conformation prediction. This structured training process allows for the effective adaptation of the model to the complexities of T cell receptor interactions.

#### Two-stage pretraining

In the first stage, we pre-trained UpTCR using dimer data from TCRα-antigen, TCRβ-antigen, and antigen-HLA interactions. This phase primarily focused on training four monomer encoders: TCRαformer, TCRβformer, Antiformer, and HLAformer. For the TCRα-antigen and TCRβ-antigen datasets, we employed the SoftCLIP algorithm, while the antigen-HLA interactions were processed using the SoftTri algorithm. These three datasets were integrated and trained simultaneously. The training was conducted with a batch size of 256, a learning rate of 5e-4, and a weight decay of 1e-2. Thus, the four pretrained monomer encoders were obtained.

In the second stage, we pretrained UpTCR using trimer data from TCRα-antigen-HLA, TCRβ-antigen-HLA, and TCRα-TCRβ-antigen interactions. This phase primarily focused on training two fusion encoders: TCRαβFusionformer and AntiHLAFusionformer. The single representations obtained from the monomer encoders were input into the fusion module to generate integrated representations. All three datasets were simultaneously trained using the SoftCLIP algorithm. The training was conducted with a batch size of 256, a learning rate of 5e-4, and a weight decay of 1e-2. Thus, the two pretrained fusion encoders were obtained.

#### Task-specific finetuning

After two-stage pretraining, we obtained four pretrained monomer encoders and two pretrained fusion encoders, which were utilized for downstream tasks. 1) As for the TCR-antigen-HLA binding specificity finetuning, the TCRαβ and antigen-HLA representations can be extracted from the pretrained encoders and were then fed into the TCR-antigen-HLA binding specificity prediction module to obtain the predicted logits. The binary cross-entropy between predicted logits and labels was calculated as the objective function. The model is optimized using the Adam optimizer for 100 epochs with a learning rate of 5e-4, and the batch size is set as 256. 2) As for the modality-missing prediction, we fed the available data into the corresponding pretrained encoders to obtain their representations, followed by the modality-missing prediction module to obtain the predicted logits. The model is also optimized using the Adam optimizer for 100 epochs with a learning rate of 5e-4 and the batch size is set as 256. 3) As for the antigen-HLA binding affinity prediction, we acquired the antigen-HLA unified representation through the pretrained Antiformer, HLAformer, and AntiHLAFusionformer. Then the unified representation was fed into the antigen-HLA binding affinity prediction module to obtain the predicted affinity. The mean square error (MSE) between the predicted affinity and the true labels is computed as the objective function. The model is optimized using the Adam optimizer for 100 epochs with a learning rate of 5e-4 and the batch size is set as 256. 4) As for the interaction conformation prediction, we fed each paired representation into the interaction conformation prediction module to obtain residue-level distances. A modified MSE loss divided by the distance between residues was employed to reduce the impact on predictions from distant residue pairs. All six paired distances were simultaneously optimized, with the Adam optimizer for 100 epochs with a learning rate of 5e-4 and the batch size is set as 1.

#### Benchmarking and competitor evaluation

To ensure a fair and rigorous comparison, all machine learning-based competitor models were implemented and retrained using the exact same curated and filtered training dataset utilized for UpTCR. We strictly adhered to the original input feature requirements of each competitor model. We acknowledge that UpTCR is a foundation model that benefits from large-scale, multi-stage pretraining, a capability not present in the specialized competitor models. This difference in training scheme further highlights the architectural advantage of our proposed approach.

### In vitro T cell response experiments

#### Cell lines and reagents

The Jurkat, Clone E6-1 cell line was obtained from ATCC (TIB-152). CD8 was overexpressed in the Jurkat cell line. Jurkat and CD8^+^ Jurkat cells were cultured in RPMI-1640 (ATCC modification; Gibco, A10491-01) supplemented with 10% FBS (Gibco, 10270106) and 100 μg/ml of penicillin and streptomycin. The HLA-A*02:01-positive A375 cell line was obtained from ATCC (CRL-1619) and cultured in DMEM (Gibco, C11885500BT) supplemented with 10% FBS and 100 μg/ml of penicillin and streptomycin. The 293FT cell line was obtained from Invitrogen (Thermo Fisher Scientific, R70007) and cultured in DMEM (Gibco, C11885500BT) supplemented with 10% FBS and 100 μg/ml of penicillin and streptomycin. No additional STR profiling or mycoplasma testing was performed in this study.

#### Peptide synthesis

Lyophilized peptides for neoantigens were manufactured at ≥95% purity by Sangon Biotech (Shanghai, China). The peptides were verified by high-performance liquid chromatography and stored at –80 °C for testing the T cell reactivity.

#### Expression constructs and lentiviral transduction

The lentiviral vector was obtained from VectorBuilder. The TCR α and β chains were linked by a 2 A self-cleaving peptide. The TCR α chain, 2 A linker, and β chain fusion gene fragment was inserted into the lentiviral vector. 1.5×10^6^ 293FT cells were plated in 6 cm plates in 5 ml DMEM containing 10% FBS without antibiotics at sufficient density to provide 60% to 70% confluence after 24 h. Cells were co-transfected with 2 µg of lentiviral vector DNA and 1.5 µg of packaging plasmid (psPAX2) and 1 µg envelope plasmid (pMD2.G) using PEI (Polysciences, 23966). Transfection medium was replaced with 5 ml complete medium after 6 h incubation, and lentiviral supernatants were collected after 60 h. Jurkat cells were resuspended in lentiviral supernatant at 24-well flat-bottom tissue for 24 h. After 24 h, the transduced cells were resuspended at 5×10^5^/ml in culture medium and TCR+ transduced cells were sorted on the TurboRFP/PE channel (the parental CD8+ Jurkat line already uniformly expressed CD8). Post-sort purity was greater than 90% for both CD8+ and TCR+ cells.

#### Functional analysis of TCR^+^ Jurkat cells

TCR^+^ CD8^+^ Jurkat cells (1×105 per well) were co-cultured for 24 h at 37 °C under 5% CO₂ in 24-well plates with HLA-A*02:01 A375 cells (1×105 per well; effector-to-target ratio 1:1) that had been pre-pulsed overnight with 1 μg ml–1 of different antigen variants or unrelated peptides. TCR^+^ CD8^+^ Jurkat cells or supernatants were then harvested and evaluated for detection. PMA/ionomycin (BioLegend, cat. no. 423304) was used as a receptor-independent positive control (Supplementary Fig. S29). DMSO (Coolaber) and an unrelated peptide were used as the negative controls.

#### Flow cytometric analysis

To assess the activation of TCR^+^ CD8^+^ Jurkat cells following antigen peptide stimulation, cells were harvested and stained for 30 min at room temperature with a Zombie Aqua Fixable Viability kit (BioLegend, cat. no. 423102), anti-CD8 (clone RPA-T8, PE/Dazzle 594, BioLegend, cat. no. 301058), anti-CD69 (clone FN50, Brilliant Violet 421, BioLegend, cat. no. 310930). The dilution of all the antibodies was 1:100. After incubation at room temperature for 30 min, cells were resuspended in the staining buffer and analyzed using CytoFLEX S flow cytometer with CytExpert software (version not recorded).

#### Cytokine release detection

Cytokine release in supernatants was detected by a human IL-2 ELISA kit (Multi Sciences, EK102) according to the manufacturer’s instructions.

Newly generated plasmids and engineered cell lines are available from the corresponding authors upon request.

### Statistical analyses

For computational benchmarks (Figs. 2a–h and 3a–f, and the corresponding Supplementary bar charts), UpTCR was compared with each baseline or ablation using a two-sided unpaired Student’s *t*-test across *n* = 5 independent training-and-evaluation runs or cross-validation folds. *P* values were not adjusted for multiple comparisons. Differences in ROC AUC (Fig. 2i–l) were assessed with a two-sided DeLong test. Two-group comparisons of predicted binding probabilities (Fig. 5a, f) and residue-contact AUCs (Fig. 6a,b) used a two-sided Mann–Whitney *U* test without adjustment. For the prospective cell-based assays (*n* = 3 independent experimental batches), overall differences among peptide conditions were assessed in GraphPad Prism 10. Interleukin-2 secretion (Fig. 8b) was analyzed with a Kruskal–Wallis test followed by uncorrected two-sided Dunn’s tests versus DMSO. CD69⁺ frequency (Fig. 8c) was analyzed with ordinary one-way ANOVA followed by Tukey’s post hoc tests versus DMSO. A threshold of *P* < 0.05 was considered statistically significant. Exact *P* values are provided in the Source Data. Performance metrics (AUC, AUPR, MSE, Pearson correlation, and Spearman correlation) were calculated in Python. Multiple sequence alignment was performed using the MEGA11 software^61^. Sequence motifs were visualized with WebLogo 3 software^62^. Structures were rendered in PyMOL^63^. Computational model training and evaluation were implemented in Python (version not recorded) with PyTorch 2.0.1, NumPy 1.24.3, SciPy 1.10.1, pandas 2.0.2, Transformers 4.30.2 and scikit-learn (version not recorded), consistent with the public code environment.

### Reporting summary

Further information on research design is available in the Nature Portfolio Reporting Summary linked to this article.

## Supplementary information

Supplementary Information

Reporting Summary

Transparent Peer Review file

## Source data

Source Data

## Data Availability

The processed TCR–antigen–HLA datasets generated in this study have been deposited in Zenodo^64^ under accession code 15128399 and at Hugging Face [https://huggingface.co/datasets/DDDead/Uptcr_data]. The in-house breast cancer transfer evaluation uses processed TCR and peptide amino-acid sequences from these deposits (the analysis-ready protein sequences used in this study). Public source datasets are available from VDJdb^45^ [https://vdjdb.cdr3.net/], PIRD^49^ [https://db.cngb.org/pird/], McPAS-TCR^50^ [https://friedmanlab.weizmann.ac.il/McPAS-TCR/], IEDB^51,52^ [https://www.iedb.org/], 10x Genomics^53^ [https://www.10xgenomics.com/datasets], STCRDab^56^ [https://opig.stats.ox.ac.uk/webapps/stcrdab-stcrpred/Browser] and the IEDB MHC-I weekly benchmark^55^ [http://tools.iedb.org/auto_bench/mhci/weekly/]. All other data are available in the article and its Supplementary files or from the corresponding author upon request. Source data are provided with this paper.

## Code availability

The source code used in this study is available at GitHub [https://github.com/tylerlv/UpTCR] under the MIT License and has been archived in Zenodo^65^ under accession code 21979241. Model weights have been deposited in Zenodo^66^ under accession code 20520000.
