# Supplementary material for: UpTCR: a unified progressive knowledge transfer foundation model for robust T-cell receptor-antigen binding recognition
Source: Nat Commun. 2026 Sep 24;17:11086. doi: 10.1038/s41467-026-78075-x (PMC13696701; doi:10.1038/s41467-026-78075-x)
Supplement: Supplementary file 1 [file 41467_2026_78075_MOESM1_ESM.pdf]

UpTCR: a unified progressive knowledge transfer foundation model for robust T-cell receptor-antigen binding recognition

Supplementary Note

1. Pre-training data

Data source

The pretraining data for UpTCR was systematically curated from multiple publicly available databases to ensure comprehensive coverage of TCR-antigen-HLA interactions. Primary public sources included VDJdb, McPAS-TCR, PIRD, IEDB, and 10X Genomics datasets, which were downloaded until Nov. 2024. We implemented a standardized preprocessing pipeline that included: (1) removal of sequences with ambiguous residues or incomplete CDR3 regions; (2) normalization of HLA allele nomenclature using the IPD-IMGT/HLA database standards; (3) quality control based on source-specific confidence metrics (*e.g.*, VDJdb confidence scores ≥ 1). Following preprocessing, the curated data were categorized according to their modality completeness to facilitate the progressive pretraining of UpTCR.

De-redundancy

To avoid data leakage and guarantee an unbiased evaluation of downstream tasks, we implemented a rigorous de-redundancy procedure on the incomplete dataset used during pretraining. Sequence similarity between the entire pretraining corpus and the final test datasets was assessed using the MMseqs2 tool. Any pretraining instance was removed if it satisfied at least one of the following similarity thresholds in relation to the downstream test data: (1) antigen sequence similarity exceeding 30% sequence identity; (2) TCRα CDR sequence similarity exceeding 30% sequence identity; (3) TCRβ CDR sequence similarity exceeding 30% sequence identity.

Pretraining data in stage 1

Stage-1 pretraining utilized pairwise interaction data to establish foundational representations of individual components. The dataset comprised three distinct dimeric categories:

- TCRα-Antigen Interactions: We collected 291 validated TCRα-antigen pairs after the de-redundancy procedure for SoftCLIP pretraining.
- TCRβ-Antigen Interactions: a total of 39,768 validated TCRβ-antigen pairs were compiled after the de-redundancy process for SoftCLIP pretraining.

- Antigen-HLA Interactions: A total of 26,083 positive pairs with quantitative binding affinity measurements were obtained from IEDB, covering major HLA class I alleles. Affinity values (IC_{50}) were log-transformed and normalized to a consistent scale for model training. In addition, we collected 85,329 negative pairs with binding affinity measurements as decoy for SoftTri pretraining.

The data were split into training/validation sets (9:1 ratio) while ensuring no overlapping antigens or TCRs between splits. This stage primarily focused on training the four monomer encoders (TCRaformer, TCRbformer, Antiformer, HLAformer) using specialized loss functions: SoftCLIP for TCR-antigen alignment and SoftTri for affinity-informed antigen-HLA pretraining.

Pretraining data in stage 2

Building upon Stage 1, stage-2 incorporated higher-order interactions to train the fusion encoders:

- TCRα-TCRβ-Antigen Trimers: 326 paired αβ-TCR complexes with known antigen specificity.
- TCRα-Antigen-HLA Trimers: 12,124 paired TCRα-antigen-HLA complexes.
- TCRβ-Antigen-HLA Trimers: 43,382 paired TCRβ-antigen-HLA complexes.

All trimeric data underwent stringent filtering to ensure chain pairing accuracy and HLA restriction consistency. The training leveraged representations from the Stage 1-pretrained encoders, with the TCRαβFusionformer and AntiHLAFusionformer learning to integrate features from respective component pairs. SoftCLIP objectives were used to align fused representations with remaining components.

2. TCR specificity prediction data

Data source and negative samples

The finetuning dataset for TCR specificity prediction included complete TCRα-TCRβ-antigen-HLA binding data (36061). For each antigen-HLA complex in our dataset, we employed a well-established random pairing strategy to generate negative samples. Specifically, TCR sequences known to bind to other antigen-HLA complexes were randomly paired with non-cognate antigen-HLA combinations to create presumed negative interactions. This approach resulted in a final dataset with a positive-to-negative sample ratio of approximately 1:5, ensuring adequate representation of both binding and non-binding events.

To comprehensively evaluate UpTCR's generalization capabilities, we defined distinct evaluation settings based on the available data density for each unique antigen-HLA pair, including majority, few-shot, zero-shot, and one positive zero-shot settings.

Majority learning setting

This setting assesses the model's performance in a standard, data-rich environment where high confidence can be built. The Majority Set includes Antigen-HLA complexes where the total number of associated TCRs (including both positive and generated negative samples) exceeds 100 entries. In this setting, all Antigen-HLA types represented in the test set were also present in the training set, and the model was trained on a substantial volume of TCR data for each complex. This configuration allows the model to encounter substantial TCR diversity for each antigen-HLA complex during training, simulating conditions where comprehensive binding data is available for most targets. The majority setting primarily assesses the model's ability to achieve peak performance when abundant training data is accessible.

Few-shot learning setting

This setting tests the model's ability to learn specificity from limited examples, a scenario common for rare epitopes. The Few-Shot setting is defined by Antigen-HLA complexes where the total number of associated TCRs (positive and negative samples) is less than 50 entries. Similar to the Majority Set, all Antigen-HLA types in the test set were observed during training, but the model encountered only a minimal number of TCR data points for each complex. This challenging evaluation tests the model's capability to learn effectively from limited supervision, mimicking practical situations where only scarce binding data is available for certain antigen-HLA complexes. The few-shot setting is particularly relevant for predicting TCR specificity against novel antigens or rare HLA alleles where experimental data is inherently limited.

Zero-shot learning setting

The zero-shot scenario was designed to evaluate the model's generalization to completely unseen antigen-HLA complexes. To prevent data leakage and ensure rigorous evaluation, we implemented strict sequence similarity filtering between the training and test sets. We evaluated two zero-shot settings of increasing stringency: (1) 50% sequence identity filtering, in which any training sample sharing > 50% identity with test sequences was removed; and (2) 30% sequence identity filtering, which applies an even more conservative threshold to further minimize potential information leakage through distant homologs. Both settings ensure that the model is tested on complexes with novel interaction surfaces, assessing its capacity to generalize learned binding principles to unseen targets without any direct training examples.

3. Evaluation metrics

Area Under the Receiver Operating Characteristic Curve (AUC-ROC)

The AUC-ROC is a metric that evaluates the overall discriminative power of the model. The Receiver Operating Characteristic (ROC) curve plots the True Positive Rate (Sensitivity) against the False Positive Rate (1 - Specificity) across all possible classification thresholds. A high AUC score indicates the model's ability to effectively rank positive binding events higher than negative non-binding events.

Area Under the Precision-Recall Curve (AUPR)

The Area Under the Precision-Recall curve (AUPR) is crucial for evaluating performance on highly imbalanced datasets, which is characteristic of molecular binding data. The PR curve plots Precision (Positive Predictive Value) against Recall (Sensitivity). AUPR is highly sensitive to the presence of false positives and provides a more realistic measure of performance in practical scenarios where experimental validation focuses on minimizing costly false positive predictions.

AUC0.1

The partial AUC at a false positive rate of 0.1 (AUC0.1) quantifies the model's discriminative performance within the high-specificity regime (FPR ≤ 0.1). Unlike the full AUC-ROC, which evaluates performance across all operating thresholds, AUC0.1 focuses exclusively on the region where the model's most confident predictions reside. This metric is particularly relevant for practical applications where only a small number of top-ranked candidates can be selected for costly downstream experimental validation, and minimizing false positives among the highest-confidence predictions is paramount.

Mean Squared Error (MSE)

The Mean Squared Error (MSE) is the fundamental loss function and evaluation metric used for our regression tasks, particularly in training the Interaction Conformation Prediction Module. MSE measures the average squared difference between the model's predicted continuous value ($\hat{y}$) and the true ground-truth value (y), providing a direct measure of the prediction accuracy in the original units (e.g., Angströms for distance prediction):

$$\text{MSE} = \frac{1}{n} \sum_{i=1}^{n} (y_i - \hat{y}_i)^2$$

Pearson Correlation Coefficient

The Pearson Correlation Coefficient measures the linear relationship between the predicted quantitative values and the ground-truth values. It assesses how accurately the model predicts the magnitude of the output, assuming a linear fit. This is particularly relevant when evaluating the fidelity of predicted contact distances or known quantitative binding affinity values:

$$\rho = \frac{\sum_{i=1}^{n} (x_i - \bar{x})(y_i - \bar{y})}{\sqrt{\sum_{i=1}^{n} (x_i - \bar{x})^2} \sqrt{\sum_{i=1}^{n} (y_i - \bar{y})^2}}$$

Spearman's Rank Correlation Coefficient

Spearman's Rank Correlation Coefficient measures the monotonic relationship between the predicted values and the true values. Unlike Pearson, it assesses the accuracy of the relative ranking (ordering) of samples. This metric is essential for structural and affinity prediction tasks because correctly prioritizing the strongest binders or the shortest distances is often more critical than achieving precise numerical accuracy:

$$\rho_s = 1 - \frac{6 \sum d_i^2}{n(n^2 - 1)}$$

where d_i is the difference between the ranks of the predicted and true values for sample i.

4. Baselines for TCR-specificity prediction

To ensure a rigorous and equitable comparison, all machine learning-based competitor models were re-implemented and retrained using the identical, curated fine-tuning dataset and the exact same predefined splits utilized for UpTCR. This standardized approach was maintained across all evaluation scenarios—the majority set, few-shot setting, and zero-shot setting—thus isolating performance differences to the architectural advantages and knowledge transfer capabilities of the UpTCR foundation model, rather than variations in training data volume or quality.

DLpTCR (Deep Learning for peptide-TCR recognition) is an ensemble deep learning framework that integrates both sequence-based and physicochemical features of peptides and TCRs. It employs a one-dimensional convolutional neural network (1D-CNN) to extract local motif features from the amino acid sequences of peptides and TCR CDR3β regions. These features are then fused and passed to a fully connected network for binding prediction.

NetTCR2.0 represents a significant advancement over its predecessor by incorporating paired TCR alpha and beta chain sequences. This method uses a shallow CNN architecture to process the amino acid sequences of the peptide, the TCRα CDR3, and the TCRβ CDR3. The model learns informative sequence motifs directly from the data.

ERGO (AE & LSTM) a pioneering framework that uses a fixed-length feature vector to represent TCRs, enabling the prediction of antigen specificity. ERGO-AE uses an autoencoder (AE) trained on a large corpus of TCR sequences to learn a compressed, meaningful representation (embedding) of any TCR sequence. This embedding is then used as input to a classifier to predict binding. ERGO-LSTM replaces the autoencoder with a Long Short-Term Memory (LSTM) network to learn the TCR representation, potentially capturing more complex, long-range dependencies within the TCR sequence.

pMTnet is a notable approach that attempts to integrate antigen-HLA binding information with TCR recognition. The model first predicts the probability of antigen presentation by a specific HLA allele using a separate module. This information is then combined with TCR sequence data (CDR3β) in a neural network to predict TCR binding.

PanPep introduces a meta-learning framework designed to improve generalization to unseen antigens, a significant challenge in the field. It employs a Model-Agnostic Meta-Learning (MAML) strategy. The model is trained on a variety of antigens such that it can quickly adapt to predict TCR binding for a new, previously unseen antigen with only a few examples (few-shot learning).

EPACT (Epitope-anchored contrastive transfer learning) is a recently developed method that leverages transfer learning and contrastive learning for paired TCRαβ specificity prediction. It pre-trains a model using a large, unlabeled corpus of TCR sequences to learn generalizable TCR representations. It then fine-tunes the model on labeled TCR-antigen pairs using an epitope-anchored contrastive loss, which pulls TCRs recognizing the same antigen closer in the embedding space while pushing others apart.

5. Baselines for antigen-HLA binding affinity

To ensure a rigorous and equitable comparison of prediction fidelity, we implemented a standardized evaluation strategy across all quantitative binding affinity benchmarks. All baseline models for which source code was available (DeepAttentionPan, DBT, TransPHLA, ACME, DeepMHCI) were re-implemented and retrained using the identical quantitative binding data utilized for training UpTCR's Antigen-HLA module. This ensures that observed performance differences are due to architectural advantages rather than data heterogeneity. This comparison was conducted across distinct settings: Random-Split Validation and Novel-HLA Split Validation. As the NetMHCpan series method does not provide open-source code to support customized retraining or integration into our specific data pipelines, we were unable to retrain it. Therefore, we evaluated different versions of NetMHCpan on a separate, independent held-out test set derived from our curated corpus to ensure the comparison against the widely accepted industry standard is fair, even if not fully retrained.

DeepAttentionPan integrates attention mechanisms within a pan-specific deep learning framework. This method combines the pan-specific concept of NetMHCpan with a deep neural network featuring dedicated attention modules. This allows it to weigh the importance of different amino acids in the peptide and the HLA binding environment dynamically.

DBT (Differential Boundary Tree) adopts a distinct, non-neural network approach based on instance-based learning. It uses a tree structure where each node represents a training sample (peptide-HLA complex). Prediction for a new sample is made by traversing the tree along a path determined by similarity metrics, effectively comparing the new instance to the most similar known examples.

TransPHLA is a transformer-based model that represents a shift towards modern natural language processing (NLP) architectures in immunoinformatics. It models peptides and HLA pseudo-sequences as "sentences" of amino acid "words." Using the transformer architecture, which relies on self-attention, it captures long-range dependencies and contextual relationships within and between the peptide and HLAs.

ACME incorporates an attention mechanism into a convolutional framework. It combines CNNs for local feature extraction with an attention mechanism to weight the importance of different positions within the peptide and MHC pseudo-sequence. This allows the model to focus on critical residues governing binding.

DeepMHCI is a deep learning model that utilizes convolutional neural networks to predict MHC-I binding peptides. It employs a convolutional neural network (CNN) architecture to automatically learn informative

sequence motifs from the amino acid sequences of peptides and MHC alleles. The model is designed to capture both local residue patterns and global sequence features relevant for binding.

The **NetMHCpan** series is widely regarded as the gold-standard and most cited tool for predicting peptide-HLA-I binding affinity. This method employs artificial neural networks (ANNs) trained on a massive corpus of quantitative MHC-peptide binding data. Its key innovation is the "pan-specific" approach, which allows it to make predictions for HLA alleles not seen during training by leveraging sequence similarity between different alleles. It uses pseudo-sequences representing the HLA peptide-binding groove.

Supplementary Figures

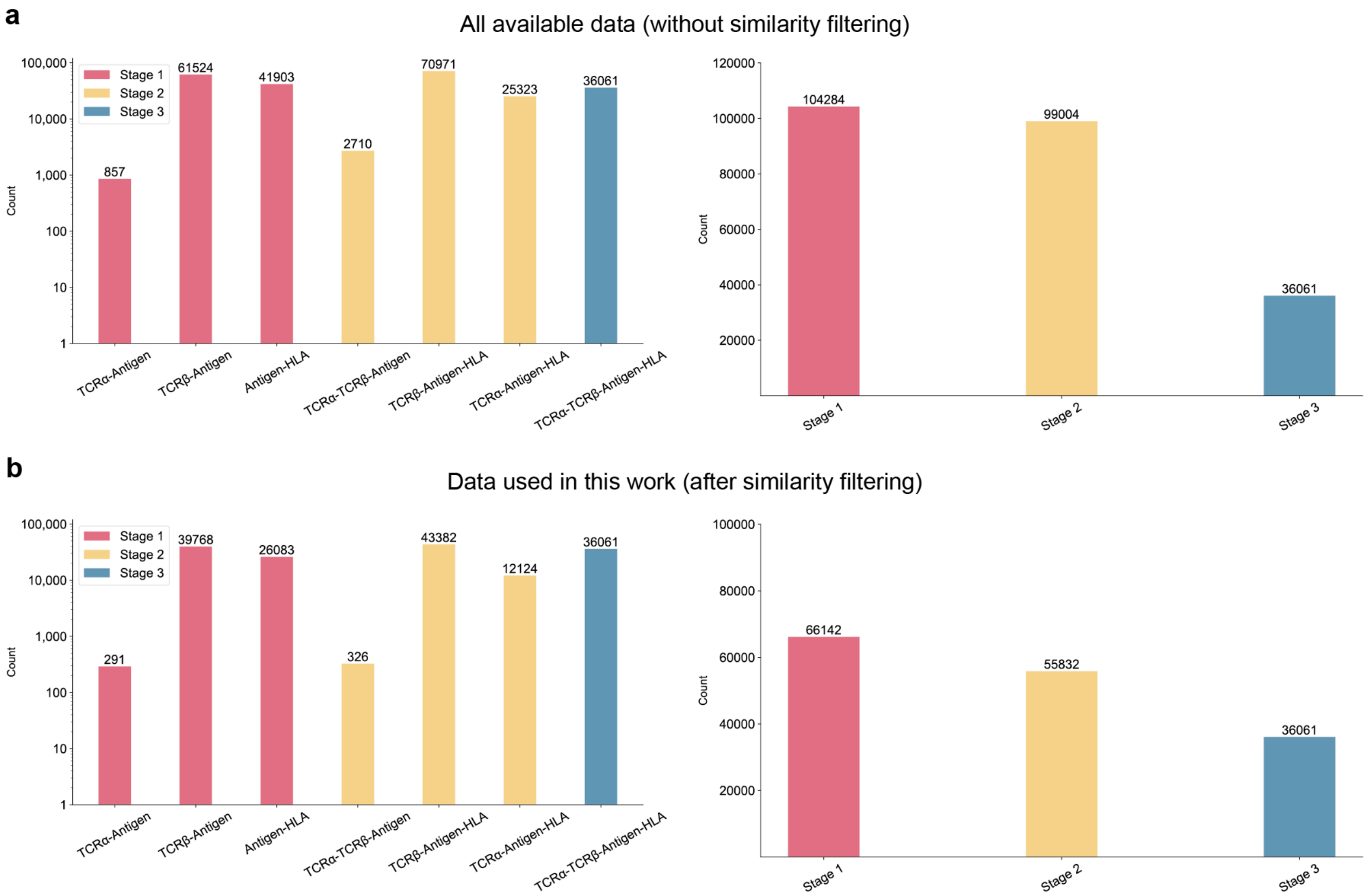

Figure S1. Data composition and modality availability before and after similarity filtering. a, Bar plot showing the total available data for each interaction type across three pretraining stages before applying similarity-based deduplication. Stage 1 includes dimeric interactions: TCRα–Antigen, TCRβ–Antigen, and Antigen–HLA. Stage 2 includes trimeric interactions: TCRα–TCRβ–Antigen, TCRα–Antigen–HLA, and TCRβ–Antigen–HLA. Stage 3 includes complete TCRα–TCRβ–Antigen–HLA tetrameric data. Heights of stacked bars indicate total sample counts per modality. **b,** Corresponding data counts after applying sequence-similarity filtering to remove highly homologous samples. This stringent filtering ensures minimal data leakage between pretraining and finetuning sets. *n* is the count displayed for that bar.

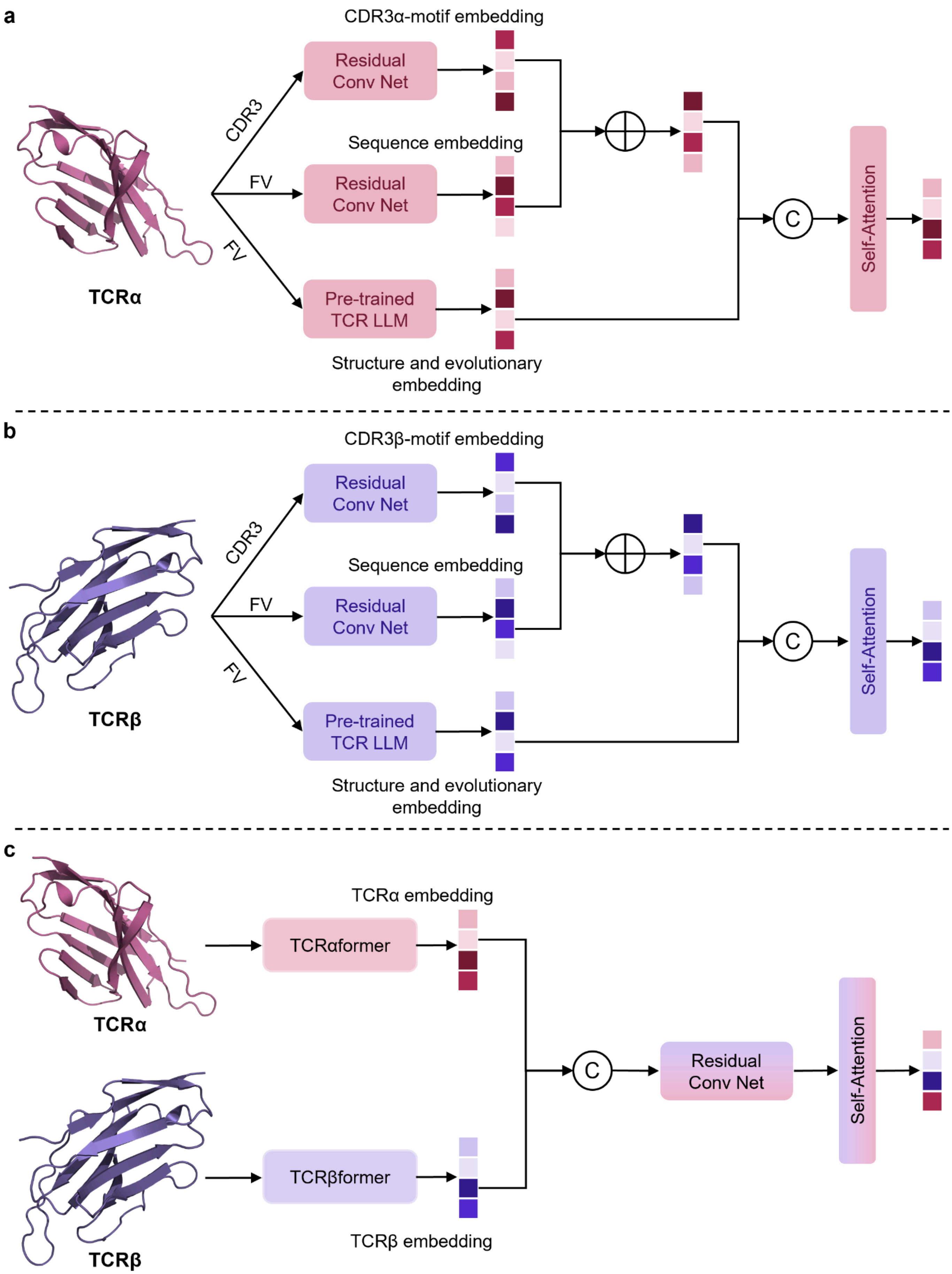

Figure S2. Detailed architectures of the TCRαformer, TCRβformer, and TCRαβFusionformer modules. a, Schematic of the TCRαformer module. The input consists of the full-length TCRα sequence.

The full-length sequence is processed through a residual convolutional neural network. Simultaneously, the CDR3 region is encoded via a weight-sharing residual convolutional neural network block to extract critical local motifs. These two feature streams are integrated (denoted by the circled plus sign ⊕), and the combined representation is further enhanced by incorporating evolutionary and structural embeddings from a pretrained TCR-specific large language model. The final output is fed into a multi-head self-attention layer to capture global contextual features, providing the comprehensive representation of the TCRα chain. **b,** Schematic of the TCRβformer module. This module employs an architecture identical to the TCRαformer, but is applied to the TCRβ chain sequence to generate its dedicated representation. **c,** Schematic of the TCRαβFusionformer module. The individual representations of TCRα and TCRβ from their respective formers are first concatenated. The fused feature vector is then processed by a residual convolutional neural network block and a multi-head self-attention layer to learn interactive features between the two chains.

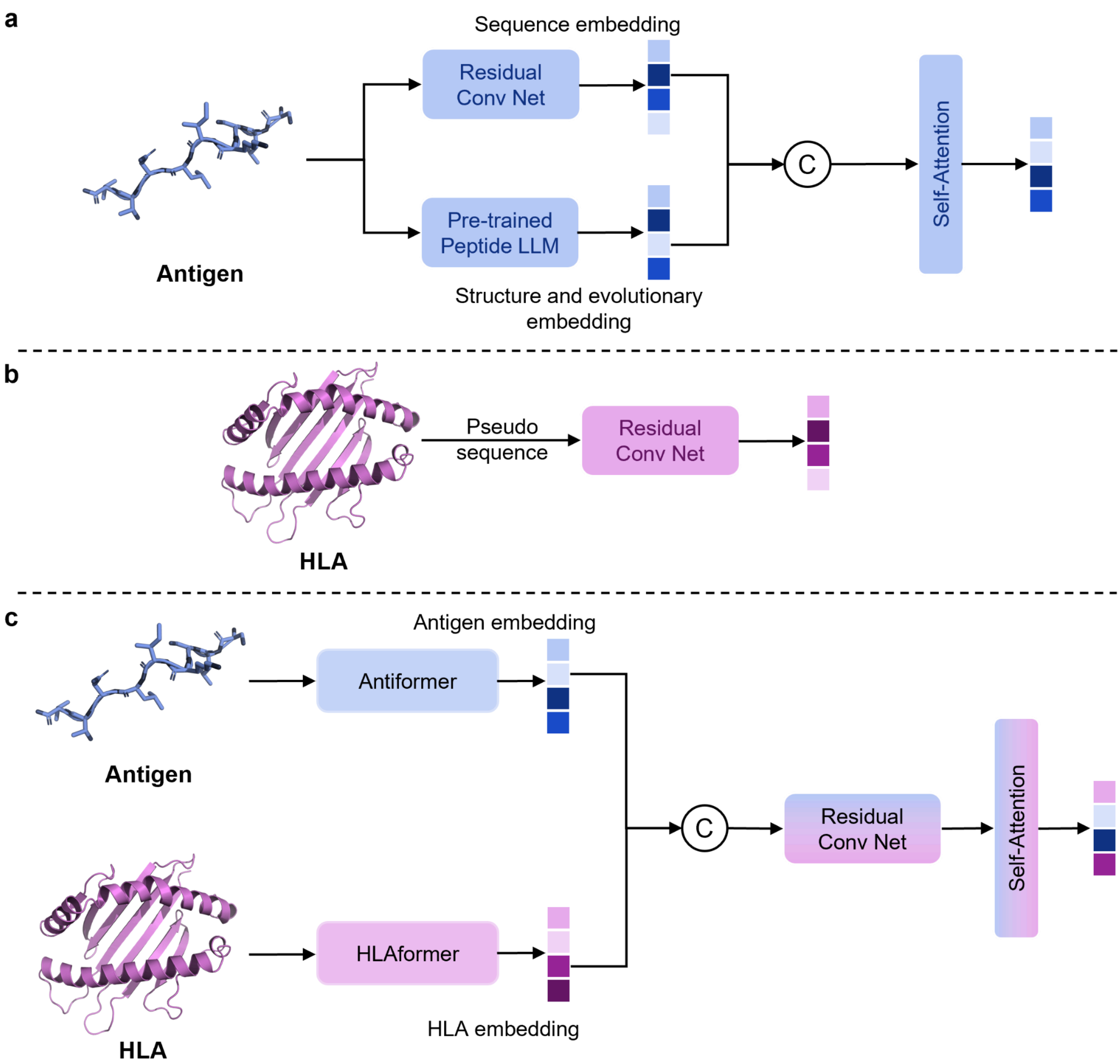

Figure S3. a-c, Detailed architectures of the Antiformer, HLAformer, and AntiHLAFusionformer modules. a, Schematic of the Antiformer module. The antigen peptide sequence is input into a residual convolutional neural network block to capture its sequence motifs. In parallel, evolutionary residue-level embeddings are generated using a peptide-specific protein language model. The motif features and evolutionary embeddings are fused and the combined representation is refined through a multi-head self-attention layer. **b,** Schematic of the HLAformer module. The HLA pseudo-sequence and corresponding biophysical and biochemical property features (Atchley factors, BLOSUM scores) serve as the input. A residual convolutional neural network extracts local features from the pseudo-sequence. **c,** Schematic of the AntiHLAFusionformer module. The individual representations of the antigen (from Antiformer) and HLA (from HLAformer) are concatenated. The concatenated vector is passed through a residual convolutional neural network and a multi-head self-attention layer to learn the interaction features at the antigen-HLA interface, outputting a unified representation of the antigen-HLA complex.

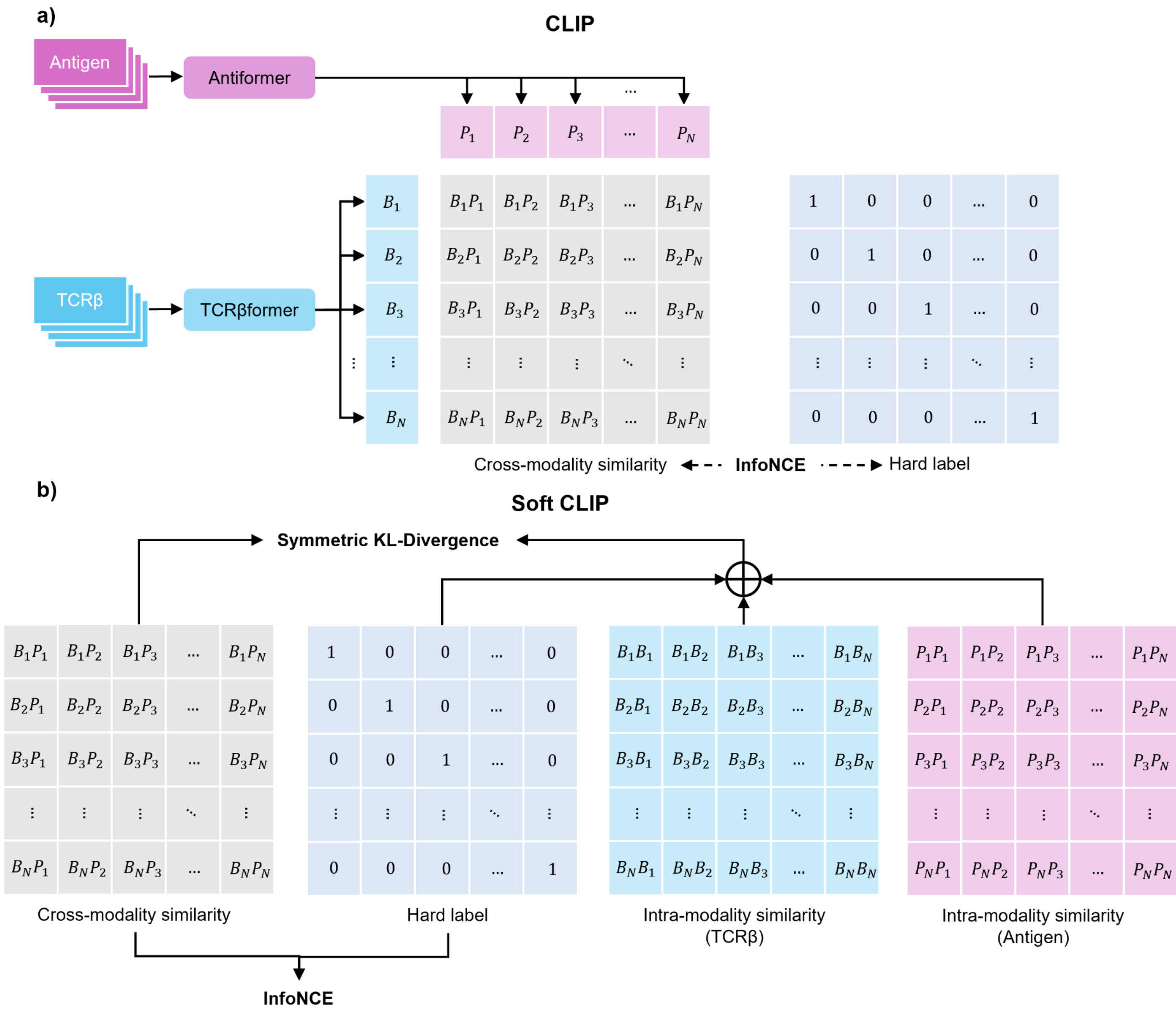

Figure S4. Schematic comparison of the original CLIP and the proposed SoftCLIP strategy. a, The standard CLIP approach utilizes the InfoNCE (Information Noise Contrastive Estimation) loss, which enforces strict alignment between positive pairs (diagonal elements, labeled 1) and penalizes all other pairs as hard negatives (labeled 0). **b,** The proposed SoftCLIP strategy incorporates biological similarity to mitigate false negatives. The circled "+" symbol represents the weighted addition of the hard labels (ground truth) and the intra-modal similarity matrices (derived from sequence homology) to create "softened" target labels. This allows the model to learn that biologically similar sequences (*e.g.*, homologous antigens) may share binding potential, rather than treating them as unrelated negatives. While this schematic illustrates the TCRβ-antigen pair, the same SoftCLIP mechanism is applied to TCRα-Antigen pairs and the alignment of fused representations in pretraining stage 2.

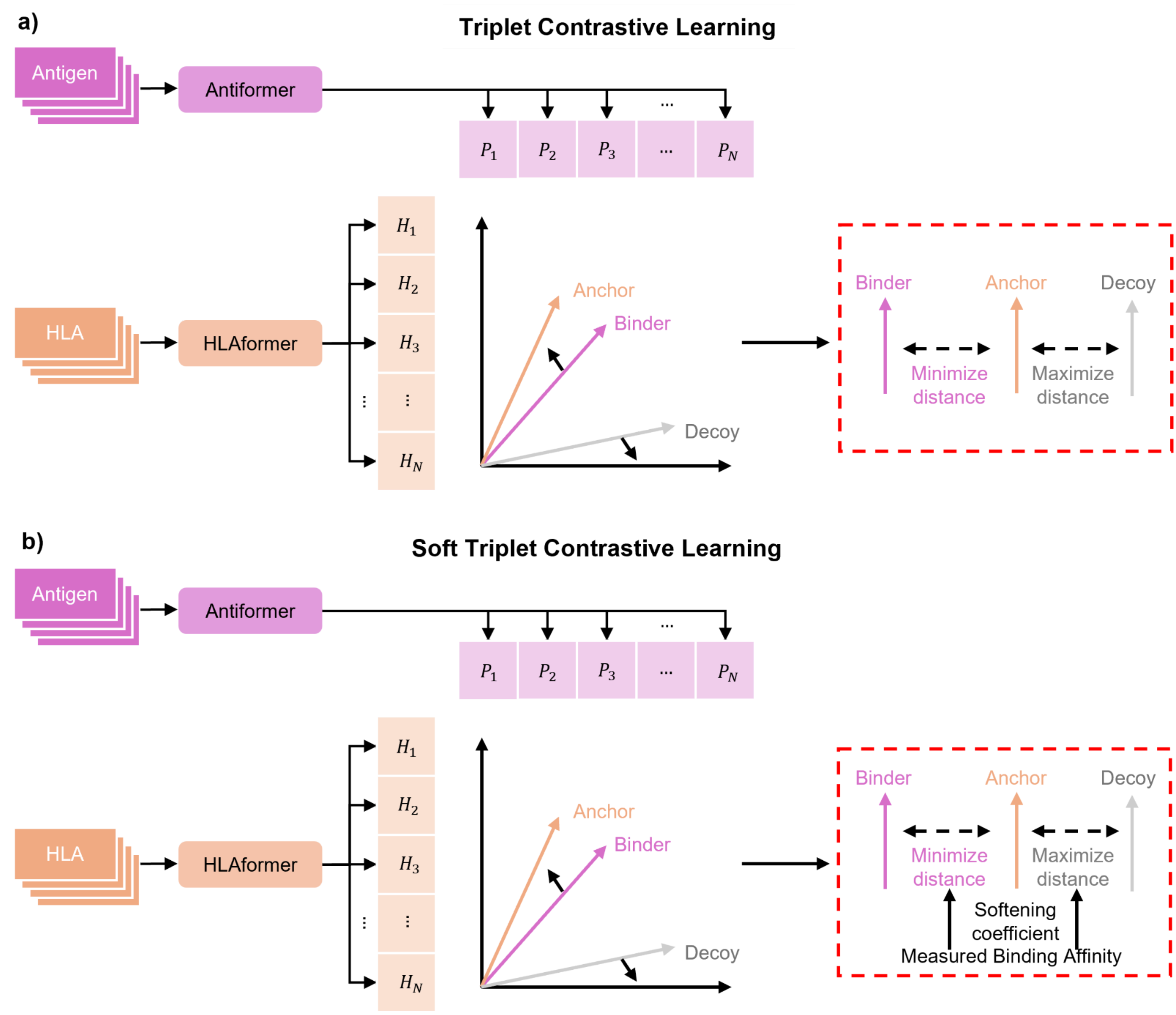

Figure S5. Comparison of the original triplet contrastive learning (a) and the proposed soft triplet contrastive learning strategy (b). a, Schematic of standard triplet contrastive learning. The method employs a fixed margin to minimize the distance between an anchor (HLA) and a positive sample (binding antigen) while maximizing the distance to a negative sample (non-binding antigen). This approach treats all negative samples equally, regardless of their actual binding affinity to the HLA molecule. **b,** Architecture of the proposed SoftTri loss function, which incorporates binding affinity measurements as weighting factors in the objective function. The positive weight is inversely proportional to the anchor-positive affinity (stronger binding receives higher weight), while the negative weight is proportional to the anchor-negative affinity (weaker binders that might still exhibit some interaction receive reduced penalty). This affinity-aware weighting scheme enables the model to learn a more biologically realistic embedding space that reflects the continuous nature of binding affinity rather than treating it as a binary phenomenon.

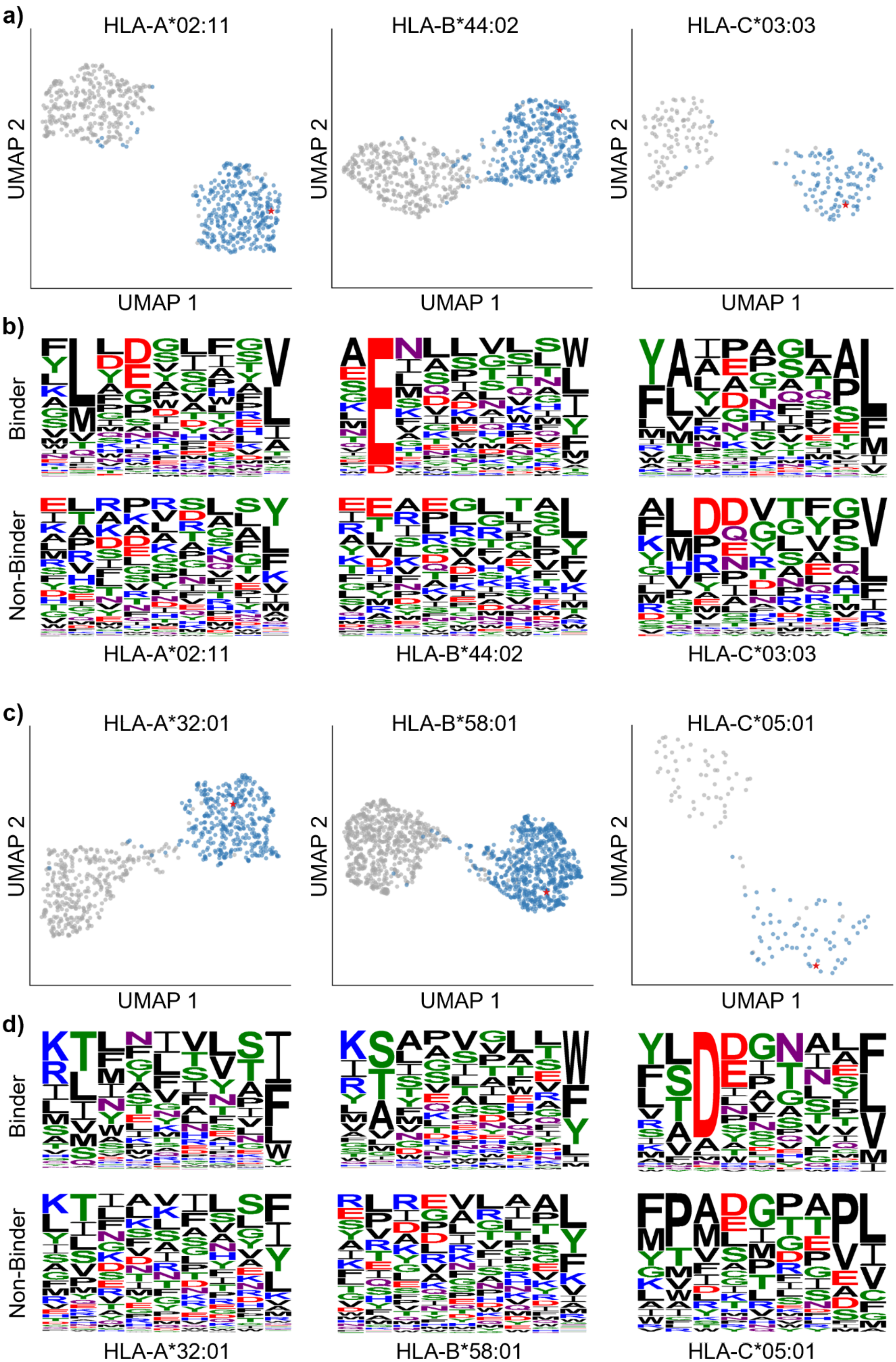

a)
HLA-A*02:11
HLA-B*44:02
HLA-C*03:03
UMAP 2
UMAP 1
b)
Binder
Non-Binder
HLA-A*02:11
HLA-B*44:02
HLA-C*03:03
c)
HLA-A*32:01
HLA-B*58:01
HLA-C*05:01
UMAP 2
UMAP 1
d)
Binder
Non-Binder
HLA-A*32:01
HLA-B*58:01
HLA-C*05:01

Figure S6. a, UMAP projections of the HLA-specific antigen embeddings, including different HLA locus, *i.e.* HLA-A*02:11 (left), HLA-B*44:02 (middle), and HLA-C*03:03 (right). The HLA, binders, and non-binders are colored in red, blue, and gray, respectively. **b**, Sequence motifs of antigen binders and non-binders targeting HLA-A*02:11 (left), HLA-B*44:02 (middle), and HLA-C*03:03 (right). **c,** UMAP projections of the HLA-specific antigen embeddings, including different HLA locus, *i.e.* HLA-A*32:01 (left), HLA-B*58:01 (middle), and HLA-C*05:01 (right). The HLA, binders, and non-binders are colored in red, blue, and gray, respectively. **d**, Sequence motifs of antigen binders and non-binders targeting HLA-A*32:01 (left), HLA-B*58:01 (middle), and HLA-C*05:01 (right).

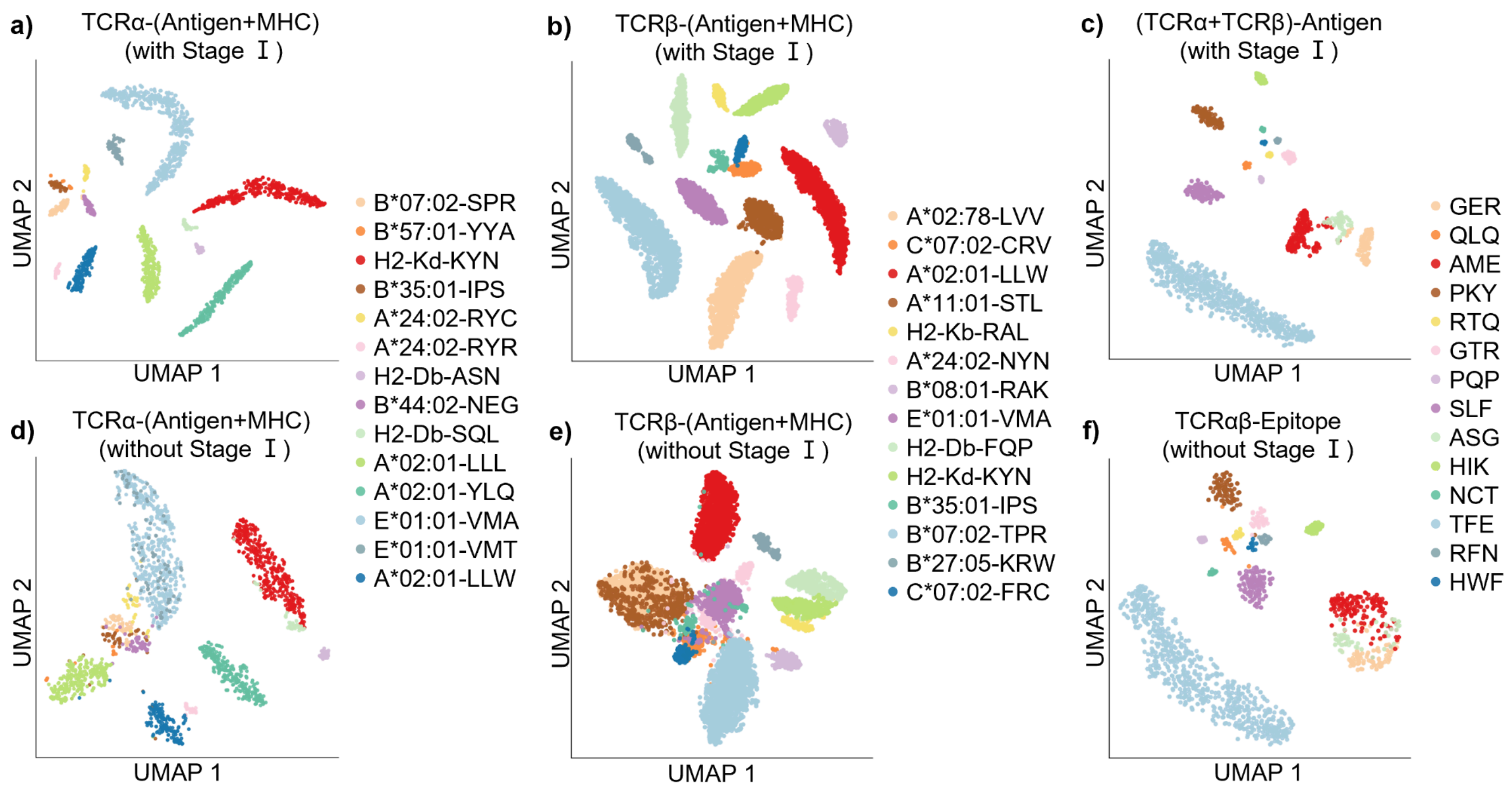

Figure S7. a-c, UMAP projections of the predicted antigen-HLA-specific TCRα clusters (**a**), the predicted antigen-HLA-specific TCRβ clusters (**b**), and the predicted TCRα-TCRβ-specific antigen clusters (**c**) with stage 1 pretraining. **d-e**, UMAP of the predicted antigen-HLA-specific TCRα clusters (**d**), the predicted antigen-HLA-specific TCRβ clusters (**e**), and the predicted TCRα-TCRβ-specific antigen clusters (**f**) without stage 1 pretraining.

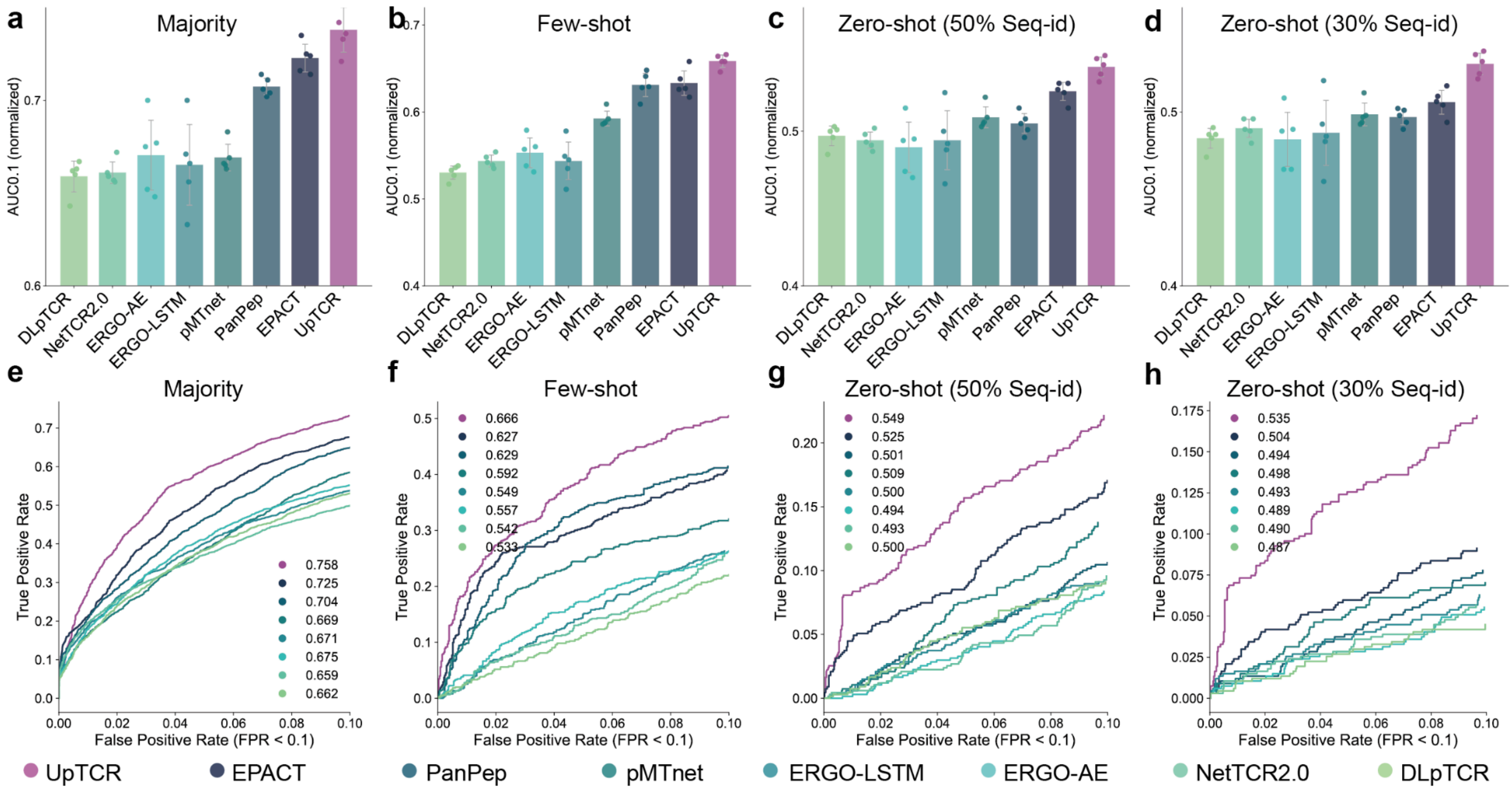

Figure S8. Early retrieval performance assessed by partial AUC at a false positive rate of 0.1 (AUC0.1) across four evaluation settings. a-d, Bar plots of normalized AUC0.1 for all benchmark methods under the majority (**a**), few-shot (**b**), zero-shot with 50% sequence identity filtering (**c**), and zero-shot with 30% sequence identity filtering (**d**) settings. AUC0.1 quantifies the discriminative performance within the high-specificity regime (FPR ≤ 0.1), reflecting how well each model ranks its most confident predictions. **e-h,** Corresponding partial ROC curves (FPR ≤ 0.1) for the majority (**e**), few-shot (**f**), zero-shot with 50% sequence identity filtering (**g**), and zero-shot with 30% sequence identity filtering (**h**) settings. UpTCR consistently achieves the highest AUC0.1 across all settings. Individual data points from *n* = 5 independent runs are overlaid.

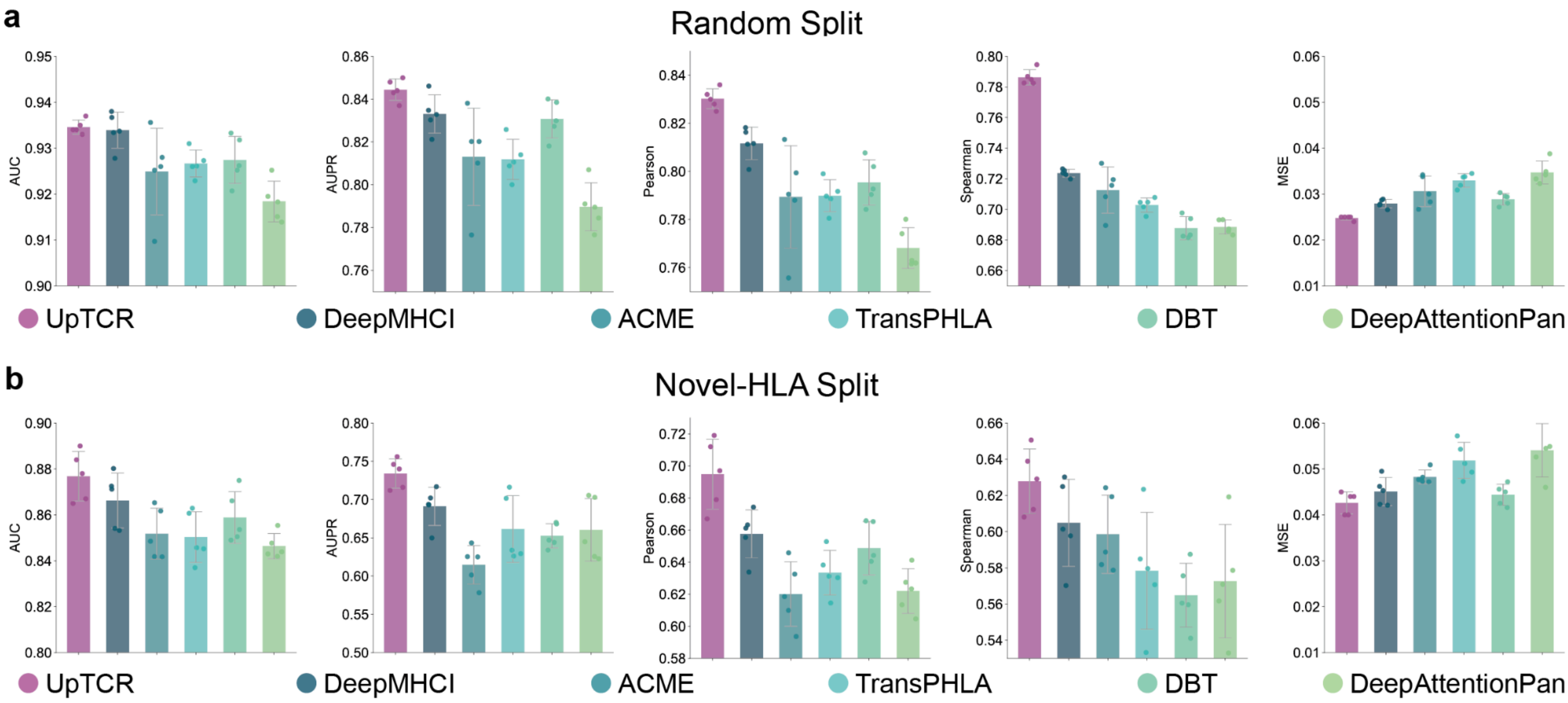

Figure S9. a,b, Bar plots of HLA-antigen binding affinity prediction performance of the baseline methods for predicting antigen-HLA binding affinity under random split (**e**) and novel-HLA split (**f**) settings. From left to right: AUC, AUPR, Pearson, Spearman, and MSE. The bars represent the average metric and the error bars indicate the standard deviations. Individual data points from n = 5 independent runs are overlaid.

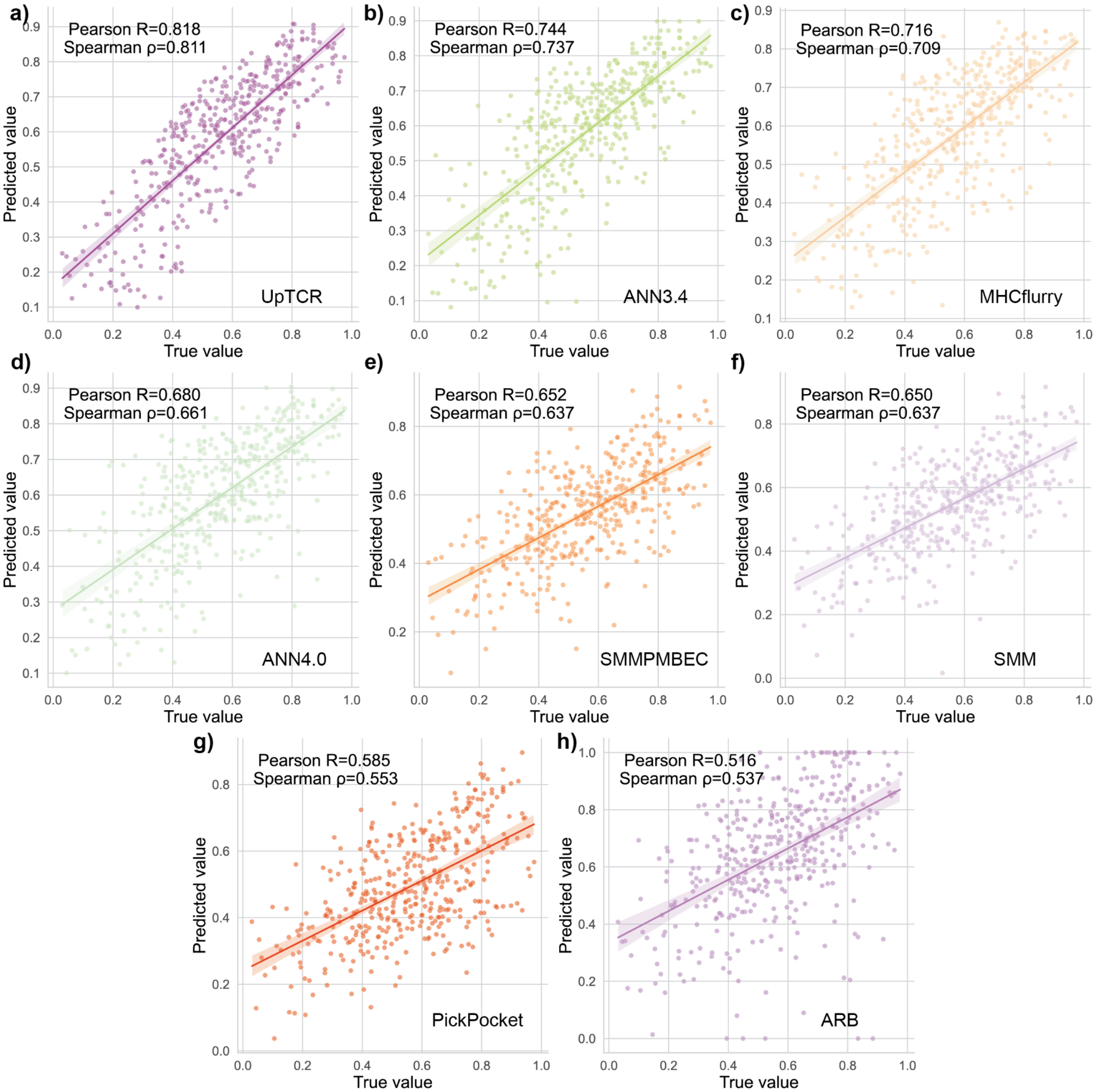

Figure S10. a-h, Performance of UpTCR (**a**) with several well-established state-of-the-art approaches, including ANN3.4 (**b**), MHCflurry (**c**), ANN4.0 (**d**), SMMPMBEC (**e**), SMM (**f**), PickPocket (**g**), and ARB (**h**), on antigen-HLA independent test data in terms of Pearson correlation and Spearman correlation. The independent test data were acquired from weekly data with IC_{50} binding affinity measurements in the IEDB database. The duplicate data with the training set were removed to avoid data leakage.

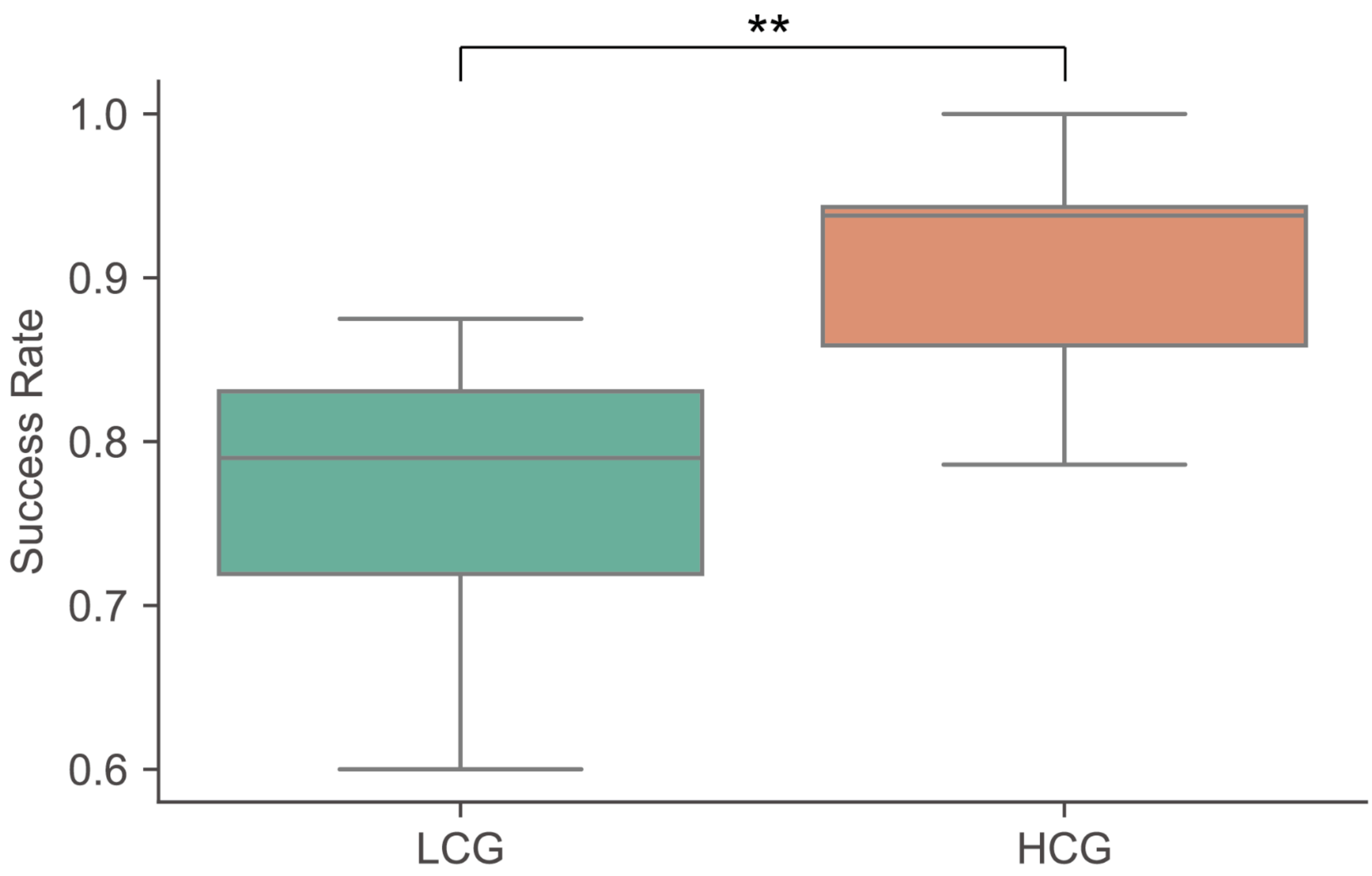

Figure S11. Distribution of success rate of UpTCR in predicting TCR cross-reactivity in terms of low-conservation group (LCG, left) and high-conservation group (HCG, right), n = 10 TCR groups per class. A significant difference was observed in the two groups, with a P-value < 0.01. ** indicates P < 0.01. Two-sided unpaired Student's t-test, P = 0.0016.

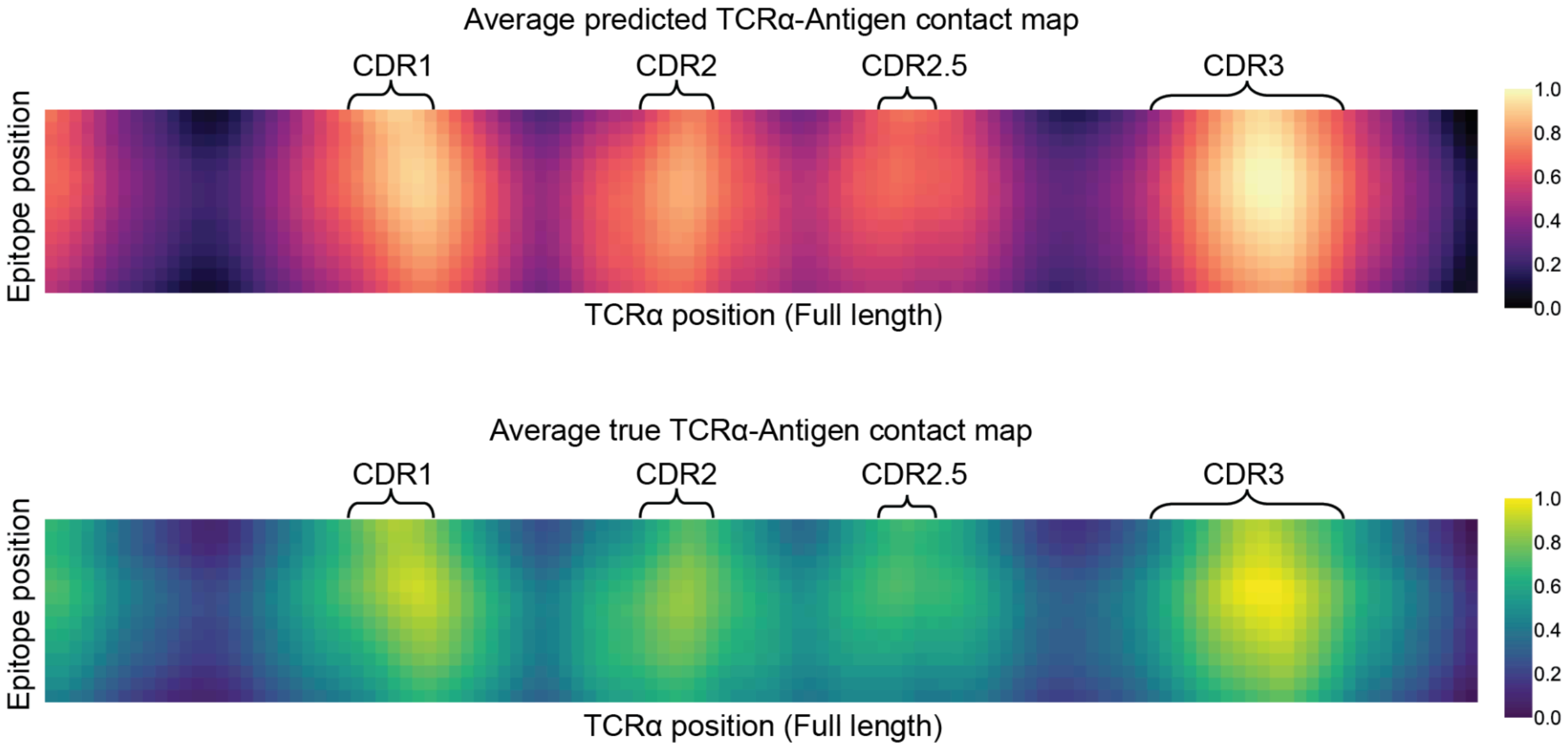

Figure S12. TCRα-Antigen residue contact relation based on UpTCR predictions (red) and crystal structures (green), both averaged over 164 complexes.

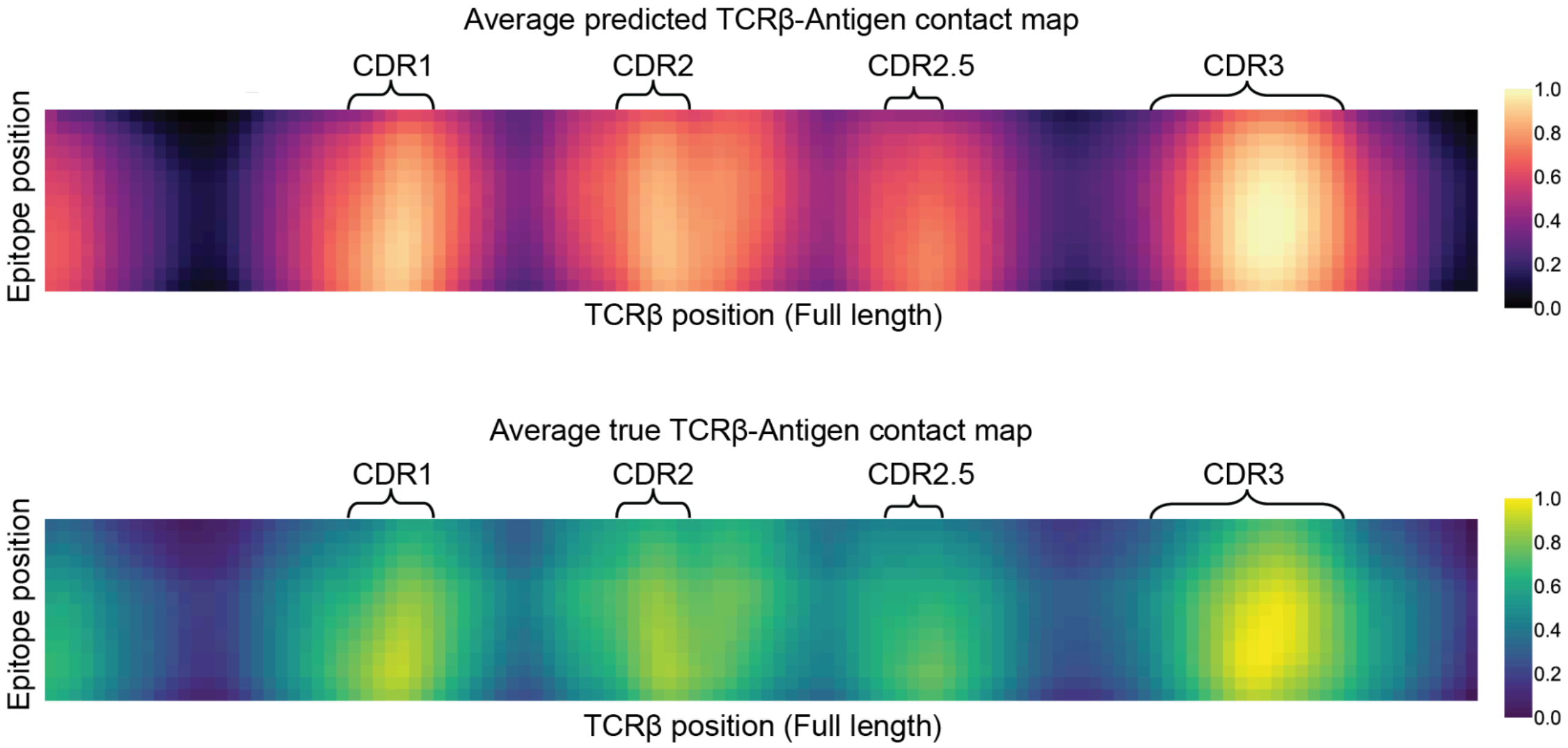

Figure S13. TCRβ-Antigen residue contact relation based on UpTCR predictions (red) and crystal structures (green), both averaged over 164 complexes.

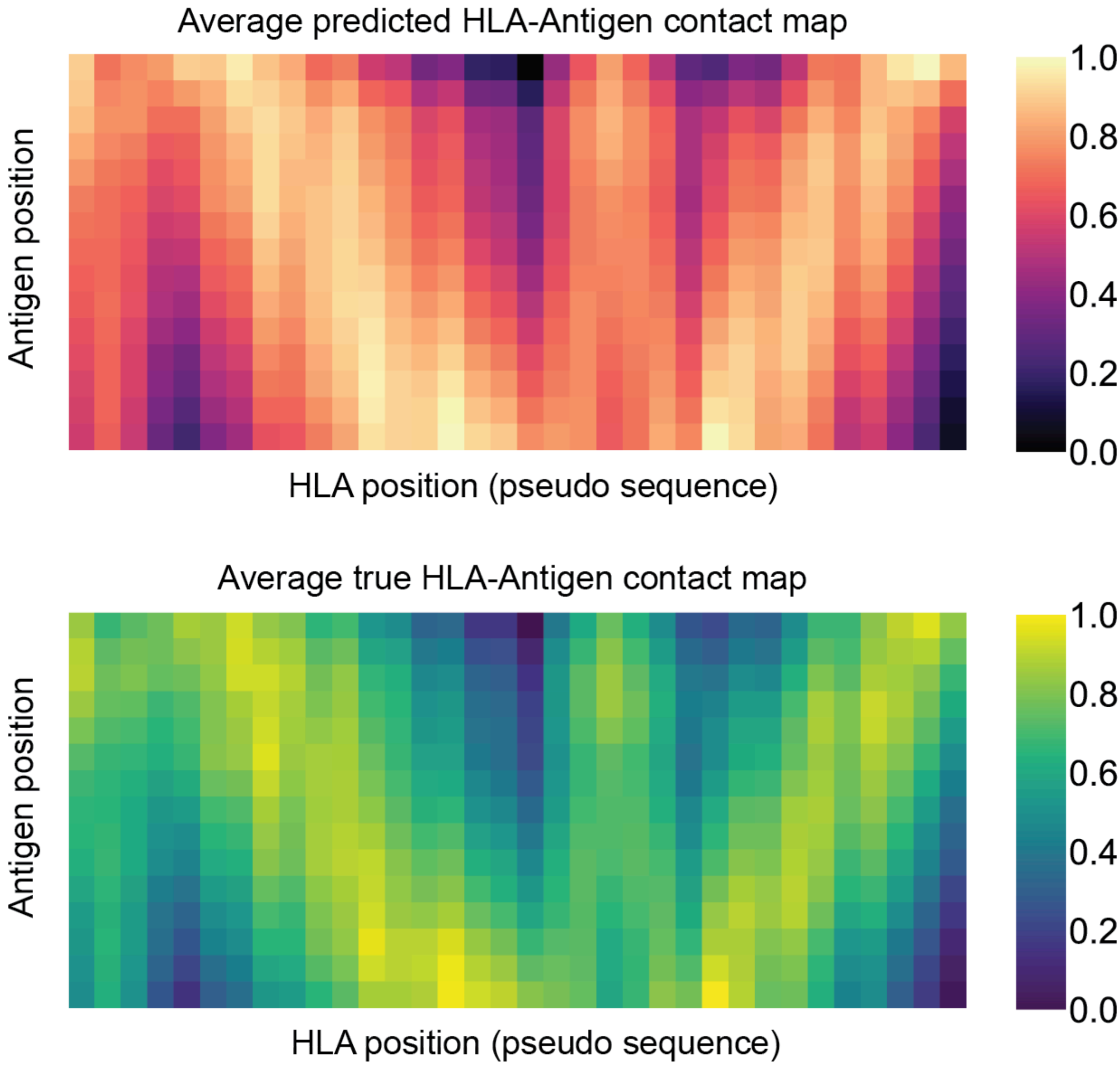

Figure S14. HLA-Antigen residue contact relation based on UpTCR predictions (red) and crystal structures (green), both averaged over 164 complexes.

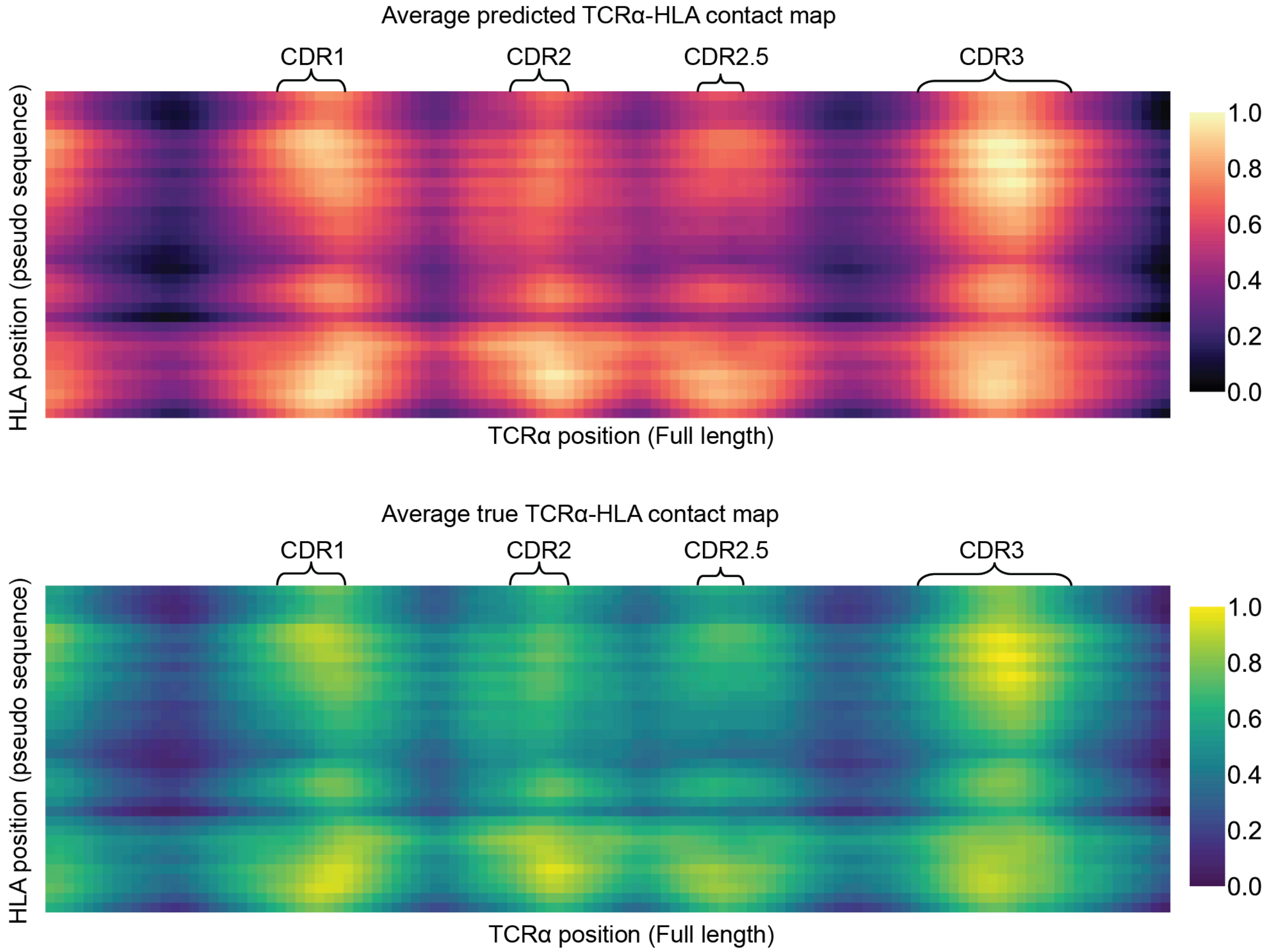

Figure S15. TCRα-HLA residue contact relation based on UpTCR predictions (red) and crystal structures (green), both averaged over 164 complexes.

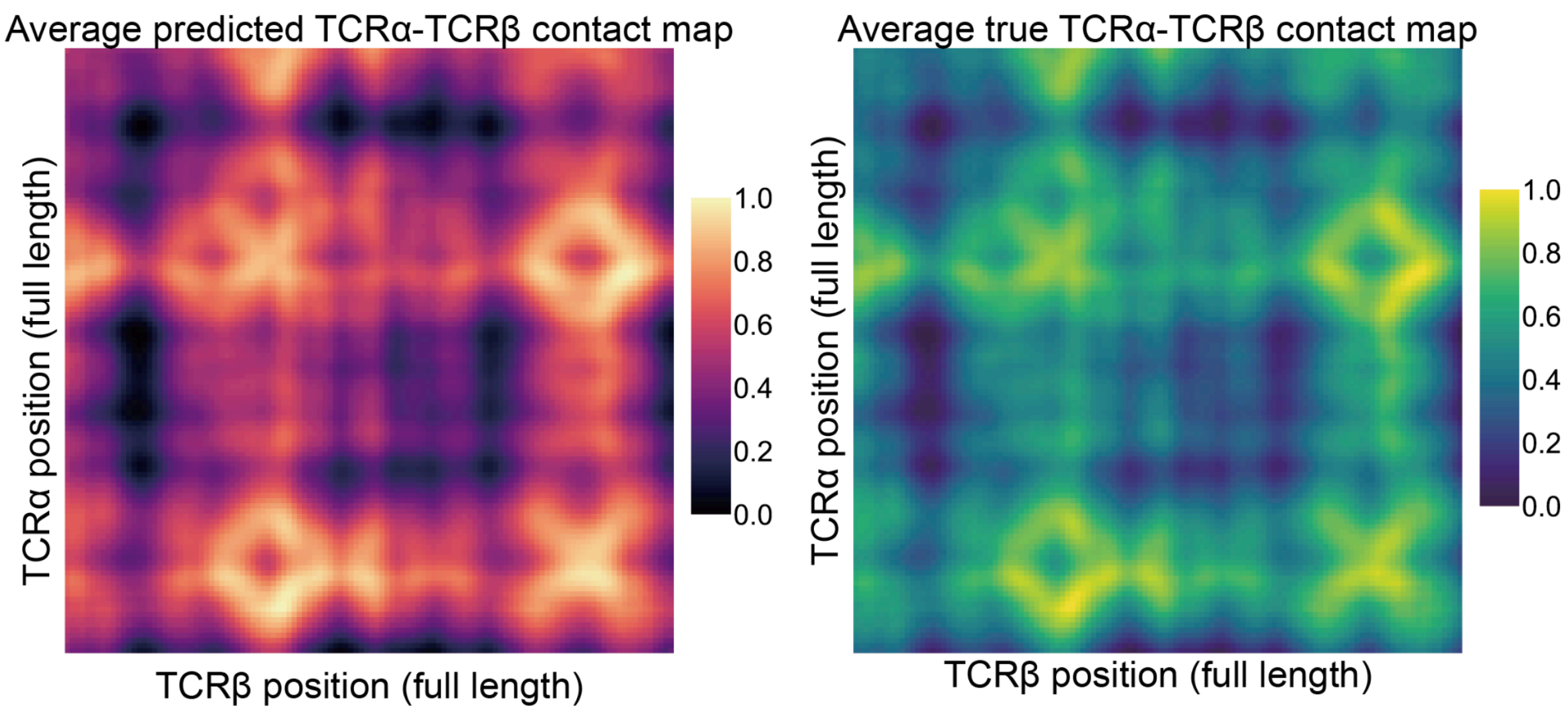

Figure S16. TCRα-TCRβ residue contact relation based on UpTCR predictions (red) and crystal structures (green), both averaged over 164 complexes.

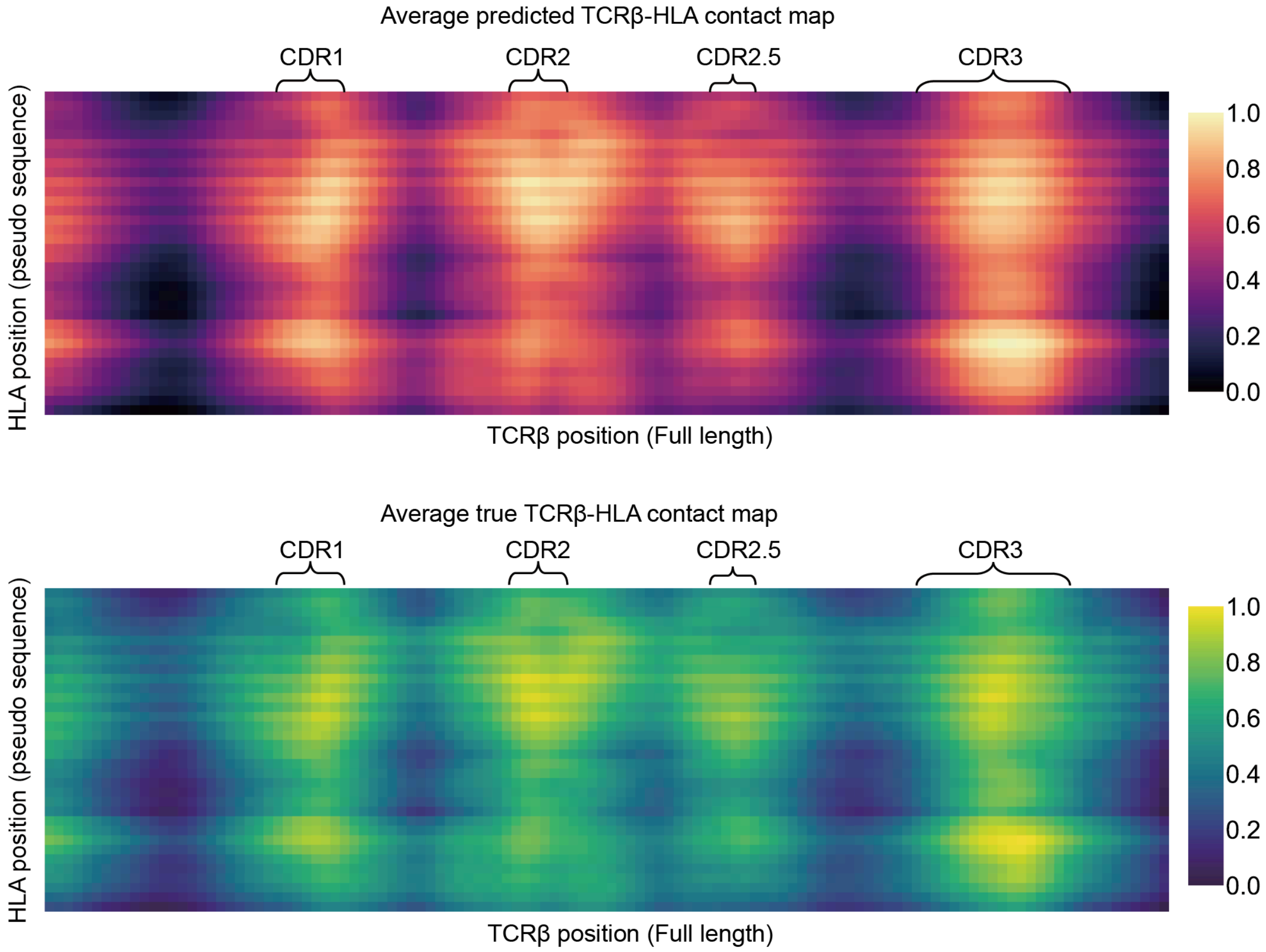

Figure S17. TCRβ-HLA residue contact relation based on UpTCR predictions (red) and crystal structures (green), both averaged over 164 complexes.

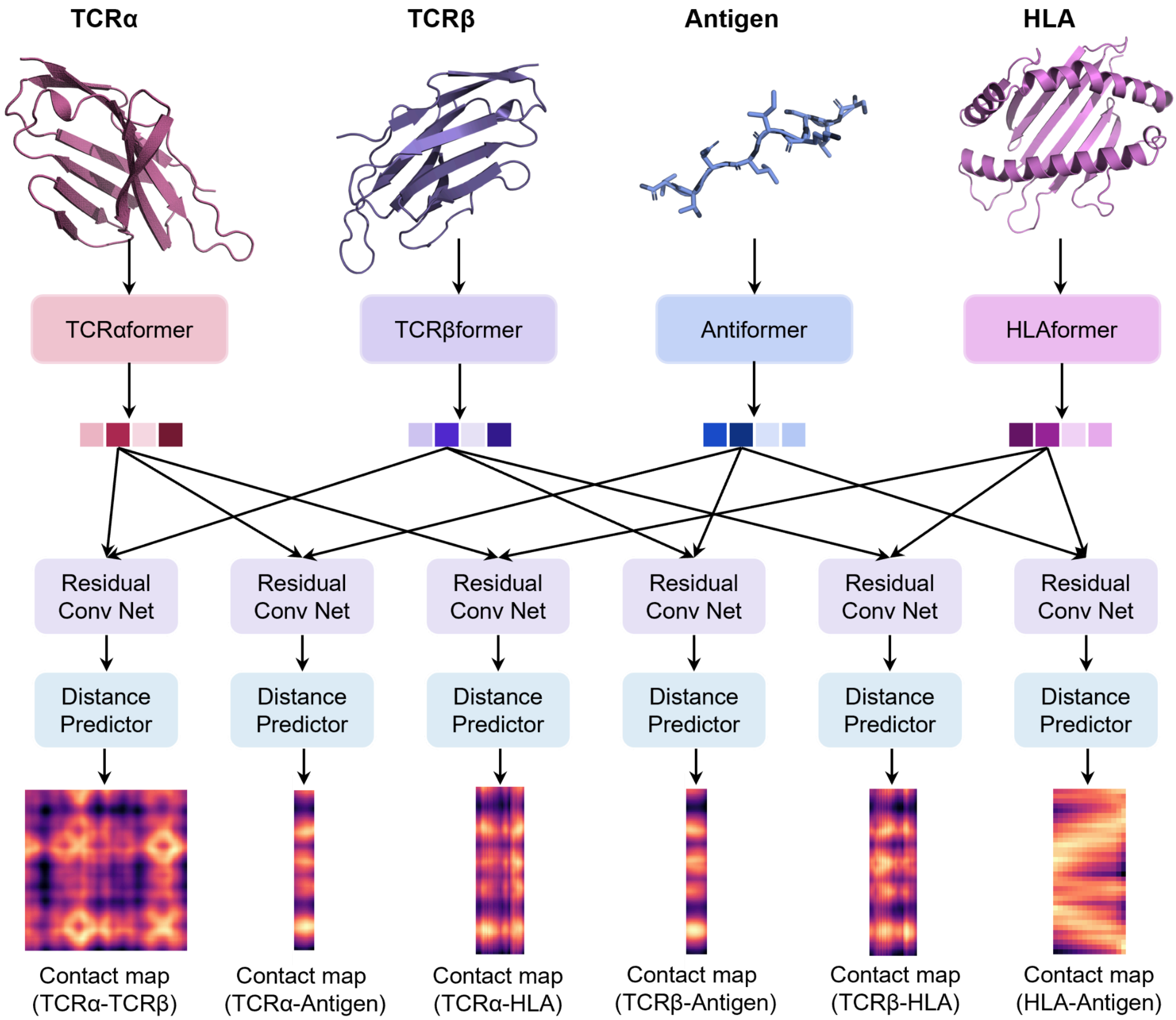

Figure S18. Detailed architectures of the interaction conformation prediction module. The schematic illustrates the neural network designed to predict residue-level contact maps for any pairwise interaction within the TCR–antigen–HLA complex. The module takes four molecular components as inputs: TCRα, TCRβ, antigen, and HLA. Each component is encoded into feature embeddings by its corresponding pretrained encoder—TCRαformer, TCRβformer, Antiformer, and HLAformer, respectively. These embeddings are processed in parallel through six residual convolutional network blocks, each dedicated to a unique molecular pair: TCRα–TCRβ, TCRα–antigen, TCRα–HLA, TCRβ–antigen, TCRβ–HLA, and antigen–HLA. Each ResCNN block is followed by a distance predictor, which estimates inter-residue distances. The final outputs are six contact maps, visualized as heatmaps. This architecture enables comprehensive and interpretable modeling of all pairwise interactions in the ternary complex.

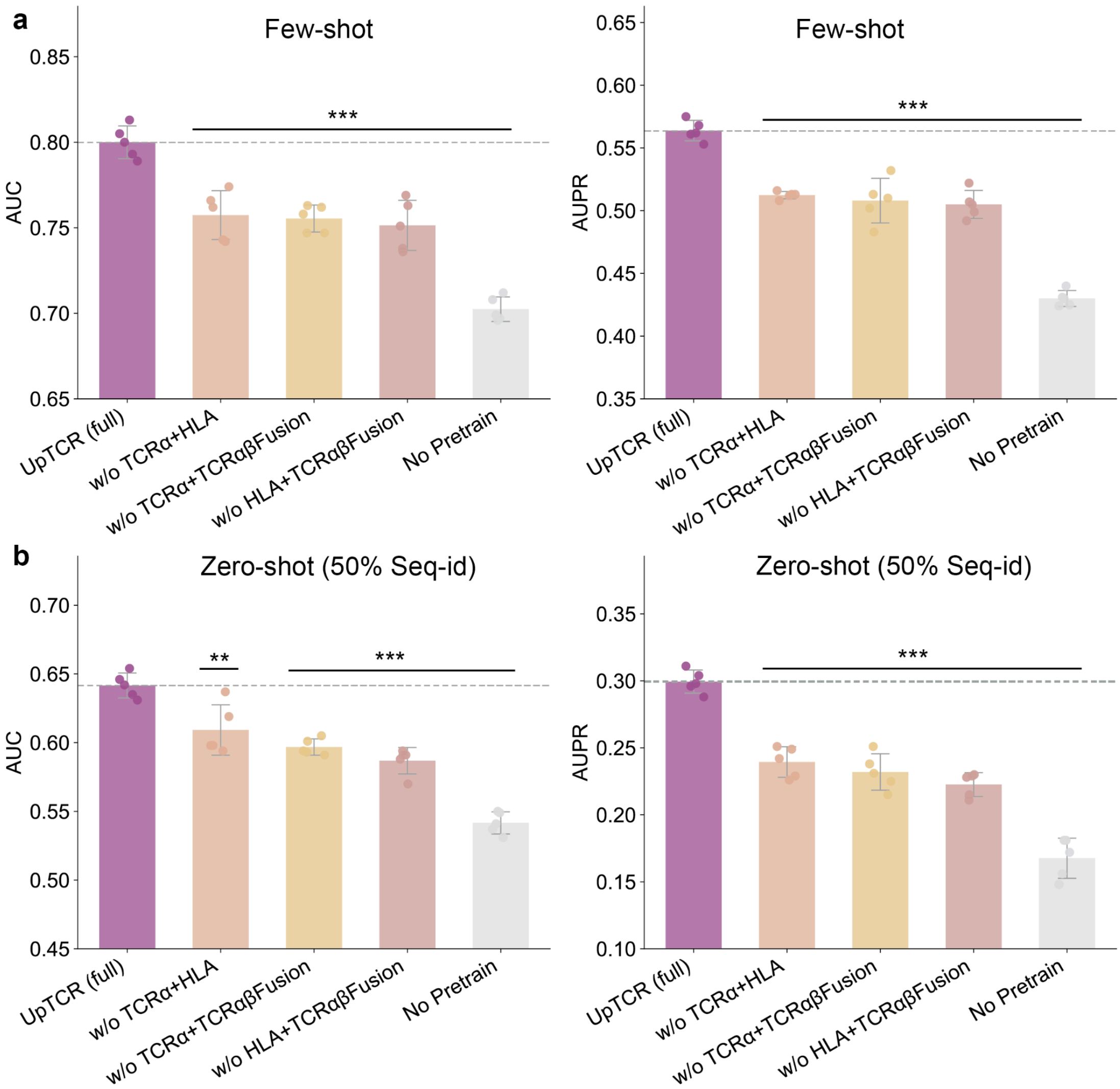

Figure S19. Multi-component pretraining ablation revealed synergistic contributions of individually non-significant modules. a, AUC (left) and AUPR (right) for TCR-antigen-HLA binding specificity prediction under the few-shot setting when pairs of individually non-significant pretrained modules (TCRαformer, HLAformer, and TCRαβFusionformer) are simultaneously ablated, alongside a fully non-pretrained variant.. **b,** Same analysis under the zero-shot setting (50% sequence identity filtering). The significant decline across all pairwise ablation combinations demonstrated that the progressive pretraining modules function synergistically. * $P < 0.05$, ** $P < 0.01$, *** $P < 0.001$. Bars show the mean and error bars show the s.d. Individual data points from $n = 5$ independent runs are overlaid. Statistical comparisons versus UpTCR used a two-sided unpaired Student's t-test.

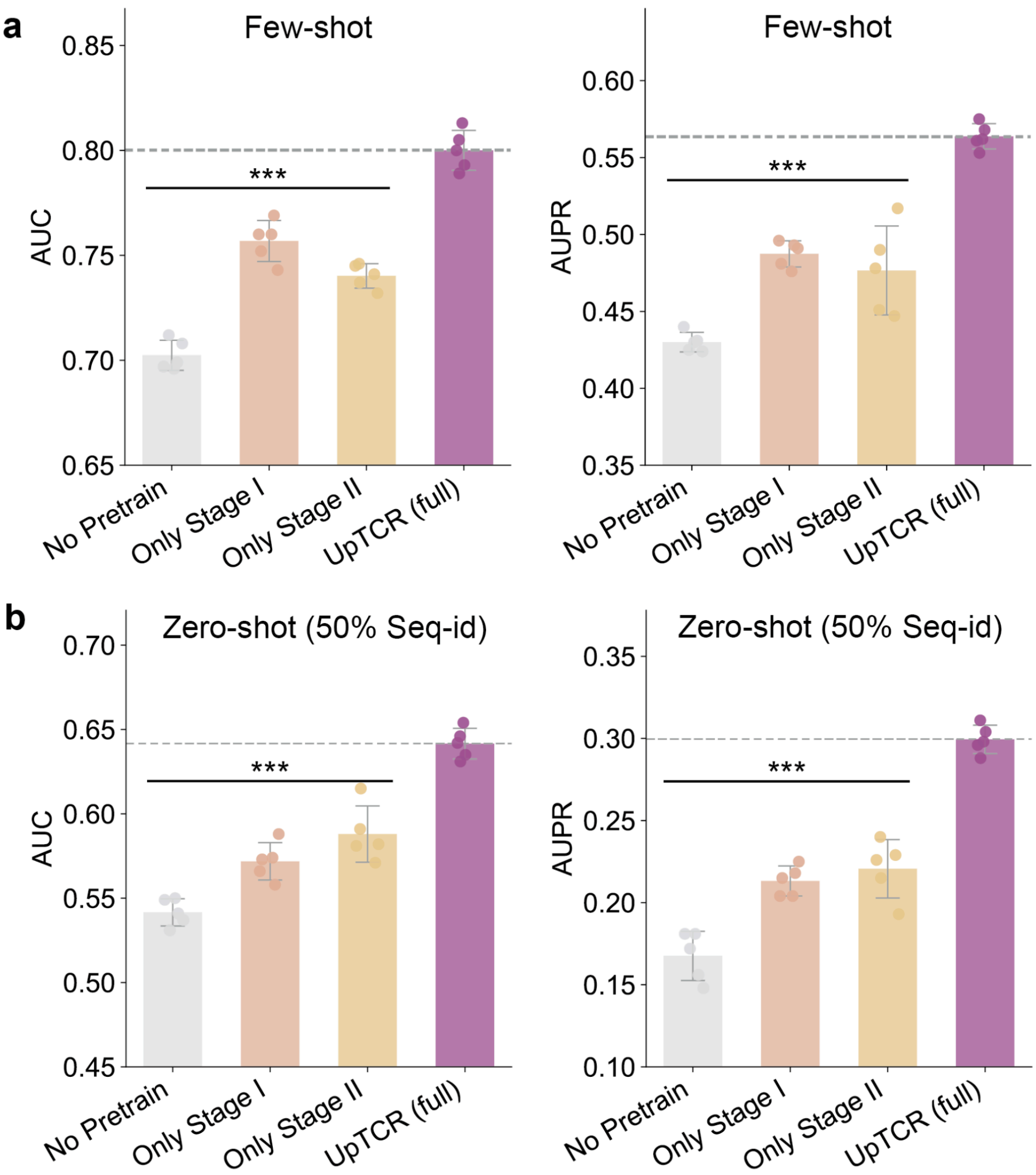

Figure S20. Stage-wise pretraining ablation demonstrated the contributions of both pretraining stages. a, AUC (left) and AUPR (right) for TCR-antigen-HLA binding specificity prediction under the few-shot setting across four pretraining configurations: No Pretrain (all encoders randomly initialized), Only Stage I (dimer-level pretraining only), Only Stage II (trimer-level pretraining only), and UpTCR (full progressive pretraining). **b,** Same analysis under the zero-shot setting (50% sequence identity filtering). *** $P < 0.001$. Bars show the mean and error bars show the s.d. Individual data points from $n = 5$ independent runs are overlaid. Statistical comparisons versus UpTCR used a two-sided unpaired Student's t-test.

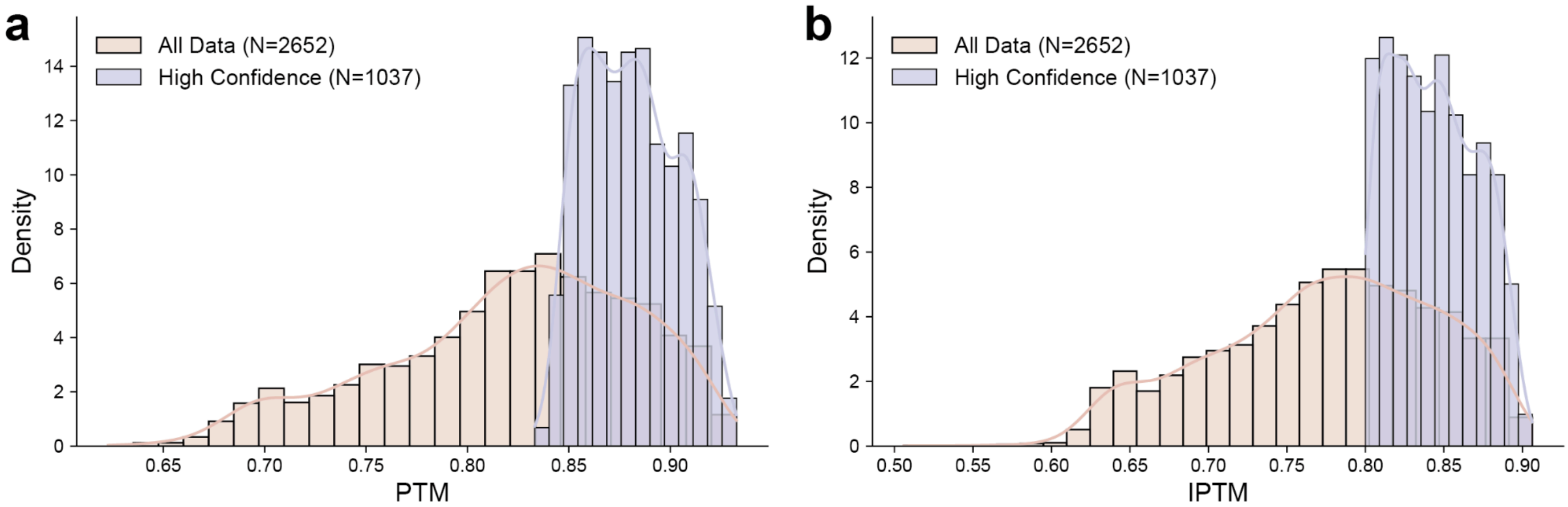

Figure S21. Quality assessment of AlphaFold-predicted TCR-antigen-HLA complex structures used for contact map training. a-b, Distribution of predicted Template Modeling (pTM) scores (**a**) and interface predicted Template Modeling (ipTM) scores (**b**) for all AlphaFold-predicted structures (orange, *n* = 2,652) and the high-confidence subset selected for training (purple, *n* = 1,037).

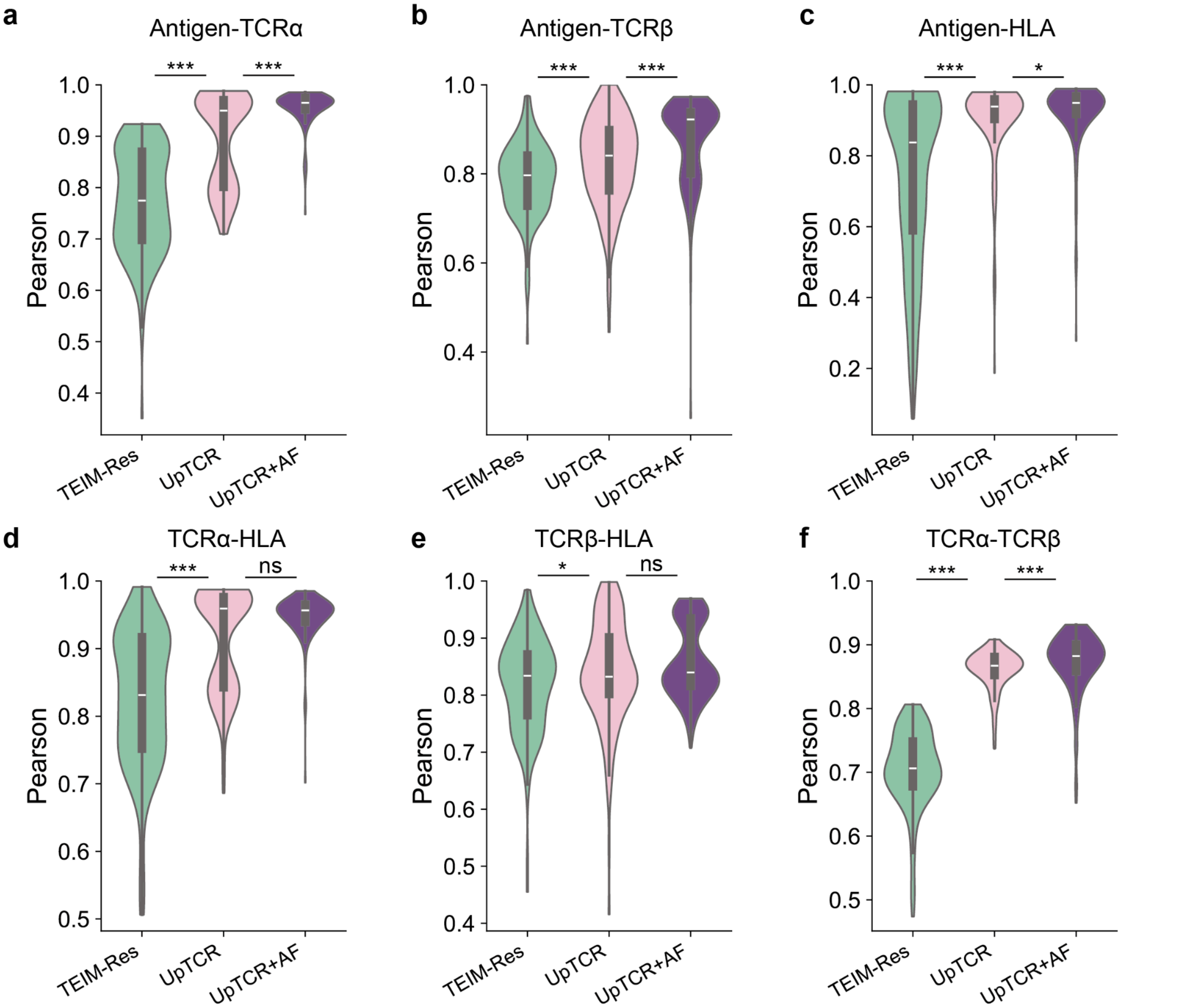

Figure S22. Pearson correlation for residue-level distance prediction across all pairwise interactions. a-b, Violin plots displaying the Pearson correlation coefficients between predicted and ground-truth residue-pair distances for TEIM-Res, UpTCR, and UpTCR+AF across six pairwise interaction types. From left to right, top row: antigen-TCRα, antigen-TCRβ, antigen-HLA; bottom row: TCRα-HLA, TCRβ-HLA, and TCRα-TCRβ. UpTCR+AF denotes UpTCR further trained with high-confidence AlphaFold-predicted complex structures. UpTCR and UpTCR+AF consistently and significantly achieve higher Pearson correlations than TEIM-Res, demonstrating superior accuracy in predicting inter-residue distances. * $P < 0.05$, ** $P < 0.01$, *** $P < 0.001$, ns indicates no significant difference.

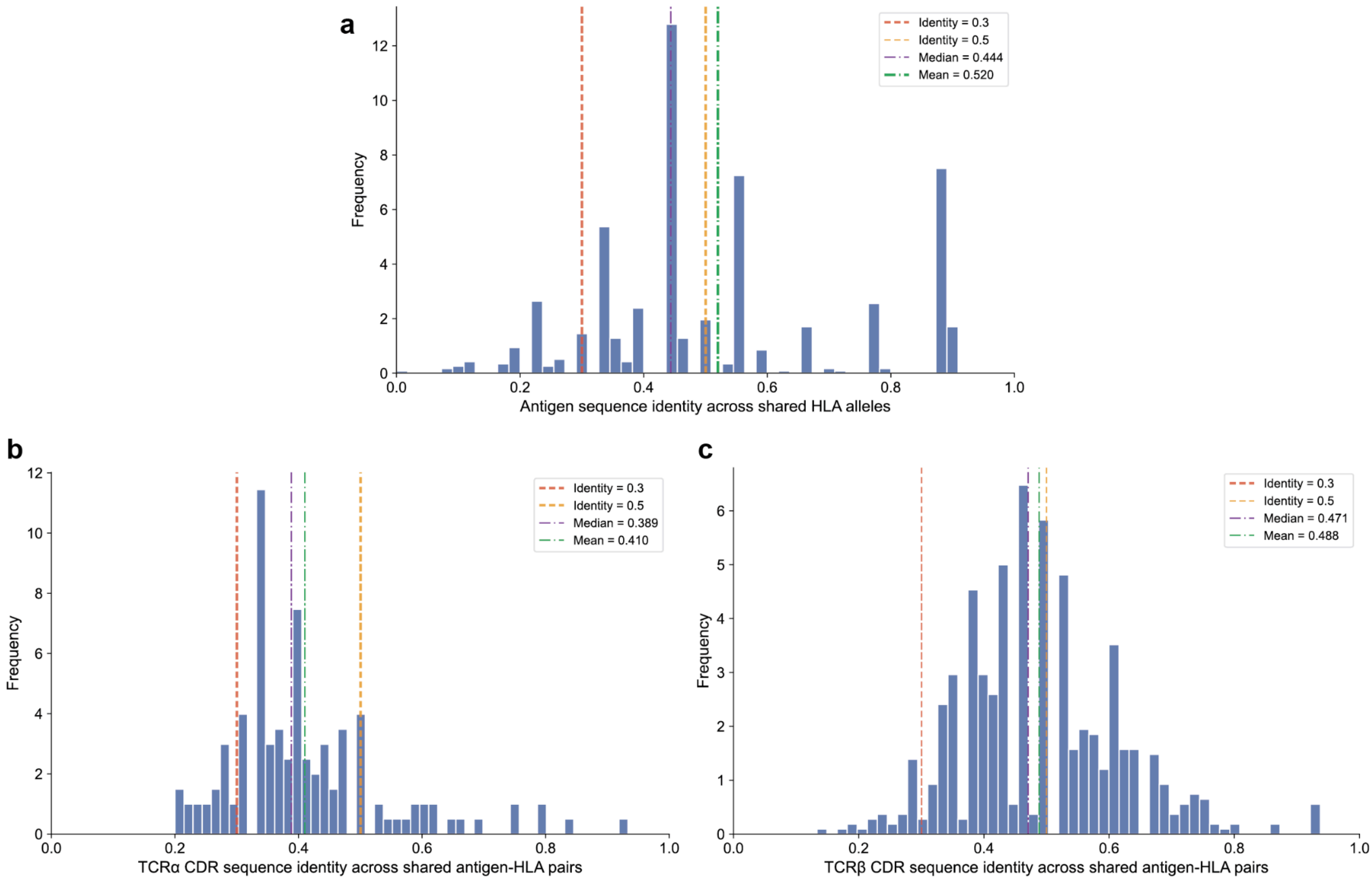

Figure S23. Pairwise sequence identity distributions among functionally related sequences. a, Distribution of pairwise antigen sequence identity among peptides that bind the same HLA allele. The median identity is 0.444 and the mean is 0.520. **b,** Distribution of pairwise TCRα CDR sequence identity among TCRs recognizing the same antigen-HLA pair. The median identity is 0.389 and the mean is 0.410, with the majority of pairs falling between the 30% and 50% thresholds. **c,** Distribution of pairwise TCRβ CDR sequence identity among TCRs recognizing the same antigen-HLA pair. The median identity is 0.471 and the mean is 0.488.

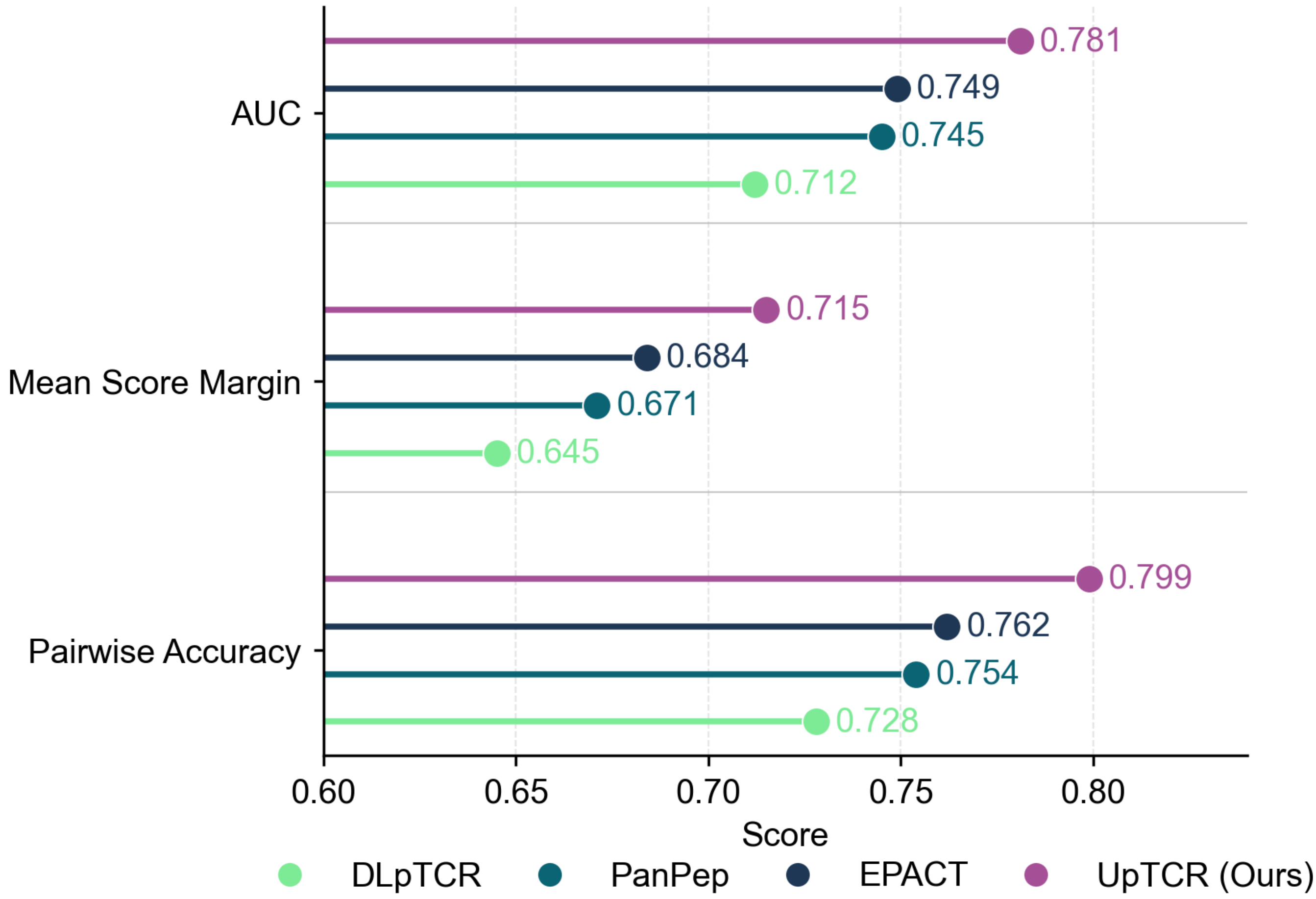

Figure S24. Hard negative discrimination performance on mutation epitope pairs. Comparison of UpTCR and three baseline methods on a hard negative benchmark consisting of 3,495 TCR-epitope pairs, where each TCR is paired with one confirmed binding epitope and one non-binding epitope differing by amino acid substitutions. Performance was assessed by three metrics: pairwise accuracy (proportion of pairs where the binding epitope received a higher predicted score than the non-binding epitope), mean score margin (average difference between positive and negative prediction scores), and area under the receiver operating characteristic curve (AUC). UpTCR consistently outperformed all baselines across all metrics.

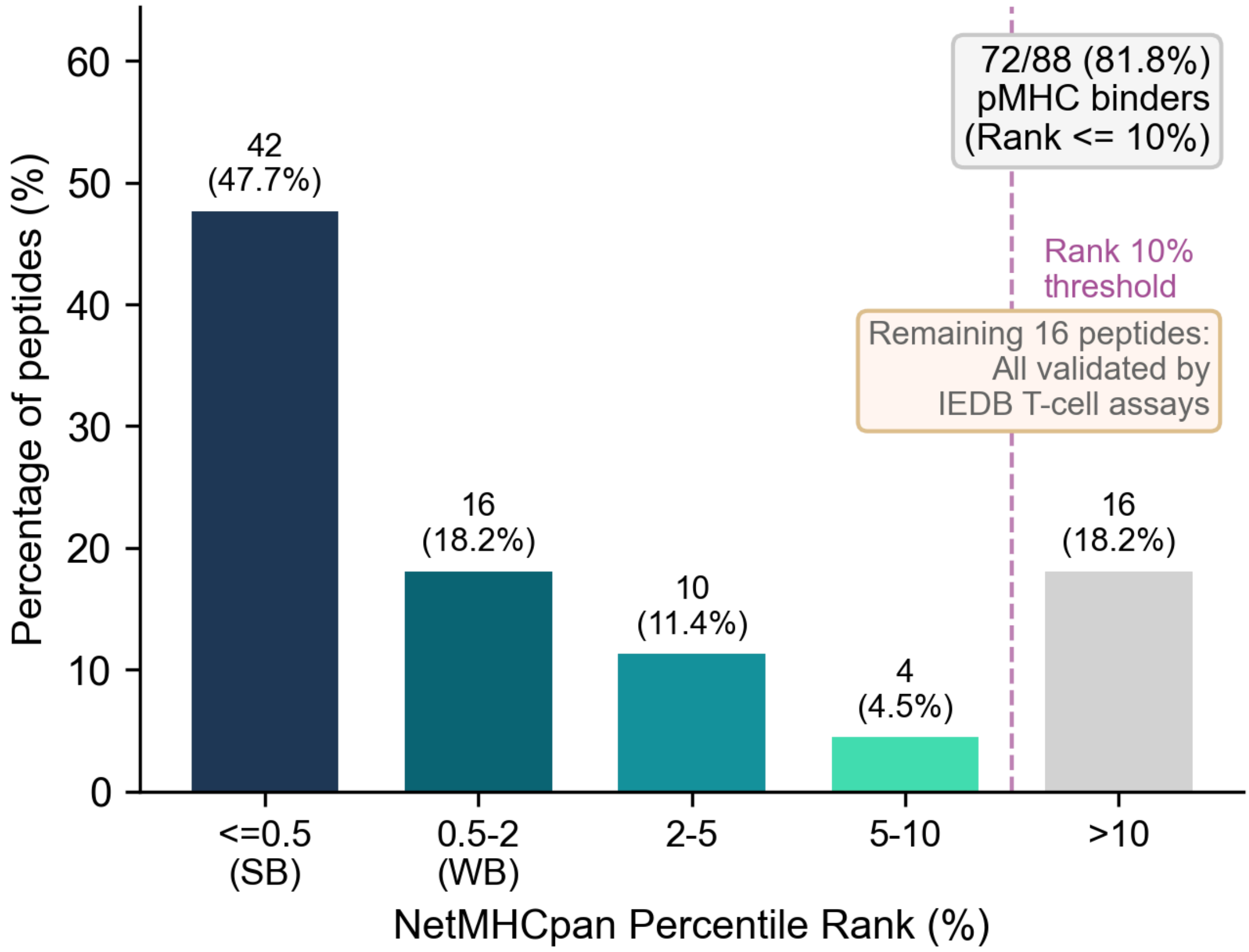

Figure S25. Predicted pMHC binding affinity of cross-reactive peptides in the low-conservation group. Distribution of NetMHCpan 4.1 predicted percentile ranks for 88 peptides from the low-conservation cross-reactivity groups (conservation ratio < 0.5). Peptides were classified as strong binders (SB; percentile rank ≤ 0.5%), weak binders (WB; 0.5% < rank ≤ 2%), or non-binders (rank > 10%) according to standard thresholds. Of the 88 peptides, 72 (81.8%) were predicted to bind their cognate HLA molecule (rank ≤ 10%). The remaining 16 peptides, predominantly cysteine-rich sequences, were independently confirmed as positive T-cell epitopes via experimentally validated T-cell assay records in the IEDB database, indicating that the prediction discrepancy reflects a known limitation of NetMHCpan for such sequences rather than a genuine absence of HLA presentation.

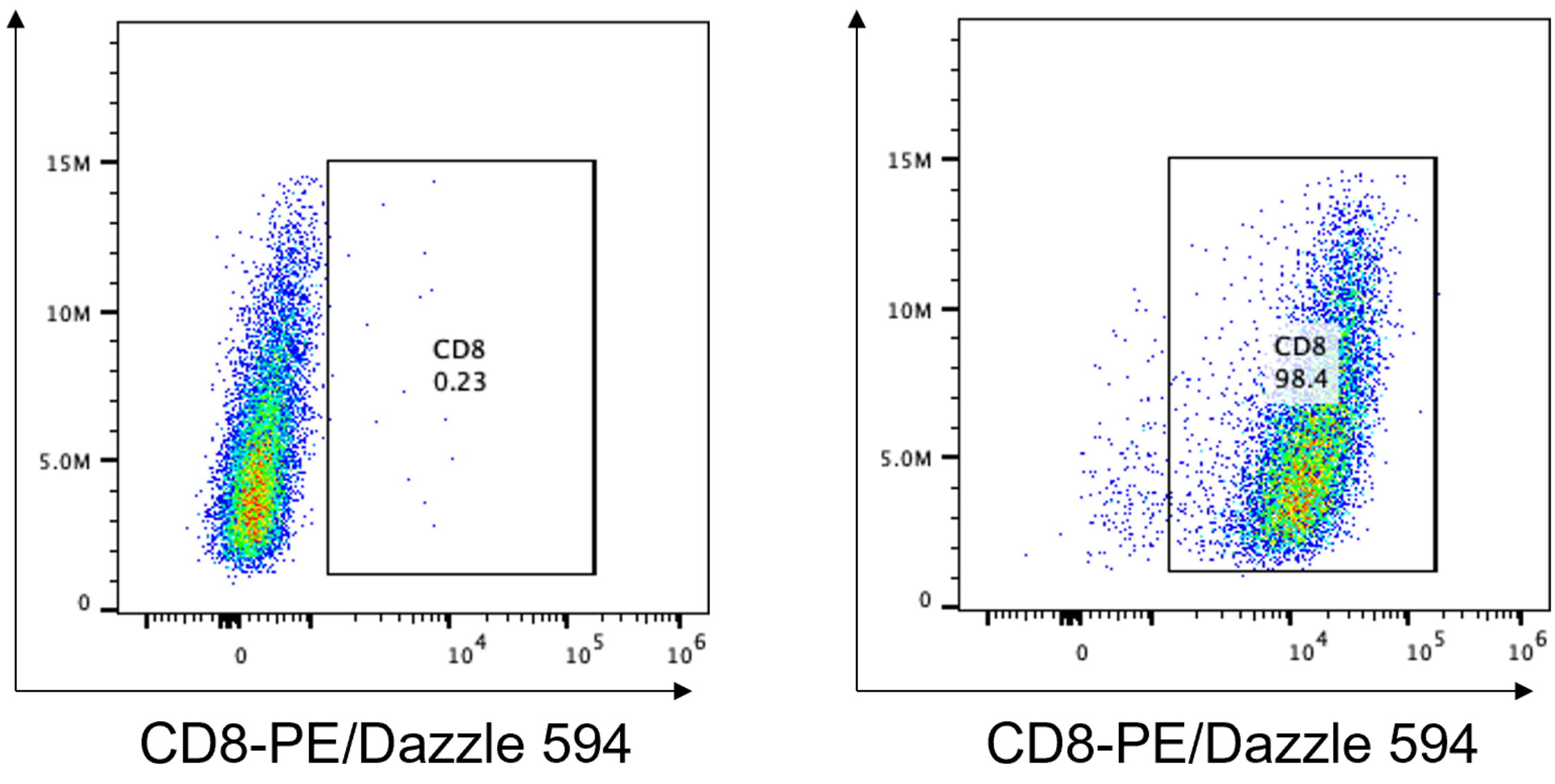

Figure S26. Flow cytometry validation of CD8 expression in engineered Jurkat T cells. Representative flow cytometry plots showing CD8 expression in parental Jurkat T cells and CD8-transduced Jurkat T cells. Parental Jurkat T cells showed negligible CD8 expression, consistent with their endogenous $CD4^{+}CD8^{-}$ phenotype, whereas CD8-transduced Jurkat T cells exhibited robust CD8 expression. These results confirm successful generation of CD8-expressing Jurkat T cells for TCR stimulation and activation assays.

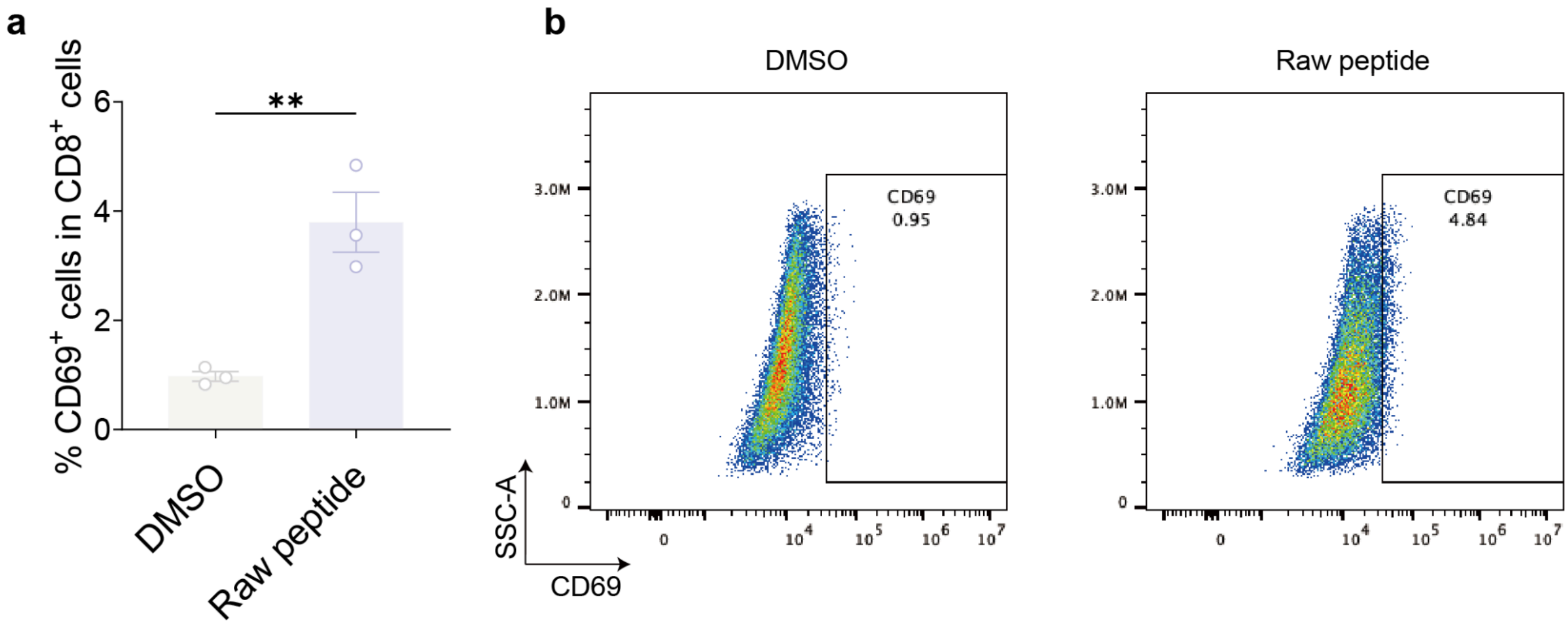

Figure S27. Flow cytometry validation of the MEL8 TCR response to the native Melan-A peptide. a, Quantification of $CD69^{+}$ cells after stimulation with DMSO or the native Melan-A peptide (Raw peptide) in MEL8 TCR-expressing cells. Data are presented as mean ± SD from three independent batches. b, Representative flow cytometry plots showing CD69 expression after DMSO or native Melan-A peptide stimulation. $^{**}P < 0.01$.

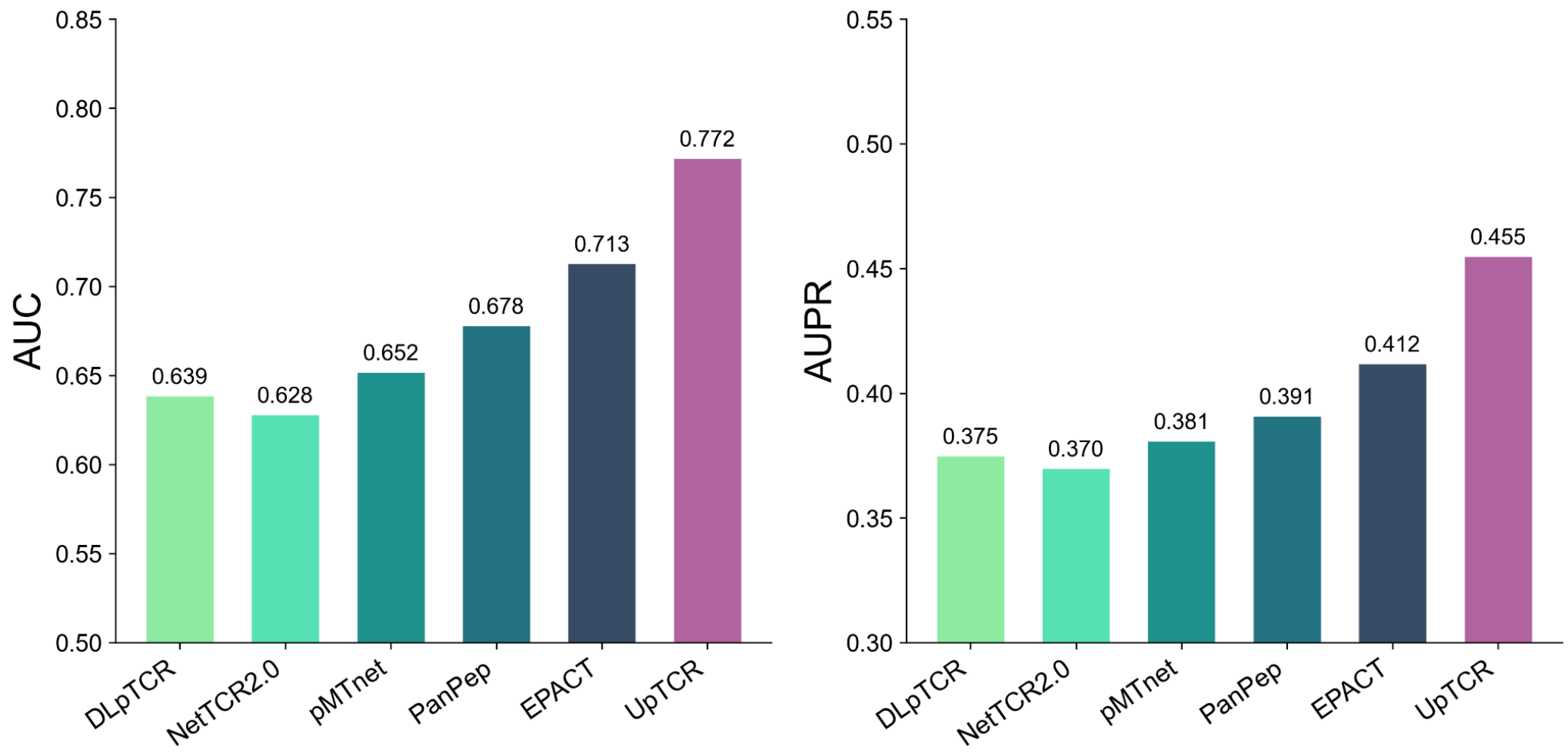

Figure S28. Performance of UpTCR and baselines on the previously unseen HLA-B*27:05-restricted autoantigen system from Wang et al.[1] UpTCR achieved higher performance than other methods, with an AUC of 0.772 (Left) and an AUPR of 0.455 (Right).

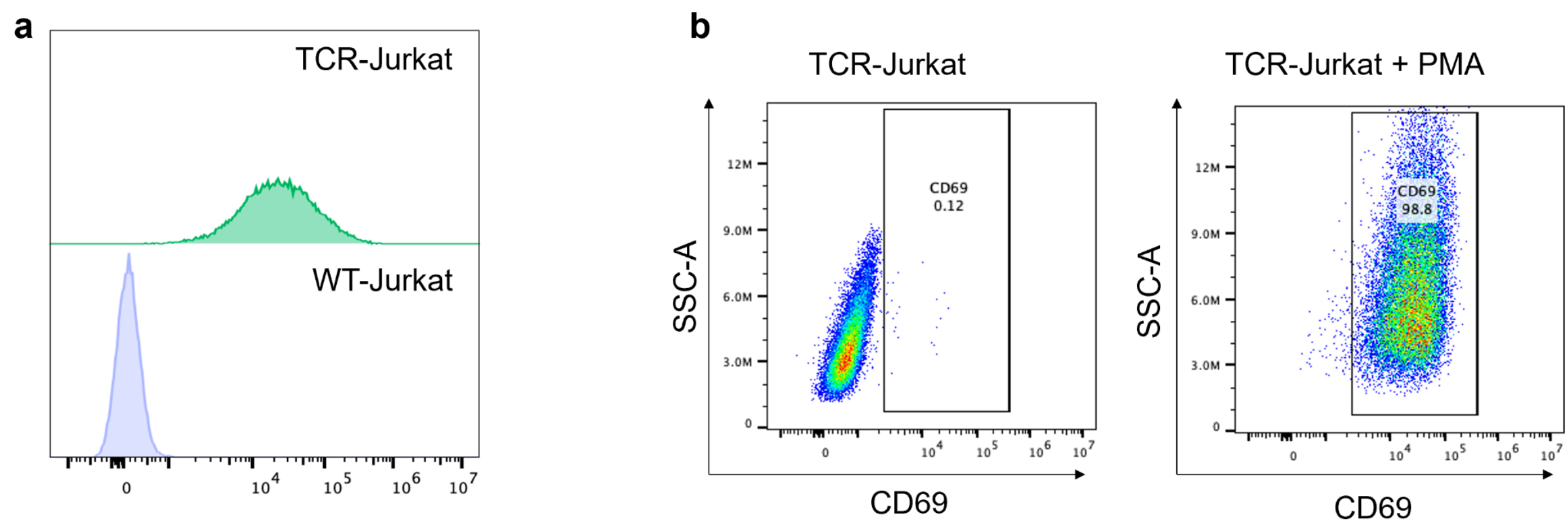

Figure S29. PMA/ionomycin positive control confirms the signaling competence of the engineered TCR-Jurkat reporter line. **a**, Flow cytometry histograms showing TCR expression in engineered TCR$^+$ CD8$^+$ Jurkat cells versus parental CD8$^+$ Jurkat cells, confirming successful TCR transduction. **b,** Representative flow cytometry plots of CD69 expression without stimulation (left) and after PMA/ionomycin stimulation (right). PMA/ionomycin, which bypasses proximal TCR engagement to directly activate downstream signaling, induced near-complete CD69 upregulation (98.8% CD69$^+$), showing that the reporter line is signaling-competent with a broad activation window.

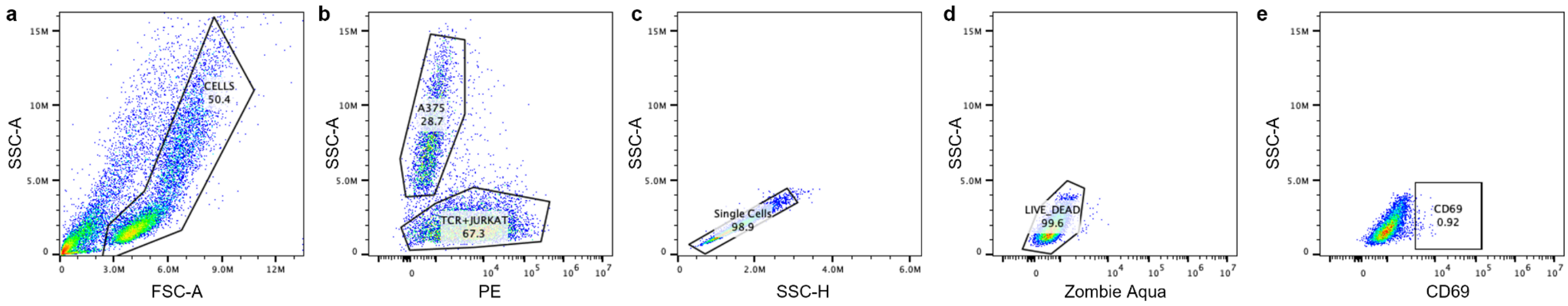

Figure S30. Gating strategy for flow-cytometric analysis of TCR+ Jurkat and A375 co-cultures. **a,** Debris was excluded on FSC-A versus SSC-A (Cells). **b,** A375 target cells and TCR+ Jurkat effector cells were distinguished on TCR (PE; TurboRFP) versus SSC-A. **c,** Doublets were excluded on SSC-H versus SSC-A (Single cells). **d,** Dead cells were excluded as Zombie Aqua-positive (Live). **e,** CD69+ frequency was quantified among viable TCR+ Jurkat cells on CD69 (BV421) versus SSC-A.

Supplementary Tables

Table S1. TCR information in the cross-reactivity prediction.

	alpha_j	beta_j	alpha_v	beta_v	CDR3α	CDR3β
TCR-C1	TRAJ37*01	TRBJ1-2*01	TRAV13-1*02	TRBV10-3*02	CAASLNTGKLIF	CAILGQGYGYTF
TCR-C2	TRAJ49	TRBJ1-2	TRAV24	TRBV6-5	CARNTGNQFYF	CASSPVTGGIYGYTF
TCR-C3	TRAJ15*01	TRBJ2-5*01	TRAV14N-1*01	TRBV17*01	CAASETSGSWQLIF	CASSRDLGRDTQYF
TCR-C4	TRAJ27*01	TRBJ2-2*01	TRAV12-2*01	TRBV30*01	CAVNDGGRLTF	CAWSETGLGMGGWQF
TCR-C5	TRAJ34*01	TRBJ2-7*01	TRAV19*01	TRBV6-1*01	CALSGFYNTDKLIF	CASPGLAGEYEQYF
TCR-C6	TRAJ24*01	TRBJ2-7*01	TRAV12-2*01	TRBV6-5*01	CAVTKYSWGKLQF	CASRPGWMAGGVELYF
TCR-C7	TRAJ57	TRBJ2-3	TRAV21	TRBV9	CAVSSPQGGSEKLVF	CASSVGLYSTDTQYF
TCR-C8	TRAJ32*01	TRBJ1-1*01	TRAV19*01	TRBV10-1*01	CALSEARGGATNKLIF	CASSRDTVNTEAFF
TCR-C9	TRAJ12*01	TRBJ2-4*01	TRAV12-3*01	TRBV12-4*01	CAMRGDSSYKLIF	CASSLWEKLAKNIQYF
TCR-C10	TRAJ48*01	TRBJ2-4*01	TRAV14D-1*01	TRBV13-3*01	CAALYGNEKITF	CASSDAGGRNTLYF
TCR-C11	TRAJ27*01	TRBJ2-2*01	TRAV12-2*01	TRBV30*02	CAVNVAGKSTF	CAWSETGLGTGELF
TCR-C12	TRAJ18*01	TRBJ2-1*01	TRAV19*01	TRBV11-3*01	CALSSRGSTLGRLYF	CASSPSGLAGSNLGNEQFF
TCR-C13	TRAJ45*01	TRBJ1-2*01	TRAV12-1*01	TRBV6-5*01	CVVALYSGGGADGLTF	CASRSQNYYGYTF
TCR-C14	TRAJ35*01	TRBJ2-4*01	TRAV9-4*01	TRBV13-2*01	CAVSGFASALTF	CASGGGGTLYF
TCR-C15	TRAJ13*01	TRBJ1-2*01	TRAV5*01	TRBV19*01	CAVSGGYQKVTF	CASTGSYGYTF
TCR-C16	TRAJ45*01	TRBJ2-1*01	TRAV12-1*01	TRBV19*01	CVVATYSGGGADGLTF	CASSIIAGSSYNEQF
TCR-C17	TRAJ58*01	TRBJ2-2*01	TRAV9D-3*01	TRBV13-1*01	CAVSAKGTGSKLSF	CASSDAPGQLYF
TCR-C18	TRAJ23*01	TRBJ2-2*01	TRAV38-1*01	TRBV10-2*01	CAYNQGGKLIF	CASSVLNTGELF
TCR-C19	TRAJ45*01	TRBJ2-2*01	TRAV12-1*01	TRBV10-3*01	CVVTEYSGGGADGLTF	CASRGQNTGELF
TCR-C20	TRAJ3*01	TRBJ1-1*01	TRAV12-1*01	TRBV6-1*01	CVVPLYSSASKIIF	CASSDTPDLNTEAF

Table S2. Screened antigen variants in the prospective study.

Antigen	Sequence	Sequence identity to raw
Raw sequence (MELAN-10)	EAAGIGILTV	-
AHV1	ELAGIAILTV	80%
AHV2	ELAGIGILAV	80%
AHV3	AAGIGILTVI	80%
AHV4	FLAGGIGIRTL	54.5%
AHV5	ALGGLLTMV	50%
AHV6	SMLGIGIVPV	50%
AHV7	LGIGVITI	40%
AHV8	SLWLGILSV	40%
ALV	SMAGIGIVDV	60%

Table S3. TCR sequence and HLA used in the prospective study.

	Sequence
TCRα	KEVEQNSGPLSVPEGAIASLNCTYSDRGSQSFFWYRQYSGKSPELIMSIYSNGDKED GRFTAQLNKASQYVSLLIRDSQPSDSATYLCAVQKLVFGTGTRLLVSPNIQNPDPAVY QLRDSKSSDKSVCLFTDFDSQTNVSQSKDSDVYITDKCVLDMRSMDFKSNSAVAWS NKSDFACANAFNNSIIPEDTF
TCRβ	NAGVTQTPKFQVLKTGQSMTLQCAQDMNHEYMSWYRQDPGMGLRLIHYSVGAGI TDQGEVPNGYNVSRSTTEDFPLRLLSAAPSQTSVYFCASSYSFTEATYEQYFGPGTR LTVTEDLKNVFPPEVAVFEPSEAEISHTQKATLVCLATGFYPDHVELSWWVNGKEVH SGVCTDPQPLKEQPALNDSRYALSSRLRVSATFWQDPRNHFRCQVQFYGLSENDEW TQDRAKPVTQIVSAEAWGRAD

Table S4. PBMC expansion and viability after antigen stimulation.

Group	Day 0 cell count	Day 7 cell count	Viability (%)	Expansion rate (%)
Group 1	3.74×10^5	9.50×10^5	91.94	153.88
Group 2	3.74×10^5	1.28×10^6	91.36	242.11
Group 3	3.74×10^5	6.44×10^5	87.61	72.06
Group 4	3.74×10^5	1.13×10^6	91.54	201.20
Group 5	3.74×10^5	7.92×10^5	93.84	111.76
Group 6	3.74×10^5	7.46×10^5	91.83	99.33
Group 7	3.74×10^5	8.90×10^5	92.06	137.83
Group 8	3.74×10^5	7.28×10^5	81.16	94.52
Negative control	3.74×10^5	5.67×10^5	88.8	51.60
Total	3.74×10^5	7.72×10^6	-	106.43

Supplementary References

1. Wang, N. *et al.* Deep peptide recognition profiling decodes TCR specificity and enables disease-associated antigen discovery. *Nat. Biotechnol.* https://doi.org/10.1038/s41587-026-03128-x (2026) doi:10.1038/s41587-026-03128-x.
