# Supplementary material for: UpTCR: a unified progressive knowledge transfer foundation model for robust T-cell receptor-antigen binding recognition
Source: Nat Commun. 2026 Sep 24;17:11086. doi: 10.1038/s41467-026-78075-x (PMC13696701; doi:10.1038/s41467-026-78075-x)
Supplement: Supplementary file 2 [file 41467_2026_78075_MOESM2_ESM.pdf]

nature portfolio

Corresponding author(s): Jianhua Yao, Kai Miao, Xiao Liu, Xiang Pan

Last updated by author(s): Aug 26, 2026

Reporting Summary

Nature Portfolio wishes to improve the reproducibility of the work that we publish. This form provides structure for consistency and transparency in reporting. For further information on Nature Portfolio policies, see our Editorial Policies and the Editorial Policy Checklist.

Statistics

For all statistical analyses, confirm that the following items are present in the figure legend, table legend, main text, or Methods section.

n/a	Confirmed	
[ ]	[x]	The exact sample size (n) for each experimental group/condition, given as a discrete number and unit of measurement
[ ]	[x]	A statement on whether measurements were taken from distinct samples or whether the same sample was measured repeatedly
[ ]	[x]	The statistical test(s) used AND whether they are one- or two-sided<br>*Only common tests should be described solely by name; describe more complex techniques in the Methods section.*
[x]	[ ]	A description of all covariates tested
[x]	[ ]	A description of any assumptions or corrections, such as tests of normality and adjustment for multiple comparisons
[ ]	[x]	A full description of the statistical parameters including central tendency (e.g. means) or other basic estimates (e.g. regression coefficient) AND variation (e.g. standard deviation) or associated estimates of uncertainty (e.g. confidence intervals)
[ ]	[x]	For null hypothesis testing, the test statistic (e.g. F, t, r) with confidence intervals, effect sizes, degrees of freedom and P value noted<br>*Give P values as exact values whenever suitable.*
[x]	[ ]	For Bayesian analysis, information on the choice of priors and Markov chain Monte Carlo settings
[x]	[ ]	For hierarchical and complex designs, identification of the appropriate level for tests and full reporting of outcomes
[ ]	[x]	Estimates of effect sizes (e.g. Cohen's d, Pearson's r), indicating how they were calculated

Our web collection on statistics for biologists contains articles on many of the points above.

Software and code

Policy information about availability of computer code

Data collection	Flow-cytometry data were acquired on a CytoFLEX S cytometer (Beckman Coulter). ELISA optical densities were recorded using the Multi Sciences human IL-2 kit (EK102). No custom data-collection software was developed.
Data analysis	Python (version not recorded), PyTorch 2.0.1, NumPy 1.24.3, SciPy 1.10.1, pandas 2.0.2, Transformers 4.30.2 and scikit-learn (version not recorded) were used for model training and computational analysis. GraphPad Prism 10 was used for Figure 8. MEGA11, WebLogo 3, PyMOL (version not recorded), UMAP (version not recorded) and AlphaFold 3 were used as described in the Methods. Custom code (MIT License): https://github.com/tylerlv/UpTCR, archived at https://doi.org/10.5281/zenodo.21979241.

For manuscripts utilizing custom algorithms or software that are central to the research but not yet described in published literature, software must be made available to editors and reviewers. We strongly encourage code deposition in a community repository (e.g. GitHub). See the Nature Portfolio guidelines for submitting code & software for further information.

Data

Policy information about availability of data

All manuscripts must include a data availability statement. This statement should provide the following information, where applicable:

- Accession codes, unique identifiers, or web links for publicly available datasets
- A description of any restrictions on data availability
- For clinical datasets or third party data, please ensure that the statement adheres to our policy

The model weights and processed data are available on Zenodo: https://zenodo.org/records/15128399. The curated datasets of TCR-antigen-HLA binding are derived from VDJdb44(https://vdjdb.cdr3.net/), PIRD47(https://db.cngb.org/pird/), McPAS-TCR48(https://friedmanlab.weizmann.ac.il/McPAS-TCR/), and IEDB49,50 (https://www.iedb.org/), and 10X Genomics51(https://www.10xgenomics.com/datasets) databases. The curated datasets of TCR-antigen-HLA complexes were downloaded from the STCRDab53 database (https://opig.stats.ox.ac.uk/webapps/stcrdab-stcrpred/Browser). The antigen-HLA binding affinity data were collected from the IEDB49,50 database (https://www.iedb.org/). The antigen-HLA independent test data were collected from weekly data52 between Oct. 2021 and Nov. 2024 with binding affinity measurements in the IEDB database (http://tools.iedb.org/auto_bench/mhci/weekly/).

Research involving human participants, their data, or biological material

Policy information about studies with human participants or human data. See also policy information about sex, gender (identity/presentation), and sexual orientation and race, ethnicity and racism.

Reporting on sex and gender	Sex and/or gender was not used as an inclusion criterion, was not stratified and was not analyzed as a covariate because the cohort comprises five adults and is too small for a powered sex-disaggregated analysis. How sex was determined was not recorded.
Reporting on race, ethnicity, or other socially relevant groupings	Age was not collected and was not analyzed as a covariate. The transfer evaluation used antigen-specific TCR-peptide pairs from five adults with clinically diagnosed breast cancer.
Population characteristics	Five adults with clinically diagnosed breast cancer (three Luminal subtype and two TNBC). Written informed consent was obtained. No additional compensation was provided.
Recruitment	Consecutive eligible patients at the participating clinical center were enrolled after written informed consent, and peripheral blood was collected. Enrollment was not based on predicted TCR repertoire. Any self-selection associated with consent to an academic study is noted; it is not expected to bias the computational transfer evaluation.
Ethics oversight	All experiments were approved by the ethics committee and institutional review board at Jiangnan University. Written informed consent was obtained from all participants. No protocol number was provided.

Note that full information on the approval of the study protocol must also be provided in the manuscript.

Field-specific reporting

Please select the one below that is the best fit for your research. If you are not sure, read the appropriate sections before making your selection.

[x] Life sciences [ ] Behavioural & social sciences [ ] Ecological, evolutionary & environmental sciences

For a reference copy of the document with all sections, see nature.com/documents/nr-reporting-summary-flat.pdf

Life sciences study design

All studies must disclose on these points even when the disclosure is negative.

Sample size	No formal power calculation was performed. Computational benchmarks used n = 5 independent training-and-evaluation runs or 5 cross-validation folds, a conventional size for reporting mean +/- s.d. of model performance. Prospective IL-2 and CD69 assays used n = 3 independent experimental batches. Cross-reactivity and structural analyses used all available TCR–antigen pairs or complex structures after the filters described in the Methods.
Data exclusions	No data were excluded from the reported analyses except by the pre-specified filters described in the Methods (sequence-identity de-redundancy, structure completeness, and AlphaFold pTM/ipTM quality filters).
Replication	Computational benchmarks were repeated across n = 5 independent runs or folds with a consistent ranking of methods. Cell-based assays were performed in n = 3 independent experimental batches with the same qualitative outcome. All attempts at replication were successful.

Randomization

Computational samples were assigned by the predefined majority, few-shot, zero-shot and random/novel-HLA splits described in the Methods, not by experimental randomization. In cell assays, parallel cultures from the same engineered reporter line were allocated to peptide or control conditions. Human participants were not randomized.

Blinding

Investigators were not blinded. Computational analyses used predefined splits and automated metrics. In the prospective cell assays, peptide identity was known because each condition required a distinct peptide stock; quantification used gates applied uniformly across conditions.

Reporting for specific materials, systems and methods

We require information from authors about some types of materials, experimental systems and methods used in many studies. Here, indicate whether each material, system or method listed is relevant to your study. If you are not sure if a list item applies to your research, read the appropriate section before selecting a response.

Materials & experimental systems

n/a	Involved in the study	
☐	☒	Antibodies
☐	☒	Eukaryotic cell lines
☒	☐	Palaeontology and archaeology
☒	☐	Animals and other organisms
☒	☐	Clinical data
☒	☐	Dual use research of concern
☒	☐	Plants

Methods

n/a	Involved in the study	
☒	☐	ChIP-seq
☐	☒	Flow cytometry
☒	☐	MRI-based neuroimaging

Antibodies

Antibodies used

PBMC sort (1:50): FITC anti-human CD3 clone OKT3 (BioLegend 317306); APC anti-human CD8a clone HIT8a (BioLegend 300912); PE anti-human CD137 clone 4B4-1 (BD Biosciences 555956); propidium iodide (BioLegend 421301). Jurkat assay (1:100): anti-CD8 clone RPA-T8, PE/Dazzle 594 (BioLegend 301058); anti-CD69 clone FN50, Brilliant Violet 421 (BioLegend 310930); Zombie Aqua Fixable Viability kit (BioLegend 423102). PMA/ionomycin, BioLegend 423304.

Validation

Commercial antibodies were used as validated by the manufacturers for flow cytometry. PBMC-sorting antibodies were used at 1:50; Jurkat-assay antibodies were used at 1:100, as stated in the Methods.

Eukaryotic cell lines

Policy information about cell lines and Sex and Gender in Research

Cell line source(s)

Jurkat, Clone E6-1 (ATCC, TIB-152), A375 (ATCC, CRL-1619) and 293FT (Invitrogen/Thermo Fisher Scientific, R70007) were used. Culture media and serum: RPMI 1640 ATCC modification (Gibco A10491-01), DMEM (Gibco C11885500BT) and FBS (Gibco 10270106).

Authentication

Cell lines were used as provided by the vendors. No additional STR profiling was performed in this study.

Mycoplasma contamination

Mycoplasma testing was not performed independently in this study.

Commonly misidentified lines
(See ICLAC register)

NA

Plants

Seed stocks	NA
Novel plant genotypes	NA
Authentication	NA

Flow Cytometry

Plots

Confirm that:

- [x] The axis labels state the marker and fluorochrome used (e.g. CD4-FITC).
- [x] The axis scales are clearly visible. Include numbers along axes only for bottom left plot of group (a 'group' is an analysis of identical markers).
- [x] All plots are contour plots with outliers or pseudocolor plots.
- [x] A numerical value for number of cells or percentage (with statistics) is provided.

Methodology

Sample preparation	After overnight peptide pulse (1 µg ml^{-1}) of HLA-A*02:01 A375 cells and 24 h co-culture (1:1, 1x10^5 cells each, 24-well plates), TCR+ CD8+ Jurkat cells were harvested and stained for 30 min at room temperature with Zombie Aqua (BioLegend 423102), anti-CD8 clone RPA-T8 (BioLegend 301058) and anti-CD69 clone FN50 (BioLegend 310930), all at 1:100.
Instrument	Data were acquired on a CytoFLEX S flow cytometer (Beckman Coulter) using CytExpert software (version not recorded).
Software	CytExpert (Beckman Coulter) for acquisition; analyses as described in the Methods and figure legends.
Cell population abundance	CD69+ frequency among viable CD8+ TCR+ Jurkat cells after 24 h stimulation (n = 3 independent experimental batches). Positive control: PMA/ionomycin (BioLegend 423304). Negative controls: DMSO (Coolaber) and unrelated peptide. IL-2: Multi Sciences EK102.
Gating strategy	Viable (Zombie Aqua-negative) CD8+ cells were gated and CD69+ frequency was quantified. Representative plots with tick labels and decade labeling on fluorescence axes are shown in Figure 8d and Supplementary Figures S26, S27 and S29, in the same format. The gating strategy is described in the Methods.

- [x] Tick this box to confirm that a figure exemplifying the gating strategy is provided in the Supplementary Information.
