# Supplementary material for: UpTCR: a unified progressive knowledge transfer foundation model for robust T-cell receptor-antigen binding recognition
Source: Nat Commun. 2026 Sep 24;17:11086. doi: 10.1038/s41467-026-78075-x (PMC13696701; doi:10.1038/s41467-026-78075-x)
Supplement: Supplementary file 3 [file 41467_2026_78075_MOESM3_ESM.pdf]

nature portfolio

Peer Review File

UpTCR: a unified progressive knowledge transfer foundation model for robust T-cell receptor-antigen binding recognition

Corresponding Author: Dr Xiang Pan

This file contains all reviewer reports in order by version, followed by all author rebuttals in order by version.

Version 0:

Reviewer comments:

Reviewer #1

(Remarks to the Author)
The authors present UpTCR, a unified progressive knowledge-transfer foundation model for TCRαβ–antigen–HLA binding prediction under incomplete data conditions. The model hierarchically transfers prior knowledge from dimeric and trimeric to tetrameric complexes and incorporates soft contrastive learning to mitigate noise arising from missing data. By integrating architectural priors, UpTCR aims to improve TCR specificity prediction, an hot topic in immunotherapy. Overall, UpTCR outperforms existing methods across the evaluated datasets and benchmarks.

While the proposed approach is promising and the results are encouraging, I have several concerns. Among others, the 3D structural analysis and the treatment of pairwise residue-level interactions are, in my opinion, weak and the discussion and comparisons would benefit from some additional information. Below, I outline my concerns in more detail.

Major Comments

1)
The claim that UpTCR facilitates the elucidation of pairwise residue-level contacts is not sufficiently supported. In Figure 6, I cannot clearly infer residue–residue interactions. The figure presents interactions across the full-length TCR, and while one can generally observe that CDR regions interact (e.g., via lighter colors in the contact maps shown in Figure 6b), it is not possible to identify which specific residues (amino acid description) and residue numbers are involved in these interactions. If the authors intend to claim residue-level interpretability, the manuscript would benefit from:
o Contact maps focused specifically on CDR regions, rather than the full TCR length, together with explicit annotation of residue indices and amino-acids identities.
o Clear examples illustrating which amino acids in the TCR interact with which amino acids in the antigen or HLA.

2)
Regarding the 3D structural analysis presented and the use of ipTM scores.
In Figure 7, violin plots show differences in ipTM scores between the high-confidence prediction group (HCPG) and the low-confidence prediction group (LCPG), with ipTM score being slightly higher for HCPG (around 0.49) than for LCPG (around 0.46). Considering that the ipTM score (as used in AlphaFold) is a confidence metric for interface quality where values below 0.6 often indicate transient or potentially incorrect binding, this raises important concerns. How can HCPG be described as having high-confidence interfaces when the ipTM values are below commonly accepted thresholds for reliable binding?
The authors should clarify:
o The rationale for defining “high confidence prediction group” in this context.
o Whether alternative interface-quality metrics were considered.

Additionally, Figures 7h and 7i would greatly benefit from a more informative structural presentation. Specifically: (1) Highlighting specific residue–residue interactions; (2) Indicating distances between interacting residues and (3) Clearly marking which CDRs are involved in antigen and MHC binding.
In their current form, these panels appear visually cluttered (“spaghetti-like”) and do not clearly show the secondary structure, CDRs and the residues. In particular, in the luminal subtype, some CDRs appear distant from the MHC and somewhat disordered.

3)
The residue-contact relationships are derived from 164 experimental structures. While valuable, this is a relatively limited structural dataset. Have the authors considered incorporating high-confidence structural models, such as model generated by AI, to expand coverage and improve the resolution of these interaction maps? This could substantially strengthen the structural component of the study.

4)
In the cross-reactivity analysis, the three antigen variants recognized by the same TCR appear to be highly similar in sequence. I wonder how does UpTCR perform when antigens differ by single-point mutations that disrupt binding? And if you have performed some analysis of the UpTCR performance when sequence-divergent peptides bind the same TCR? If available, results addressing these more challenging and relevant scenarios would be a plus.

5)
EPACT appears to be a strong competitor. For example, in Supplementary Figure S8a, the performance difference between EPACT and UpTCR is not statistically significant in the “majority setting”. Given this, it is unclear why, in the section “Translational validation of UpTCR in breast cancer across different subtypes”, the benchmark comparison is performed only against PanPep and not EPACT. The authors could justify this choice or include EPACT in the comparison.

6)

What is AUC when truncated at a false positive rate of 0.1 (i.e., AUC0.1)? This metric is particularly informative and a good complement to the analysis performed, as it emphasizes early retrieval performance, how well the model performs when only the most confident predictions are considered. Moreover, only a small number of prediction can be tested experimentally. Could the authors also provide AUC0.1 as a comparison metric in the benchmrak? AUC0.1 values per antigen in each set and for each tool would be a plus.

Minor Comments
The manuscript refers to Atchley factors and Blosum information as “biomedical properties” (Results, line 150, and Figure 1). These are more accurately described as bio-physicochemical rather than biomedical properties. The authors should clarify why the term “biomedical” is used here and in what sense these properties are considered biomedical.

(Remarks on code availability)

Reviewer #2

(Remarks to the Author)
A neural network method, UpTCR is developed to predict TCR-MHC-antigen complexes, and it also provides some mechanistic insights to TCR recognition. Pairwise information (TCR-antigen or antigen-MHC) is 5.6x more abundant than tertiary (TCR-Antigen-HLA) and the method conceptually is capitalizing on exploiting it.
The head-head comparisons of performance show an improvement over current state of the art. Most valuable results of the method when it can utilize generalizations to predict novelty (looking at: specialized zero shot training cases). However, several issues need to be addressed to clarify the actual performance. Also, it is not clear how much the method benefits from the integration of pairwise data, as claimed, or something else.

2. Pre-training conditions need more justification. Antigen and TCR sequences with 50% or higher identity were removed, but there are many functionally similar cases at lower sequence identity levels. e.g. cutoff for antigen sequence identity should/could be justified through the analysis of sequence variability among the same HLA binders.

3. This previous question is also prompted by the alarming difference in performance when comparing zero-shot and specialized zero shot results (going from 0.841 to 0.682 AUC) and thus it is unclear if more strict filtering conditions could further affect these results. And if significant results largely reserved for cases with reasonable templates in the training set.

4. The most convincing results are the head-to-head comparisons between different methods (irrespectively of the type of datasets), where (I assume) the same datasets were used for all NN training, pre-training and testing. This fact should be discussed clearly.

5. In case of TCR, the sequence identity comparison should focus on the functionally relevant and variable CDR regions, not the full protein.

6. Discussion is missing about the unusual results that the few-shot testing is (although not much but) inferior to Zero-Shot training (0.824 vs 0.841).

7. Interesting that (Fig 3D) pretraining with TCR or HLA only (i.e. using pairwise complexes), results in statistically

indistinguishable performance from the full pretraining approach. This might suggest that most of the advance is coming from the actual technical details (or some other factors?) of the method and NOT from the conceptual advance of including TCR-antigen-HLA training simultaneously. This should be discussed and further explored (and it is also in contrast to lines 673-678 where it is argued to be a “distinctive advantage")

8. Similarly, Fig 3A, when various modularities are ablated, it seems that some/most(?) modules can be removed without affecting performance (zero shot cases). It is not clear because for some reason these comparisons are not shown (i.e. the statistical significance of difference between UpTCR and the rest of various ablated UpTCR is not shown, just the significance of differences are shown among the rest).Discussed in (313-345)

9. Figure 5 and corresponding discussion is not very insightful. Would be more useful if the # of or % of false positives were also presented. It is unclear even in case of "high predicted binding probability" how many more false positive were also predicted. A particular useful analysis would mix true positives with false positive antigens using the same “Levenshtein Distance”.

10. The perspective validation of predicted peptides for MELAN-10: details are not provided. How were the variants generated? How many? And was MELAN-10 part of the original training of the TCR-ag-HLA system or any similar peptides? This is because among all the enumerated peptides for MELAN-10: (table S2), the 3 high affinity peptides identified have 9/10, 9/10 and 9/9 identical matches to MELAN-10, while the low affinity one has 6/9 identical residues. A simple sequence comparison would end up with similar conclusion. However, it would be impressive if MELAN -10 or similar peptides were not part of the training.
In addition, the MELAN-10 peptide should be included in tables S2. I had to look it up from the literature to do the above assessment of sequences.

11. Fig 8 panel 3, was there any repeats of flow cytometry ? The legends states “representative”. If more were done the average and std can be provided with p- values. The differences appear to be small by the single one example provided.

12. Contact map prediction Fig 6. Some of the contacts are very conserved especially with HLA-Antigen, and in can case of TCR -antigen it is also similar. Therefore the quality of contact map prediction needs to be put in some context, compared to some other references. Otherwise it is very difficult to make a quantitative sense of these spectrum maps .

Minor
13. Repetition (repeated paragraphs) of the same arguments, same references: lines 70-77 and 78-84
Repetition of testing concepts: 244-247 and 258-260

14. The Results section provides excessive qualitative discussion about the trained system (incl. Fig 1), which could be drastically reduced. Qualitative discussions should be replaced with the actual performance numbers e.g. (lines 267-275 and 276-280 320-345 etc. “significantly outperformed” “best performing” “ significantly improved” "certain degree of performance decline” "most significant impact on performance” (?) Meanwhile it is mentioned 7 times that a p-value significance was used at 0.05 cutoff, which is understood but unnecessarily cited so many times.

(Remarks on code availability)
1. The Github deposit needs better organization:
It took more than 6 hours for data download at Gbit speed connection.

2. The program requires an outdated operating linux system (CENTOS 8 years 2020-21) and outdated CUDA environment (CUDA 11), which most users will not have access to anymore.

3. After we located a backdated linux and CUDA system we downloaded the package.It is not working as a critical file is missing:

"The file model_weights.zip cannot be found on Zenodo: https://doi.org/10.5281/zenodo.15128399"

Reviewer #3

(Remarks to the Author)
The authors propose soft contrastive pretraining and multi-scale modelling strategies to predict TCR-peptide-HLA interactions from integrated TCR-peptide-HLA datasets with various missing data. Then, the authors show that this approach can improve the predictions compared to the previously published methods. Finally, the authors show wet lab validations on their TCR prediction algorithm.

Major Comments

1. The authors report that UpTCR predictions align well with a curated cross-reactivity dataset. However, these datasets have assay-dependent definitions of reactivity (e.g., T cell lines vs. primary T cells). To strengthen this claim, the authors should independently validate the predicted cross-reactive peptides for a subset of TCRs.

2. It would be informative to assess whether the low-conservation peptides retain measurable binding affinity to their HLA

molecules.

3. Inclusion of non-HLA-A2-restricted TCRs would substantially enhance the generalizability of the approach, especially as the model is described as pan-specific.

4. In Figure 6, the interaction conformation prediction module was trained on only 164 crystal structures. Given the high dimensionality of residue-level contact maps, this dataset size may be insufficient to avoid overfitting. The authors should provide a validation using a strictly held-out structural dataset not used during any part of the pretraining or finetuning phases.

5. In Figure 7, T cells were stimulated with peptide pools on days 3 and 7 of a 7-day culture. Repeated stimulation within a short window can induce activation-induced cell death (AICD) or promote dysfunctional expansion in primary CD8+ T cells. The authors should justify why this specific regimen was chosen and how it might impact the reliability of the resulting TCR dataset.

6. The "unique address" peptide matrix logic for identifying antigen-specific TCRs should be more clearly explained to ensure that potential overlapping specificities within the eight peptide pools did not introduce false positives in the Luminal or TNBC cohorts.

7. Figure 8 experiment would benefit from well-characterized literature-based positive and negative control TCRs to benchmark against the Melan-A-specific TCR used. The variants tested (e.g., Antigen HV1-3) closely resemble the canonical MART1 epitope. Given the claim that the model operates in zero-shot settings, experimental evaluation using variant peptides with significantly lower sequence identity would provide a more compelling demonstration of model robustness.

8. For the specificity task, negative samples were generated via a 1:5 ratio of positive to shuffled negative pairs. While the authors argue that background sampling introduces bias, random shuffling can create "biologically impossible" negatives that the model may find too easy to distinguish. The inclusion of "hard negatives"—such as non-binding homologous peptides or TCRs—would provide a more realistic assessment of the model's discriminative boundary.

9. The authors demonstrate that pretraining helps, but it is unclear if the specific "triplet" stage (Stage II) provides a significant marginal gain over just the "dimer" stage (Stage I) for all tasks. A clearer ablation study isolating the performance increase attributed solely to the transition from Stage I to Stage II pretraining would clarify the necessity of this multi-scale approach.

Minor Comments
1. In Table S1, the column header "alpha_v" is duplicated; the second instance should be corrected to "beta_v" to correctly identify the V-gene usage for the beta chain.

2. To ensure reproducibility, the table should include the full CDR3 sequences (both alpha and beta chains) and the specific predicted peptide targets for all 20 reported cross-reactive TCR groups. Currently, only J and V gene identifiers are provided.

(Remarks on code availability)

Reviewer #4

(Remarks to the Author)
I co-reviewed this manuscript with one of the reviewers who provided the listed reports. This is part of the Nature Communications initiative to facilitate training in peer review and to provide appropriate recognition for Early Career Researchers who co-review manuscripts.

(Remarks on code availability)

Version 1:

Reviewer comments:

Reviewer #1

(Remarks to the Author)
The manuscript has improved and my comments have been addressed. I consider the current version suitable for publication.

(Remarks on code availability)

Reviewer #2

(Remarks to the Author)
The authors did an excellent job responding to my questions.

I would like to note that, while the response to my Comment 2 is perfectly acceptable, the interpretation of the results could be made somewhat more cautious. Qualitatively, an AUC value of 0.59 indicates very limited predictive performance for practical purposes. It is factually correct that this is better than the next best method, which has an AUC of approximately 0.52 and is essentially close to random. However, in cases where no suitable training data are available to support inference by the ML approach (i.e., zero-shot training at a 30% identity cutoff between the training and testing sets) an AUC of 0.59 suggests only limited biologically useful information. Thus, there remains considerable scope for future work.

I leave this comment for the authors to consider. Overall, the revision was excellent. Congratulations.

(Remarks on code availability)

Reviewer #3

(Remarks to the Author)
The new computational analyses, together with the expanded functional validation, resolve majority of the concerns raised in the first round, and the manuscript is considerably stronger. However, two issues remain outstanding, which should be settled before publication because they bear on whether the functional and cross-reactivity claims can be independently interpreted and verified.

1. The prospective validation in Figure 8 reads out TCR engagement as CD69 upregulation (and IL-2 secretion) in an engineered Jurkat line, and the revision helpfully adds a literature-derived positive-control TCR (MEL8) confirming that the cognate Melan-A peptide drives a response. However, the assay still lacks a receptor-independent positive control establishing that the Jurkat line itself is signaling-competent across the full panel. Jurkat cells are known to have a relatively narrow activation window in CD69-based readouts, and without such a control it is difficult to interpret the weaker or negative responses - in particular for the low-identity variants (AHV4-AHV8) and the predicted low-binder - as genuine absence of TCR recognition rather than limited assay sensitivity.

Please include a PMA/ionomycin (receptor-bypassing) positive control alongside the peptide stimulations to demonstrate maximal signaling capacity of the line, run within the same experiments as Figure 8b-d. Confirming the key discriminations in a more responsive reporter system will strengthen the conclusion that the variant-specific differences reflect true differences in TCR recognition.

2. The cross-reactivity analysis (Figures 3f, 4a and 5; TCR-C1 to TCR-C20) is one of the manuscript's central interpretability claims, yet the materials needed to verify it are incomplete. The revised Table S1 lists only the V/J genes and CDR3α/CDR3β sequences for each TCR group, and the corresponding source-data sheet contains only a TCR identifier and a peptide column. Neither carries the HLA restriction of each TCR group or the originating (reported) epitope. This omission matters: a set of peptides can be interpreted as genuinely cross-reactive for a given TCR only if they are co-presented by the same restricting allele, and without the per-group HLA there is no way to confirm that the listed peptides share a restriction element rather than reflecting heterogeneous restrictions pooled together. Relatedly, the high- and low-conservation peptides are not labeled as to provenance, so it is not possible to tell which sequences are naturally occurring, gene-derived epitopes and which are synthetic, model-proposed variants - a distinction that bears directly on how the low-conservation results should be read.

Please annotate each cross-reactive TCR group (C1-C20) with its HLA restriction and its reported/source epitope (for example, GP33-41 / H2-Db for TCR-C10) and flag every peptide in the high- and low-conservation sets as synthetic versus gene-derived. Including the per-group co-presentation evidence (e.g., the NetMHCpan binding check already performed for the low-conservation peptides) in the same table would let a reader confirm shared restriction at a glance.

Co-Reviewer Comments

Many of the comments have been addressed, but there still remains few concerns.

For comment 3 (Figure 3f, Figure 4a, Figure 5 cross-reactivity analysis of TCR-C1 to C20), please include the MHC restrictions of TCR-C1–C20 along with their reported epitopes (for example, GP33–41/H2-Db for TCR C10). Please also label the synthetic vs gene-derived high/low-conservation peptides, as I was unable to locate them in the source file or supplementary tables, though I may have missed them.

Moreover, if feasible, it would be interesting to assess how UpTCR performs on recently published datasets such as the autoantigen/HLA-B27 restricted TCRs reported by Wang et al (2026 Nature Biotechnology). Even a brief analysis or discussion of these cases could help contextualize the generalizability across newly characterized antigen-TCR pairs.

Wang, N., Yeh, H., Lai, B., Perera, J., Jude, K. M., Risch, I., ... & Garcia, K. C. (2026). Deep peptide recognition profiling decodes TCR specificity and enables disease-associated antigen discovery. Nature Biotechnology, 1-11.

For comment 7 (Figure 8), it would be helpful to consider a more responsive T cell line. Inclusion of PMA/ionomycin positive control could show signaling competence in the T cell line. In its current setup, the Jurkat line used may have limited sensitivity to distinguish between cognate and non-cognate peptide in a CD69 upregulation readout.

(Remarks on code availability)

Reviewer #4

(Remarks to the Author)
I co-reviewed this manuscript with one of the reviewers who provided the listed reports. This is part of the Nature Communications initiative to facilitate training in peer review and to provide appropriate recognition for Early Career Researchers who co-review manuscripts.

(Remarks on code availability)

Version 2:

Reviewer comments:

Reviewer #3

(Remarks to the Author)
The revised manuscript appears to have adequately addressed this reviewer's comments. Thank you for the opportunity to review this manuscript.

(Remarks on code availability)

Reviewer #4

(Remarks to the Author)
I co-reviewed this manuscript with one of the reviewers who provided the listed reports. This is part of the Nature Communications initiative to facilitate training in peer review and to provide appropriate recognition for Early Career Researchers who co-review manuscripts.

(Remarks on code availability)

Open Access This Peer Review File is licensed under a Creative Commons Attribution 4.0 International License, which permits use, sharing, adaptation, distribution and reproduction in any medium or format, as long as you give appropriate credit to the original author(s) and the source, provide a link to the Creative Commons license, and indicate if changes were made.
In cases where reviewers are anonymous, credit should be given to 'Anonymous Referee' and the source.
The images or other third party material in this Peer Review File are included in the article's Creative Commons license, unless indicated otherwise in a credit line to the material. If material is not included in the article's Creative Commons license and your intended use is not permitted by statutory regulation or exceeds the permitted use, you will need to obtain permission directly from the copyright holder.
To view a copy of this license, visit https://creativecommons.org/licenses/by/4.0/

Point-by-point response

Manuscript title: "UpTCR: a unified progressive knowledge transfer foundation model for robust T-cell receptor-antigen binding recognition"

We sincerely thank all reviewers for their valuable feedback that we have used to improve the quality of our manuscript. Here we include a point-by-point response, where *reviewers' comments are presented in italics*, our responses are in blue, and changes made to the manuscript are shown in red (*figure captions are in italics*).

The revised manuscript includes substantial new computational benchmarking, expanded wet-lab experimental validations, refinements to the main figures (Figures 2, 3, 5, 6, 7, 8) and Supplementary Figures and Tables, ten new Supplementary Figures, and one new Supplementary Table to address the reviewers' comments. Furthermore, more detailed method description, result analysis and discussion have been incorporated to enhance the rigor and clarity of this work.

Reviewer #1 (Remarks to the Author):

Summary

The authors present UpTCR, a unified progressive knowledge-transfer foundation model for TCRαβ–antigen–HLA binding prediction under incomplete data conditions. The model hierarchically transfers prior knowledge from dimeric and trimeric to tetrameric complexes and incorporates soft contrastive learning to mitigate noise arising from missing data. By integrating architectural priors, UpTCR aims to improve TCR specificity prediction, a hot topic in immunotherapy. Overall, UpTCR outperforms existing methods across the evaluated datasets and benchmarks.

While the proposed approach is promising and the results are encouraging, I have several concerns. Among others, the 3D structural analysis and the treatment of pairwise residue-level interactions are, in my opinion, weak and the discussion and comparisons would benefit from some additional information. Below, I outline my concerns in more detail.

Response: We sincerely thank Reviewer #1 for the constructive and detailed evaluation. We appreciate the reviewer's emphasis on strengthening the 3D structural analysis and residue-level interaction interpretability. In the revised manuscript, we have substantially improved both aspects, including zoomed-in CDR-level contact maps with residue annotations (Comment 1), redesigned structural visualizations with distance measurements (Comment 2), and AlphaFold-augmented training data (Comment 3). The detailed responses to each comment are provided below.

Comment 1)

The claim that UpTCR facilitates the elucidation of pairwise residue-level contacts is not sufficiently supported. In Figure 6, I cannot clearly infer residue–residue interactions. The figure presents interactions across the full-length TCR, and while one can generally observe that CDR regions interact (e.g., via lighter colors in the contact maps shown in Figure 6b), it is not possible to identify which specific residues (amino acid description) and residue numbers are involved in these interactions.

If the authors intend to claim residue-level interpretability, the manuscript would benefit from:

- *Contact maps focused specifically on CDR regions, rather than the full TCR length, together with explicit annotation of residue indices and amino-acids identities.*

- *Clear examples illustrating which amino acids in the TCR interact with which amino acids in the antigen or HLA.*

Response: We thank the reviewer for this constructive suggestion regarding the visualization of residue-level contacts. We agree that the original Figure 6 only presented contact maps across the full-length TCR, making it difficult to identify specific interacting residue pairs. In the revised manuscript, we have substantially improved the structural interpretability of Figure 6. Specifically:

(1) We now provide zoomed-in contact maps focused on CDR regions with explicit annotation of amino acid identities and residue positions. For a representative TCR-antigen-HLA complex (PDB ID: 6R2L), we provided detailed CDR3β-antigen contact maps (revised Figure 6d) and HLA-antigen contact maps (revised Figure 6e), each annotated with one-letter amino acid codes and residue positions on both axes. These maps enable direct identification of specific interacting residue pairs. For example, residues S99, I100, S101, and S102 of TCRβ exhibit strong contacts with the antigen peptide, consistent with their spatial proximity observed in the crystal structure.

(2) For both zoomed-in panels, we present direct comparisons of our predictions (top) and experimentally determined ground truth (bottom), demonstrating that the predicted contact patterns closely recapitulate the crystallographically observed interactions.

These revisions are described in the updated Results section and the revised Figure 6 legend. To be clearer, the detailed revised content is presented below.

Result (page 25):

We further presented a representative case (PDB ID: 6R2L) to exemplify UpTCR's ability in elucidating interactions within the TCR-antigen-HLA complex (Figure 6c). The antigen peptide first binds to the HLA molecule to form an antigen-HLA complex and is then presented on the cell surface. Subsequently, T cells recognize the HLA-antigen complex through their surface TCRs, with CDR3 playing a dominant role in the specific recognition of the antigen peptide, while CDR1, CDR2, and CDR2.5 primarily interact with the α-helical domains of the HLA molecule. Our results demonstrated that UpTCR can effectively predict the contact maps across all six pairwise interactions, including TCRα-TCRβ, TCRα-HLA, TCRβ-HLA, TCRα-antigen, TCRβ-antigen, and antigen-HLA (Supplementary Figure S12-S17). To illustrate the residue-level specificity captured by UpTCR, we generated zoomed-in contact maps for CDR3β-antigen and HLA-antigen interactions with amino acid identities and residue indices (Figure 6d,e). In the CDR3β-antigen contact map, residues S99, I100, S101, and S102 of TCRβ exhibited strong predicted contacts with the antigen peptide, consistent with their spatial proximity to the bound

peptide in the crystal structure. In the HLA-antigen contact map, each antigen residue displayed a distinct contact profile with specific HLA residues lining the binding groove, reflecting the position-dependent anchoring of the peptide within the HLA binding cleft. Notably, the predicted contact patterns aligned with the experimentally determined ground truth, correctly identifying key interacting residue pairs across both interfaces.

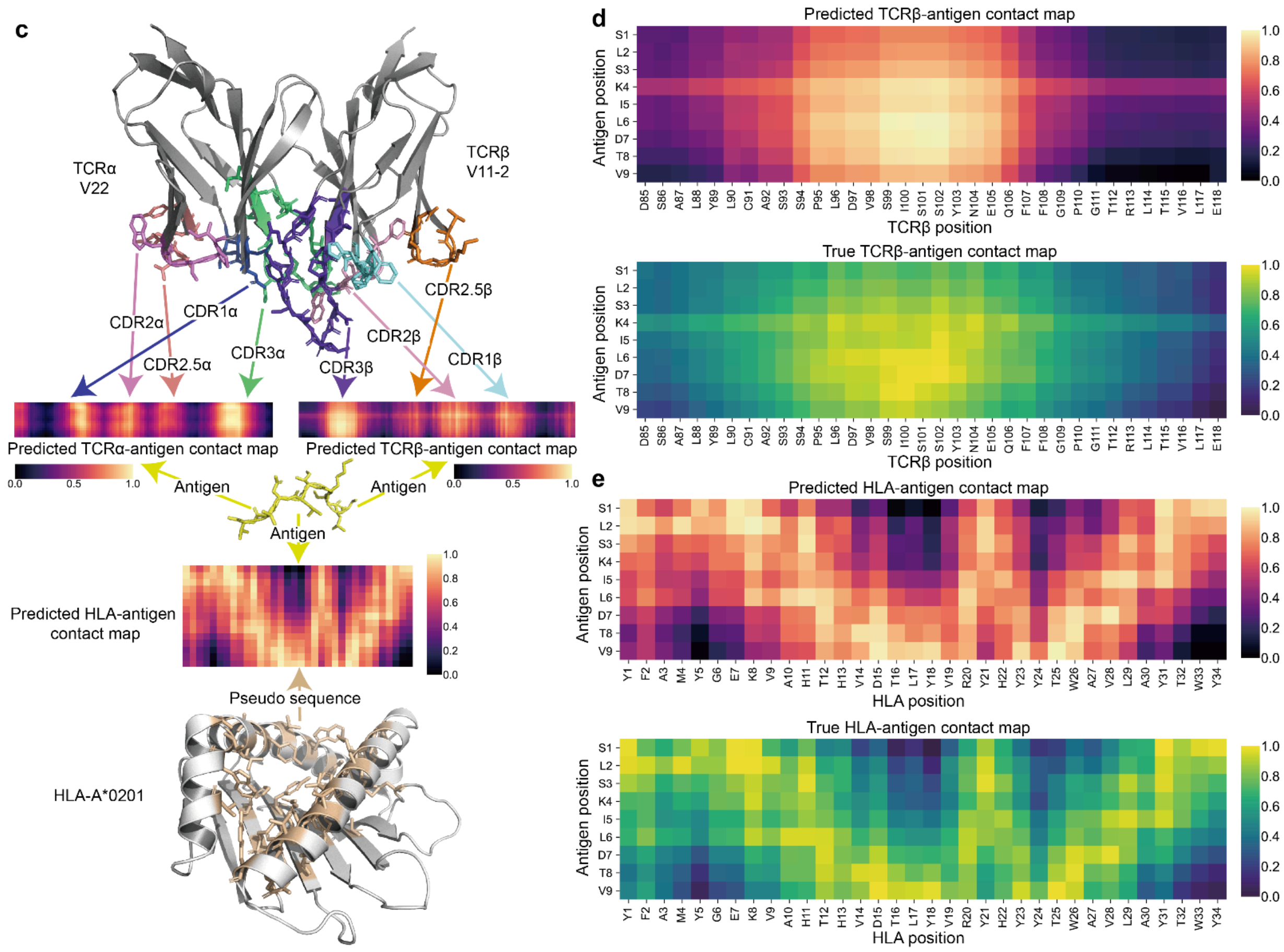

Figure 6. ***…*** ***d,*** *Zoomed-in residue-level contact maps with amino acid identities and residue indices for CDR3β-antigen interactions, comparing UpTCR predictions (top) with experimentally determined ground truth (bottom).* ***e,*** *Zoomed-in residue-level contact maps with amino acid identities and residue indices for HLA-antigen interactions comparing UpTCR predictions (top) with experimentally determined ground truth (bottom).*

Comment 2)

Regarding the 3D structural analysis presented and the use of ipTM scores. In Figure 7, violin plots show differences in ipTM scores between the high-confidence prediction group (HCPG) and the low-confidence prediction group (LCPG), with ipTM score being slightly higher for HCPG (around 0.49) than for LCPG (around 0.46). Considering that the ipTM score (as used in AlphaFold) is a confidence metric for interface quality where values below 0.6 often indicate transient or potentially

incorrect binding, this raises important concerns. How can HCPG be described as having high-confidence interfaces when the ipTM values are below commonly accepted thresholds for reliable binding?

The authors should clarify:

- *The rationale for defining "high confidence prediction group" in this context.*
- *Whether alternative interface-quality metrics were considered.*

Additionally, Figures 7h and 7i would greatly benefit from a more informative structural presentation. Specifically: (1) Highlighting specific residue–residue interactions; (2) Indicating distances between interacting residues and (3) Clearly marking which CDRs are involved in antigen and MHC binding. In their current form, these panels appear visually cluttered ("spaghetti-like") and do not clearly show the secondary structure, CDRs and the residues. In particular, in the luminal subtype, some CDRs appear distant from the MHC and somewhat disordered.

Response: We thank the reviewer for this rigorous assessment of the structural analysis in Figure 7. We fully acknowledge the concerns regarding the ipTM scores and the quality of the visualizations, and have made substantial revisions to address both issues.

1) Regarding the ipTM scores and HCPG/LCPG terminology:

- We appreciate the reviewer's critical observation. Upon investigation, we identified that the low ipTM values reported in the original manuscript were primarily attributable to the use of full-length MHC sequences (365 residues) as input for AlphaFold3. The membrane-proximal α3 domain and transmembrane regions led to low prediction confidence.
- We recognize that accurate structural prediction of TCR-pMHC complexes remains an open challenge for current AI-based tools, as the inherent flexibility and diversity of CDR loops limit prediction accuracy. Given these limitations, we have removed the group-level statistical comparison between the high-confidence prediction groups (HCPG) and low-confidence prediction groups (LCPG) from the revised manuscript (original Figure 7f,g) to ensure scientific rigor and avoid over-interpretation of computationally modeled interfaces.
- We now provide one representative AlphaFold3-predicted TCR–antigen–HLA complex structure for each breast cancer subtype, selected based on high structural confidence (ipTM > 0.8). The structural visualizations have been completely redesigned following the reviewer's recommendations as below.

2) Regarding the structural visualization:

- We have completely redesigned the structural panels (revised Figure 7f–k) following the reviewer's recommendations. The revised panels now feature: (1) cartoon representation of TCR molecules with clearly annotated CDR regions (CDR1, CDR2, CDR3) ; (2) zoomed-in views of key molecular interfaces showing CDR3β-antigen contacts and CDR1/CDR2β-HLA contacts separately; (3) specific interacting residues labeled by amino acid identity and residue number; and (4) inter-atomic distances indicated in Ångströms with hydrogen bonds highlighted by yellow dashed lines. For the Luminal subtype, residues S94, F96, and S100 of TCRβ form contacts with antigen residues L2, M4, and W5 ranging from 3.1 Å to 4.2 Å, while R30 and T53 of TCRβ engage HLA residue T78. For the TNBC subtype, CDR3β residue T98 contacts antigen residue P4, and CDR2β residues N52 and V54 form a hydrogen bond with HLA residue Q96 at 2.7 Å.

These revisions are reflected in the updated Results section and revised Figure 7. To be clearer, the revised content is presented below.

Results (page 28):

To provide structural insights into the predicted TCR-antigen-HLA interactions, we employed AlphaFold3 to model representative high-confidence TCR-antigen-HLA complex structures. For the Luminal subtype (Figure 7f), the predicted structure revealed a canonical TCR binding orientation, with CDR3β positioned centrally over the antigen peptide and CDR1β/CDR2β engaging the HLA α-helices. Zoomed-in views of the CDR3β-antigen interface (Figure 7g) identified specific interacting residue pairs, including contacts between S94, E96, and S100 of TCRβ and residues L2, M4, and W5 of the antigen peptide, with inter-atomic distances ranging from 3.1 Å to 4.2 Å. At the CDR1β/CDR2β-HLA interface (Figure 7h), residues R30 and T53 of TCRβ formed hydrogen bonds with A173 and E178 of the HLA. For the TNBC subtype (Figure 7i–k), a similar pattern was observed, with CDR3β residue T98 contacting antigen residue P4 at a distance of 2.7 Å (Figure 7j), and CDR2β residues N52 and V54 forming hydrogen bonds with HLA residue R99 and Q96 (Figure 7k). These observations demonstrate that UpTCR's high-confidence predictions correspond to biologically plausible binding conformations with molecular contacts.

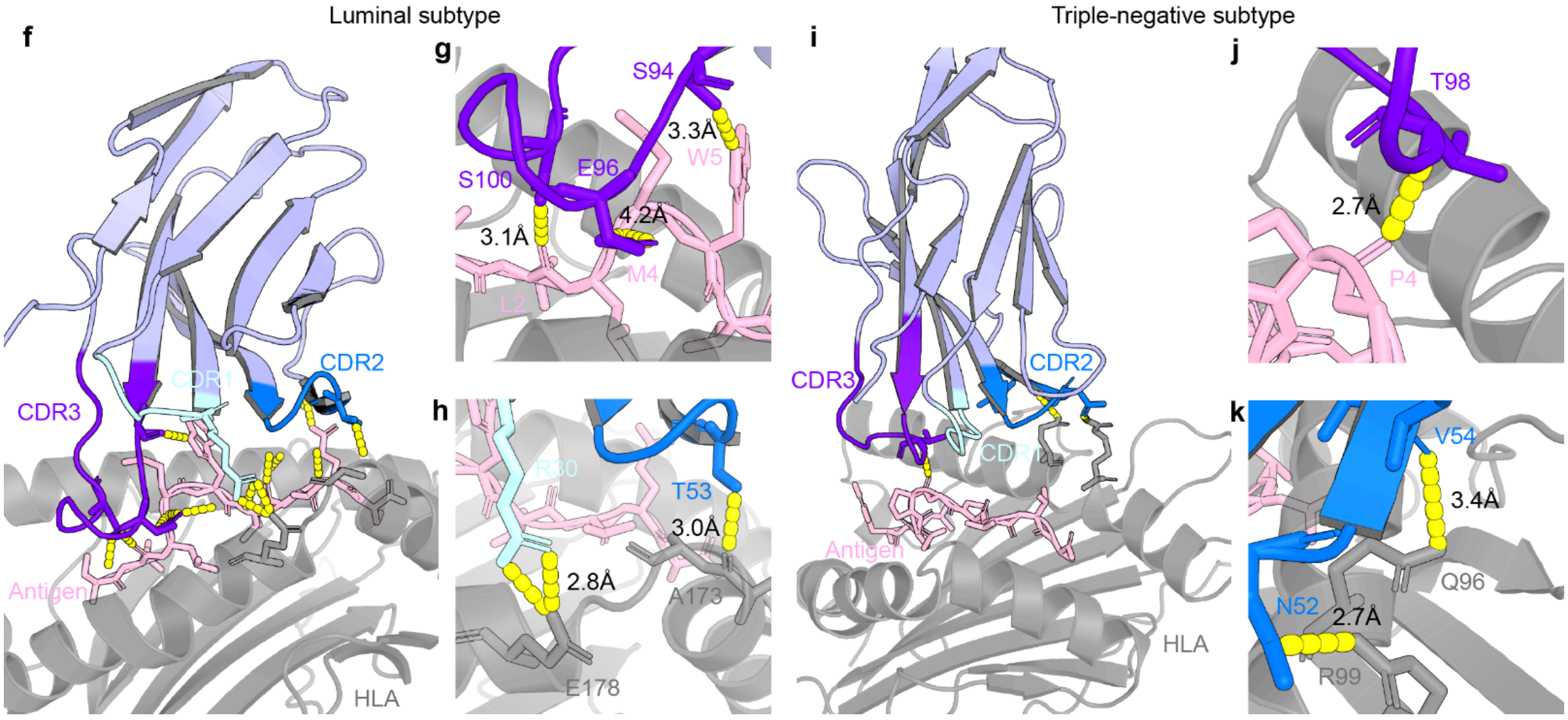

Figure 7. … f-h, *Representative AlphaFold3-predicted TCR–antigen–HLA complex structures for the Luminal subtype. The left panel **(f)** displayed the overall architecture with annotated CDR regions (CDR1, CDR2, CDR3) of the TCRβ chain (ipTM: 0.8, pTM: 0.82). The HLA molecule is shown in gray, the antigen peptide in pink (stick), and the TCR in purple/blue (cartoon). Right panels **(g-h)** present zoomed-in views of key molecular interfaces: CDR3β–antigen contacts **(g)** and CDR1/CDR2β–HLA contacts **(h)**, with specific interacting residues labeled by amino acid identity, residue number, and distance. Hydrogen bonds are highlighted by yellow dashed lines. **i-k,** Representative AlphaFold3-predicted TCR-antigen-HLA complex structure for the TNBC subtype, presented in the same format as **f–h** (ipTM: 0.89, pTM: 0.87). The overall complex architecture **(i)** is shown with CDR annotations, accompanied by zoomed-in views of CDR3β-antigen contacts **(j)** and CDR2β–HLA contacts **(k)**, with interacting residues and distances annotated as described above.*

Comment 3)

The residue-contact relationships are derived from 164 experimental structures. While valuable, this is a relatively limited structural dataset. Have the authors considered incorporating high-confidence structural models, such as model generated by AI, to expand coverage and improve the resolution of these interaction maps? This could substantially strengthen the structural component of the study.

Response: We thank the reviewer for this valuable suggestion. We agree that 164 experimental crystal structures represent a limited structural dataset, and that incorporating AI-predicted structures is a promising strategy to expand coverage.

In the revised manuscript, we have implemented this recommendation by employing AlphaFold3 to predict TCR-antigen-HLA complex structures for tetrameric sequences in our partial curated dataset, yielding a total of 2,652 predicted structures. To ensure structural reliability, we applied stringent quality filters based on predicted Template Modeling (pTM > 0.8) and interface predicted Template Modeling (ipTM > 0.8) scores, retaining 1,037 high-confidence structures. Using this augmented dataset, we trained an enhanced variant, UpTCR+AF, which combines the 164 experimentally determined crystal structures with the 1,037 AlphaFold-predicted structures.

We evaluated UpTCR+AF against both UpTCR (trained on crystal structures only) and TEIM-Res across all six pairwise interaction types. UpTCR+AF further improved upon UpTCR in most interaction types, demonstrating that augmenting the limited crystal structure data with high-confidence computational models meaningfully enhances the model's ability to predict residue-level contacts.

The description of the AlphaFold-augmented structural data has been added to the Methods section, and the revised content has been added to the revised Results section, Figure 6, and corresponding Supplementary Figures.

To be clearer, the revised content is presented below.

Results (page 24):

…This matrix is then processed by a convolutional neural network to predict the continuous spatial distance between residue pairs. We finetuned and cross-validated UpTCR using 164 experimentally determined TCR-antigen-HLA complex structures as ground truth. To further expand the structural training data, we constructed an augmented variant, UpTCR+AF, by incorporating 1,037 high-confidence AlphaFold-predicted TCR-antigen-HLA complex structures filtered by stringent predicted Template Modeling (pTM) and interface predicted Template Modeling (ipTM) thresholds (pTM > 0.8 and ipTM > 0.8, Supplementary Figure S21).

…

We evaluated the contact prediction performance using both binary classification (AUC) and continuous distance regression (Pearson correlation) metrics. In cross-validation on the 164 crystal structures, UpTCR significantly outperformed TEIM-Res[1] across all six pairwise interaction types in both AUC ($P < 0.05$, Figure 6a) and Pearson correlation ($P < 0.05$, Supplementary Figure S22). UpTCR+AF further improved upon UpTCR in most interaction types, demonstrating the benefit of augmenting the limited crystal structure data with high-confidence computational models. Notably, the improvement was most pronounced for TCRα-TCRβ contact map predictions (Pearson:

TEIM-Res 0.705 versus UpTCR 0.806 versus UpTCR+AF 0.826), reflecting the capacity of additional structural data to capture the complex chain-pairing interface.

Methods (page 40):

AlphaFold-augmented structural data. To expand the structural training data beyond the 164 experimentally determined crystal structures, we employed AlphaFold3 to predict TCR-antigen-HLA complex structures for all available tetrameric sequences in our curated dataset. A total of 2,652 structures were generated. To ensure structural reliability, we applied stringent quality filters based on the predicted Template Modeling (pTM) and interface predicted Template Modeling (ipTM) scores, retaining 1,037 high-confidence structures (Supplementary Figure S21). The augmented variant (UpTCR+AF) was trained using the combined dataset of 164 crystal structures and 1,037 AlphaFold-predicted structures.

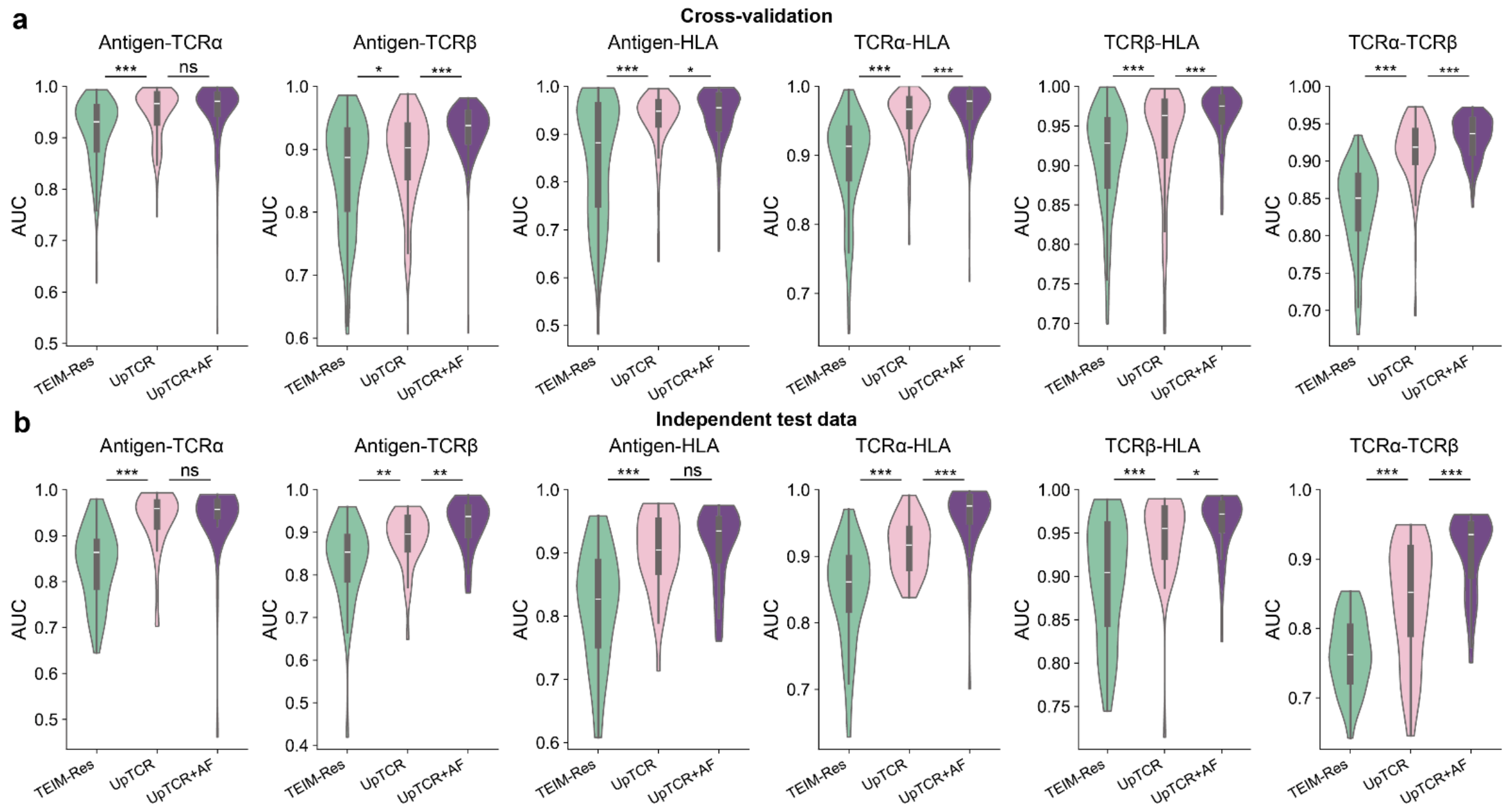

Figure 6. UpTCR facilitates investigations of any pairwise residue contact relationship among full-length TCRα, full-length TCRβ, Antigen, and HLA. a-b, *Violin plots displaying the cross-validation (**a**) and independent test (**b**) AUC for predicting residue-level contact maps between any pairwise components by TEIM-Res, UpTCR, and UpTCR+AF. From left to right: antigen-TCRα, antigen-TCRβ, antigen-HLA, TCRα-HLA, TCRβ-HLA, and TCRα-TCRβ. UpTCR+AF denotes UpTCR further trained with additional high-confidence AlphaFold-predicted complex structures. The independent test set comprises 39 TCR-antigen-HLA complex structures that were excluded from all stages of pretraining and fine-tuning.*

Comment 4)

In the cross-reactivity analysis, the three antigen variants recognized by the same TCR appear to be highly similar in sequence. I wonder how does UpTCR perform when antigens differ by single-point mutations that disrupt binding? And if you have performed some analysis of the UpTCR performance when sequence-divergent peptides bind the same TCR? If available, results addressing these more challenging and relevant scenarios would be a plus.

Response: We thank the reviewer for raising these two challenging and biologically important scenarios. We have addressed both explicitly in the revised manuscript.

(1) Single-point mutations that disrupt binding. We constructed a dedicated hard-negative evaluation set in which each true binding epitope is paired with closely related non-binding variants differing by only 1–4 residues, all presented by the same HLA and engaging the same TCR. This benchmark comprises 3,495 binder/non-binder pairs, of which 92.7% differ by a single amino acid substitution. On this stringent set, UpTCR achieved a pairwise accuracy of 0.799, consistently outperforming DLpTCR, PanPep, and EPACT across all metrics (Supplementary Figure S24). These results demonstrate that the model is sensitive to binding-disrupting point mutations rather than merely detecting overall sequence similarity.

To be clearer, the revised content is presented below.

Results (page 10-11):

To further evaluate UpTCR's discriminative power in a biologically stringent setting, we constructed a hard-negative benchmark comprising 3,495 TCR-epitope pairs. In each pair, a TCR is matched with one experimentally confirmed binding epitope and one non-binding homologous epitope differing by only a few amino acid substitutions (92.7% with a single substitution) and originating from the same pathogen. This design ensures that the model cannot exploit global sequence dissimilarity and must instead identify binding-critical residue positions. UpTCR achieved a pairwise accuracy of 0.799, consistently outperforming DLpTCR, PanPep, and EPACT across all metrics (Supplementary Figure S24), demonstrating its ability to discriminate biologically plausible hard negatives from true binders.

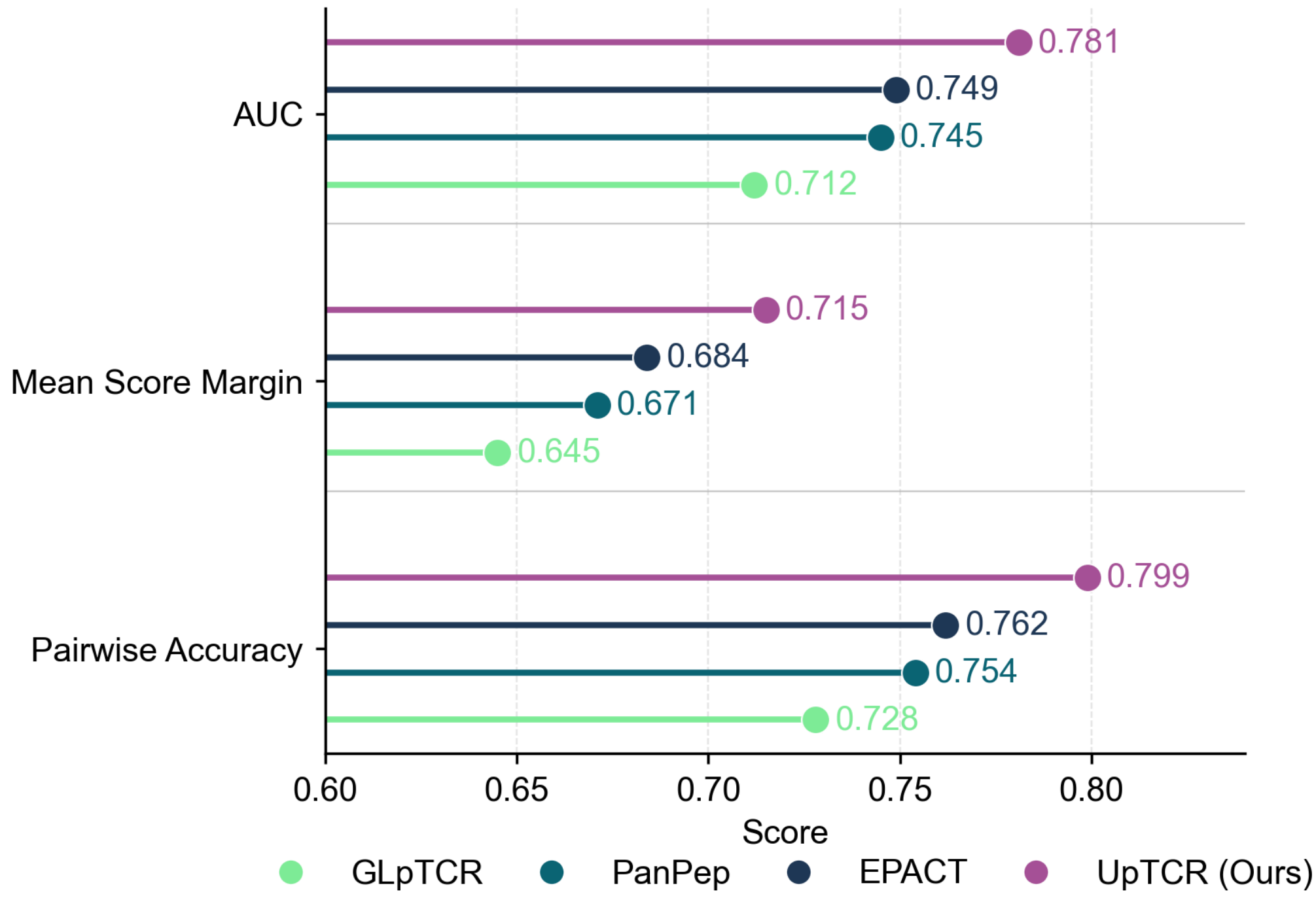

Figure S24. Hard negative discrimination performance on mutation epitope pairs. *Comparison of UpTCR and three baseline methods on a hard negative benchmark consisting of 3,495 TCR-epitope pairs, where each TCR is paired with one confirmed binding epitope and one non-binding epitope differing by amino acid substitutions. Performance was assessed by three metrics: pairwise accuracy (proportion of pairs where the binding epitope received a higher predicted score than the non-binding epitope), mean score margin (average difference between positive and negative prediction scores), and area under the receiver operating characteristic curve (AUC). UpTCR consistently outperformed all baselines across all metrics.*

Sequence-divergent peptides binding the same TCR. This scenario is directly captured by our low-conservation cross-reactivity groups (conservation ratio < 0.5), in which the antigens recognized by the same TCR share fewer than half of their residue positions and exhibit high sequence diversity. On these 10 TCR groups, UpTCR achieved significant discrimination between binding and non-binding antigens in all cases ($P < 0.05$, Figure 5f), assigning a mean predicted probability of 0.801 to confirmed cross-reactive antigens versus 0.347 to non-binding controls (2.30-fold difference), with an average classification accuracy of 74.2%. While this performance is expectedly lower than for the high-conservation group (90.3%), it demonstrates that UpTCR captures binding patterns beyond simple sequence similarity, consistent with its progressive pretraining on diverse interaction data that enables generalization to sequence-divergent binding partners.

Results (page 25):

In this study, we collected data on 20 groups of TCR cross-reactivity (see Methods, Supplementary Table S1), with each TCR binding to multiple different antigens. For each TCR group, we additionally sampled the same number of non-binding antigens as negative controls to assess the model's discriminative power. … A position was regarded as conserved if a specific amino acid residue appeared at that position in more than half of the sequences. The conservation ratio (CR) was calculated as the number of conserved positions relative to the total length of the multiple sequence alignment. Based on this, we categorized the TCR cross-reactivity data into two groups: the high-conservation group (CR > 0.5) and the low-conservation group (CR < 0.5).

…

In the low-conservation group, UpTCR also achieved significant discrimination for all 10 TCR groups (P < 0.05, Figure 5f), with a mean predicted probability of 0.801 for binding antigens versus 0.347 for non-binding antigens (2.30-fold difference) and an average accuracy of 74.2%.

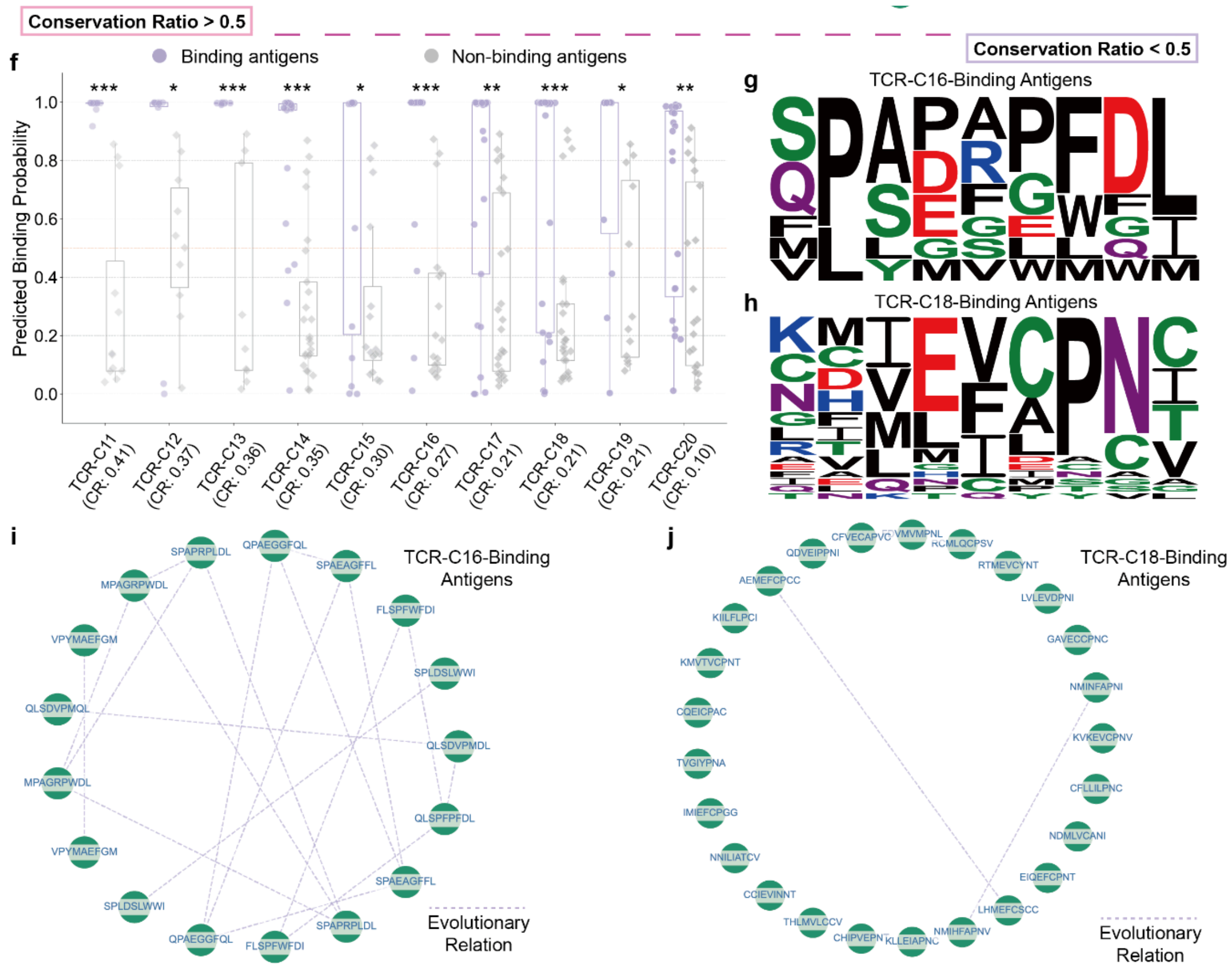

Figure 5. *… **f,** Predicted binding probabilities for 10 cross-reactive TCR groups in the low-conservation group. Purple dots represent confirmed cross-reactive antigens and gray diamonds represent non-binding antigens.* ***g,h,*** *Sequence motifs of the cross-reactive antigens targeting TCR-C16 (**g**) and TCR-C18 (**h**).* ***i,j,*** *Sequence homology networks for antigens targeting*

*TCR-C16 (i) and TCR-C18 (j). The sparse connectivity in the low-conservation group reflects high sequence diversity among cross-reactive antigens. *P < 0.05, **P < 0.01, ***P < 0.001.*

Comment 5)

EPACT appears to be a strong competitor. For example, in Supplementary Figure S8a, the performance difference between EPACT and UpTCR is not statistically significant in the "majority setting". Given this, it is unclear why, in the section "Translational validation of UpTCR in breast cancer across different subtypes", the benchmark comparison is performed only against PanPep and not EPACT. The authors could justify this choice or include EPACT in the comparison.

Response: We thank the reviewer for this pertinent observation. We agree that EPACT is a strong competitor and that its omission from the breast cancer translational validation was an oversight in the original manuscript. In the revised manuscript, we have included EPACT alongside PanPep in the translational validation benchmark for both the Luminal and TNBC cohorts. The updated results (revised Figure 7b-e) demonstrate that UpTCR consistently outperforms both EPACT and PanPep across all transfer data ratios (10%–40%) in terms of AUC, AUPR, Precision, Recall, and F1-score.

To be clearer, the revised content is presented below.

Results (page 28):

We benchmarked UpTCR against PanPep and EPACT, two competitive methods known for their transferability with limited training data, under varying amounts of transfer data (10% to 40%). In both Luminal (Figure 7b) and TNBC (Figure 7c) cohorts, UpTCR consistently achieved superior performance in both AUC and AUPR under various transfer data. Notably, UpTCR reached high AUPR using only 10% of transfer data, whereas PanPep required 40% to achieve comparable performance (Figure 7b,c, right). Further comprehensive quantitative evaluation (Figure 7d,e) demonstrated UpTCR's superiority across multiple key metrics, including Precision, Recall, AUC, AUPR, and F1-score in both breast cancer subtypes at different transfer data ratios. This highlights UpTCR's robustness and efficiency in few-shot samples, which is critical for personalized immunotherapy applications where patient-specific data are often scarce.

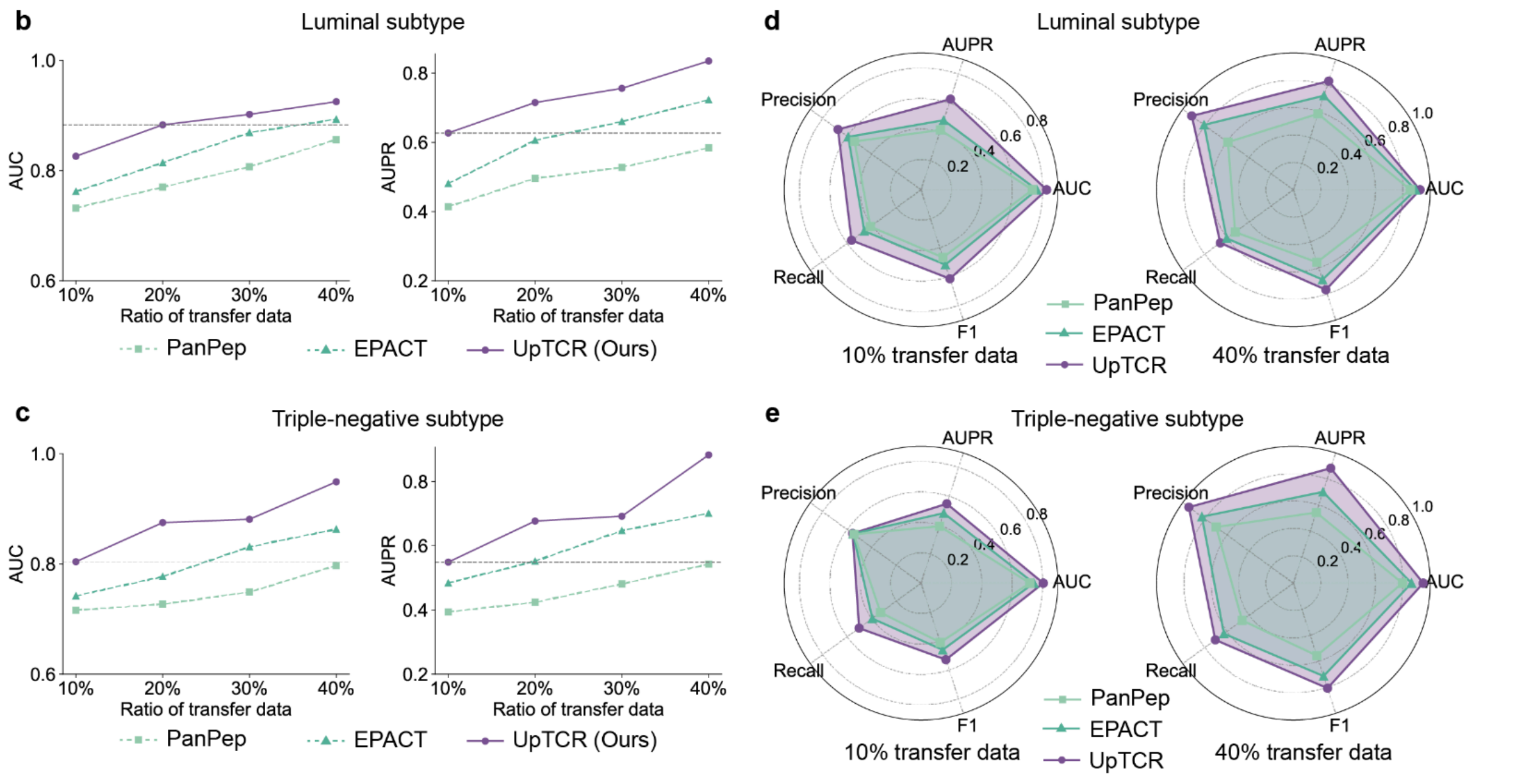

***Figure 7. … b,c,** AUC (left) and AUPR (right) performance of UpTCR, EPACT, and PanPep in predicting TCR binding specificity across the Luminal (**b**) and TNBC (**c**) subtypes with increasing ratios of transfer data (10%-40%). UpTCR consistently outperforms both EPACT and PanPep across all transfer data ratios. **d,e,** Radar plots quantitatively comparing UpTCR, EPACT, and PanPep across five key metrics (AUC, AUPR, Precision, Recall, and F1-score) for the Luminal (**d**) and TNBC (**e**) subtypes at 10% (left) and 40% (right) transfer data ratios. UpTCR dominates across all metrics in both subtypes, with the advantage being most pronounced in the low-data regime (10%).*

Comment 6)

What is AUC when truncated at a false positive rate of 0.1 (i.e., AUC0.1)? This metric is particularly informative and a good complement to the analysis performed, as it emphasizes early retrieval performance, how well the model performs when only the most confident predictions are considered. Moreover, only a small number of prediction can be tested experimentally. Could the authors also provide AUC0.1 as a comparison metric in the benchmrak? AUC0.1 values per antigen in each set and for each tool would be a plus.

Response: We thank the reviewer for this excellent suggestion. We agree that AUC0.1 (partial AUC at FPR ≤ 0.1) is a highly informative metric that captures early retrieval performance, a property of particular practical relevance, as only a limited number of computational predictions can typically be validated experimentally.

In the revised manuscript, we have computed AUC0.1 for all benchmark methods across all four evaluation settings (majority, few-shot, zero-shot with 50% sequence identity filtering, and zero-shot with 30% sequence identity filtering). The results are presented in a new Supplementary Figure, which includes: (1) bar plots of normalized AUC0.1 for each method under all four settings, and (2) corresponding partial ROC curves (FPR ≤ 0.1) visualizing the discriminative performance within the high-specificity regime. UpTCR consistently achieved the highest AUC0.1 across all settings, with statistically significant improvements over all baseline methods.

To clarify these revisions, the revised content is presented below.

Results (page 10):

To further assess the early retrieval performance of UpTCR, we computed the partial AUC at a false positive rate of 0.1 (AUC0.1) across all four settings (Supplementary Figure S8). UpTCR consistently achieved the highest AUC0.1 among all benchmark methods ($P < 0.01$) across all settings, demonstrating superior discriminative performance within the high-specificity regime. The result indicates that UpTCR's most confident predictions are reliable for prioritizing candidates for downstream experimental validation.

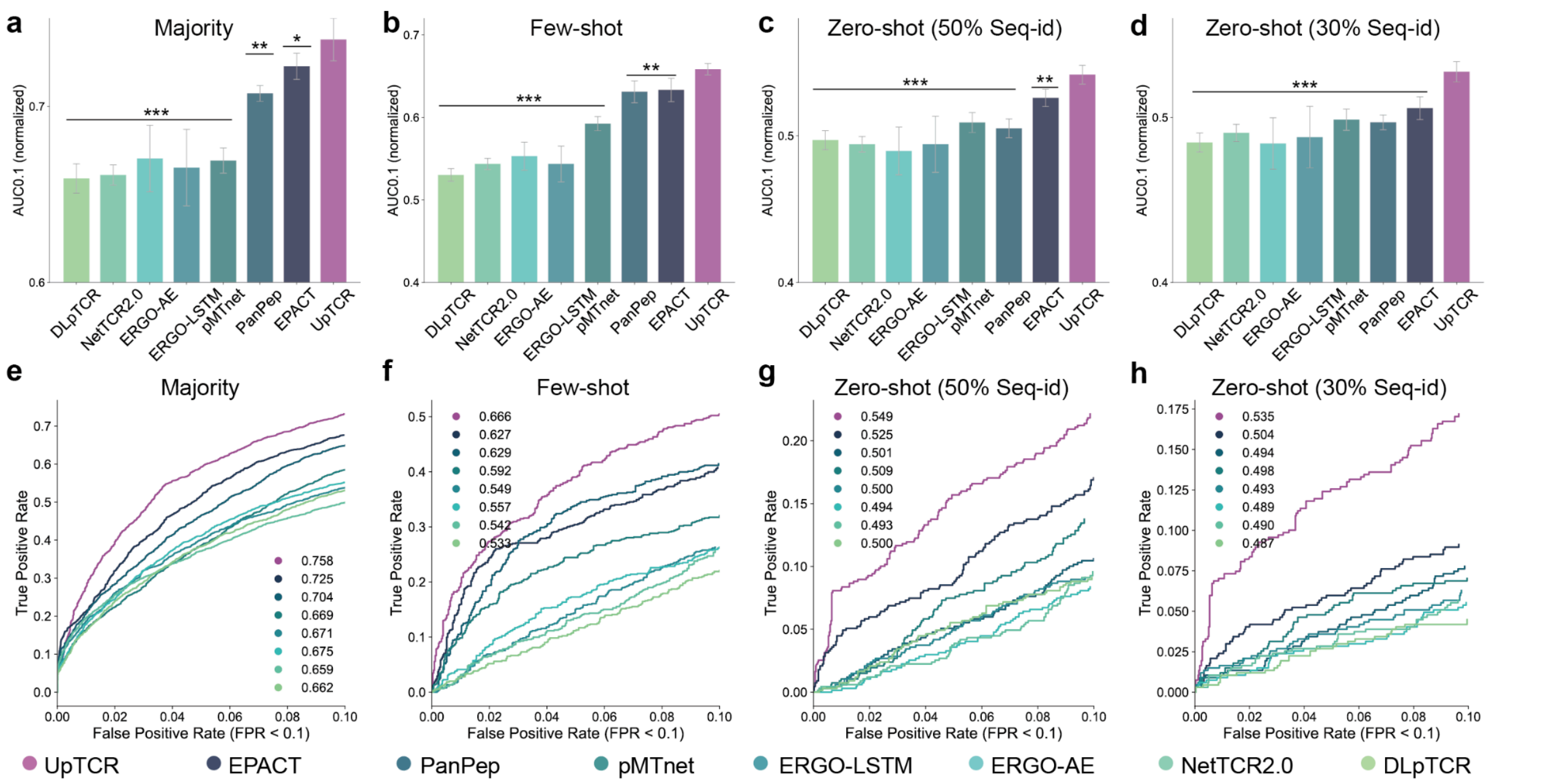

Figure S8. Early retrieval performance assessed by partial AUC at a false positive rate of 0.1 (AUC0.1) across four evaluation settings. a-d, *Bar plots of normalized AUC0.1 for all benchmark methods under the majority (**a**), few-shot (**b**), zero-shot with 50% sequence identity filtering (**c**), and zero-shot with 30% sequence identity filtering (**d**) settings. AUC0.1 quantifies the discriminative performance within the high-specificity regime (FPR ≤ 0.1), reflecting how well each model ranks its most confident predictions.* ***e-h,*** *Corresponding partial ROC curves (FPR ≤*

*0.1) for the majority (**e**), few-shot (**f**), zero-shot with 50% sequence identity filtering (**g**), and zero-shot with 30% sequence identity filtering (**h**) settings. UpTCR consistently achieves the highest AUC0.1 across all settings. * P < 0.05, ** P < 0.01, *** P < 0.001, ns indicates no significant difference.*

Comment 7)

The manuscript refers to Atchley factors and Blosum information as "biomedical properties" (Results, line 150, and Figure 1). These are more accurately described as bio-physicochemical rather than biomedical properties. The authors should clarify why the term "biomedical" is used here and in what sense these properties are considered biomedical.

Response: We thank the reviewer for this correction. We agree that the term "biomedical" is imprecise in this context. Atchley factors encode quantitative biophysical and biochemical attributes of amino acids (such as polarity, secondary structure propensity, molecular volume, codon diversity, and electrostatic charge), while BLOSUM matrices capture evolutionary substitution frequencies. These are more accurately characterized as biophysicochemical properties rather than biomedical properties.

We have revised "biomedical properties" to "bio-physicochemical properties" throughout the manuscript. To be clearer, the revised content is presented below.

Results (page 6):

For HLA, pseudo sequence embeddings and bio-physicochemical properties (*e.g.*, Atchley factor and BLOSUM) are used.

Reviewer #2 (Remarks to the Author):

Summary

A neural network method, UpTCR is developed to predict TCR-MHC-antigen complexes, and it also provides some mechanistic insights to TCR recognition. Pairwise information (TCR-antigen or antigen-MHC) is 5.6x more abundant than tertiary (TCR-Antigen-HLA) and the method conceptually is capitalizing on exploiting it.

Response: We sincerely thank Reviewer #2 for the thorough and encouraging evaluation, and for recognizing the central conceptual contribution of UpTCR, capitalizing on the abundant pairwise data to improve binding prediction performance. We address each of the points raised in detail below.

Comment 1)

The head-head comparisons of performance show an improvement over current state of the art. Most valuable results of the method when it can utilize generalizations to predict novelty (looking at: specialized zero shot training cases). However, several issues need to be addressed to clarify the actual performance. Also, it is not clear how much the method benefits from the integration of pairwise data, as claimed, or something else.

Response: We thank the reviewer for this comprehensive assessment. We agree that clarifying the actual performance under rigorous conditions and disentangling the contributions of pairwise data integration from other factors are essential.

In the revised manuscript, we have comprehensively addressed these issues as follows:

(1) Rigorous performance evaluation. Based on the analysis of pairwise sequence identity distributions among functionally related sequences, we adopted a stricter 30% sequence identity threshold for pretraining data de-redundancy to eliminate potential information leakage (addressed in Comment 2). For the downstream benchmark, we evaluated zero-shot generalization under two sequence-similarity filtering levels between training and test sets: 50% and 30%. Under both conditions, UpTCR consistently and significantly outperformed all baselines, confirming that its advantage reflects generalization rather than residual homology (addressed in Comments 2 and 3).

(2) Quantifying the benefit of pairwise data integration. To directly address whether the improvement stems from the progressive pretraining, we performed: (a) a multi-component ablation revealing synergistic contributions among pretrained modules that are masked when only individual modules

are ablated (addressed in Comment 7); (b) modality-missing evaluations showing that all variants with partial data still outperform baselines (addressed in Comment 8); and (c) a stage-wise ablation demonstrating that the progressive Stage I → Stage II structure provides a 6.9% AUC improvement in zero-shot that neither stage achieves alone (addressed in Comment 7).

(3) Fair benchmarking. All competitor models were retrained using the identical curated and filtered dataset to ensure the fair benchmarking.

Collectively, these analyses demonstrate that UpTCR's performance improvement is driven by the synergistic combination of progressive pretraining and multi-scale feature optimization, with both components making essential and quantifiable contributions.

Comment 2)

Pre-training conditions need more justification. Antigen and TCR sequences with 50% or higher identity were removed, but there are many functionally similar cases at lower sequence identity levels. e.g. cutoff for antigen sequence identity should/could be justified through the analysis of sequence variability among the same HLA binders.

Response: We thank the reviewer for this important methodological point. We agree that functionally similar peptides can exist below 50% sequence identity, and that the chosen threshold should be empirically justified. Following this suggestion, we have adopted a more stringent filtering criterion and provide both empirical justification and a comprehensive sensitivity analysis.

1) Stricter pretraining data filtering. Following the reviewer's suggestion, we analyzed the distribution of pairwise sequence identities among experimentally confirmed binders presented by the same HLA allele (Figure S23a). The median pairwise antigen identity among same-HLA binders was 0.444 and the mean was 0.520, suggesting that functionally related sequences can persist above the original 50% threshold. For TCR CDR regions, the median pairwise identity among TCRs recognizing the same antigen-HLA pair was 0.389 (TCRα) and 0.471 (TCRβ) (Figure S23b,c). Guided by these empirical distributions, we adopted a stricter 30% sequence-identity threshold for pretraining data filtering to minimize potential information leakage from the pretraining stage to downstream tasks. The model was re-pretrained with this filtered dataset. Thus, we note that due to the more stringent de-redundancy filtering applied to the pretraining data (30%), the absolute performance values exhibit slight variations compared to those reported in the original manuscript.

2) Additional 30% zero-shot evaluation. In addition to the original 50% sequence-identity zero-shot setting, we now include a more stringent 30% zero-shot evaluation (revised Figure 2d,h,l,p). Under

this challenging condition, UpTCR (AUC = 0.594) remained significantly superior to all baselines including the strongest competitor EPACT (AUC = 0.522, $P < 0.001$). Notably, compared with the 50% zero-shot setting, UpTCR maintained the highest absolute performance under 30% filtering (AUC 0.594 vs. EPACT 0.522).

We have added these analyses to the revised manuscript and expanded Figure 2 to include the 30% sequence-identity filtering setting.

Methods - (page 37):

A pretraining entry was excluded if any component shared greater than 30% sequence identity with the downstream test data (antigen sequence identity > 30%, TCRα CDR identity > 30%, or TCRβ CDR identity > 30%). This uniform threshold was empirically justified: the median pairwise identities among functionally related sequences (same-HLA binders: 0.444; same-antigen-HLA TCRα: 0.389; TCRβ: 0.471; Supplementary Figure S23) all fall above 30%, confirming that this threshold effectively eliminates the majority of potentially confounding homologous pairs.

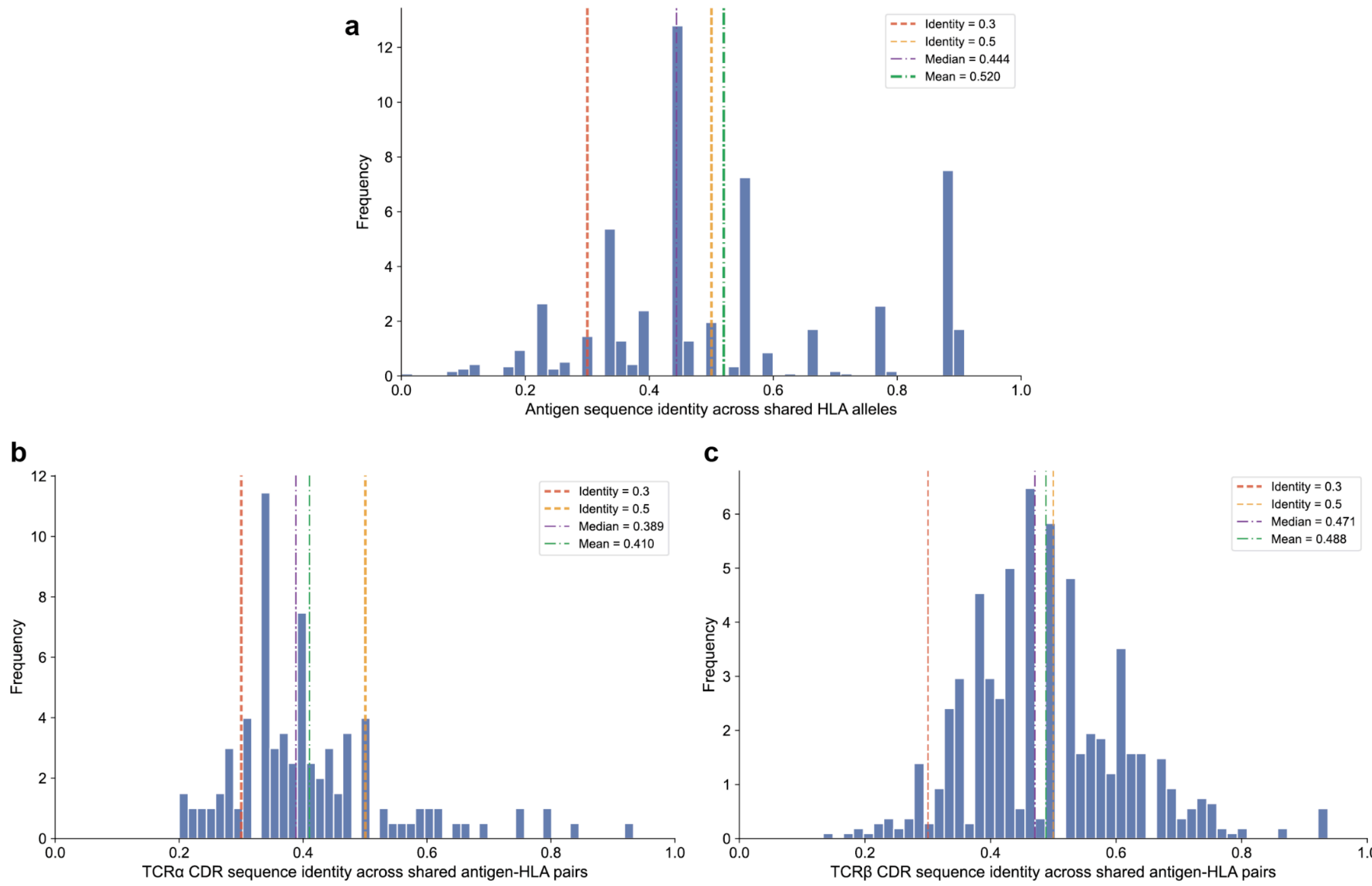

Figure S23. Pairwise sequence identity distributions among functionally related sequences. a, *Distribution of pairwise antigen sequence identity among peptides that bind the same HLA allele. The median identity is 0.444 and the mean is 0.520.* ***b,*** *Distribution of pairwise TCRα CDR sequence identity among TCRs recognizing the same antigen-HLA pair. The median identity is*

0.389 and the mean is 0.410, with the majority of pairs falling between the 30% and 50% thresholds. ***c,*** *Distribution of pairwise TCRβ CDR sequence identity among TCRs recognizing the same antigen-HLA pair. The median identity is 0.471 and the mean is 0.488.*

Results - (page 10):

…We then assessed UpTCR in two zero-shot settings of increasing stringency, where none of the antigens in the test set appeared in the training data. Under 50% sequence identity filtering, UpTCR achieved an AUC of 0.642 and AUPR of 0.296, exceeding the next-best baseline by 6.5% in AUC (Figure 2c,g). Under a more stringent 30% threshold, UpTCR maintained the highest AUC of 0.594 and AUPR of 0.252, outperforming all baselines (*P* < 0.001, Figure 2d,h). The corresponding ROC and PR curves across all four settings (Figure 2i-p) further corroborated these findings across all four settings.

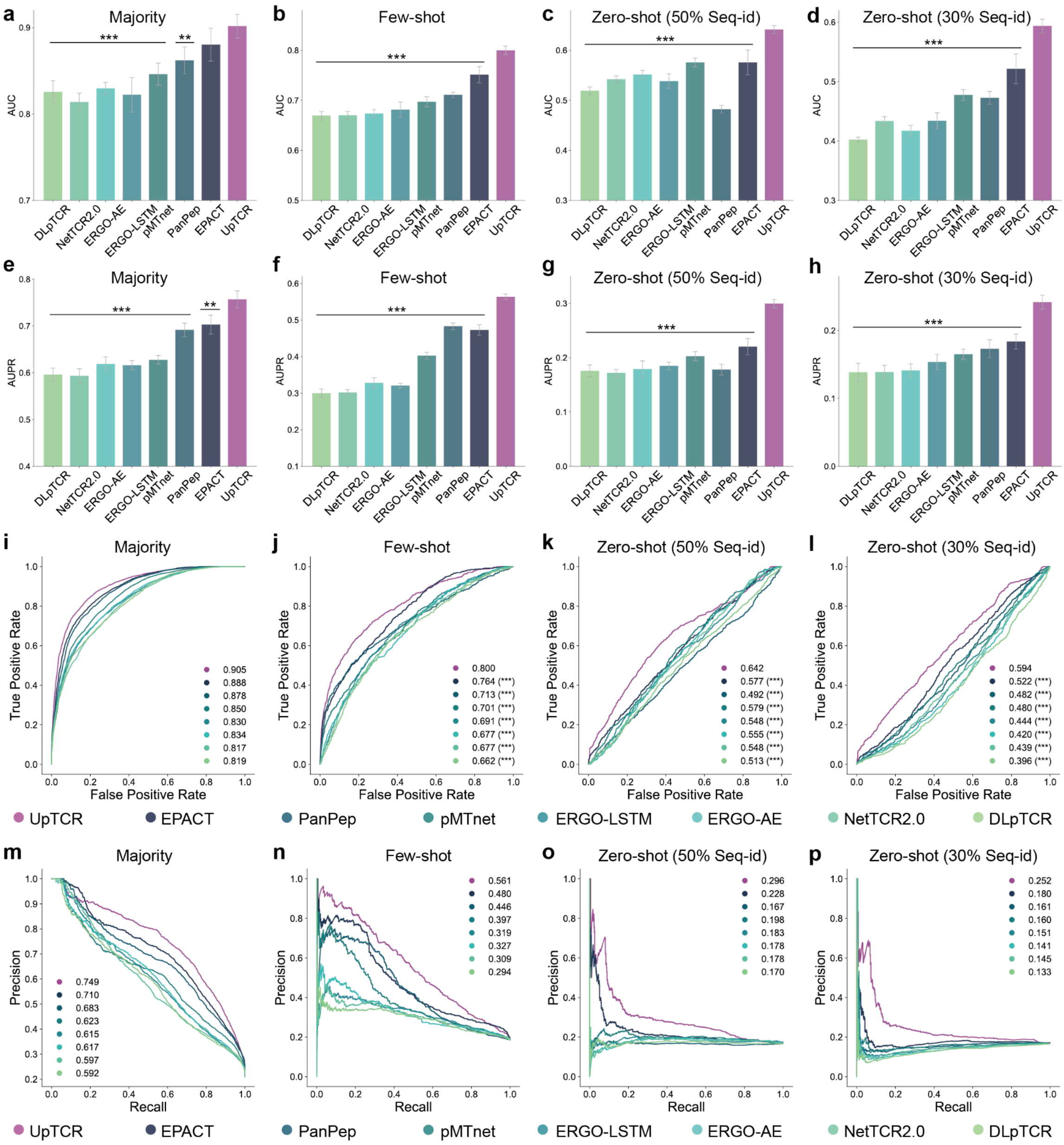

Figure 2. UpTCR achieves superior performance in predicting TCR-antigen-HLA binding specificity across diverse evaluation settings. a-d, *Bar plots of AUC for all benchmark methods under the majority (**a**), few-shot (**b**), zero-shot with 50% sequence identity filtering (**c**), and zero-shot with 30% sequence identity filtering (**d**) settings. The few-shot setting refers to a scenario in which only a limited number of training samples are available for each antigen. The zero-shot setting represents a more challenging scenario in which the model is required to predict TCR-antigen-HLA binding specificity for antigens that are entirely absent from the training data, with sequence identity filtering applied to ensure strict separation between training and test sets. Bars represent the mean AUC and error bars indicate standard deviations.* ***e-h,*** *Bar plots of AUPR under the same four settings: majority (**e**), few-shot (**f**), zero-shot with 50% sequence identity filtering (**g**), and zero-shot with 30% sequence identity filtering (**h**).* ***i–l,*** *Receiver Operating Characteristic (ROC) curves for all benchmark methods under the majority (**i**), few-shot (**j**), zero-shot with 50% sequence identity filtering (**k**), and zero-shot with 30% sequence identity filtering (**l**) settings. Each curve represents one method, with the legend indicating the AUC value. Statistical significance of the improvement in AUC was assessed by DeLong's test.* ***m–p,*** *Precision-Recall (PR) curves for all benchmark methods under the majority (**m**), few-shot (**n**), zero-shot with 50% sequence identity filtering (**o**), and zero-shot with 30% sequence identity filtering (**p**) settings. * P < 0.05, ** P < 0.01, *** P < 0.001.*

Comment 3)

This previous question is also prompted by the alarming difference in performance when comparing zero-shot and specialized zero shot results (going from 0.841 to 0.682 AUC) and thus it is unclear if more strict filtering conditions could further affect these results. And if significant results largely reserved for cases with reasonable templates in the training set.

Response: We thank the reviewer for this important concern regarding the performance gap between the original zero-shot settings and the potential influence of residual template homology.

We clarify that in the original manuscript, the standard zero-shot setting only ensured that antigens in the test set were absent from the training set by name, without imposing any sequence similarity constraint. In contrast, the specialized zero-shot setting enforced a strict 50% sequence identity filtering threshold. This difference in filtering stringency, rather than a fundamental limitation of the model, was the primary factor driving the observed performance gap (0.841 -> 0.682 AUC).

We recognize that a zero-shot evaluation without explicit sequence similarity filtering does not adequately guard against information leakage through homologous sequences and may overestimate generalization performance. To present a more rigorous and conservative evaluation, we have removed the original unfiltered zero-shot results from the revised manuscript. In the revised Figure 2, we now present two zero-shot settings with explicit sequence similarity constraints: (1) zero-shot with 50% sequence identity filtering, and (2) zero-shot with 30% sequence identity filtering.

Critically, to address the reviewer's concern about whether the results are "largely reserved for cases with reasonable templates", we note that under the 30% filtering condition, where no close homologs exist in the training set, UpTCR achieves an AUC of 0.594 and AUPR of 0.252, significantly outperforming all baselines ($P < 0.001$). The decrease from 50% to 30% filtering is moderate (~5% in AUC), and UpTCR's relative advantage over competing methods is preserved under both conditions. These results demonstrate that UpTCR's performance is not contingent upon the availability of closely homologous templates in the training set, but rather reflects the model's ability to learn transferable binding principles through progressive pretraining.

To be clearer, the revised content is presented below.

Result (page 10):

In the majority setting, where the majority of relevant data for each antigen is present in the training set, UpTCR achieved an AUC (Area Under the Curve) of 0.905 and an AUPR (Area Under the Precision-Recall Curve) of 0.749, outperforming all baselines (Figure 2a,e). In the few-shot setting, where only a limited number of training samples are available for each antigen, UpTCR attained an AUC of 0.800 and AUPR of 0.561, outperforming the next-best method (EPACT) by 3.6% in AUC and 8.1% in AUPR (Figure 2b,f). We then assessed UpTCR in two zero-shot settings of increasing stringency, where none of the antigens in the test set appeared in the training data. Under 50% sequence identity filtering, UpTCR achieved an AUC of 0.642 and AUPR of 0.296, exceeding the next-best baseline by 6.5% in AUC (Figure 2c,g). Under a more stringent 30% threshold, UpTCR maintained the highest AUC of 0.594 and AUPR of 0.252, outperforming all baselines ($P < 0.001$, Figure 2d,h). The corresponding ROC and PR curves across all four settings (Figure 2i-p) further corroborated these findings across all four settings.

The revised Figure 2 and its legend are provided in response to Comment 2 above (page 22-23).

Comment 4)

The most convincing results are the head-to-head comparisons between different methods (irrespectively of the type of datasets), where (I assume) the same datasets were used for all NN training, pre-training and testing. This fact should be discussed clearly.

Response: We thank the reviewer for highlighting this important point. The reviewer's assumption is correct: all machine learning-based competitor models were retrained using the identical curated and filtered training dataset as UpTCR. This standardized protocol was consistently applied across all evaluation settings (majority, few-shot, and zero-shot) to ensure that observed performance differences reflect solely methodological advantages rather than variations in training data.

We have made this point more explicit in the revised manuscript as below.

Results - (page 10):

To ensure a rigorous and fair comparison, all competing models were implemented and retrained using the same curated and filtered dataset utilized for UpTCR.

Comment 5)

In case of TCR, the sequence identity comparison should focus on the functionally relevant and variable CDR regions, not the full protein.

Response: We thank the reviewer for this suggestion. We agree that the CDR regions are the functionally relevant and variable segments responsible for antigen recognition, and that sequence identity filtering based on the full-length TCR protein would be less meaningful due to the high conservation of framework regions.

We would like to clarify that in our implementation, TCR sequence identity filtering was indeed performed on the CDR regions. We acknowledge that some descriptions in the original manuscript were insufficiently clear. We have revised all relevant descriptions throughout the manuscript to consistently and clearly state "TCR CDR region sequence identity", ensuring no confusion about the scope of the filtering.

To be clearer, the revised content is presented below.

Methods (page 37):

A pretraining entry was excluded if any component shared greater than 30% sequence identity with the downstream test data (antigen sequence identity > 30%, TCRα CDR identity > 30%, or TCRβ CDR identity > 30%).

Comment 6)

Discussion is missing about the unusual results that the few-shot testing is (although not much but) inferior to Zero-Shot training (0.824 vs 0.841).

Response: We thank the reviewer for this astute observation. We agree that this result appears counterintuitive and warrants clarification.

Upon re-examination, we identified that the original zero-shot setting only ensured that antigens in the test set were absent from the training set, without applying sequence similarity filtering. As a result, homologous data could still be present in the training data, leading to inflated performance that exceeded those of the few-shot setting.

We recognize that for a meaningful zero-shot evaluation, sequence similarity filtering is essential to prevent potential information leakage through homologous sequences. In the revised manuscript, we have therefore removed the original unfiltered zero-shot results and instead present two zero-shot settings with explicit sequence similarity constraints: 50% and 30% sequence identity filtering. Note that due to the more stringent de-redundancy filtering applied to the pretraining data (30%), the absolute performance values exhibit slight variations compared to those reported in the original manuscript. Under these revised settings, the performance follows the expected trend: few-shot (AUC 0.800) > zero-shot at 50% filtering (AUC 0.642) > zero-shot at 30% filtering (AUC 0.594). The stricter filtering increases the difficulty of generalization. Importantly, UpTCR consistently outperforms all baselines across all settings, confirming its robustness under stringent evaluation conditions.

To be clearer, the revised content is presented below.

Results (page 10):

In the majority setting, where the majority of relevant data for each antigen is present in the training set, UpTCR achieved an AUC (Area Under the Curve) of 0.905 and an AUPR (Area Under the Precision-Recall Curve) of 0.749, outperforming all baselines (Figure 2a,e). In the few-shot setting, where only a limited number of training samples are available for each antigen,

UpTCR attained an AUC of 0.800 and AUPR of 0.561, outperforming the next-best method (EPACT) by 3.6% in AUC and 8.1% in AUPR (Figure 2b,f). We then assessed UpTCR in two zero-shot settings of increasing stringency, where none of the antigens in the test set appeared in the training data. Under 50% sequence identity filtering, UpTCR achieved an AUC of 0.642 and AUPR of 0.296, exceeding the next-best baseline by 6.5% in AUC (Figure 2c,g). Under a more stringent 30% threshold, UpTCR maintained the highest AUC of 0.594 and AUPR of 0.252, outperforming all baselines (P < 0.001, Figure 2d,h). The corresponding ROC and PR curves across all four settings (Figure 2i-p) further corroborated these findings across all four settings.

The revised Figure 2 and its legend are provided in response to Comment 2 above (page 22-23).

Comment 7)

Interesting that (Fig 3D) pretraining with TCR or HLA only (i.e. using pairwise complexes), results in statistically indistinguishable performance from the full pretraining approach. This might suggest that most of the advance is coming from the actual technical details (or some other factors?) of the method and NOT from the conceptual advance of including TCR-antigen-HLA training simultaneously. This should be discussed and further explored (and it is also in contrast to lines 673-678 where it is argued to be a "distinctive advantage")

Response: We thank the reviewer for this insightful observation and the opportunity to clarify the respective contributions of conceptual design and technical implementation.

We agree that the original Figure 3d shows that ablating certain individual pretrained modules results in performance that is statistically indistinguishable from the full model in some metrics. However, we respectfully note that this does not imply the pretrained modules are dispensable; rather, it reflects a compensatory mechanism inherent in the progressive pretraining framework, when one module's pretraining is removed, the remaining modules can partially offset the loss, masking individual contributions.

To directly verify this hypothesis, we performed multi-component ablation experiments in which pairs of these individually non-significant modules were simultaneously removed. All pairwise combinations resulted in statistically significant performance declines (P < 0.05, Supplementary Figure S19), confirming that the compensatory capacity is disrupted when multiple modules are ablated together. Removing all pretraining entirely led to the largest decline (AUC decreased by 8.8% in the few-shot setting and 11.1% in the zero-shot setting).

To further address the reviewer's broader question of whether the improvement stems from the conceptual advance (progressive pretraining design) or merely from technical details, we point to the complementary evidence from the stage-wise ablation (Supplementary Figure S20). That analysis demonstrates that the progressive structure itself, specifically, the sequential transfer from Stage I to Stage II, provides significant marginal gains (*e.g.*, 6.9% AUC improvement in zero-shot) that cannot be achieved by either stage alone. This confirms that the progressive pretraining design represents a genuine conceptual contribution beyond any individual technical component.

We have revised the Discussion to more accurately characterize this finding, replacing the original "distinctive advantage" language with a more precise description acknowledging that UpTCR's performance arises from the synergistic combination of progressive pretraining design and multi-scale feature engineering.

Results (pages 13-14):

Although ablation of certain individual modules (TCRαformer, HLAformer, TCRαβFusionformer) resulted in statistically indistinguishable performance degradation across some metrics and settings, this did not indicate that these modules are dispensable. This may be due to a compensatory mechanism, whereby remaining pretrained modules can partially compensate for the loss of a single component. To verify this, we performed multi-component ablation experiments in which pairs of these individually non-significant modules were simultaneously removed. Ablation of any two of these modules concurrently resulted in statistically significant performance declines compared to the full model ($P < 0.05$, Supplementary Figure S19). Furthermore, removal of all pretraining modules led to a substantial performance decline (*e.g.*, AUC decreased by 8.8% in the few-shot setting and 11.1% in the zero-shot setting). The above results confirm the critical importance of the pretrained modules.

To clarify the respective contributions of the two pretraining stages, we performed a stage-wise ablation study comparing four configurations: No Pretrain, Only Stage I (dimer-level pretraining), Only Stage II (trimer-level pretraining without Stage I initialization), and the complete UpTCR. Under the few-shot setting, transitioning from Only Stage I to Full UpTCR yielded improvements of 4.8% in AUC and 8.0% in AUPR; under the zero-shot setting, the marginal gains were 6.9% in AUC and 8.1% in AUPR. Both Only Stage I and Only Stage II performed significantly worse than Full UpTCR ($P < 0.001$, Supplementary Figure S20), yet achieved comparable levels to each other (e.g., few-shot AUC: 0.752 versus 0.745) , indicating that the two stages contribute complementary rather than redundant knowledge. Stage I establishes foundational molecular

representations by aligning the embeddings of individual components into a shared latent space, whereas Stage II builds upon these representations to capture higher-order interaction patterns by integrating fused TCRαβ and antigen-HLA complexes through trimer-level contrastive learning.

Discussion (page 34):

UpTCR employs holistic modeling of the full-length TCR, incorporating not only the dominant CDR3 loops but also the complementary variable regions, including CDR1, CDR2, and CDR2.5, alongside the framework regions. This comprehensive representation captures the multifaceted nature of TCR binding, reflecting the biological reality where multiple segments contribute synergistically to antigen-HLA recognition. The multi-component and stage-wise ablations confirm that these modules function synergistically and are collectively indispensable.

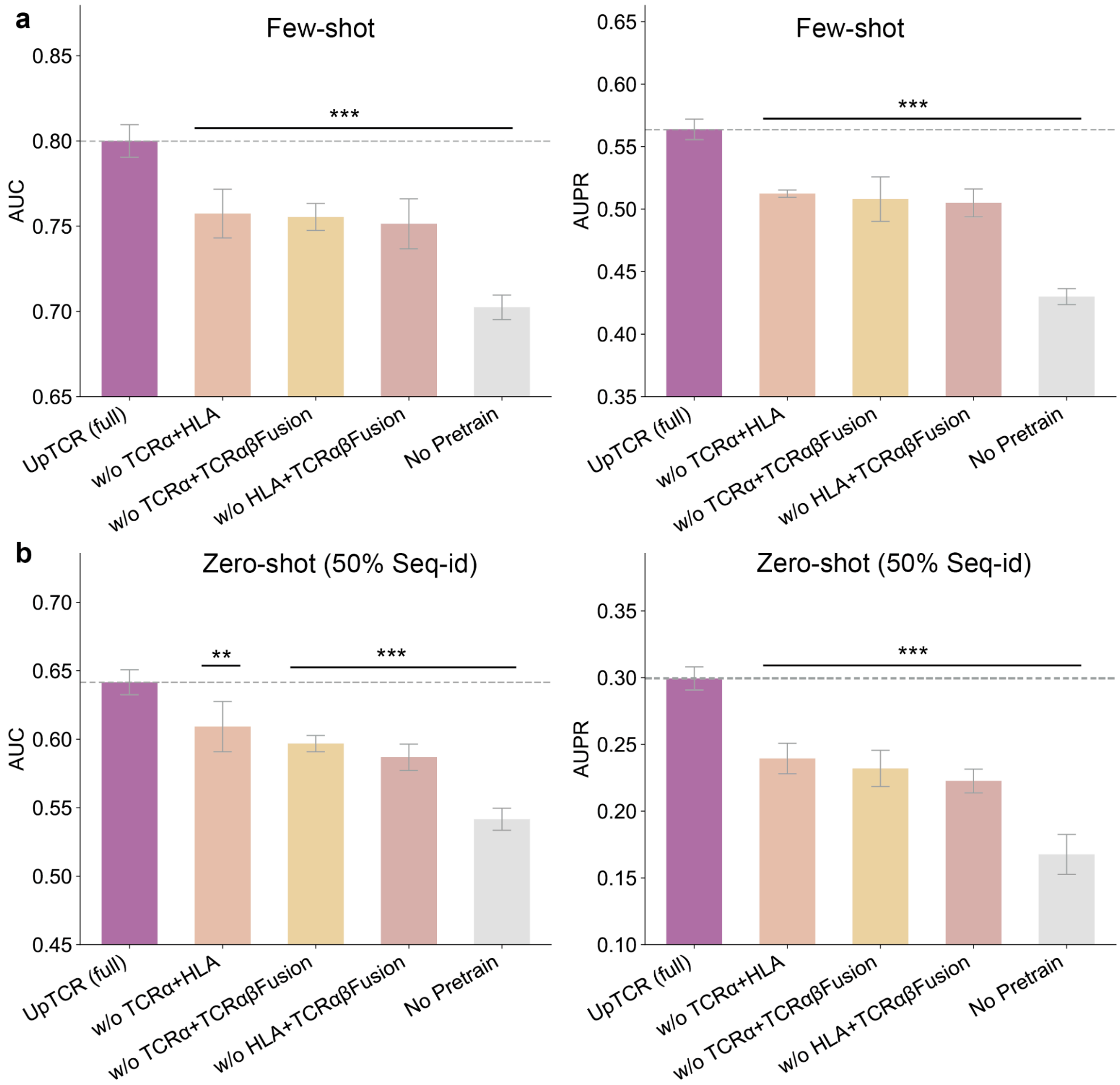

Figure S19. Multi-component pretraining ablation revealed synergistic contributions of individually non-significant modules. a, *AUC (left) and AUPR (right) for TCR-antigen-HLA binding specificity prediction under the few-shot setting when pairs of individually non-significant pretrained modules (TCRαformer, HLAformer, and TCRαβFusionformer) are simultaneously ablated, alongside a fully non-pretrained variant..* ***b,*** *Same analysis under the zero-shot setting (50% sequence identity filtering). The significant decline across all pairwise ablation*

*combinations demonstrated that the progressive pretraining modules function synergistically. * P < 0.05, ** P < 0.01, *** P < 0.001.*

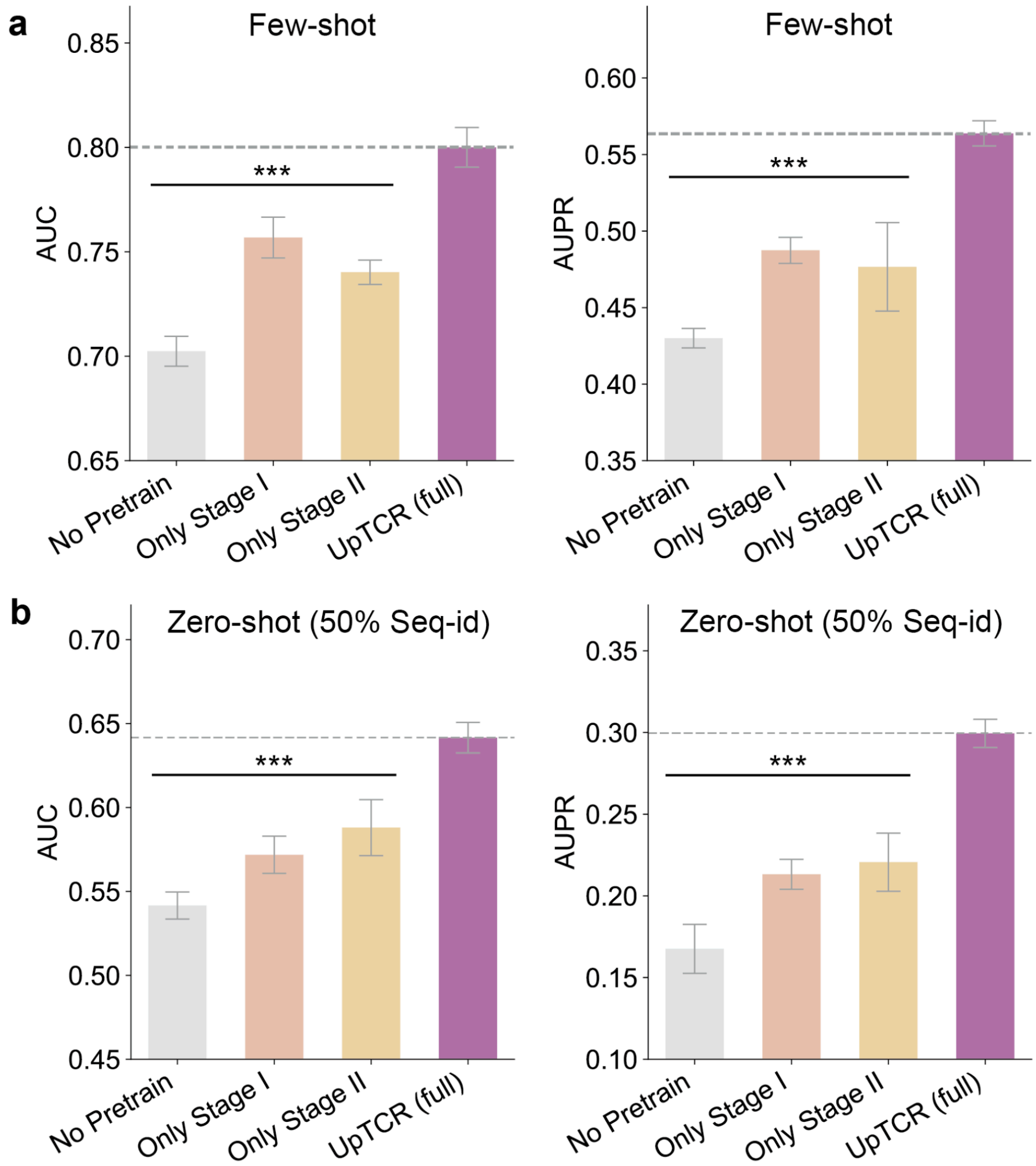

Figure S20. Stage-wise pretraining ablation demonstrated the contributions of both pretraining stages. a, *AUC (left) and AUPR (right) for TCR-antigen-HLA binding specificity prediction under the few-shot setting across four pretraining configurations: No Pretrain (all encoders randomly initialized), Only Stage I (dimer-level pretraining only), Only Stage II (trimer-level pretraining only), and UpTCR (full progressive pretraining).* ***b,*** *Same analysis under the zero-shot setting (50% sequence identity filtering). *** P < 0.001.*

Comment 8)

Similarly, Fig 3A, when various modularities are ablated, it seems that some/most(?) modules can be removed without affecting performance (zero shot cases). It is not clear because for some reason these comparisons are not shown (i.e. the statistical significance of difference between UpTCR and

the rest of various ablated UpTCR is not shown, just the significance of differences are shown among the rest).Discussed in (313-345)

Response: We thank the reviewer for pointing out this omission. We agree that the statistical significance between the full UpTCR model and each modality-ablated variant should have been explicitly presented.

In the original Figure 3a,b, we only displayed significance tests between the ablated UpTCR variants and the baselines, but did not include the comparison between the full UpTCR and each ablated variant. We have now computed and added these missing significance tests to the revised Figure 3a,b. The results show that the complete UpTCR achieves statistically significant improvements over the modality-ablated variants in both few-shot and zero-shot settings. These results demonstrate that incorporating complete TCRα and HLA information provides a statistically significant additional benefit.

The above clarification has been added in the revised manuscript and is presented below.

Results (page 13):

In the few-shot setting, the variants achieved AUCs of 0.784 (w/o TCRα), 0.765 (w/o HLA), and 0.748 (w/o both), representing declines of 2.0%–6.5% relative to the complete model (AUC 0.800, $P < 0.01$). In the zero-shot setting, the variants achieved AUCs of 0.598 (w/o TCRα), 0.603 (w/o HLA), and 0.585 (w/o both), representing declines of 6.1%–8.9% relative to the complete model (AUC 0.642, $P < 0.01$). This finding underscores the importance of incorporating TCRα and HLA molecule information for a comprehensive understanding of TCR-antigen-HLA interactions.

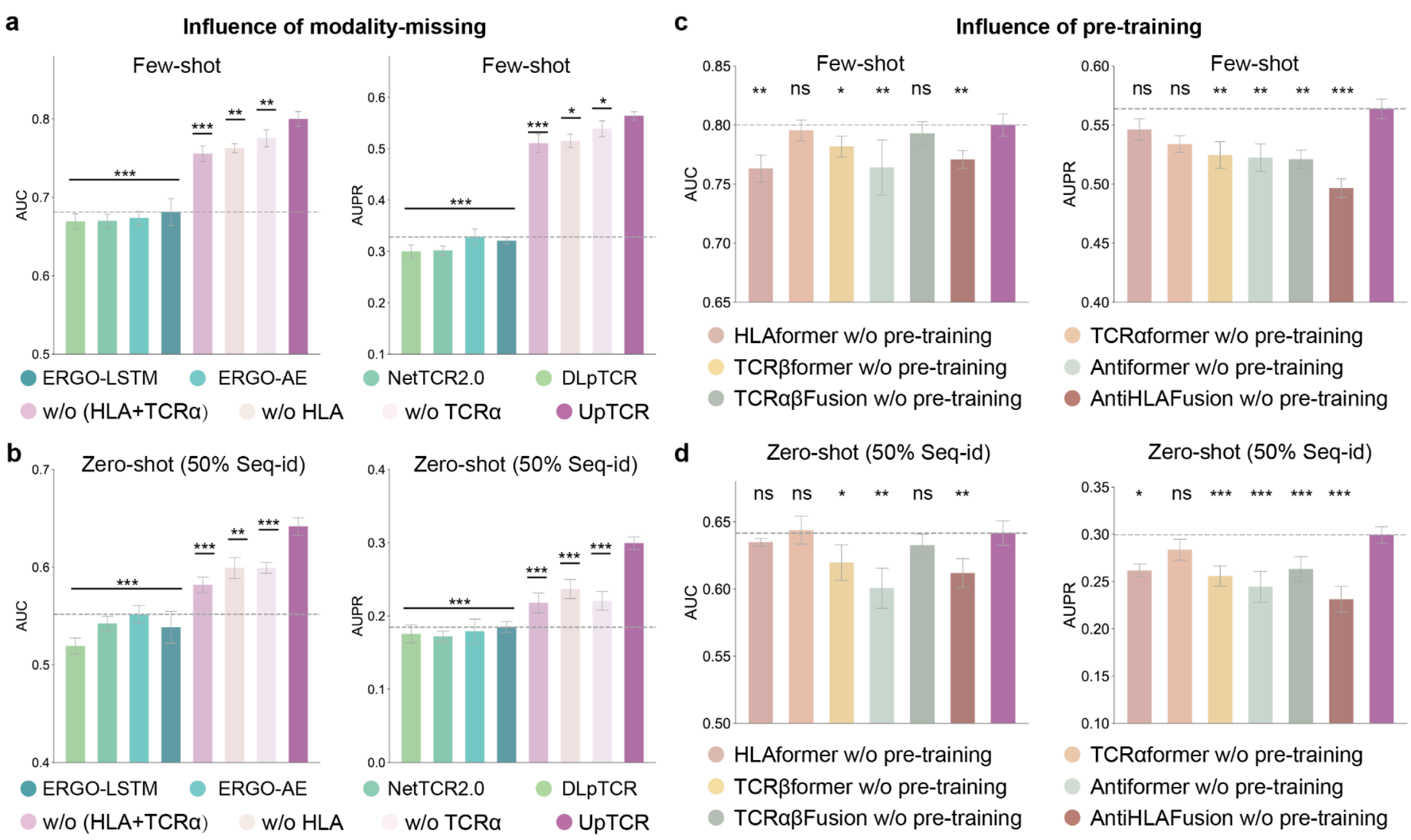

Figure 3. UpTCR achieves superior performance in modality-missing TCR specificity prediction and antigen-HLA affinity prediction. a,b, *Bar plots of AUC (left) and AUPR (right) when several modalities are missing for predicting TCR-antigen-HLA binding specificity under few-shot (**a**) and zero-shot (**b**) settings. Even when both HLA and TCRα are missing, UpTCR still significantly outperforms baselines. The bars represent the average metrics and the error bars are the standard deviations.* ***c,d,*** *Bar plots of AUC (left) and AUPR (right) when the pretrained modules were replaced with non-pretrained versions for predicting TCR-antigen-HLA binding specificity under few-shot (**c**) and zero-shot (**d**) settings. The bars represent the average metrics and the error bars indicate the standard deviations. Statistical significance was calculated between all variants and the UpTCR using paired Student's t-test…*

Comment 9)

Figure 5 and corresponding discussion is not very insightful. Would be more useful if the # of or % of false positives were also presented. It is unclear even in case of "high predicted binding probability" how many more false positive were also predicted. A particular useful analysis would mix true positives with false positive antigens using the same "Levenshtein Distance".

Response: We thank the reviewer for this constructive suggestion. We agree that the original Figure 5 lacked information about false positives, making it difficult to assess the model's discriminative boundary. We have substantially revised this analysis.

In the revised Figure 5a,f, we now present predicted binding probabilities for both confirmed cross-reactive antigens (positives) and non-binding antigens (negatives) side by side for each of the 20 TCR groups, with statistical significance assessed by Mann-Whitney U test. In the high-conservation group, the mean predicted probability for binding antigens was 0.927 versus 0.245 for non-binding antigens (3.79-fold difference), yielding an average classification accuracy of 90.4% at the 0.5 threshold ($P < 0.001$ for all 10 groups; Figure 5a). In the low-conservation group, binding antigens received a mean score of 0.779 versus 0.335 for negatives (2.33-fold difference), with an accuracy of 74.3% ($P < 0.05$ for all 10 groups; Figure 5f).

Regarding the reviewer's suggestion to use negatives with the same Levenshtein distance as the positives: we carefully considered this approach, as it would indeed provide a more stringent test of the model's discriminative boundary. However, its implementation poses a fundamental challenge for the high-conservation groups. In these groups, the confirmed cross-reactive antigens themselves share very small Levenshtein distances (often ≤ 2). Consequently, peptides at similarly small edit

distances are likely to retain binding capacity to the same TCR, making their assignment as 'negatives' unreliable and potentially introducing false-negative labeling. Given the absence of experimentally confirmed non-binding sequences at such close edit distances in the current literature, we instead constructed negatives by selecting peptides presented by the same HLA allele with a Levenshtein distance > 3 from all confirmed binding antigens, ensuring higher confidence in their non-binding status.

The above results have been added in the revised manuscript as follows:

Results (page 21):

To investigate the ability of UpTCR to predict TCR cross-reactivity, we analyzed its performance in identifying cases where a single TCR binds to multiple antigens. Accurate prediction of TCR cross-reactivity is critical for informing therapeutic strategies and enhancing our understanding of immune responses. In this study, we collected data on 20 groups of TCR cross-reactivity (see Methods, Supplementary Table S1), with each TCR binding to multiple different antigens. For each TCR group, we additionally sampled the same number of non-binding antigens as negative controls to assess the model's discriminative power.

…

In the high-conservation group, UpTCR significantly discriminated against cross-reactive binding antigens from non-binding antigens across all 10 TCR groups ($P < 0.001$ for all comparisons, Figure 5a). The mean predicted binding probability for confirmed cross-reactive antigens was 0.927, compared with 0.245 for non-binding antigens (a 3.79-fold difference), yielding an average classification accuracy of 90.4% at the 0.5 decision threshold. This high success rate may be attributed to the presence of highly conserved residues among the antigens (Figure 5b,c), which often exhibit significant sequence homology (Figure 5d,e).

…

In the low-conservation group, UpTCR also achieved significant discrimination for all 10 TCR groups ($P < 0.05$, Figure 5f), with a mean predicted probability of 0.779 for binding antigens versus 0.335 for non-binding antigens (2.33-fold difference) and an average accuracy of 74.3%.

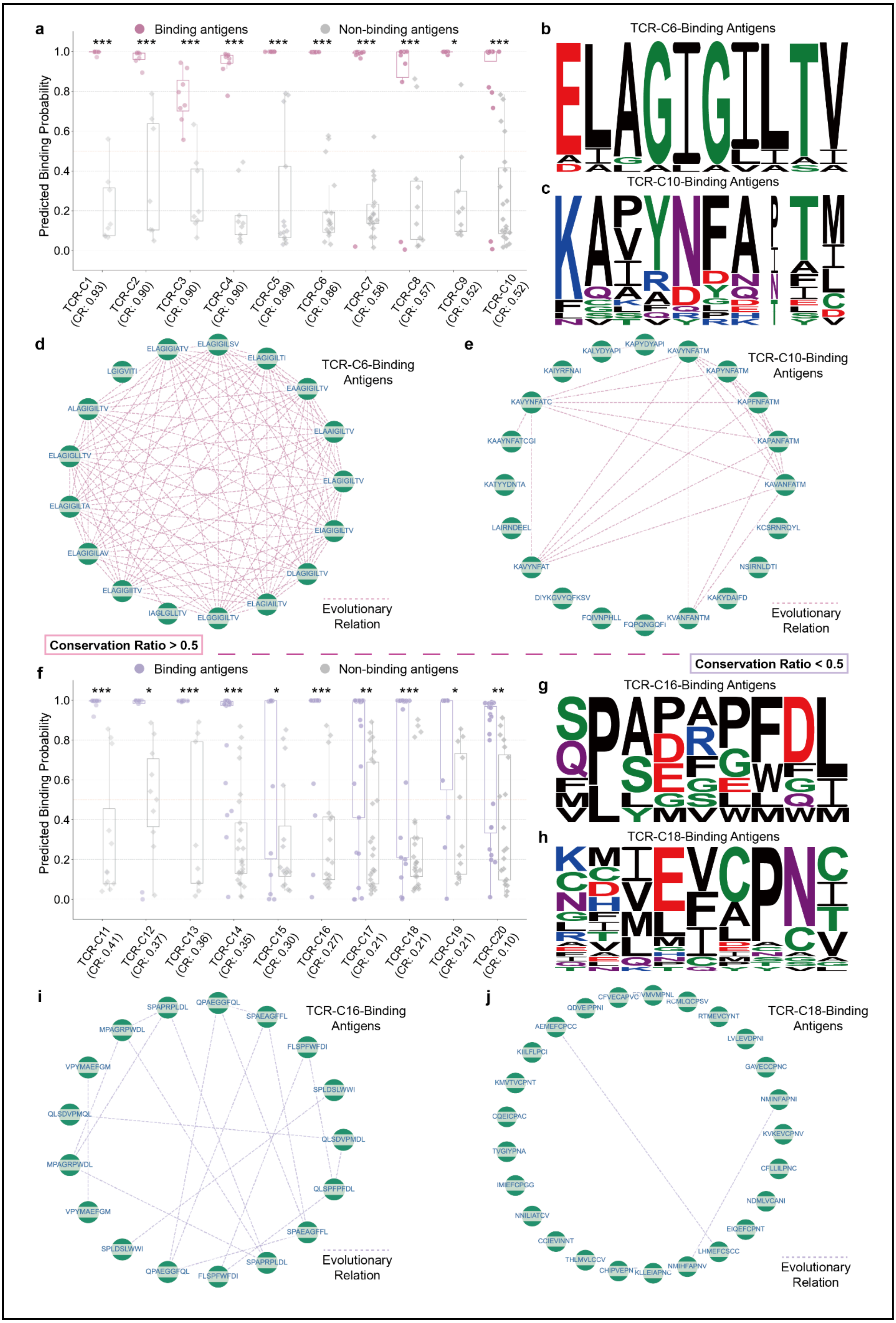
a
Binding antigens
Non-binding antigens
Predicted Binding Probability
*** *** *** *** *** *** *** *** * ***
TCR-C1 (CR: 0.93)
TCR-C2 (CR: 0.90)
TCR-C3 (CR: 0.90)
TCR-C4 (CR: 0.90)
TCR-C5 (CR: 0.89)
TCR-C6 (CR: 0.86)
TCR-C7 (CR: 0.58)
TCR-C8 (CR: 0.57)
TCR-C9 (CR: 0.52)
TCR-C10 (CR: 0.52)
b
TCR-C6-Binding Antigens
c
TCR-C10-Binding Antigens
d
TCR-C6-Binding Antigens
Evolutionary Relation
e
TCR-C10-Binding Antigens
Evolutionary Relation
Conservation Ratio > 0.5
Conservation Ratio < 0.5
f
Binding antigens
Non-binding antigens
Predicted Binding Probability
*** * *** *** * *** ** *** * **
TCR-C11 (CR: 0.41)
TCR-C12 (CR: 0.37)
TCR-C13 (CR: 0.36)
TCR-C14 (CR: 0.35)
TCR-C15 (CR: 0.30)
TCR-C16 (CR: 0.27)
TCR-C17 (CR: 0.21)
TCR-C18 (CR: 0.21)
TCR-C19 (CR: 0.21)
TCR-C20 (CR: 0.10)
g
TCR-C16-Binding Antigens
h
TCR-C18-Binding Antigens
i
TCR-C16-Binding Antigens
Evolutionary Relation
j
TCR-C18-Binding Antigens
Evolutionary Relation

Figure 5. UpTCR is capable of investigating TCR cross-reactivity. ***a,*** *Predicted binding probabilities for 10 cross-reactive TCR groups (TCR-C1 to TCR-C10) in the high-conservation group (conservation ratio > 0.5). For each TCR, pink dots represent experimentally confirmed cross-reactive antigens (binding) and gray diamonds represent non-binding antigens. The conservation ratio for each group is indicated in parentheses. The red dashed line denotes the 0.5 decision threshold.* ***b,c,*** *Sequence motifs of the antigens targeting TCR-C6 (**b**) and TCR-C10 (**c**).* ***d,e,*** *Sequence homology networks for antigens targeting TCR-C6 (d) and TCR-C10 (e). Nodes represent individual antigen sequences and edges connect sequences with a Levenshtein distance < 3, indicating high sequence similarity.* ***f,*** *Predicted binding probabilities for 10 cross-reactive TCR groups in the low-conservation group. Purple dots represent confirmed cross-reactive antigens and gray diamonds represent non-binding antigens.* ***g,h,*** *Sequence motifs of the cross-reactive antigens targeting TCR-C16 (**g**) and TCR-C18 (**h**).* ***i,j,*** *Sequence homology networks for antigens targeting TCR-C16 (i) and TCR-C18 (j). The sparse connectivity in the low-conservation group reflects high sequence diversity among cross-reactive antigens. *P < 0.05, **P < 0.01, ***P < 0.001.*

Comment 10)

The perspective validation of predicted peptides for MELAN-10: details are not provided. How were the variants generated? How many? And was MELAN-10 part of the original training of the TCR-ag-HLA system or any similar peptides? This is because among all the enumerated peptides for MELAN-10: (table S2), the 3 high affinity peptides identified have 9/10, 9/10 and 9/9 identical matches to MELAN-10, while the low affinity one has 6/9 identical residues. A simple sequence comparison would end up with similar conclusion. However, it would be impressive if MELAN -10 or similar peptides were not part of the training.

In addition, the MELAN-10 peptide should be included in tables S2. I had to look it up from the literature to do the above assessment of sequences.

Response: We thank the reviewer for this detailed and important question. We address each point and have substantially expanded both the experiment and its documentation.

1) Variant generation. Starting from the native Melan-A decamer (EAAGIGILTV), we constructed a comprehensive in silico variant library by systematically introducing amino acid substitutions, insertions, and deletions. For edit distances 1-2, where the variant space is tractable, all possible variants were exhaustively enumerated (~50,000 variants). For edit distances 3-5, where the

combinatorial space grows to millions or more, we uniformly sampled 20,000 variants per level. This yielded a total library of approximately 110,000 candidate variants spanning diverse peptide lengths (8–12 residues) and sequence identities (40%–90%). For each candidate, we predicted the binding probability to the cognate TCR using UpTCR and ranked the variants by predicted score.

2) Training-set independence. We confirmed that neither the native Melan-A peptide (EAAGIGILTV) nor the cognate TCR sequence was present in the training data used for this prospective experiment. Furthermore, any peptides or TCR CDRs sharing greater than 30% sequence identity with the test sequences were removed during our de-redundancy filtering. The predictions therefore reflect genuine generalization rather than memorization of training examples.

3) Addressing sequence similarity and expanding to low-identity variants. We fully acknowledge the reviewer's important point that the three originally selected high-binding variants (80% identity) closely resemble the canonical epitope, and that a simple sequence comparison might yield a similar ranking for such near-identical peptides. To provide a substantially more compelling demonstration, we expanded the experimentally validated variant set to include five additional peptides with considerably lower identity to the native epitope (40%–54.5%; Table S2). UpTCR predicted all eight variants as high-binding, and experimental validation confirmed T-cell activation across the full identity range (Figure 8b,c). Critically, the results directly refute a simple sequence-similarity explanation: AntigenLV shares 60% identity with the native peptide yet was predicted as non-binding and experimentally confirmed as non-activating, whereas AHV7 and AHV8 share only 40% identity yet were predicted as high-binding and elicited robust T-cell responses. This dissociation between sequence identity and binding prediction demonstrates that UpTCR captures biophysical determinants of TCR recognition beyond trivial sequence similarity.

4) Table S2. The revised Table S2 now includes the native Melan-A peptide sequence alongside all tested variants, annotated with their sequence identity to the native epitope.

To be clearer, the revised content is presented below.

Results - (page 31-32):

Starting from the native Melan-A decamer (EAAGIGILTV), we constructed a comprehensive *in silico* variant library by systematically introducing amino acid substitutions, insertions, and deletions across peptide positions, thereby generating variants spanning a range of peptide lengths and sequence identities. For edit distances 1-2, where the combinatorial space is tractable, all possible variants were exhaustively enumerated. For edit distances 3-5, we uniformly sampled 20,000 variants per level to ensure broad coverage while maintaining computational tractability,

yielding a total library of approximately 110,000 variants. For each candidate, we predicted the binding probability using UpTCR and ranked the variants by predicted score. From the ranked list, we selected eight predicted high-binding variants (AHV1-AHV8) spanning a wide range of sequence identity to the native peptide (40%-80%) and one predicted low-binding variant (AntigenLV, 60% identity) for experimental validation (Table S2). Notably, several predicted high-binding variants (AHV4-AHV8) share substantially lower sequence identity (40%-54.5%) with the native epitope, providing a stringent evaluation of the model's generalization capability.

…

Consistent with the computational predictions, the predicted high-binding variants AHV1-AHV8 induced higher IL-2 secretion than the predicted low-binding variant AntigenLV and unrelated peptide controls (Figure 8b). Similarly, flow cytometry analysis showed an increased proportion of CD69+ cells among CD8+ T cells upon stimulation with AHV1-AHV8, whereas AntigenLV, unrelated peptide, and DMSO controls induced minimal activation (Figure 8c,d). Importantly, the low-identity variants also elicited T cell activation, supporting the ability of UpTCR to identify TCR-recognized peptide variants beyond near-canonical sequence similarity.

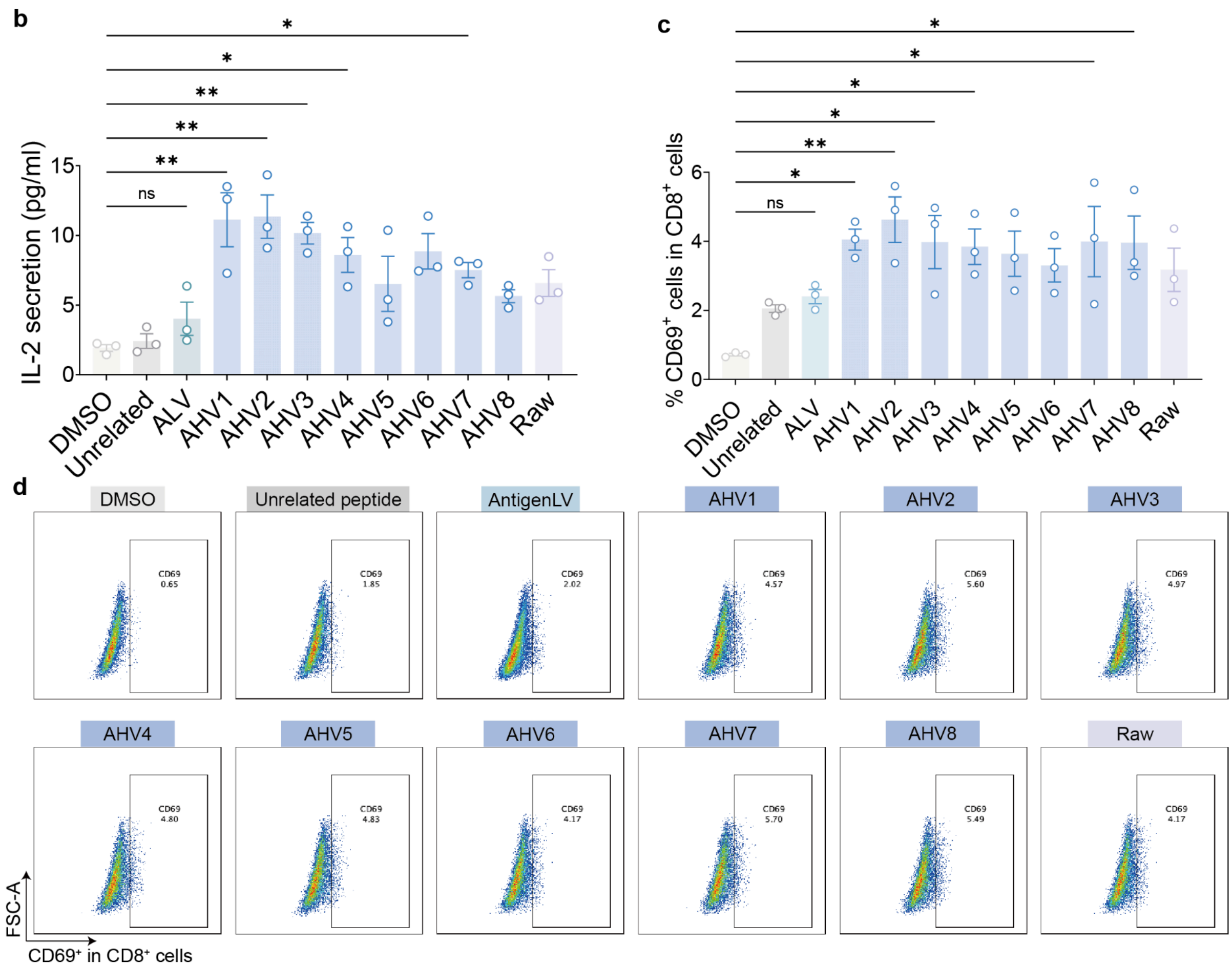

Figure 8.* … *b,* *IL-2 secretion measured by ELISA after 24 h stimulation with DMSO, unrelated peptide, AntigenLV, AHV1–AHV8, or the native peptide (Raw) (n = 3 independent batches).* *c,

Frequency of CD69⁺ cells among CD8⁺ T cells measured by flow cytometry after 24 h stimulation with the indicated peptides or controls (n = 3 independent batches). ***d,*** *Representative flow cytometry plots showing CD69 expression in CD8⁺ T cells following stimulation with DMSO, unrelated peptide, AntigenLV, AHV1–AHV8, or the native peptide (Raw). Data are presented as mean ± SD. Statistical significance was assessed by one-way ANOVA with multiple-comparison correction (**b-c**). * P < 0.05, ** P < 0.01, *** P < 0.001, **** P < 0.0001, ns indicates no significant difference.*

Table S2. Screened antigen variants in the prospective study.

Antigen	Sequence	Sequence identity to raw
Raw sequence (MELAN-10)	EAAGIGILTV	-
AntigenHV1	ELAGIAILTV	80%
AntigenHV2	ELAGIGILAV	80%
AntigenHV3	AAGIGILTVI	80%
AntigenHV4	FLAGGIGIRTL	54.5%
AntigenHV5	ALGGLLTMV	50%
AntigenHV6	SMLGIGIVPV	50%
AntigenHV7	LGIGVITI	40%
AntigenHV8	SLWLGILSV	40%
AntigenLV	SMAGIGIVDV	60%

Comment 11)

Fig 8 panel 3, was there any repeats of flow cytometry ? The legends states "representative". If more were done the average and std can be provided with p- values. The differences appear to be small by the single one example provided.

Response: We thank the reviewer for this point regarding statistical rigor. The flow cytometry experiments were performed in 3 independent batches.

In the revised Figure 8, we now present: (1) the quantification of CD69⁺ frequency among CD8⁺ T cells as mean ± SD across the three independent batches (Figure 8c), with statistical significance

assessed by one-way ANOVA with multiple-comparison correction; and (2) representative flow cytometry plots for visual reference (Figure 8d). The statistical analysis confirms that all predicted high-binding variants (AHV1–AHV8) induce significantly greater CD69^{+} frequencies compared with negative controls ($P < 0.05$), whereas AntigenLV shows no significant activation.

To be clearer, the revised content is presented below.

Results - (page 31-32):

Consistent with the computational predictions, the predicted high-binding variants AHV1-AHV8 induced higher IL-2 secretion than the predicted low-binding variant AntigenLV and unrelated peptide controls (Figure 8b). Similarly, flow cytometry analysis showed an increased proportion of CD69+ cells among CD8+ T cells upon stimulation with AHV1-AHV8, whereas AntigenLV, unrelated peptide, and DMSO controls induced minimal activation (Figure 8c,d). Importantly, the low-identity variants also elicited T cell activation, supporting the ability of UpTCR to identify TCR-recognized peptide variants beyond near-canonical sequence similarity.

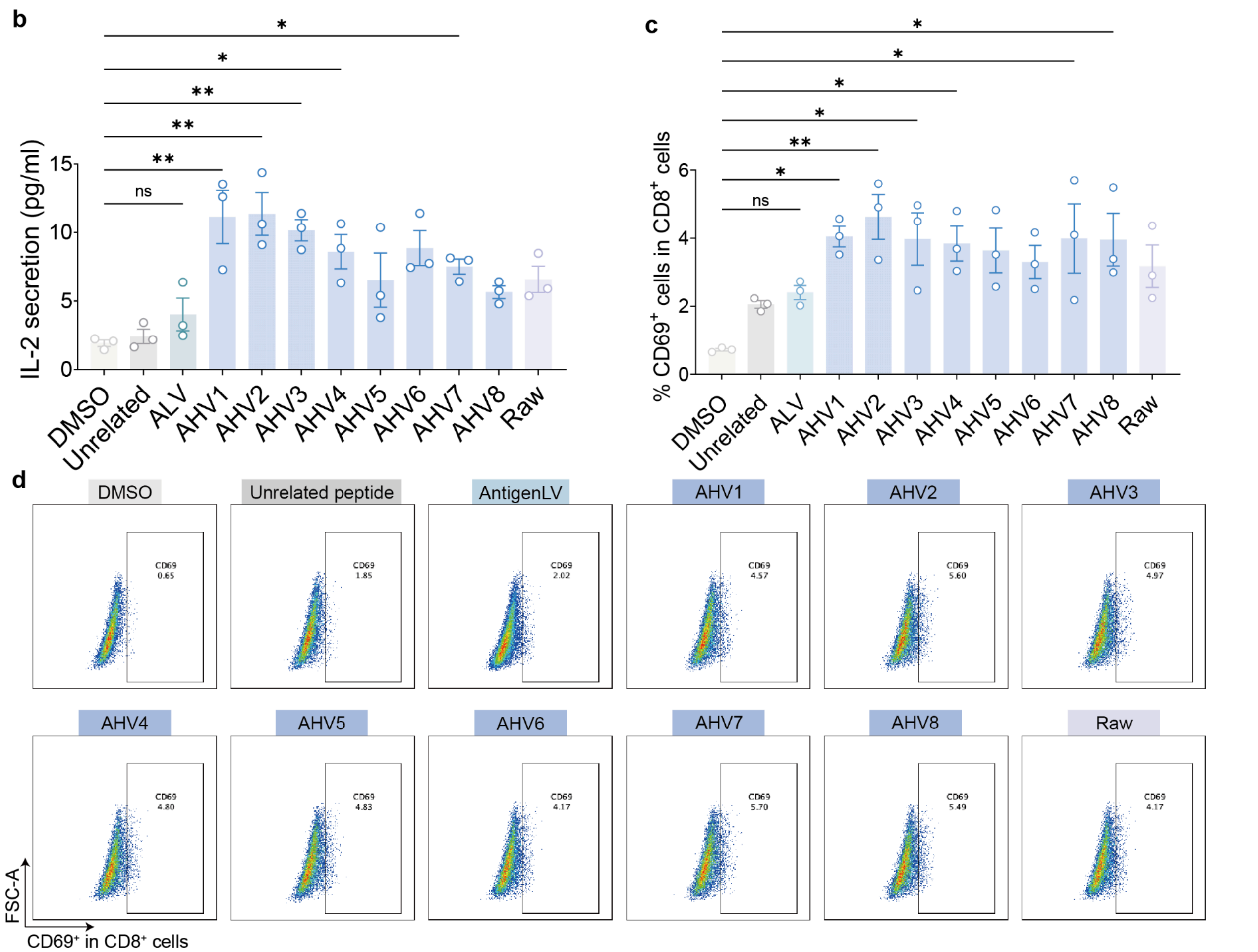

Figure 8. *… **b,** IL-2 secretion measured by ELISA after 24 h stimulation with DMSO, unrelated peptide, AntigenLV, AHV1–AHV8, or the native peptide (Raw) (n = 3 independent batches). **c,***

Frequency of $CD69^{+}$ cells among $CD8^{+}$ T cells measured by flow cytometry after 24 h stimulation with the indicated peptides or controls (n = 3 independent batches). ***d,*** *Representative flow cytometry plots showing CD69 expression in $CD8^{+}$ T cells following stimulation with DMSO, unrelated peptide, AntigenLV, AHV1–AHV8, or the native peptide (Raw). Data are presented as mean ± SD. Statistical significance was assessed by one-way ANOVA with multiple-comparison correction (**b-c**). * P < 0.05, ** P < 0.01, *** P < 0.001, **** P < 0.0001, ns indicates no significant difference.*

Comment 12)

Contact map prediction Fig 6. Some of the contacts are very conserved especially with HLA-Antigen, and in can case of TCR -antigen it is also similar. Therefore the quality of contact map prediction needs to be put in some context, compared to some other references. Otherwise it is very difficult to make a quantitative sense of these spectrum maps .

Response: We thank the reviewer for this important point. We agree that the original contact map evaluation relied solely on binary classification metrics (AUC), which may partially reflect conserved interaction patterns. To provide a more rigorous quantitative context, we have made the following additions in the revised manuscript:

To provide a more rigorous quantitative assessment, we now report Pearson correlation coefficients for continuous inter-residue distance prediction in addition to binary contact AUC. Unlike AUC, Pearson correlation directly evaluates the accuracy of predicted spatial distances and is less susceptible to inflation by conserved contact patterns. Notably, UpTCR consistently and significantly outperformed TEIM-Res across all six pairwise interaction types (*P* < 0.05, Supplementary Figure S22). Importantly, the improvement was most pronounced for the TCRα-TCRβ interaction (Pearson: TEIM-Res 0.705 vs. UpTCR 0.860 vs. UpTCR+AF 0.873), which represents the most structurally variable interface across different complexes.

The above revised content has been properly added to the revised manuscript according to the reviewer's suggestions, which is presented as below.

Results (page 24):

We evaluated the contact prediction performance using both binary classification (AUC) and continuous distance regression (Pearson correlation) metrics. In cross-validation on the 164 crystal

structures, UpTCR significantly outperformed TEIM-Res[1] across all six pairwise interaction types in both AUC (*P* < 0.05, Figure 6a) and Pearson correlation (*P* < 0.05, Supplementary Figure S22). UpTCR+AF further improved upon UpTCR in most interaction types, demonstrating the benefit of augmenting the limited crystal structure data with high-confidence computational models. Notably, the improvement was most pronounced for TCRα-TCRβ contact map predictions (Pearson: TEIM-Res 0.705 versus UpTCR 0.860 versus UpTCR+AF 0.873), reflecting the capacity of additional structural data to capture the complex chain-pairing interface.

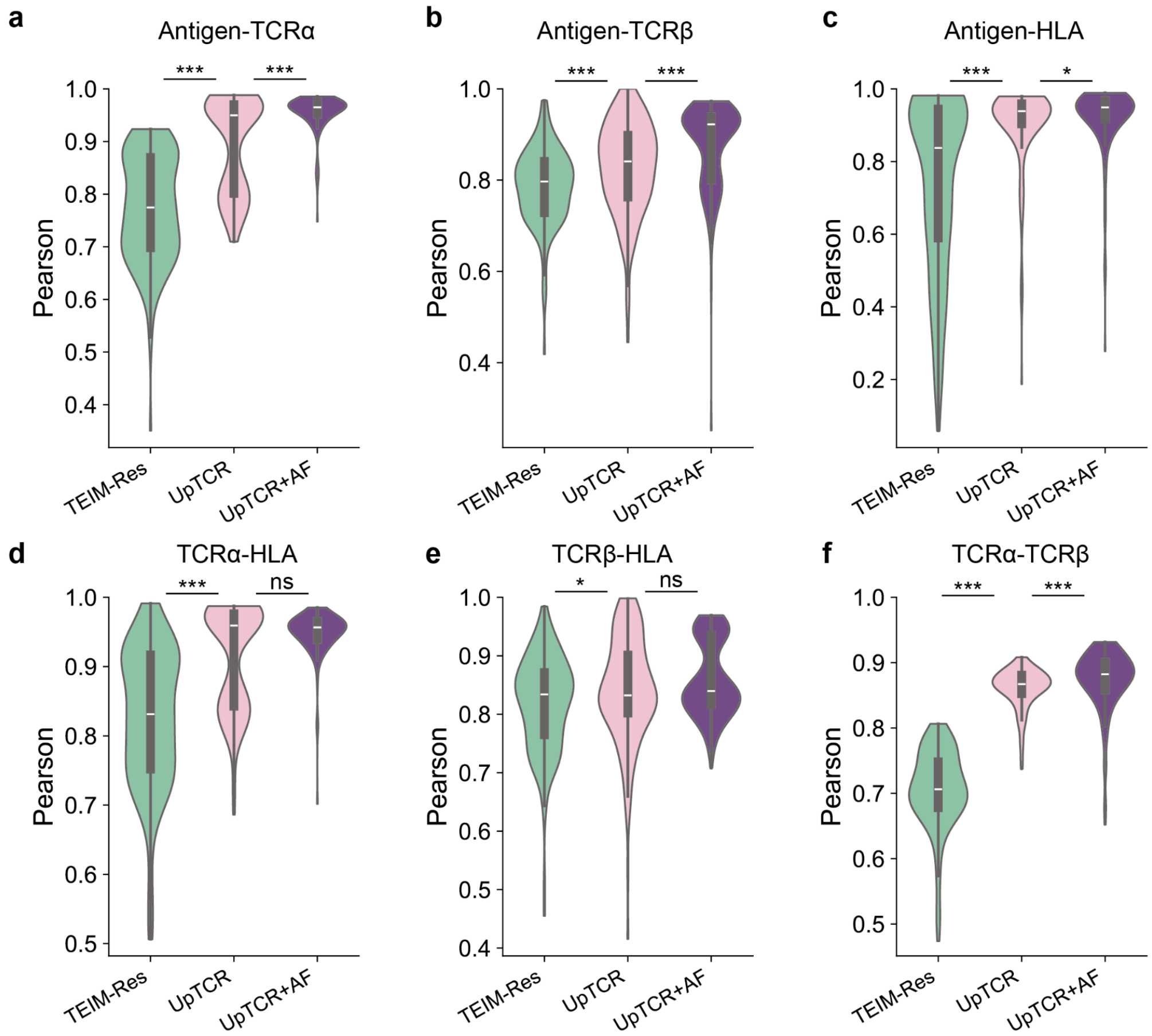

Figure S22. Pearson correlation for residue-level distance prediction across all pairwise interactions. a-b, *Violin plots displaying the Pearson correlation coefficients between predicted and ground-truth residue-pair distances for TEIM-Res, UpTCR, and UpTCR+AF across six pairwise interaction types. From left to right, top row: antigen-TCRα, antigen-TCRβ, antigen-HLA; bottom row: TCRα-HLA, TCRβ-HLA, and TCRα-TCRβ. UpTCR+AF denotes UpTCR further trained with high-confidence AlphaFold-predicted complex structures. UpTCR and UpTCR+AF consistently and significantly achieve higher Pearson correlations than TEIM-Res, demonstrating superior accuracy in predicting inter-residue distances. * P < 0.05, ** P < 0.01, *** P < 0.001, ns indicates no significant difference.*

Comment 13)

Repetition (repeated paragraphs) of the same arguments, same references: lines 70-77 and 78-84

Repetition of testing concepts: 244-247 and 258-260

Response: We thank the reviewer for identifying these redundancies. We have carefully revised the manuscript to eliminate the repetitive content:

(1) Lines 70-77 and 78-84: These passages contained overlapping discussions of the challenges in TCR-antigen-HLA modeling. We have consolidated these two paragraphs into a single, streamlined paragraph that presents each challenge and its corresponding motivation only once, avoiding repeated argumentation and references.

(2) Lines 244-247 and 258-260: These passages repeated similar descriptions of the evaluation settings. We have merged the text to present each testing concept only once.

We have also performed a thorough review of the entire manuscript to identify and remove additional repetitive content, ensuring a more concise and readable presentation. To be clearer, the revised content is presented below.

Introduction (page 3-4):

However, the binding of peptides to HLA is only the initial phase in tumor-specific immune responses. To achieve a comprehensive understanding of this process, it is essential to consider TCR-antigen binding recognition, as exemplified by tools such as NetTCR[2], ERGO[3], DLpTCR[4], TITAN[5], PanPep[6], and TEIM-Res[1]. More recently, a handful of approaches, such as pMTnet[7] and UnifyImmun[8], simultaneously consider antigen-HLA binding and TCR-antigen binding, leading to improved results in pairwise binding prediction. Furthermore, studies such as PISTE[9] and EPACT[10] have been proposed to directly predict TCR-antigen-HLA interactions, a more challenging task due to the complex interaction landscape and extensive molecular diversities[11].

Results (page 10):

We evaluated UpTCR's predictive performance in predicting TCR-antigen-HLA recognition across four distinct settings: majority, few-shot, zero-shot with 50% sequence identity filtering, and zero-shot with 30% sequence identity filtering. For this evaluation, binding specificity is defined as a binary classification task, where a label of 1 indicates a positive binding event and 0 indicates non-binding.

Comment 14)

The Results section provides excessive qualitative discussion about the trained system (incl. Fig 1), which could be drastically reduced. Qualitative discussions should be replaced with the actual performance numbers e.g. (lines 267-275 and 276-280 320-345 etc. "significantly outperformed" "best performing" " significantly improved" "certain degree of performance decline" "most significant impact on performance" (?) Meanwhile it is mentioned 7 times that a p-value significance was used at 0.05 cutoff, which is understood but unnecessarily cited so many times.

Response: We thank the reviewer for this constructive feedback on the writing style. We agree that the Results section relied too heavily on qualitative descriptors and would benefit from more precise quantitative reporting.

In the revised manuscript, we have made the following changes:

(1) Streamlined the description of the model architecture in the Results section. The qualitative discussion around Figure 1 has been condensed to focus on the key design principles, with technical details deferred to the Methods section.

(2) Replaced vague qualitative statements with specific performance numbers. For example, statements such as "significantly outperformed" and "best performing" have been supplemented with concrete AUC/AUPR values and percentage improvements.

(3) Reduced redundant mentions of statistical testing. The p-value threshold ($P < 0.05$) is now stated in the Methods section and Figure legends and referenced concisely in the Results without repeated citation of the testing procedure.

These revisions have been applied throughout the Results section to ensure a more concise, data-driven presentation. The specific revised text can be found on pages 6-7 (model description), page 10 (benchmark results), and page 13 (modality-missing analysis) of the revised manuscript.

To be clearer, some revised content is presented below.

Results (page 6-7):

UpTCR employs a modular divide-merge architecture to model TCRαβ-antigen-HLA interactions (Figure 1). Individual encoders first capture the distinct properties of TCRα, TCRβ, antigen, and HLA. Pairwise fusion encoders then integrate TCRαβ and antigen-HLA complexes, and finally, the unified tetramer representation is used for downstream prediction. Specifically, UpTCR

comprises monomer modeling encoders (TCRαformer, TCRβformer, Antiformer, and HLAformer), dimer complex fusion encoders (TCRαβFusionformer and AntiHLAFusionformer), and task-specific finetuning modules (Figure 1a).

Results (page 10):

In the majority setting, where the majority of relevant data for each antigen is present in the training set, UpTCR achieved an AUC (Area Under the Curve) of 0.905 and an AUPR (Area Under the Precision-Recall Curve) of 0.749, outperforming all baselines (Figure 2a,e). In the few-shot setting, where only a limited number of training samples are available for each antigen, UpTCR attained an AUC of 0.800 and AUPR of 0.561, outperforming the next-best method (EPACT) by 3.6% in AUC and 8.1% in AUPR (Figure 2b,f). We then assessed UpTCR in two zero-shot settings of increasing stringency, where none of the antigens in the test set appeared in the training data. Under 50% sequence identity filtering, UpTCR achieved an AUC of 0.642 and AUPR of 0.296, exceeding the next-best baseline by 6.5% in AUC (Figure 2c,g). Under a more stringent 30% threshold, UpTCR maintained the highest AUC of 0.594 and AUPR of 0.252, outperforming all baselines ($P < 0.001$, Figure 2d,h). The corresponding ROC and PR curves across all four settings (Figure 2i-p) further corroborated these findings across all four settings.

Results (page 13):

In the few-shot setting, the variants achieved AUCs of 0.784 (w/o TCRα), 0.765 (w/o HLA), and 0.748 (w/o both), representing declines of 2.0%–6.5% relative to the complete model (AUC 0.800, $P < 0.01$). In the zero-shot setting, the variants achieved AUCs of 0.598 (w/o TCRα), 0.603 (w/o HLA), and 0.585 (w/o both), representing declines of 6.1%–8.9% relative to the complete model (AUC 0.642, $P < 0.01$). This finding underscores the importance of incorporating TCRα and HLA molecule information for a comprehensive understanding of TCR-antigen-HLA interactions.

Reviewer #2 (Remarks on code availability):

Comment 15)

The Github deposit needs better organization:

It took more than 6 hours for data download at Gbit speed connection.

Response: We sincerely thank the reviewer for pointing out this critical issue, and we apologize for the inconvenience and frustration caused by the sub-optimal data organization and the slow

download speed. To fully resolve this issue and improve data accessibility, we have implemented the following major modifications:

To prevent network-induced transmission failures and support resumed downloads, we have utilized the 7z solid-compression algorithm to partition the full 41 GiB dataset into 4 structured volumes, hosted at Hugging Face (https://huggingface.co/datasets/DDDead/Uptcr_data) for high-speed access. Reviewers can now download these chunks concurrently or sequentially. A clear step-by-step decompression guide has been explicitly provided in our GitHub README.md.

We also note that due to the massive sheer volume of the native deep learning files, downloading the full 41 GiB dataset may still take some time depending on peak network routing.

To be clearer, the revised content is presented below.

Readme

6. **Download Processed Data:**

The processed data (~41 GiB) includes TCR-antigen-HLA binding data, structure data, and pretrained embeddings. We provide two download options:

Option A: Single archive (for stable network connections)

Download `data.zip` from Zenodo: https://doi.org/10.5281/zenodo.15128399, and extract it to the `UpTCR/data` directory:

```
unzip data.zip -d ./data
```

Option B: Split volumes (for unreliable networks with resume support)

To prevent network-induced transmission failures, we have also partitioned the dataset into 4 volumes (`data.7z.001` to `data.7z.004` , 10 GiB each), hosted at Hugging Face: https://huggingface.co/datasets/DDDead/Uptcr_data, for high-speed access.

Ensure the `p7zip` package is installed on your system. Navigate to your `UpTCR` root directory, move all 4 downloaded files into the `UpTCR/` folder, and run the following command on the **first** volume (the program will automatically detect and merge the remaining parts):

```
7z x data.7z.001 -o./data
```

Directory structure after extraction:

```
UpTCR/data/
├── finetune/        # Processed TCR-antigen-HLA binding data for training
├── structure/       # Processed TCR-antigen-HLA structure data
└── pretrained_emb/  # Pretrained embeddings
```

> **Note:** The `pretrained_emb` directory is large, consisting of 41,599 antigen embeddings, 27,066 TCRa embeddings, and 27,946 TCRb embeddings. Decompression may take a considerable amount of time.

Comment 16)

The program requires an outdated operating linux system (CENTOS 8 years 2020-21) and outdated CUDA environment (CUDA 11), which most users will not have access to anymore.

Response: We thank the reviewer for raising this concern regarding software compatibility. We would like to clarify that UpTCR is fully compatible with modern Linux distributions and CUDA environments. The CentOS 8 and CUDA 11 specifications listed in the repository reflect the development environment in which the software was originally built and tested, but they do not represent strict system requirements. UpTCR runs successfully on current Linux distributions (*e.g.*, Ubuntu 22.04/24.04) and supports CUDA 11.x through 12.x, as the underlying PyTorch framework maintains backward compatibility across these versions.

To avoid confusion, we have updated the repository documentation to explicitly state the supported range of operating systems and CUDA versions, and clarified that the listed environment represents a tested configuration rather than a minimum requirement.

To be clearer, the revised content is presented below.

Readme

1. **Hardware requirements**: This project requires only a standard computer with enough RAM and a NVIDIA GPU to support operations. We ran the demo using the following specs:
    - CPU: 10 cores, 2.5 GHz/core
    - RAM: 40GB
    - GPU: NVIDIA TESLA P40, V100, A100, A6000
    - CUDA: 11.0, 12.0
2. **System requirements**: This tool is supported for Linux. The tool has been tested on the following system:
    - CentOS Linux release 8.2.2.2004
    - Ubuntu 22.04
    - Ubuntu 24.04
3. **Clone the Repository**:

```
git clone https://github.com/tylerlv/UpTCR.git
cd UpTCR
```

4. **Install Required Packages**: The basic environment requirements are:
    - Python: 3.10/3.11
    - CUDA: 11.0/12.0

Comment 17)

After we located a backdated linux and CUDA system we downloaded the package.It is not working as a critical file is missing:

"The file model_weights.zip cannot be found on Zenodo: https://doi.org/10.5281/zenodo.15128399"

Response: We sincerely apologize for this oversight. Upon checking the Zenodo repository, we confirmed that the model weights file was inadvertently omitted during the initial upload. We have now uploaded the complete model weights to the Zenodo repository (https://doi.org/10.5281/zenodo.20520000) and verified that all files listed in the README are accessible. We have also performed a full end-to-end test of the installation and inference pipeline to confirm that the package runs correctly with the uploaded weights. We regret any inconvenience this caused during the review process.

To be clearer, the revised content is presented below.

Readme

5. **Download Model Weights:**

Download the `model_weights.zip` file and extract it to the `UpTCR/model_weights` directory. The model_weights.zip is available on Zenodo: https://doi.org/10.5281/zenodo.20520000

After extraction, the `UpTCR/model_weights` directory should contain the following:

```
UpTCR/model_weights/
├── pretrained/
└── finetune
```

The `UpTCR/model_weights/pretrained` directory contains the pretrained individual encoders and fusion encoders.

The `UpTCR/model_weights/finetune` directory should contain the following:

```
UpTCR/model_weights/finetune/
├── TCRABpMHC_fewshot/
├── TCRABpMHC_unseen/
├── TCRABp_fewshot/
├── TCRABp_unseen/
├── TCRBpMHC_fewshot/
├── TCRBpMHC_unseen/
├── TCRBp_fewshot/
└── TCRBp_unseen/
```

Each sub-directory contains the fine-tuned models for direct test.

Reviewer #3 (Remarks to the Author):

Summary

The authors propose soft contrastive pretraining and multi-scale modelling strategies to predict TCR-peptide-HLA interactions from integrated TCR-peptide-HLA datasets with various missing data. Then, the authors show that this approach can improve the predictions compared to the previously published methods. Finally, the authors show wet lab validations on their TCR prediction algorithm.

Response: We sincerely thank Reviewer #3 for the insightful summary and the positive recognition of our work. We address each of the comments in detail below.

Comment 1)

The authors report that UpTCR predictions align well with a curated cross-reactivity dataset. However, these datasets have assay-dependent definitions of reactivity (e.g., T cell lines vs. primary T cells). To strengthen this claim, the authors should independently validate the predicted cross-reactive peptides for a subset of TCRs.

Response: We thank the reviewer for this important and constructive suggestion. We agree that the curated cross-reactivity dataset aggregates data from heterogeneous experimental systems, which may introduce assay-dependent variations.

To address this concern, we have taken two steps. 1) First, we now acknowledge the assay heterogeneity of the curated cross-reactivity dataset and have revised the wording to avoid over-interpreting this retrospective analysis. 2) Second, we note that we have already performed a controlled prospective validation that is conceptually aligned with the reviewer's suggestion. Specifically, we used UpTCR to predict cross-reactive peptide variants for a well-characterized Melan-A-specific TCR, and then experimentally validated these predictions under a single, unified assay system (Jurkat T cells expressing the cognate TCR, with IL-2 secretion and CD69 expression as readouts). The results confirmed that UpTCR-predicted high-binding variants elicited robust T-cell activation, whereas the predicted low-binding variant did not, providing independent experimental evidence for UpTCR's ability to predict TCR cross-reactivity in a controlled setting.

We acknowledge that extending such prospective validation to multiple TCRs with diverse HLA restrictions and broader peptide panels would further strengthen the conclusions. However,

synthesizing and functionally evaluating multiple TCR constructs and their corresponding peptide panels under standardized conditions represents a substantial experimental undertaking that is beyond the scope of the current revision. We now explicitly discuss both the assay heterogeneity limitation and the need for broader experimental validation in the revised Discussion.

To be clearer, the revised content is presented below.

Discussion (page 35)

We acknowledge that certain limitations remain to be addressed. First, the curated cross-reactivity dataset used in the retrospective analysis aggregates reactivity annotations derived from heterogeneous experimental systems, including T-cell lines and primary T cells, which may introduce assay-dependent biases. Although UpTCR achieved high prediction accuracy in this setting and its cross-reactivity predictions were prospectively validated using a unified experimental assay, extending such controlled validation to a broader panel of TCRs across diverse HLA restrictions and assay platforms remains an important direction for future work.

Comment 2)

It would be informative to assess whether the low-conservation peptides retain measurable binding affinity to their HLA molecules.

Response: We thank the reviewer for this important suggestion. We agree that for low-conservation cross-reactive peptides, it is essential to verify that these peptides retain the capacity to be presented by their cognate HLA molecules.

To directly address this point, we assessed the predicted pMHC binding affinity of the 78 peptides in the low-conservation cross-reactivity groups using NetMHCpan 4.1. Peptides were classified according to standard percentile-rank thresholds: strong binders (SB; rank $\leq$ 0.5%), weak binders (WB; 0.5% < rank $\leq$ 2%), and binders (rank $\leq$ 10%). Of the 78 peptides, 63 (80.8%) were predicted to bind their cognate HLA molecules (Supplementary Figure S25). Notably, the remaining 15 peptides, predominantly cysteine-rich sequences, have been independently confirmed as positive T-cell epitopes restricted to HLA-A*02:01 via experimentally validated T-cell assay records in the IEDB database. This indicates that the prediction discrepancy reflects a known limitation of NetMHCpan for such sequences rather than a genuine absence of HLA presentation.

Collectively, these results confirm that the low-conservation cross-reactive peptides retain HLA presentation capacity, supporting the biological plausibility of our cross-reactivity analysis. We have added this analysis as Supplementary Figure S25 and described the results in the revised manuscript.

Methods (page 39)

To assess whether the low-conservation cross-reactive peptides retain HLA presentation capacity, we evaluated their predicted pMHC binding affinity using NetMHCpan 4.1. Of the 78 peptides in the low-conservation groups, 63 (80.8%) were predicted as HLA binders (percentile rank ≤ 10%; Supplementary Figure S25). Additionally, the remaining 15 peptides have been independently confirmed as positive T-cell epitopes via IEDB T-cell assay records.

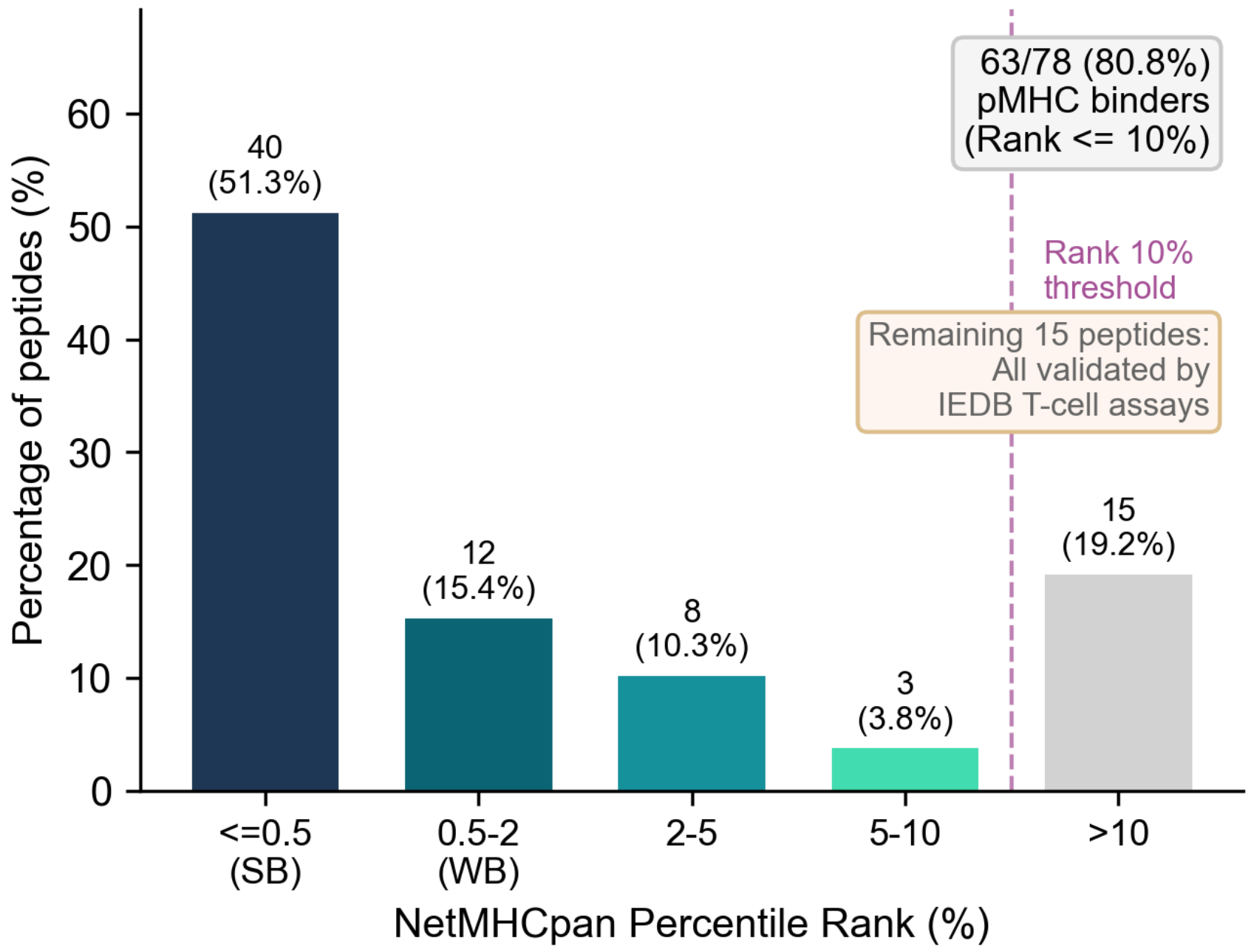

Figure S25. Predicted pMHC binding affinity of cross-reactive peptides in the low-conservation group. *Distribution of NetMHCpan 4.1 predicted percentile ranks for 78 peptides from the low-conservation cross-reactivity groups (conservation ratio < 0.5). Peptides were classified as strong binders (SB; percentile rank ≤ 0.5%), weak binders (WB; 0.5% < rank ≤ 2%), or non-binders (rank > 10%) according to standard thresholds. Of the 78 peptides, 63 (80.8%) were predicted to bind their cognate HLA molecule (rank ≤ 10%). The remaining 15 peptides, predominantly cysteine-rich sequences, were independently confirmed as positive T-cell epitopes restricted to HLA-A*02:01 via experimentally validated T-cell assay records in the IEDB database, indicating that the prediction discrepancy reflects a known limitation of NetMHCpan for such sequences rather than a genuine absence of HLA presentation.*

Comment 3)

Inclusion of non-HLA-A2-restricted TCRs would substantially enhance the generalizability of the approach, especially as the model is described as pan-specific.

Response: We thank the reviewer for this important suggestion. We agree that inclusion of non-HLA-A2-restricted TCRs is important for the generalizability of UpTCR.

We would like to clarify that while the experimental validation study predominantly involves HLA-A2-restricted TCRs, the computational benchmarking throughout the manuscript is pan-specific. Specifically, the cross-reactivity analysis encompasses TCRs restricted by multiple HLA alleles, such as HLA-A*02:01, HLA-B*07:02, HLA-B*27:05, HLA-B*35:08, and HLA-B*57:03.

Furthermore, we emphasize that UpTCR is pan-specific by both design and training. Architecturally, UpTCR represents HLA molecules using pseudo-sequences rather than allele-specific encodings, enabling the model to generalize across any HLA allele by learning residue-level binding patterns. In terms of training data, both the two-stage pretraining corpus and the finetuning dataset are not restricted to HLA-A*02:01. The novel-HLA split evaluation (Figure 3f), in which test-set HLA alleles were entirely absent from training, directly demonstrates the model's ability to generalize to unseen HLA restrictions. In addition, we visualized the learned embeddings across different HLA alleles (Figure 4a, Supplementary Figure S6a,c). Thus, the pan-specificity of UpTCR is inherent to its architecture and supported by all stages of model development.

We also acknowledge that additional wet-lab validation using non-HLA-A2-restricted TCRs would further strengthen the experimental demonstration of pan-specificity. Unfortunately, generating and functionally testing additional non-HLA-A2-restricted TCR systems requires substantial experimental resources. We have therefore clarified in the revised Discussion that the prospective experimental validation is currently focused on an HLA-A*02:01-restricted system, whereas the broader pan-specificity of UpTCR is supported by computational evaluations across diverse HLA restrictions.

To be clearer, the revised content is presented below.

Discussion (page 35)

Second, although the generalizability of UpTCR across diverse HLA restrictions has been supported by our computational evaluations, future wet-lab validation using additional

non-HLA-A2-restricted TCR systems would further strengthen the experimental evidence for its pan-specific applicability.

Results (page 17)

First, we visualized the antigen-HLA embeddings, focusing on three major HLA gene loci: HLA-A, HLA-B, and HLA-C. …Similar patterns were observed for HLA-B*08:01 (Figure 4a middle) and HLA-C*15:02 (Figure 4a right), demonstrating the consistency and generalizability of the model's learned representations across different HLA alleles.

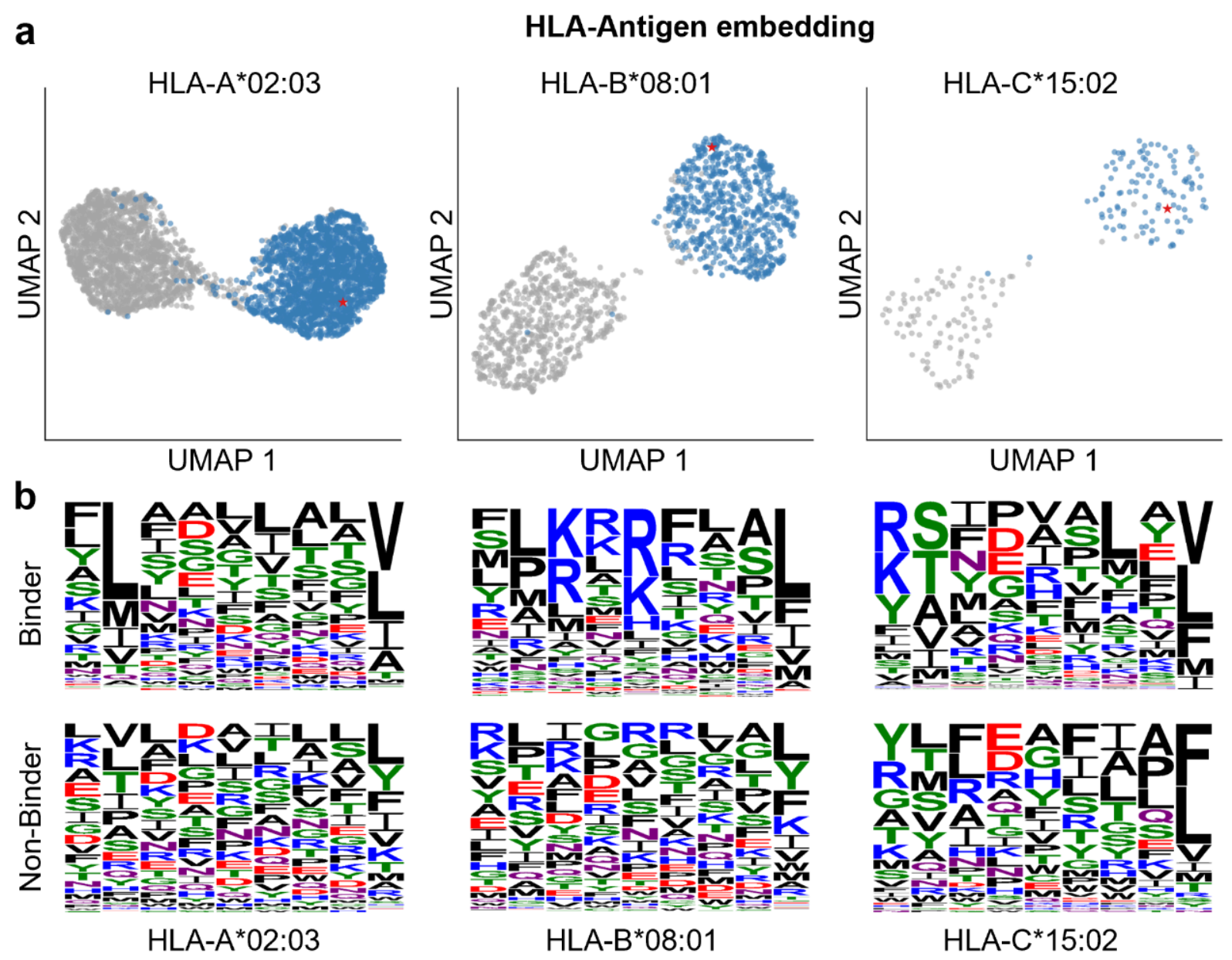

***Figure 4. UpTCR effectively captures biologically meaningful and discriminative embeddings for TCR-antigen-HLA interactions. a**, Uniform manifold approximation and projection (UMAP) of the HLA-specific antigen embeddings, including different HLA loci, i.e. HLA-A*02:03 (left), HLA-B*08:01 (middle), and HLA-C*15:02 (right). The HLA, binders, and non-binders are colored in red, blue, and gray, respectively. **b**, Sequence motifs of antigen binders and non-binders targeting HLA-A*02:03 (left), HLA-B*08:01 (middle), and HLA-C*15:02 (right). Both binders and non-binders exhibit substantial sequence heterogeneity across all three HLA alleles…*

Comment 4)

In Figure 6, the interaction conformation prediction module was trained on only 164 crystal structures. Given the high dimensionality of residue-level contact maps, this dataset size may be insufficient to avoid overfitting. The authors should provide a validation using a strictly held-out structural dataset not used during any part of the pretraining or finetuning phases.

Response: We thank the reviewer for raising this important concern regarding potential overfitting given the limited size of the training set (164 crystal structures) relative to the high dimensionality of residue-level contact maps.

To directly address this concern, we curated a strictly independent structural test set comprising 39 TCR-antigen-HLA complex structures that were completely excluded from all stages of model development, including pretraining and fine-tuning. On this held-out test set, UpTCR outperformed TEIM-Res across all six pairwise interaction types (Figure 6b), confirming that the model generalizes to unseen structural data rather than merely memorizing the training structures. Furthermore, to mitigate the data scarcity issue raised by the reviewer, we explored data augmentation using high-confidence AlphaFold-predicted complex structures. The augmented variant, denoted UpTCR+AF, achieved the highest performance on the independent test set, with a mean AUC improvement of 3.1% over UpTCR across all tasks.

The description of the independent structural test set has been added to the Methods section, and the results are presented in revised Figure 6b, which is presented as below.

Results (page 24-25):

To rigorously assess model's generalizability, we curated an independent test set of 39 complex structures, which were not used in any stage of pretraining and finetuning. On this held-out set, UpTCR consistently outperformed TEIM-Res across all six interaction types (Figure 6b), confirming the model's ability to generalize to unseen structural data. UpTCR+AF achieved the highest performance on the independent test set, with a mean AUC improvement of 3.1% over UpTCR across all tasks.

Methods (page 39-40):

We also constructed a held-out independent test set. This test set was curated from TCR-antigen-HLA complex structures[12]. From this resource, we identified 39 available quaternary complex structures (containing complete TCRα, TCRβ, antigen, and HLA chains) that were deposited in the Protein Data Bank. To ensure complete independence, these structures were not used at any stage of pretraining or fine-tuning to provide an unbiased evaluation.

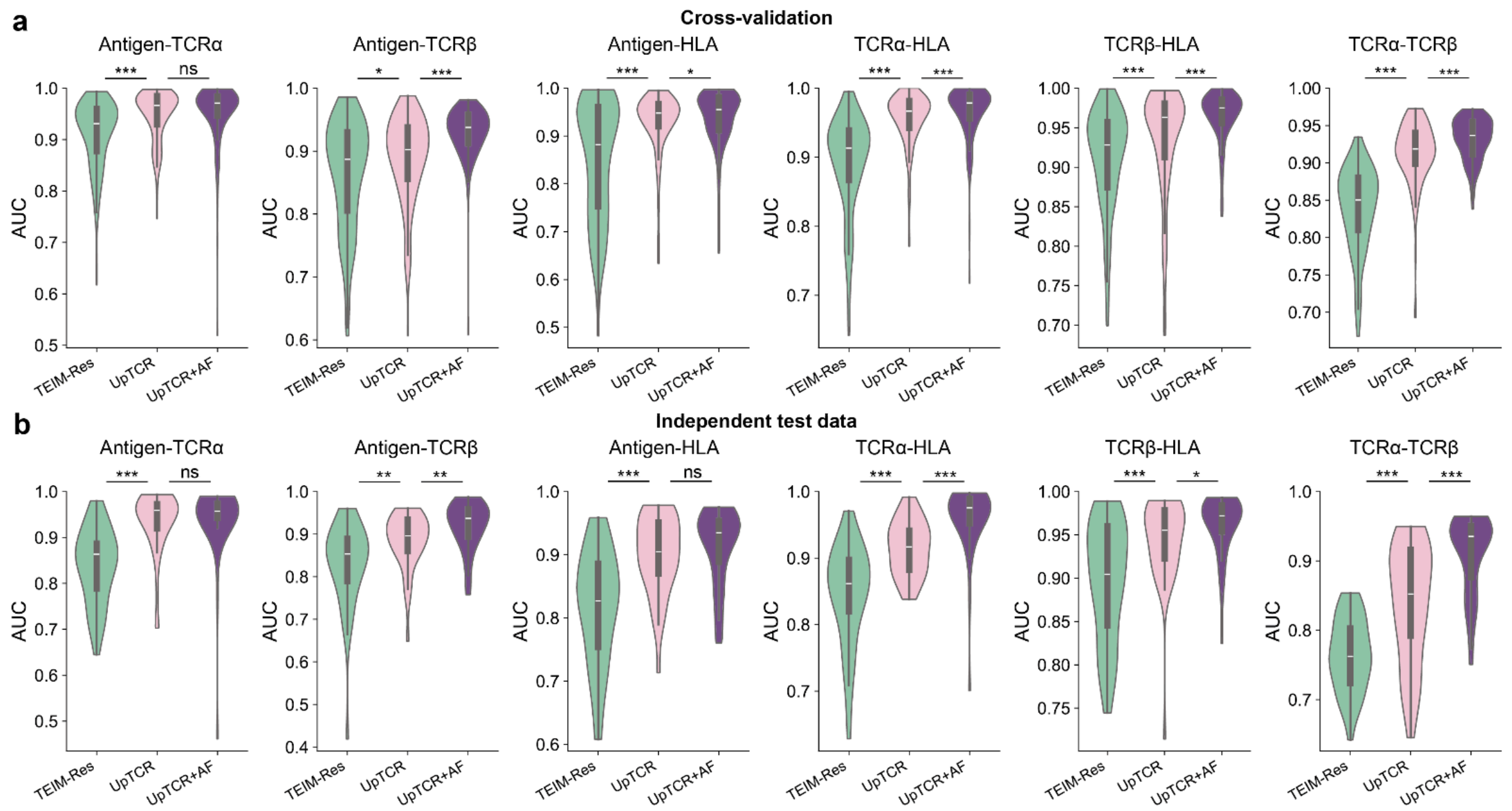

Figure 6. UpTCR facilitates investigations of any pairwise residue contact relationship among full-length TCRα, full-length TCRβ, Antigen, and HLA. a-b, *Violin plots displaying the cross-validation (**a**) and independent test (**b**) AUC for predicting residue-level contact maps between any pairwise components by TEIM-Res, UpTCR, and UpTCR+AF. From left to right: antigen-TCRα, antigen-TCRβ, antigen-HLA, TCRα-HLA, TCRβ-HLA, and TCRα-TCRβ. UpTCR+AF denotes UpTCR further trained with additional high-confidence AlphaFold-predicted complex structures. The independent test set comprises 39 TCR-antigen-HLA complex structures that were excluded from all stages of pretraining and fine-tuning….*

Comment 5)

In Figure 7, T cells were stimulated with peptide pools on days 3 and 7 of a 7-day culture. Repeated stimulation within a short window can induce activation-induced cell death (AICD) or promote dysfunctional expansion in primary CD8+ T cells. The authors should justify why this specific regimen was chosen and how it might impact the reliability of the resulting TCR dataset.

Response: We thank the reviewer for this insightful question regarding the potential impact of the stimulation regimen on our TCR dataset. We address this concern from multiple perspectives:

First, our stimulation protocol follows the well-established Multiplex Identification of T-cell Receptor Antigen Specificity (MIRA) approach (Klinger et al., 2015) [1], which has been widely adopted for identifying antigen-specific TCR clonotypes from peripheral blood. Our protocol was designed to address the challenge of extremely low frequencies of antigen-specific T cells in

peripheral blood, and the two-stimulation strategy (day 3 and day 7) is a well-established optimization for expanding rare clones. The two-round stimulation strategy was specifically designed to: (i) initiate the activation and clonal expansion of antigen-specific T cells on day 3, and (ii) re-boost expansion and maximize CD137 upregulation on day 7, just 24 hours before cell sorting on day 8. The 4-day interval between the two stimulations provides adequate time for T cell recovery and proliferation, minimizing the risk of extensive AICD. As shown in Table S4, cell viability in all stimulated groups on day 7 ranged from 81.16% to 93.84%, comparable to the negative control (88.8%), with no signature of massive AICD.

Second, our use of CD137 (4-1BB) as the activation marker for cell sorting provides a built-in quality control against the concerns raised. CD137 is specifically upregulated on functionally activated T cells following recent antigen engagement. Cells that have undergone AICD are eliminated during flow cytometry through forward/side scatter gating and viability exclusion. Furthermore, T cells that have become dysfunctional or anergic due to overstimulation exhibit impaired CD137 upregulation and would therefore not be captured by our $CD3^{+}CD8^{+}CD137^{+}$ gating strategy. Thus, the sorted population is inherently enriched for functionally competent, antigen-responsive T cells.

Third, the inclusion of both positive (SEB) and negative (unstimulated) controls in each experiment allowed us to validate T cell viability and responsiveness throughout the culture period. TCR clonotype enrichment was calculated relative to the unstimulated control, and stringent enrichment thresholds were applied to establish the high-confidence dataset, further mitigating potential artifacts from non-specific or dysfunctional expansion.

We have revised the Methods section to clarify the rationale for the stimulation regimen and how the CD137-based sorting strategy mitigates AICD concerns.

[1] Klinger, M. et al. Multiplex identification of antigen-specific T cell receptors using a combination of immune assays and immune receptor sequencing. PloS One 10, e0141561 (2015).

Methods (page 40-41):

Each PBMC sample was divided into ten equal parts, with eight parts used for antigen stimulation with the respective peptide pools, and the remaining two serving as controls: one negative (no peptide stimulation) and one positive (stimulated with 1 μg/mL SEB). Cells were cultured for seven days, with peptide stimulation administered on days 3 and 7. This two-round stimulation regimen was adopted to expand the rare antigen-specific clones present at very low frequency in peripheral blood, while the four-day interval between stimulations allowed T-cell recovery and

proliferation, minimizing the risk of activation-induced cell death (AICD). Consistent with this, all stimulated groups remained highly viable on day 7 (81.2%–93.8%, comparable to the 88.8% of the unstimulated control) and exhibited net cell expansion (51.6%–242.1% increase in cell number; Supplementary Table S4), arguing against substantial AICD. On day 8, cells were stained with CD3, CD8, and the activation marker CD137, and $CD3^{+}CD8^{+}CD137^{+}$ activated T cells were sorted by flow cytometry. Because AICD-affected and anergic/dysfunctional cells are excluded by forward/side-scatter and viability gating and fail to upregulate CD137, this $CD3^{+}CD8^{+}CD137^{+}$ gating strategy inherently enriches for functionally competent, antigen-responsive T cells. RNA was extracted from the sorted cells for immune repertoire library construction, and bulk TCR sequencing was performed to identify TCR clonotypes enriched in each peptide pool. By applying the "unique address" design in conjunction with the sequencing data, a direct correspondence between antigen identity and TCR sequence was established, enabling accurate identification of antigen-specific TCR clones.

Table S4. PBMC expansion and viability after antigen stimulation.

Group	Day 0 cell count	Day 7 cell count	Viability (%)	Expansion rate (%)
Group 1	3.74×10^5	9.50×10^5	91.94	153.88
Group 2	3.74×10^5	1.28×10^6	91.36	242.11
Group 3	3.74×10^5	6.44×10^5	87.61	72.06
Group 4	3.74×10^5	1.13×10^6	91.54	201.20
Group 5	3.74×10^5	7.92×10^5	93.84	111.76
Group 6	3.74×10^5	7.46×10^5	91.83	99.33
Group 7	3.74×10^5	8.90×10^5	92.06	137.83
Group 8	3.74×10^5	7.28×10^5	81.16	94.52
Negative control	3.74×10^5	5.67×10^5	88.8	51.60
Total	3.74×10^5	7.72×10^6	-	106.43

Comment 6)

The "unique address" peptide matrix logic for identifying antigen-specific TCRs should be more clearly explained to ensure that potential overlapping specificities within the eight peptide pools did not introduce false positives in the Luminal or TNBC cohorts.

Response: We thank the reviewer for the opportunity to clarify the logic of our "unique address" peptide-pool matrix. The detailed principle has been described in Klinger et al., 2015. Specifically, the design is fundamentally a combinatorial encoding scheme: 32 peptides are distributed across 8 peptide pools, with each peptide assigned a unique 8-bit binary address. For example, peptide P1 has the address ACDF (pools 1, 3, 4, 6) and peptide P2 has the address BEGH (pools 2, 5, 7, 8). A T cell truly specific to a given peptide will be enriched exclusively in pools containing that peptide, and its activation pattern should strictly match the address. Regarding the concern that cross-reactivity might introduce false positives, our data processing pipeline incorporates stringent data filtering: TCRs appearing in 3 or 4 pools with a perfect match to a single peptide address are designated "YG" (strict match). TCRs appearing in 5 or more pools, which could arise from cross-reactivity or noise, are subjected to Mann-Whitney U testing and clustering analysis to statistically determine their most likely cognate peptide, effectively filtering out ambiguous signals. Moreover, the biological results from the two patient cohorts provide compelling evidence against false positives: the Luminal subtype exhibited stronger responses to antigens such as Mam-A, while the TNBC subtype showed stronger responses to survivin and KK-LC-1. This subtype-specific differential pattern strongly validates the high specificity of our screening method.

Methods (page 40-41):

In this combinatorial encoding scheme, the eight pools were labeled A–H, and each of the 32 peptides was assigned a unique combination of four of the eight pools as its "address" (e.g., P1 → pools A, C, D, F; P2 → pools B, E, G, H), such that a T cell specific to a given peptide is expected to be enriched exclusively in the four pools comprising that peptide's address.

…

The in-house TCR screening project yielded a robust dataset for analyzing antigen-specific T-cell responses in breast cancer. To ensure statistical rigor and minimize false positives, we established a high-confidence dataset by applying stringent thresholds for TCR clonotype enrichment relative to controls. Specifically, TCRs whose enrichment matched a single four-pool address (allowing at most one pool dropout, i.e., enrichment in three to four pools) were retained as high-confidence (strict-match) assignments, whereas TCRs enriched in five or more pools—potentially arising from cross-reactivity or technical noise—were resolved by Mann–Whitney U testing and clustering analysis to determine their most likely cognate peptide, thereby filtering out ambiguous signals. The subtype-specific response patterns observed (e.g., stronger Mam-A responses in the Luminal subtype and stronger survivin and KK-LC-1 responses in the TNBC subtype) further support the high specificity of this screening strategy and argue against substantial false-positive contamination.

Comment 7)

Figure 8 experiment would benefit from well-characterized literature-based positive and negative control TCRs to benchmark against the Melan-A-specific TCR used. The variants tested (e.g., Antigen HV1-3) closely resemble the canonical MART1 epitope. Given the claim that the model operates in zero-shot settings, experimental evaluation using variant peptides with significantly lower sequence identity would provide a more compelling demonstration of model robustness.

Response: We thank the reviewer for these constructive suggestions. We agree that including literature-based control TCRs and testing lower-identity peptide variants are important for strengthening the prospective validation experiment.

First, we incorporated literature-based control TCRs to benchmark the experimental system. Specifically, we included MEL8, a well-characterized Melan-A/MART-1-specific TCR reported in the literature [1], as a positive control. MEL8 TCR-expressing cells responded robustly to the native Melan-A peptide (Supplementary Figure S27, $P < 0.01$), confirming the reliability of our assay.

Second, to address the reviewer's concern regarding sequence identity, we substantially expanded the tested peptide panel. In the revision, we now include eight predicted high-binding variants (AHV1–AHV8) spanning a wide range of sequence identity to the native epitope (40%–80%).

Critically, the five newly added low-identity variants (AHV4–AHV8, 40%–54.5% identity) were all predicted by UpTCR as high-binding and were experimentally confirmed to induce T-cell activation (Figure 8b,c). These variants were absent from the training data, effectively constituting a zero-shot prediction scenario as the reviewer noted. We have updated Figure 8, the corresponding Results and Methods sections, and added Supplementary Figures S26-S27 to reflect these expanded experiments.

[1] Dolton, Garry, et al. "Targeting of multiple tumor-associated antigens by individual T cell receptors during successful cancer immunotherapy." Cell 186.16 (2023): 3333-3349.

Results (page 31)

For each candidate, we predicted the binding probability using UpTCR and ranked the variants by predicted score. From the ranked list, we selected eight predicted high-binding variants (AHV1-AHV8) spanning a wide range of sequence identity to the native peptide (40%-80%) and one predicted low-binding variant (AntigenLV, 60% identity) for experimental validation (Table S2). Notably, several predicted high-binding variants (AHV4-AHV8) share substantially lower

sequence identity (40%-54.5%) with the native epitope, providing a stringent evaluation of the model's generalization capability.

…

To further benchmark assay specificity, we included a literature-reported Melan-A-specific MEL8 TCR[13] as a positive control. MEL8 TCR-expressing cells responded significantly to the native Melan-A peptide (Supplementary Figure S27), confirming the reliability of the experimental system.

…

Consistent with the computational predictions, the predicted high-binding variants AHV1-AHV8 induced higher IL-2 secretion than the predicted low-binding variant AntigenLV and unrelated peptide controls (Figure 8b). Similarly, flow cytometry analysis showed an increased proportion of CD69+ cells among CD8+ T cells upon stimulation with AHV1-AHV8, whereas AntigenLV, unrelated peptide, and DMSO controls induced minimal activation (Figure 8c,d). Importantly, the low-identity variants also elicited T cell activation, supporting the ability of UpTCR to identify TCR-recognized peptide variants beyond near-canonical sequence similarity.

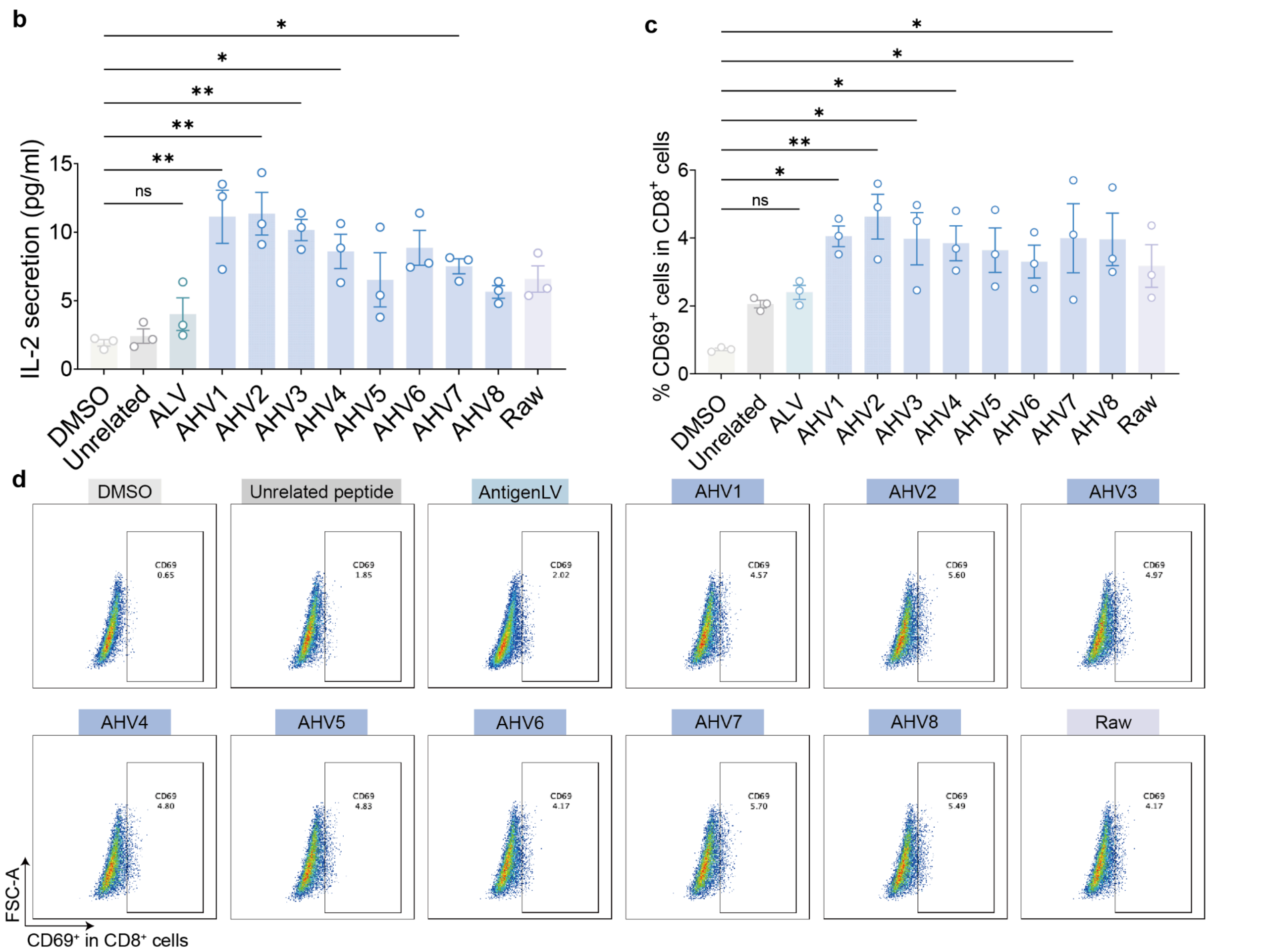

Figure 8. … b, *IL-2 secretion measured by ELISA after 24 h stimulation with DMSO, unrelated peptide, AntigenLV, AHV1–AHV8, or the native peptide (Raw) (n = 3 independent batches).* ***c,*** *Frequency of CD69$^+$ cells among CD8$^+$ T cells measured by flow cytometry after 24 h stimulation with the indicated peptides or controls (n = 3 independent batches).* ***d,*** *Representative flow cytometry plots showing CD69 expression in CD8$^+$ T cells following stimulation with DMSO, unrelated peptide, AntigenLV, AHV1–AHV8, or the native peptide (Raw). Data are presented as mean ± SD. Statistical significance was assessed by one-way ANOVA with multiple-comparison correction (**b-c**). * $P < 0.05$, ** $P < 0.01$, *** $P < 0.001$, **** $P < 0.0001$, ns indicates no significant difference.*

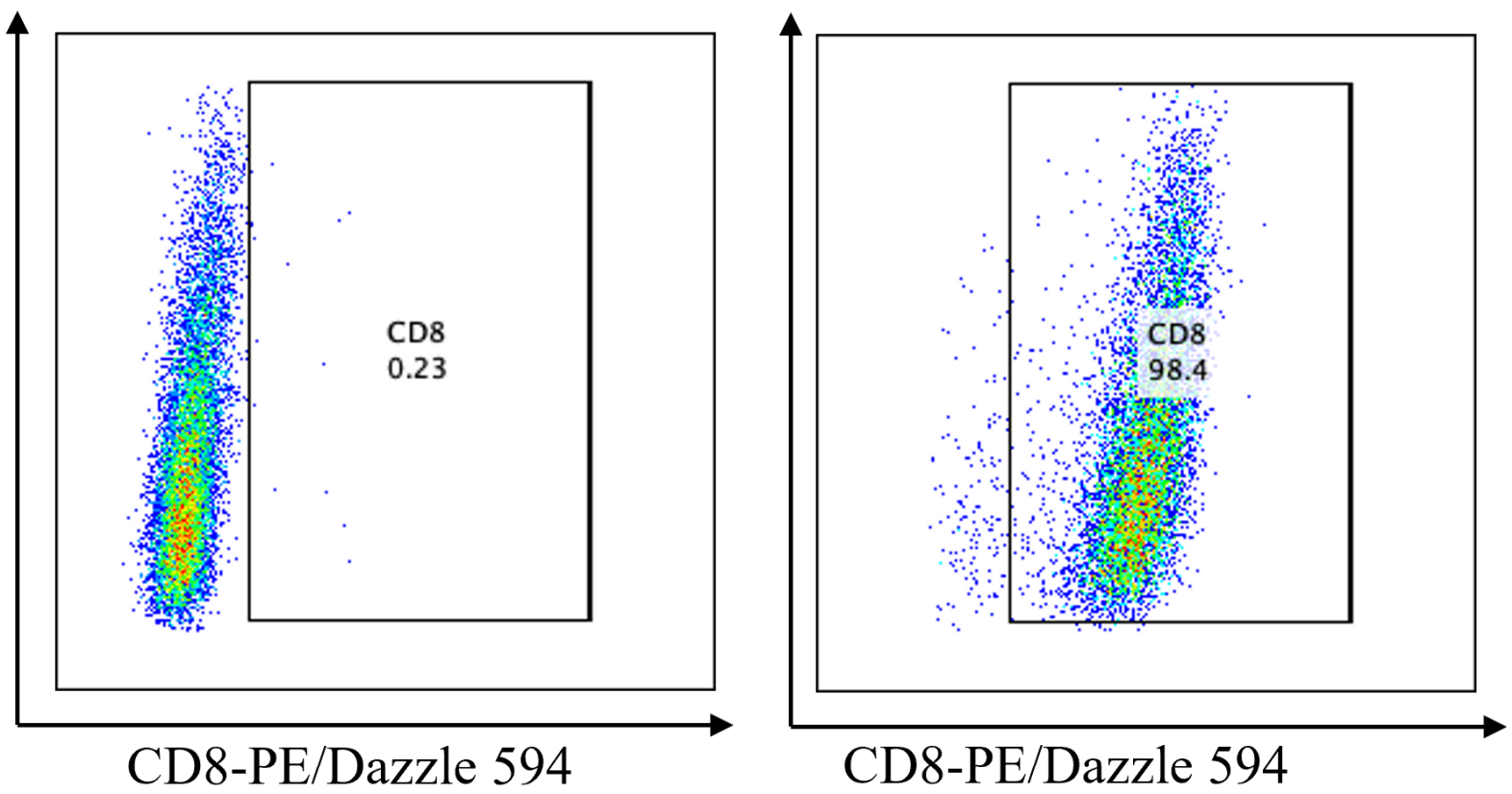

Figure S26. Flow cytometry validation of CD8 expression in engineered Jurkat T cells. *Representative flow cytometry plots showing CD8 expression in parental Jurkat T cells and CD8-transduced Jurkat T cells. Parental Jurkat T cells showed negligible CD8 expression, consistent with their endogenous CD4$^+$CD8$^-$ phenotype, whereas CD8-transduced Jurkat T cells exhibited robust CD8 expression. These results confirm successful generation of CD8-expressing Jurkat T cells for TCR stimulation and activation assays.*

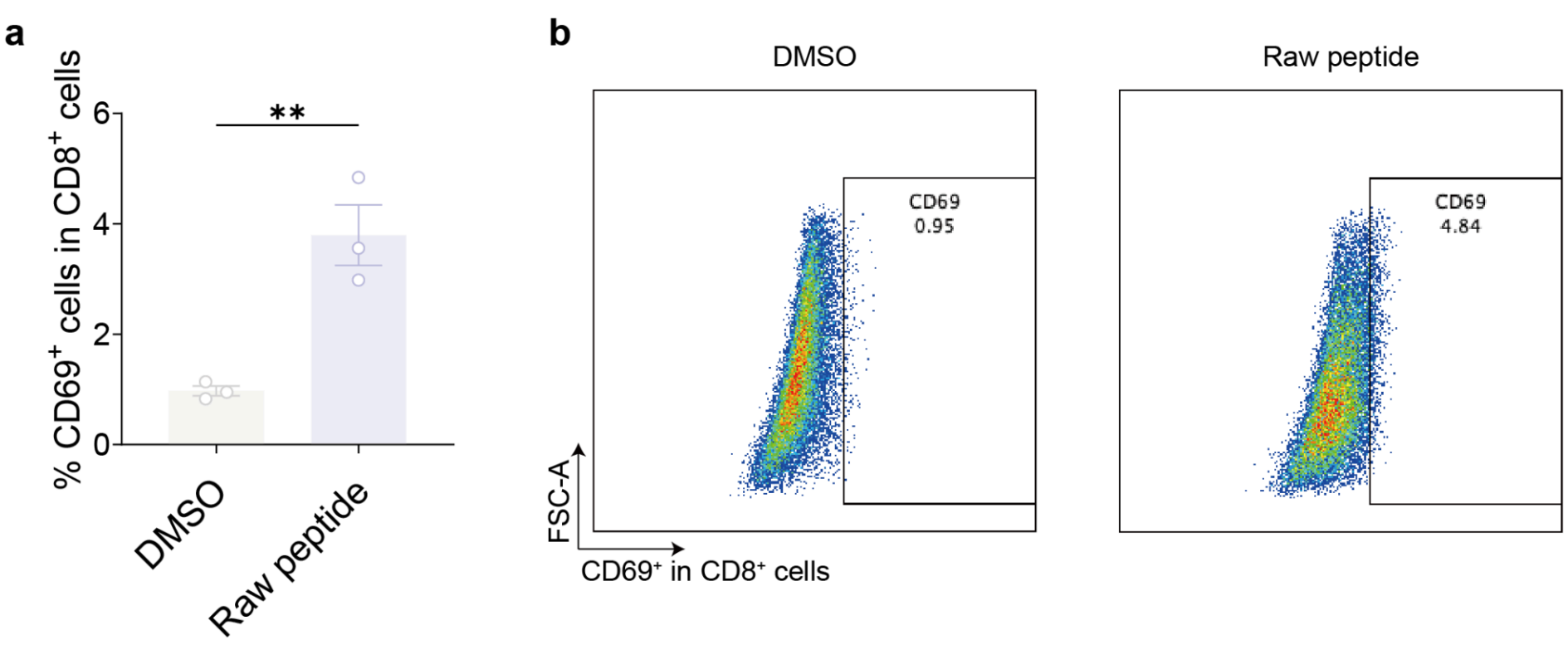

Figure S27. Flow cytometry validation of the MEL8 TCR response to the native Melan-A peptide. a, *Quantification of CD69$^+$ cells among CD8$^+$ T cells after stimulation with DMSO or the native Melan-A peptide (Raw peptide) in MEL8 TCR-expressing cells. Data are presented as mean*

*± SD from three independent batches. b, Representative flow cytometry plots showing CD69 expression within CD8⁺ T cells after DMSO or native Melan-A peptide stimulation. **P < 0.01.*

Table S2. Screened antigen variants in the prospective study.

Antigen	Sequence	Sequence identity to raw
Raw sequence (MELAN-10)	EAAGIGILTV	-
AntigenHV1	ELAGIAILTV	80%
AntigenHV2	ELAGIGILAV	80%
AntigenHV3	AAGIGILTVI	80%
AntigenHV4	FLAGGIGIRTL	54.5%
AntigenHV5	ALGGLLTMV	50%
AntigenHV6	SMLGIGIVPV	50%
AntigenHV7	LGIGVITI	40%
AntigenHV8	SLWLGILSV	40%
AntigenLV	SMAGIGIVDV	60%

Comment 8)

For the specificity task, negative samples were generated via a 1:5 ratio of positive to shuffled negative pairs. While the authors argue that background sampling introduces bias, random shuffling can create "biologically impossible" negatives that the model may find too easy to distinguish. The inclusion of "hard negatives"—such as non-binding homologous peptides or TCRs—would provide a more realistic assessment of the model's discriminative boundary.

Response: We thank the reviewer for this important suggestion. We agree that randomly shuffled negatives may generate biologically implausible pairs that are trivially separable, potentially overestimating model performance. The inclusion of hard negatives provides a more realistic and stringent assessment of the model's discriminative boundary.

To directly address this concern, we constructed a dedicated hard-negative benchmark comprising 3,495 TCR-epitope pairs. In this benchmark, each TCR is paired with one experimentally confirmed binding epitope and one closely related non-binding variant differing by only 1–4 amino acid

substitutions, while being presented by the same HLA allele. Notably, 92.7% of pairs differ by only a single amino acid substitution. This setting is substantially more challenging than random shuffling because the model cannot rely on sequence dissimilarity but must instead identify specific binding-disrupting substitutions at individual positions. These pairs were curated from experimentally validated escape mutations in the IEDB/VDJdb databases.

On this hard-negative benchmark, UpTCR achieved a pairwise accuracy of 0.799, a mean score margin of 0.715, and an AUC of 0.781, consistently outperforming DLpTCR, PanPep, and EPACT across all evaluated metrics (Supplementary Figure S24). These results demonstrate that UpTCR captures fine-grained determinants of TCR–epitope recognition and that its specificity predictions are not merely driven by the easy separability of randomly shuffled negatives.

To be clearer, the revised content is presented below.

Results (page 10-11):

To further evaluate UpTCR's discriminative power in a biologically stringent setting, we constructed a hard-negative benchmark comprising 3,495 TCR-epitope pairs. In each pair, a TCR is matched with one experimentally confirmed binding epitope and one non-binding homologous epitope differing by only a few amino acid substitutions (92.7% with a single substitution) and originating from the same pathogen. This design ensures that the model cannot exploit global sequence dissimilarity and must instead identify binding-critical residue positions. UpTCR achieved a pairwise accuracy of 0.799, consistently outperforming DLpTCR, PanPep, and EPACT across all metrics (Supplementary Figure S24), demonstrating its ability to discriminate biologically plausible hard negatives from true binders.

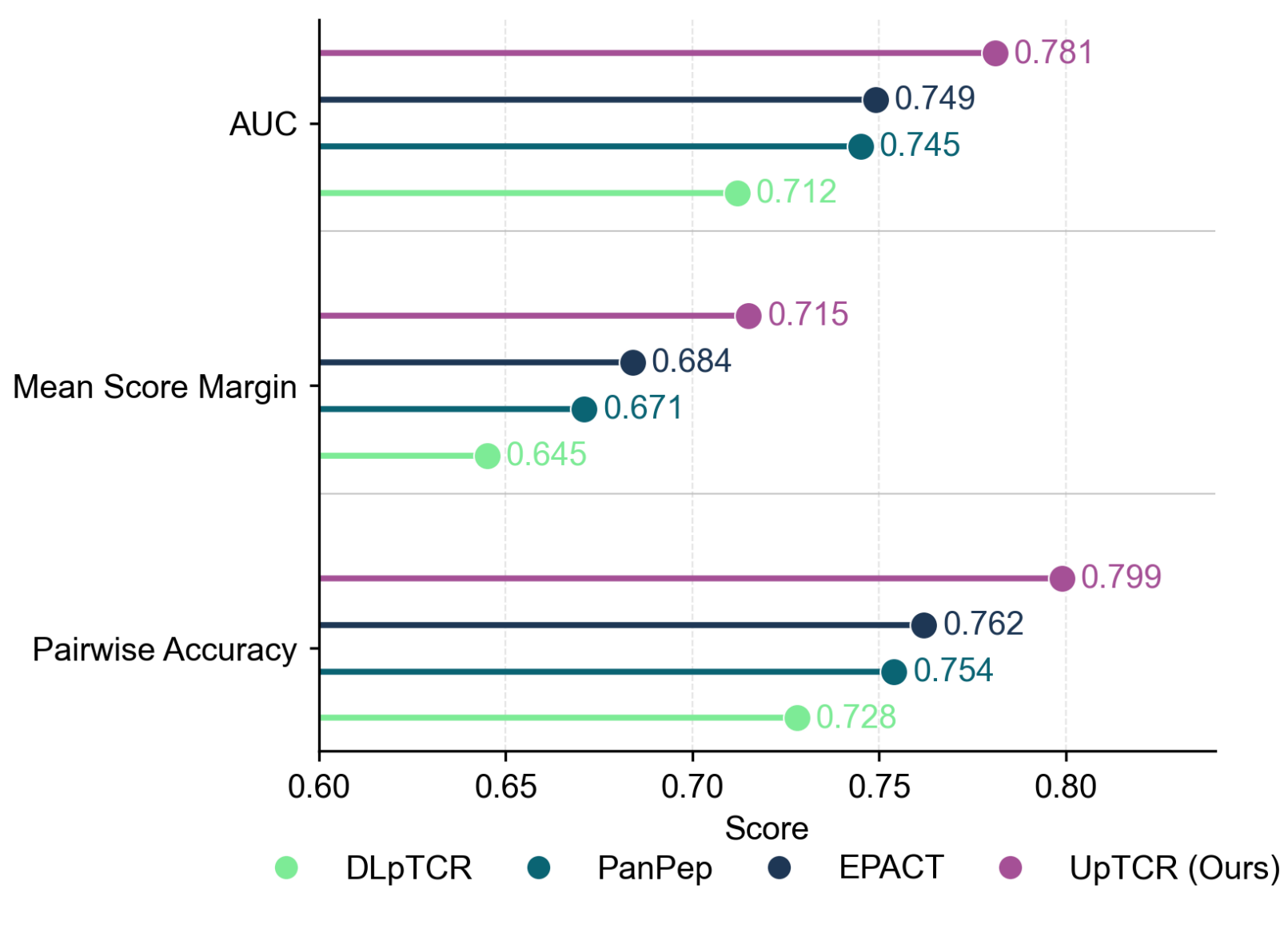

Figure S24. Hard negative discrimination performance on mutation epitope pairs. *Comparison of UpTCR and three baseline methods on a hard negative benchmark consisting of 3,495 TCR-epitope pairs, where each TCR is paired with one confirmed binding epitope and one non-binding epitope differing by amino acid substitutions. Performance was assessed by three metrics: pairwise accuracy (proportion of pairs where the binding epitope received a higher predicted score than the non-binding epitope), mean score margin (average difference between positive and negative prediction scores), and area under the receiver operating characteristic curve (AUC). UpTCR consistently outperformed all baselines across all metrics.*

Comment 9)

The authors demonstrate that pretraining helps, but it is unclear if the specific "triplet" stage (Stage II) provides a significant marginal gain over just the "dimer" stage (Stage I) for all tasks. A clearer ablation study isolating the performance increase attributed solely to the transition from Stage I to Stage II pretraining would clarify the necessity of this multi-scale approach.

Response: We sincerely thank the reviewer for this suggestion to clarify the marginal contribution of each pretraining stage. In the revised manuscript, we performed a stage-wise ablation study comparing four pretraining configurations: (1) No Pretrain, (2) Only Stage I (dimer-level pretraining of the four monomer encoders), (3) Only Stage II (trimer-level pretraining of the two fusion encoders without Stage I initialization), and (4) Full UpTCR.

The results (Supplementary Figure S20) clearly demonstrate the marginal gain of Stage II over Stage I alone. Under the few-shot setting, transitioning from Only Stage I to Full UpTCR yielded improvements of 4.8% in AUC (0.752 -> 0.800) and 8.0% in AUPR (0.481 -> 0.561). Under the zero-shot setting, the marginal gains were more pronounced, with improvements of 6.9% in AUC (0.573 -> 0.642) and 8.1% in AUPR (0.215 -> 0.296). Both Only Stage I and Only Stage II variants performed significantly worse than Full UpTCR ($P < 0.001$), confirming the necessity of the progressive multi-stage approach. Notably, Only Stage I and Only Stage II achieved comparable performance levels (*e.g.*, few-shot AUC: 0.752 vs. 0.745), yet both remained substantially inferior to the full progressive model, indicating that the two stages provide complementary rather than redundant knowledge. Stage I establishes foundational molecular representations by aligning individual component embeddings into a shared latent space, whereas Stage II builds upon these aligned representations to capture higher-order interaction patterns.

The corresponding content is added in the revised manuscript, which is presented below.

Results (page 14):

To clarify the respective contributions of the two pretraining stages, we performed a stage-wise ablation study comparing four configurations: No Pretrain, Only Stage I (dimer-level pretraining), Only Stage II (trimer-level pretraining without Stage I initialization), and the complete UpTCR. Under the few-shot setting, transitioning from Only Stage I to Full UpTCR yielded improvements of 4.8% in AUC and 8.0% in AUPR; under the zero-shot setting, the marginal gains were 6.9% in AUC and 8.1% in AUPR. Both Only Stage I and Only Stage II performed significantly worse than Full UpTCR ($P < 0.001$, Supplementary Figure S20), yet achieved comparable levels to each other (e.g., few-shot AUC: 0.752 versus 0.745) , indicating that the two stages contribute complementary rather than redundant knowledge. Stage I establishes foundational molecular representations by aligning the embeddings of individual components into a shared latent space, whereas Stage II builds upon these representations to capture higher-order interaction patterns by integrating fused TCRαβ and antigen-HLA complexes through trimer-level contrastive learning.

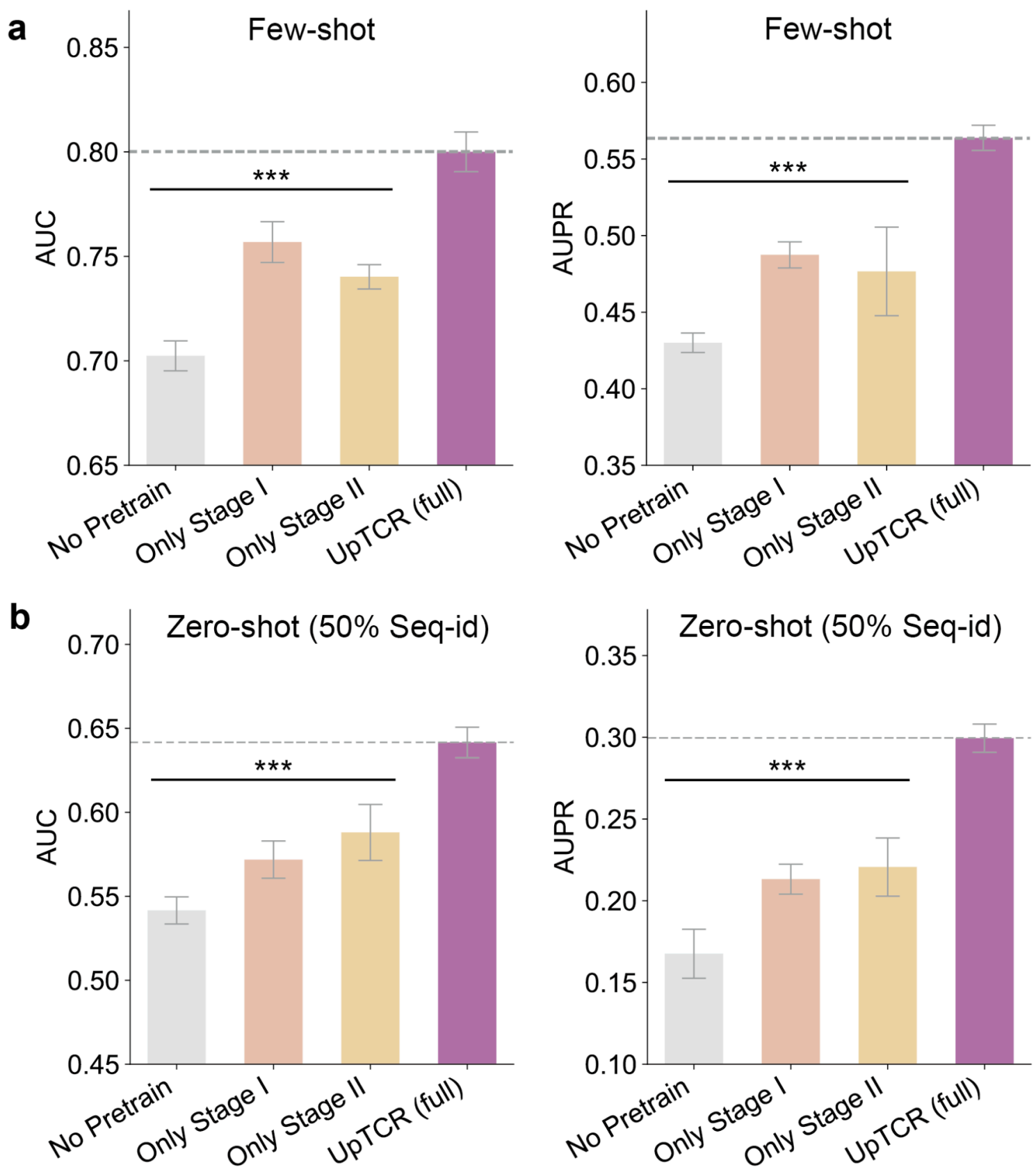

Figure S20. Stage-wise pretraining ablation demonstrated the contributions of both pretraining stages. a, *AUC (left) and AUPR (right) for TCR-antigen-HLA binding specificity prediction under the few-shot setting across four pretraining configurations: No Pretrain (all encoders randomly initialized), Only Stage I (dimer-level pretraining only), Only Stage II (trimer-level pretraining*

only), and UpTCR (full progressive pretraining). ***b,*** *Same analysis under the zero-shot setting (50% sequence identity filtering). *** P < 0.001.*

Comment 10)

In Table S1, the column header "alpha_v" is duplicated; the second instance should be corrected to "beta_v" to correctly identify the V-gene usage for the beta chain.

Response: We thank the reviewer for carefully examining Supplementary Table S1. We confirm that the duplicated "alpha_v" column header is a typographical error. The second instance has been corrected to "beta_v" in the revised Supplementary Table S1.

To be clearer, the revised content is present below.

Table S1. TCR information in the cross-reactivity prediction.

	alpha_j	beta_j	alpha_v	beta_v	CDR3α	CDR3β

Comment 11)

To ensure reproducibility, the table should include the full CDR3 sequences (both alpha and beta chains) and the specific predicted peptide targets for all 20 reported cross-reactive TCR groups. Currently, only J and V gene identifiers are provided.

Response: We thank the reviewer for this suggestion to improve reproducibility. We agree that the original Supplementary Table S1 lacked essential sequence information.

In the revised manuscript, we now provide the full CDR3α and CDR3β amino acid sequences along with the V-gene and J-gene identifiers in the revised Supplementary Table S1.

Additionally, the complete list of specific peptide targets associated with each of the 20 cross-reactive TCR groups is provided in a dedicated source data file, enabling independent researchers to reproduce the cross-reactivity analysis. For the reviewer's convenience, we present representative examples in Response Table S3-1.

To be clearer, the revised content is present below.

Table S1. TCR information in the cross-reactivity prediction.

	alpha_j	beta_j	alpha_v	beta_v	CDR3α	CDR3β
TCR-C1	TRAJ37*01	TRBJ1-2*01	TRAV13-1*02	TRBV10-3*02	CAASLNTGKLIF	CAILGQGYGYTF
TCR-C2	TRAJ49	TRBJ1-2	TRAV24	TRBV6-5	CARNTGNQFYF	CASSPVTGGIYGYTF
TCR-C3	TRAJ15*01	TRBJ2-5*01	TRAV14N-1*01	TRBV17*01	CAASETSGSWQLIF	CASSRDLGRDTQYF
…	…	…	…	…	…	…

Response Table S3-1. Representative examples of TCR-anigen.

<table>
<tr><th>TCR</th><th>Antigen</th><th>TCR</th><th>Antigen</th></tr>
<tr><td rowspan="8">TCR-C1</td><td>APRGPHGGAASGL</td><td rowspan="8">TCR-C2</td><td>NLVPMVATV</td></tr>
<tr><td>APRGPHGGAAAGL</td><td>NLVPMVASV</td></tr>
<tr><td>APRGPHGGAASGA</td><td>NLVPVVATV</td></tr>
<tr><td>APRGPHGGAASAL</td><td>NLVGMVATV</td></tr>
<tr><td>APRGPHAGAASGL</td><td>NLVAMVATV</td></tr>
<tr><td>APRGAHGGAASGL</td><td>NLVPTVATV</td></tr>
<tr><td>AARGPHGGAASGL</td><td>NLVPQVATV</td></tr>
<tr><td>APAGPHGGAASGL</td><td></td></tr>
</table>

Reviewer #4 (Remarks to the Author):

Comment 1)

I co-reviewed this manuscript with one of the reviewers who provided the listed reports. This is part of the Nature Communications initiative to facilitate training in peer review and to provide appropriate recognition for Early Career Researchers who co-review manuscripts.

Response: We sincerely thank Reviewer #4 for co-reviewing this manuscript and providing valuable feedback alongside another reviewer. We appreciate the time and effort contributed to offering constructive suggestions for improvement.

Reference

1. Peng, X. *et al.* Characterizing the interaction conformation between T-cell receptors and epitopes with deep learning. *Nat. Mach. Intell.* **5**, 395–407 (2023).
2. Montemurro, A. *et al.* NetTCR-2.0 enables accurate prediction of TCR-peptide binding by using paired TCRα and β sequence data. *Commun. Biol.* **4**, 1060 (2021).
3. Springer, I., Besser, H., Tickotsky-Moskovitz, N., Dvorkin, S. & Louzoun, Y. Prediction of specific TCR-peptide binding from large dictionaries of TCR-peptide pairs. *Front. Immunol.* **11**, 1803 (2020).
4. Xu, Z. *et al.* DLpTCR: an ensemble deep learning framework for predicting immunogenic peptide recognized by T cell receptor. *Brief. Bioinform.* **22**, bbab335 (2021).
5. Weber, A., Born, J. & Rodriguez Martínez, M. TITAN: T-cell receptor specificity prediction with bimodal attention networks. *Bioinformatics* **37**, i237–i244 (2021).
6. Gao, Y. *et al.* Pan-peptide meta learning for T-cell receptor–antigen binding recognition. *Nat. Mach. Intell.* **5**, 236–249 (2023).
7. Lu, T. *et al.* Deep learning-based prediction of the T cell receptor–antigen binding specificity. *Nat. Mach. Intell.* **3**, 864–875 (2021).
8. Yu, C., Fang, X., Tian, S. & Liu, H. A unified cross-attention model for predicting antigen binding specificity to both HLA and TCR molecules. *Nat. Mach. Intell.* https://doi.org/10.1038/s42256-024-00973-w (2025) doi:10.1038/s42256-024-00973-w.
9. Feng, Z. *et al.* Sliding-attention transformer neural architecture for predicting T cell receptor–antigen–human leucocyte antigen binding. *Nat. Mach. Intell.* **6**, 1216–1230 (2024).
10. Zhang, Y. *et al.* Epitope-anchored contrastive transfer learning for paired CD8+ T cell receptor–antigen recognition. *Nat. Mach. Intell.* https://doi.org/10.1038/s42256-024-00913-8 (2024) doi:10.1038/s42256-024-00913-8.
11. Karnaukhov, V. K. *et al.* Structure-based prediction of T cell receptor recognition of unseen epitopes using TCRen. *Nat. Comput. Sci.* **4**, 510–521 (2024).
12. Quast, N. P. *et al.* T-cell receptor structures and predictive models reveal comparable alpha and beta chain structural diversity despite differing genetic complexity. *Commun. Biol.* **8**, 362 (2025).
13. Dolton, G. *et al.* Targeting of multiple tumor-associated antigens by individual T cell receptors during successful cancer immunotherapy. *Cell* **186**, 3333–3349 (2023).

Point-by-point response

Manuscript title: "UpTCR: a unified progressive knowledge transfer foundation model for robust T-cell receptor-antigen binding recognition"

Dear Reviewers,

First, thank you all for your valuable time, constructive comments, and helpful suggestions, which have significantly improved our manuscript. Here we include a point-by-point response to the reviewers, where *reviewers' comments are presented in italics*, our responses are in blue, and changes made to the manuscript are shown in red (*figure captions are in italics*).

The revised manuscript includes new computational and experimental analyses to address the reviewers' comments. Specifically, we (1) added a PMA/ionomycin receptor-bypassing positive control; (2) annotated the cross-reactivity groups (TCR-C1 to TCR-C20) with detailed information; and (3) evaluated UpTCR on an independent HLA-B*27:05-restricted autoantigen dataset. These changes are accompanied by new Supplementary Figures with corresponding updates to Results, Methods, and Discussion.

Table of contents

Reviewer #1 (Remarks to the Author): 3

Summary 3

Reviewer #2 (Remarks to the Author): 4

Summary 4

Reviewer #3 (Remarks to the Author): 5

Summary 5

Comment 1) 5

Comment 2) 7

Reviewer #4 (Remarks to the Author): 10

Summary 10

Comment 1) 10

Comment 2) 12

Comment 3) 14

Reference 16

Reviewer #1 (Remarks to the Author):

Summary

The manuscript has improved and my comments have been addressed. I consider the current version suitable for publication.

Response: We sincerely thank the reviewer for the careful evaluation of our manuscript throughout the review process and for the positive assessment of our revisions. We are grateful for the constructive comments raised in the previous round, which have substantially improved the quality and clarity of our work. We appreciate the reviewer's recommendation that the current version is suitable for publication.

Reviewer #2 (Remarks to the Author):

Summary

The authors did an excellent job responding to my questions.

I would like to note that, while the response to my Comment 2 is perfectly acceptable, the interpretation of the results could be made somewhat more cautious. Qualitatively, an AUC value of 0.59 indicates very limited predictive performance for practical purposes. It is factually correct that this is better than the next best method, which has an AUC of approximately 0.52 and is essentially close to random. However, in cases where no suitable training data are available to support inference by the ML approach (i.e., zero-shot training at a 30% identity cutoff between the training and testing sets) an AUC of 0.59 suggests only limited biologically useful information. Thus, there remains considerable scope for future work.

I leave this comment for the authors to consider. Overall, the revision was excellent. Congratulations.

Response: We sincerely thank the reviewer for the positive assessment of our revision and for this insightful comment. We fully agree that robust prediction for entirely unseen, sequence-divergent TCR-antigen pairs remains a critical open challenge. Bridging this gap undoubtedly represents a pivotal direction for future research. We anticipate that with ongoing methodological advancements and the increasing availability of high-quality experimental data, this challenge will be effectively resolved.

Reviewer #3 (Remarks to the Author):

Summary

The new computational analyses, together with the expanded functional validation, resolve majority of the concerns raised in the first round, and the manuscript is considerably stronger. However, two issues remain outstanding, which should be settled before publication because they bear on whether the functional and cross-reactivity claims can be independently interpreted and verified.

Response: We sincerely thank the reviewer for the positive assessment of our revision in the first round. We agree that ensuring the independent interpretability and verifiability of our functional and cross-reactivity claims is paramount before publication. To fully settle these remaining issues, we have added control experiment results and enriched our data annotations in this revision. Our detailed responses are provided below.

Comment 1)

1. The prospective validation in Figure 8 reads out TCR engagement as CD69 upregulation (and IL-2 secretion) in an engineered Jurkat line, and the revision helpfully adds a literature-derived positive-control TCR (MEL8) confirming that the cognate Melan-A peptide drives a response. However, the assay still lacks a receptor-independent positive control establishing that the Jurkat line itself is signaling-competent across the full panel. Jurkat cells are known to have a relatively narrow activation window in CD69-based readouts, and without such a control it is difficult to interpret the weaker or negative responses - in particular for the low-identity variants (AHV4-AHV8) and the predicted low-binder - as genuine absence of TCR recognition rather than limited assay sensitivity.

Please include a PMA/ionomycin (receptor-bypassing) positive control alongside the peptide stimulations to demonstrate maximal signaling capacity of the line, run within the

same experiments as Figure 8b-d. Confirming the key discriminations in a more responsive reporter system will strengthen the conclusion that the variant-specific differences reflect true differences in TCR recognition.

Response: We thank the reviewer for this crucial suggestion. We agree that a receptor-independent positive control is essential to verify that the engineered Jurkat reporter line is functionally competent and to rule out the possibility that weak or negative responses are merely artifacts of limited assay sensitivity.

To address this concern, we included a PMA/ionomycin (receptor-bypassing) positive control. By directly triggering downstream signaling independent of the TCR, PMA/ionomycin defines the maximal signaling capacity of the reporter line. As demonstrated in the new Supplementary Figure S29b, PMA/ionomycin stimulation induced near-complete CD69 upregulation in the TCR-Jurkat cells (98.8% $CD69^+$). This robust response confirms that the reporter line possesses full signaling competence and a wide dynamic activation window. Consequently, these findings verify that the graded responses observed across the peptide panel reflect genuine variations in TCR recognition rather than experimental artifacts stemming from limited assay sensitivity.

We have added these results as Supplementary Figure S29 and updated the Methods, and figure legend accordingly. To be clearer, the revised content is presented below.

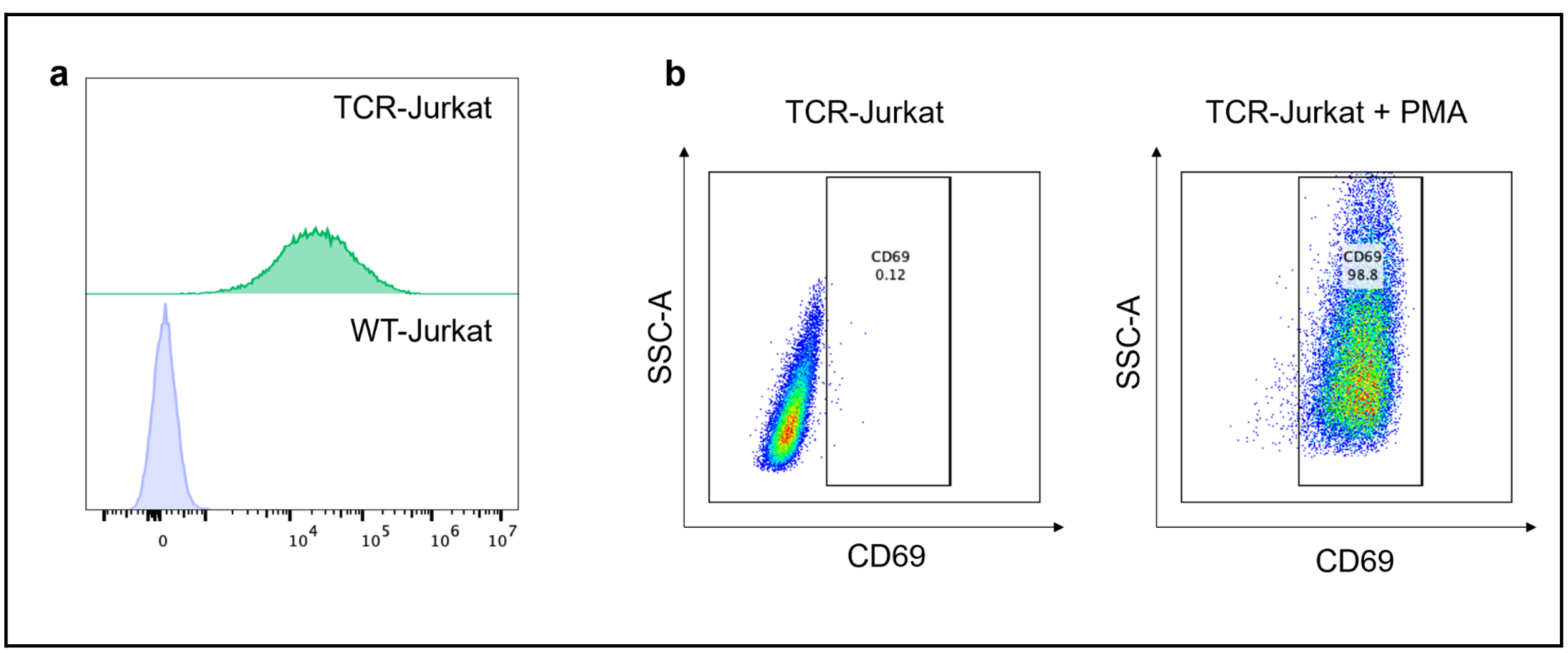

Figure S29. *PMA/ionomycin positive control confirms the signaling competence of the engineered TCR-Jurkat reporter line.* ***a****, Flow cytometry histograms showing TCR expression in engineered TCR$^+$ CD8$^+$ Jurkat cells versus parental CD8$^+$ Jurkat cells, confirming successful TCR transduction.* ***b,*** *Representative flow cytometry plots of CD69 expression without stimulation (left) and after PMA/ionomycin stimulation (right). PMA/ionomycin, which bypasses proximal TCR engagement to directly activate downstream signaling, induced near-complete CD69 upregulation (98.8% CD69$^+$), showing that the reporter line is signaling-competent with a broad activation window.*

Methods (page 48):

PMA/ionomycin (BioLegend, cat. no. 423304) was used as a positive control (Supplementary Figure S29) and DMSO and unrelated peptide as the negative controls.

Comment 2)

2. The cross-reactivity analysis (Figures 3f, 4a and 5; TCR-C1 to TCR-C20) is one of the manuscript's central interpretability claims, yet the materials needed to verify it are incomplete. The revised Table S1 lists only the V/J genes and CDR3α/CDR3β sequences for each TCR group, and the corresponding source-data sheet contains only a TCR identifier and a peptide column. Neither carries the HLA restriction of each TCR group or the originating (reported) epitope. This omission matters: a set of peptides can be interpreted as genuinely cross-reactive for a given TCR only if they are co-presented by the same restricting allele, and without the per-group HLA there is no way to confirm that the listed peptides share a restriction element rather than reflecting heterogeneous restrictions pooled together. Relatedly, the high- and low-conservation peptides are not labeled as to provenance, so it is not possible to tell which sequences are naturally occurring, gene-derived epitopes and which are synthetic, model-proposed variants - a distinction that bears directly on how the low-conservation results should be read.

Please annotate each cross-reactive TCR group (C1-C20) with its HLA restriction and its reported/source epitope (for example, GP33-41 / H2-Db for TCR-C10) and flag every peptide in the high- and low-conservation sets as synthetic versus gene-derived. Including the per-group co-presentation evidence (e.g., the NetMHCpan binding check already performed for the low-conservation peptides) in the same table would let a reader confirm shared restriction at a glance.

Response: We thank the reviewer for this important and constructive suggestion. We agree that the cross-reactivity analysis requires more complete annotation to allow independent verification, especially because genuine TCR cross-reactivity should be interpreted in the context of shared HLA restriction and peptide provenance. To address this, we have substantially expanded the corresponding source-data file. Specifically, we made the following additions and improvements:

1) Per-group HLA restriction and reported/source epitope. Every TCR group (C1–C20) is now annotated with its restricting HLA allele and its reported/source epitope, including the originating protein and its residue positions.
2) Provenance flag (synthetic vs. gene-derived) for every peptide. Each peptide in both the high- and low-conservation sets is now individually labeled as either "gene-derived" (a naturally occurring epitope documented in IEDB/VDJdb with a resolved source protein and position) or "synthetic".
3) Per-group co-presentation evidence (NetMHCpan). For every peptide we now report the NetMHCpan-4.1 predicted binding to the group's restricting allele (percentile rank), together with a binary binder flag (predicted binder at Rank ≤ 10%). This allows a reader to confirm shared restriction at a glance.

The corresponding Source Data sheet has been updated to mirror this information. We believe these additions allow readers to independently verify the shared restriction and provenance underlying every cross-reactivity claim.

To be clearer, the representative added content is presented below.

Cross-reactivity: TCR group information

TCR_group	Conservation_group	Restricting_HLA	Reported_source_epitope	Reported_source_protein	Reported_source_organism	Reported_source_position	CDR3a	CDR3b	TRAV	TRAJ	TRBV	TRBJ
C1	high	HLA-B*07:02	APRGPHGGAASGL	Cancer/testis antigen 1	Homo sapiens	60-72	CAASLNTG	CAILGQGY	TRAV13-1	TRAJ37*0	TRBV10-3	TRBJ1-2*01
C2	high	HLA-A*02:01	NLVPMVATV	65 kDa phosphoprotein	Human betaherpesvirus 5	495-503	CARNTGNQ	CASSPVTG	TRAV24	TRAJ49	TRBV6-5	TRBJ1-2
C3	high	H-2Db	ASNENMETM	Nucleoprotein	a A virus (A/Puerto Rico/8/193	366-374	CAASETSG	CASSRDLG	TRAV14N-	TRAJ15*0	TRBV17*0	TRBJ2-5*01
C4	high	HLA-A*02:01	ELAGIGILTV	ntigen recognized by T-cells 1 (MART-	Homo sapiens	26-35	CAVNDGGR	CAWSETGL	TRAV12-2	TRAJ27*0	TRBV30*0	TRBJ2-2*01
C5	high	HLA-B*35:08	LPEPLPQGQLTAY	Lytic switch protein BZLF1	human gammaherpesvirus 4	52-64	CALSGFYN	CASPGLAG	TRAV19*0	TRAJ34*0	TRBV6-1*	TRBJ2-7*01
C6	high	HLA-A*02:01	ELAGIGILTV	ntigen recognized by T-cells 1 (MART-	Homo sapiens	26-35	CAVTKYSW	CASRPGWM	TRAV12-2	TRAJ24*0	TRBV6-5*	TRBJ2-7*01
C7	high	HLA-B*27:05	LRVMMLAPF	Hypothetical membrane protein	a enterica subsp. enterica ser	232-240	CAVSSPQG	CASSVGLY	TRAV21	TRAJ57	TRBV9	TRBJ2-3
C8	high	HLA-A*02:01	MMDFFNAQM	Splicing factor U2AF 65 kDa subunit	Homo sapiens	165-173	CALSEARG	CASSRDTV	TRAV19*0	TRAJ32*0	TRBV10-1	TRBJ1-1*01
C9	high	HLA-A*02:01	ALWGPDPAAA	Insulin	Homo sapiens	15-24	CAMRGDSS	CASSLWEK	TRAV12-3	TRAJ12*0	TRBV12-4	TRBJ2-4*01
C10	high	H-2Db	KAVYNFATM	re-glycoprotein polyprotein GP comple	mphocytic choriomeningitis vir	33-41	CAALYGNE	CASSDAGG	TRAV14D-	TRAJ48*0	TRBV13-3	TRBJ2-4*01
C11	low	HLA-A*02:01	EAAGIGILTV	ntigen recognized by T-cells 1 (MART-	Homo sapiens	26-35	CAVNVAGK	CAWSETGL	TRAV12-2	TRAJ27*0	TRBV30*0	TRBJ2-2*01
C12	low	HLA-A*02:01	KLEQLDWTV	Gem-associated protein 4	Homo sapiens	762-770	CALSSRGS	CASSPSGL	TRAV19*0	TRAJ18*0	TRBV11-3	TRBJ2-1*01
C13	low	HLA-A*02:01	KLLEIAPNC	DNA 3'-5' helicase	Merkel cell polyomavirus	15-23	CVVALYSG	CASRSQNY	TRAV12-1	TRAJ45*0	TRBV6-5*	TRBJ1-2*01
C14	low	H-2Kb	SIYRYYGL	R/AcrR family transcriptional regula	Streptomyces hainanensis	99-106	CAVSGFAS	CASGGGGT	TRAV9-4*	TRAJ35*0	TRBV13-2	TRBJ2-4*01
C15	low	HLA-B*57:03	KAFSPEVIPMF	Gag-Pol polyprotein	Human immunodeficiency virus 1	49-59	CAVSGGYQ	CASTGSYG	TRAV5*01	TRAJ13*0	TRBV19*0	TRBJ1-2*01
C16	low	HLA-A*02:01	KLLEIAPNC	DNA 3'-5' helicase	Merkel cell polyomavirus	15-23	CVVATYSG	CASSIIAG	TRAV12-1	TRAJ45*0	TRBV19*0	TRBJ2-1*01
C17	low	H-2Ld	QLSPFPFDL	lutarate dehydrogenase complex compon	Mus musculus	932-940	CAVSAKGT	CASSDAPG	TRAV9D-3	TRAJ58*0	TRBV13-1	TRBJ2-2*01
C18	low	HLA-A*02:01	KLLEIAPNC	DNA 3'-5' helicase	Merkel cell polyomavirus	15-23	CAYNQGGK	CASSVLNT	TRAV38-1	TRAJ23*0	TRBV10-2	TRBJ2-2*01
C19	low	HLA-A*02:01	KLLEIAPNC	DNA 3'-5' helicase	Merkel cell polyomavirus	15-23	CVVTEYSG	CASRGQNT	TRAV12-1	TRAJ45*0	TRBV10-3	TRBJ2-2*01
C20	low	HLA-A*02:01	KLLEIAPNC	DNA 3'-5' helicase	Merkel cell polyomavirus	15-23	CVVPLYSS	CASSDTPD	TRAV12-1	TRAJ3*01	TRBV6-1*	TRBJ1-1*01

Cross-reactivity: peptide information

TCR_group	Conservation_group	Peptide	Restricting_HLA	Peptide_class	Provenance_evidence	Source_protein	Source_organism	NetMHCpan_percentile_rank	NetMHCpan_binder(Rank<=10%)
C1	high	APRGPHGGAASGL	HLA-B*07:02	gene-derived	IEDB:Homo sapiens	er/testis antig	Homo sapiens	0.05	Yes
C1	high	APRGPHGGAAAGL	HLA-B*07:02	synthetic	IEDB:no-position(analog/variant)			0.06	Yes
C1	high	APRGPHGGAASGA	HLA-B*07:02	synthetic	IEDB:no-position(analog/variant)			0.51	Yes
C1	high	APRGPHGGAASAL	HLA-B*07:02	synthetic	IEDB:no-position(analog/variant)			0.02	Yes
C1	high	APRGPHAGAASGL	HLA-B*07:02	synthetic	IEDB:no-position(analog/variant)			0.05	Yes
C1	high	APRGAHGGAASGL	HLA-B*07:02	synthetic	IEDB:no-position(analog/variant)			0.05	Yes
C1	high	AARGPHGGAASGL	HLA-B*07:02	synthetic	IEDB:no-position(analog/variant)			2.2	Yes
C1	high	APAGPHGGAASGL	HLA-B*07:02	synthetic	IEDB:no-position(analog/variant)			0.3	Yes
C2	high	NLVPMVATV	HLA-A*02:01	gene-derived	IEDB:Human betaherpesvirus 5	kDa phosphoprot	uman betaherpesvirus	0.17	Yes
C2	high	NLVPMVASV	HLA-A*02:01	synthetic	IEDB:no-position(analog/variant)			0.13	Yes
C2	high	NLVPVVATV	HLA-A*02:01	synthetic	IEDB:no-position(analog/variant)			0.15	Yes
C2	high	NLVGMVATV	HLA-A*02:01	synthetic	IEDB:no-position(analog/variant)			0.19	Yes
C2	high	NLVAMVATV	HLA-A*02:01	synthetic	IEDB:no-position(analog/variant)			0.2	Yes
C2	high	NLVPTVATV	HLA-A*02:01	synthetic	IEDB:no-position(analog/variant)			0.31	Yes
C2	high	NLVPQVATV	HLA-A*02:01	synthetic	IEDB:no-position(analog/variant)			0.25	Yes
C3	high	ASNENMETM	H-2Db	gene-derived	uenza A virus (A/Puerto Rico/8/19	Nucleoprotein	irus (A/Puerto Rico/	0.01	Yes
C3	high	ASNENWETM	H-2Db	synthetic	IEDB:no-position(analog/variant)			0.02	Yes
C3	high	ASNANMETM	H-2Db	synthetic	IEDB:no-position(analog/variant)			0.01	Yes
C3	high	ASNENAETM	H-2Db	synthetic	IEDB:no-position(analog/variant)	Nucleoprotein	H1N1 subtype	0.02	Yes
C3	high	ASAENMETM	H-2Db	synthetic	IEDB:no-position(analog/variant)			0.01	Yes
C3	high	NSNENMETM	H-2Db	synthetic	IEDB:no-position(analog/variant)			0.03	Yes
C3	high	ASNENMATM	H-2Db	synthetic	IEDB:no-position(analog/variant)			0.02	Yes
C3	high	ASNENMEAM	H-2Db	gene-derived	a A virus (A/chicken/Hong Kong/TP	Nucleoprotein	(A/chicken/Hong Kong	0.02	Yes
C4	high	ELAAIGILTV	HLA-A*02:01	synthetic	IEDB:no-position(analog/variant)			1.7	Yes
C4	high	ELAGIGALTV	HLA-A*02:01	synthetic	IEDB:no-position(analog/variant)			3.1	Yes
C4	high	ELAGIGILTV	HLA-A*02:01	synthetic	IEDB:no-position(analog/variant)			1.4	Yes
C4	high	AAGIGILTV	HLA-A*02:01	gene-derived	IEDB:Homo sapiens	igen recognized	Homo sapiens	6.8	Yes
C4	high	EAAGIGILTV	HLA-A*02:01	gene-derived	IEDB:Homo sapiens	igen recognized	Homo sapiens	9.7	Yes
C4	high	ALAGIGILTV	HLA-A*02:01	synthetic	IEDB:no-position(analog/variant)			0.15	Yes
C4	high	ELAGIGIATV	HLA-A*02:01	synthetic	IEDB:no-position(analog/variant)			1.7	Yes
C5	high	LPEPLPQGQLTAY	HLA-B*35:08	gene-derived	IEDB:human gammaherpesvirus 4	switch protein	man gammaherpesvirus	0.21	Yes
C5	high	GPEPLPQGQLTAY	HLA-B*35:08	synthetic	VDJdb:EBV	BZLF1	EBV	1.4	Yes
C5	high	LPEGLPQGQLTAY	HLA-B*35:08	synthetic	VDJdb:EBV	BZLF1	EBV	0.25	Yes
C5	high	LPEPLGQGQLTAY	HLA-B*35:08	synthetic	VDJdb:EBV	BZLF1	EBV	0.17	Yes
C5	high	LPEPLPQAQLTAY	HLA-B*35:08	synthetic	VDJdb:EBV	BZLF1	EBV	0.22	Yes
C5	high	LPEPLPQGGLTAY	HLA-B*35:08	synthetic	VDJdb:EBV	BZLF1	EBV	0.14	Yes
C5	high	LPEPLPQGQGTAY	HLA-B*35:08	synthetic	VDJdb:EBV	BZLF1	EBV	0.16	Yes
C5	high	LPEPLPQGQLGAY	HLA-B*35:08	synthetic	VDJdb:EBV	BZLF1	EBV	0.28	Yes
C5	high	LPEPLPQGQLTGY	HLA-B*35:08	synthetic	VDJdb:EBV	BZLF1	EBV	0.75	Yes
C5	high	LPEALPQGQLTAY	HLA-B*35:08	synthetic	IEDB:no-position(analog/variant)			0.25	Yes
C5	high	LPEPLPQGQATAY	HLA-B*35:08	synthetic	IEDB:no-position(analog/variant)			0.16	Yes
C5	high	LPEPLPQGALTAY	HLA-B*35:08	synthetic	IEDB:no-position(analog/variant)			0.16	Yes

Reviewer #4 (Remarks to the Author):

Summary

I co-reviewed this manuscript with one of the reviewers who provided the listed reports. This is part of the Nature Communications initiative to facilitate training in peer review and to provide appropriate recognition for Early Career Researchers who co-review manuscripts. Many of the comments have been addressed, but there still remains few concerns.

Response: We sincerely thank Reviewer #4 for the valuable feedback and constructive suggestions on our manuscript. We appreciate the time and effort devoted to helping us improve the quality of our work.

Comment 1)

For comment 3 (Figure 3f, Figure 4a, Figure 5 cross-reactivity analysis of TCR-C1 to C20), please include the MHC restrictions of TCR-C1–C20 along with their reported epitopes (for example, GP33–41/H2-Db for TCR C10). Please also label the synthetic vs gene-derived high/low-conservation peptides, as I was unable to locate them in the source file or supplementary tables, though I may have missed them.

Response: We thank the reviewer for this important and constructive suggestion. We fully agree that the cross-reactivity analysis necessitates comprehensive annotations to facilitate independent verification, particularly since genuine TCR cross-reactivity must be interpreted within the context of shared HLA restriction and peptide provenance. To address this, we have substantially expanded the corresponding source data file by implementing the following additions and enhancements:

- Group-level HLA restriction and source epitope mapping: Every TCR group (C1–C20) is now systematically annotated with its restricting HLA allele and its

reported/source epitope, including the originating protein name and specific residue positions.

- Granular provenance flagging (synthetic vs. gene-derived): Each peptide within both the high- and low-conservation sets is now individually classified as either "gene-derived" (naturally occurring epitopes documented in IEDB/VDJdb with established source proteins) or "synthetic".
- Co-presentation evidence profiling (NetMHCpan): For every peptide, we now report the NetMHCpan-4.1 predicted binding affinity to the group's restricting allele (% Rank), along with a binary binding status flag. This enables readers to verify shared restriction profiles at a glance.

The corresponding Source Data sheet has been updated to fully reflect these data. We believe these additions allow readers to independently verify the provenance underlying every cross-reactivity claim. To illustrate these updates, a representative excerpt of the newly added content is presented below:

TCR group information

TCR_group	Conservation_group	Restricting_HLA	Reported_source_epitope	Reported_source_protein	Reported_source_organism	Reported_source_position	CDR3a	CDR3b	TRAV	TRAJ	TRBV	TRBJ
C1	high	HLA-B*07:02	APRGPHGGAASGL	Cancer/testis antigen 1	Homo sapiens	60-72	CAASLNTG	CAILGQGY	TRAV13-1	TRAJ37*0	TRBV10-3	TRBJ1-2*01
C2	high	HLA-A*02:01	NLVPMVATV	65 kDa phosphoprotein	Human betaherpesvirus 5	495-503	CARNTGNQ	CASSPVTG	TRAV24	TRAJ49	TRBV6-5	TRBJ1-2
C3	high	H-2Db	ASNENMETM	Nucleoprotein	a A virus (A/Puerto Rico/8/193	366-374	CAASETSG	CASSRDLG	TRAV14N-	TRAJ15*0	TRBV17*0	TRBJ2-5*01
C4	high	HLA-A*02:01	ELAGIGILTV	ntigen recognized by T-cells 1 (MART-	Homo sapiens	26-35	CAVNDGGR	CAWSETGL	TRAV12-2	TRAJ27*0	TRBV30*0	TRBJ2-2*01
C5	high	HLA-B*35:08	LPEPLPQGQLTAY	Lytic switch protein BZLF1	human gammaherpesvirus 4	52-64	CALSGFYN	CASPGLAG	TRAV19*0	TRAJ34*0	TRBV6-1*	TRBJ2-7*01
C6	high	HLA-A*02:01	ELAGIGILTV	ntigen recognized by T-cells 1 (MART-	Homo sapiens	26-35	CAVTKYSW	CASRPGWM	TRAV12-2	TRAJ24*0	TRBV6-5*	TRBJ2-7*01
C7	high	HLA-B*27:05	LRVMMLAPF	Hypothetical membrane protein	a enterica subsp. enterica ser	232-240	CAVSSPQG	CASSVGLY	TRAV21	TRAJ57	TRBV9	TRBJ2-3
C8	high	HLA-A*02:01	MMDFFNAQM	Splicing factor U2AF 65 kDa subunit	Homo sapiens	165-173	CALSEARG	CASSRDTV	TRAV19*0	TRAJ32*0	TRBV10-1	TRBJ1-1*01
C9	high	HLA-A*02:01	ALWGPDPAAA	Insulin	Homo sapiens	15-24	CAMRGDSS	CASSLWEK	TRAV12-3	TRAJ12*0	TRBV12-4	TRBJ2-4*01
C10	high	H-2Db	KAVYNFATM	re-glycoprotein polyprotein GP comple	mphocytic choriomeningitis vir	33-41	CAALYGNE	CASSDAGG	TRAV14D-	TRAJ48*0	TRBV13-3	TRBJ2-4*01
C11	low	HLA-A*02:01	EAAGIGILTV	ntigen recognized by T-cells 1 (MART-	Homo sapiens	26-35	CAVNVAGK	CAWSETGL	TRAV12-2	TRAJ27*0	TRBV30*0	TRBJ2-2*01
C12	low	HLA-A*02:01	KLEQLDWTV	Gem-associated protein 4	Homo sapiens	762-770	CALSSRGS	CASSPSGL	TRAV19*0	TRAJ18*0	TRBV11-3	TRBJ2-1*01
C13	low	HLA-A*02:01	KLLEIAPNC	DNA 3'-5' helicase	Merkel cell polyomavirus	15-23	CVVALYSG	CASRSQNY	TRAV12-1	TRAJ45*0	TRBV6-5*	TRBJ1-2*01
C14	low	H-2Kb	SIYRYYGL	R/AcrR family transcriptional regula	Streptomyces hainanensis	99-106	CAVSGFAS	CASGGGGT	TRAV9-4*	TRAJ35*0	TRBV13-2	TRBJ2-4*01
C15	low	HLA-B*57:03	KAFSPEVIPMF	Gag-Pol polyprotein	Human immunodeficiency virus 1	49-59	CAVSGGYQ	CASTGSYG	TRAV5*01	TRAJ13*0	TRBV19*0	TRBJ1-2*01
C16	low	HLA-A*02:01	KLLEIAPNC	DNA 3'-5' helicase	Merkel cell polyomavirus	15-23	CVVATYSG	CASSIIAG	TRAV12-1	TRAJ45*0	TRBV19*0	TRBJ2-1*01
C17	low	H-2Ld	QLSPFPFDL	lutarate dehydrogenase complex compon	Mus musculus	932-940	CAVSAKGT	CASSDAPG	TRAV9D-3	TRAJ58*0	TRBV13-1	TRBJ2-2*01
C18	low	HLA-A*02:01	KLLEIAPNC	DNA 3'-5' helicase	Merkel cell polyomavirus	15-23	CAYNQGGK	CASSVLNT	TRAV38-1	TRAJ23*0	TRBV10-2	TRBJ2-2*01
C19	low	HLA-A*02:01	KLLEIAPNC	DNA 3'-5' helicase	Merkel cell polyomavirus	15-23	CVVTEYSG	CASRGQNT	TRAV12-1	TRAJ45*0	TRBV10-3	TRBJ2-2*01
C20	low	HLA-A*02:01	KLLEIAPNC	DNA 3'-5' helicase	Merkel cell polyomavirus	15-23	CVVPLYSS	CASSDTPD	TRAV12-1	TRAJ3*01	TRBV6-1*	TRBJ1-1*01

Peptide information

TCR_group	Conservation_group	Peptide	Restricting_HLA	Peptide_class	Provenance_evidence	Source_protein	Source_organism	NetMHCpan_percentile_rank	NetMHCpan_binder(Rank<=10%)
C1	high	APRGPHGGAASGL	HLA-B*07:02	gene-derived	IEDB:Homo sapiens	cer/testis antige	Homo sapiens	0.05	Yes
C1	high	APRGPHGGAAAGL	HLA-B*07:02	synthetic	IEDB:no-position(analog/variant)			0.06	Yes
C1	high	APRGPHGGAASGA	HLA-B*07:02	synthetic	IEDB:no-position(analog/variant)			0.51	Yes
C1	high	APRGPHGGAASAL	HLA-B*07:02	synthetic	IEDB:no-position(analog/variant)			0.02	Yes
C1	high	APRGPHAGAASGL	HLA-B*07:02	synthetic	IEDB:no-position(analog/variant)			0.05	Yes
C1	high	APRGAHGGAASGL	HLA-B*07:02	synthetic	IEDB:no-position(analog/variant)			0.05	Yes
C1	high	AARGPHGGAASGL	HLA-B*07:02	synthetic	IEDB:no-position(analog/variant)			2.2	Yes
C1	high	APAGPHGGAASGL	HLA-B*07:02	synthetic	IEDB:no-position(analog/variant)			0.3	Yes
C2	high	NLVPMVATV	HLA-A*02:01	gene-derived	IEDB:Human betaherpesvirus 5	kDa phosphoprote	uman betaherpesvirus	0.17	Yes
C2	high	NLVPMVASV	HLA-A*02:01	synthetic	IEDB:no-position(analog/variant)			0.13	Yes
C2	high	NLVPVVATV	HLA-A*02:01	synthetic	IEDB:no-position(analog/variant)			0.15	Yes
C2	high	NLVGMVATV	HLA-A*02:01	synthetic	IEDB:no-position(analog/variant)			0.19	Yes
C2	high	NLVAMVATV	HLA-A*02:01	synthetic	IEDB:no-position(analog/variant)			0.2	Yes
C2	high	NLVPTVATV	HLA-A*02:01	synthetic	IEDB:no-position(analog/variant)			0.31	Yes
C2	high	NLVPQVATV	HLA-A*02:01	synthetic	IEDB:no-position(analog/variant)			0.25	Yes
C3	high	ASNENMETM	H-2Db	gene-derived	uenza A virus (A/Puerto Rico/8/19	Nucleoprotein	irus (A/Puerto Rico/	0.01	Yes
C3	high	ASNENWETM	H-2Db	synthetic	IEDB:no-position(analog/variant)			0.02	Yes
C3	high	ASNANMETM	H-2Db	synthetic	IEDB:no-position(analog/variant)			0.01	Yes
C3	high	ASNENAETM	H-2Db	synthetic	IEDB:no-position(analog/variant)	Nucleoprotein	H1N1 subtype	0.02	Yes
C3	high	ASAENMETM	H-2Db	synthetic	IEDB:no-position(analog/variant)			0.01	Yes
C3	high	NSNENMETM	H-2Db	synthetic	IEDB:no-position(analog/variant)			0.03	Yes
C3	high	ASNENMATM	H-2Db	synthetic	IEDB:no-position(analog/variant)			0.02	Yes
C3	high	ASNENMEAM	H-2Db	gene-derived	a A virus (A/chicken/Hong Kong/TP3	Nucleoprotein	(A/chicken/Hong Kong	0.02	Yes
C4	high	ELAAIGILTV	HLA-A*02:01	synthetic	IEDB:no-position(analog/variant)			1.7	Yes
C4	high	ELAGIGALTV	HLA-A*02:01	synthetic	IEDB:no-position(analog/variant)			3.1	Yes
C4	high	ELAGIGILTV	HLA-A*02:01	synthetic	IEDB:no-position(analog/variant)			1.4	Yes
C4	high	AAGIGILTV	HLA-A*02:01	gene-derived	IEDB:Homo sapiens	igen recognized	Homo sapiens	6.8	Yes
C4	high	EAAGIGILTV	HLA-A*02:01	gene-derived	IEDB:Homo sapiens	igen recognized	Homo sapiens	9.7	Yes
C4	high	ALAGIGILTV	HLA-A*02:01	synthetic	IEDB:no-position(analog/variant)			0.15	Yes
C4	high	ELAGIGIATV	HLA-A*02:01	synthetic	IEDB:no-position(analog/variant)			1.7	Yes
C5	high	LPEPLPQGQLTAY	HLA-B*35:08	gene-derived	IEDB:human gammaherpesvirus 4	switch protein	man gammaherpesvirus	0.21	Yes
C5	high	GPEPLPQGQLTAY	HLA-B*35:08	synthetic	VDJdb:EBV	BZLF1	EBV	1.4	Yes
C5	high	LPEGLPQGQLTAY	HLA-B*35:08	synthetic	VDJdb:EBV	BZLF1	EBV	0.25	Yes
C5	high	LPEPLGQGQLTAY	HLA-B*35:08	synthetic	VDJdb:EBV	BZLF1	EBV	0.17	Yes
C5	high	LPEPLPQAQLTAY	HLA-B*35:08	synthetic	VDJdb:EBV	BZLF1	EBV	0.22	Yes
C5	high	LPEPLPQGGLTAY	HLA-B*35:08	synthetic	VDJdb:EBV	BZLF1	EBV	0.14	Yes
C5	high	LPEPLPQGQGTAY	HLA-B*35:08	synthetic	VDJdb:EBV	BZLF1	EBV	0.16	Yes
C5	high	LPEPLPQGQLGAY	HLA-B*35:08	synthetic	VDJdb:EBV	BZLF1	EBV	0.28	Yes
C5	high	LPEPLPQGQLTGY	HLA-B*35:08	synthetic	VDJdb:EBV	BZLF1	EBV	0.75	Yes
C5	high	LPEALPQGQLTAY	HLA-B*35:08	synthetic	IEDB:no-position(analog/variant)			0.25	Yes
C5	high	LPEPLPQGQATAY	HLA-B*35:08	synthetic	IEDB:no-position(analog/variant)			0.16	Yes
C5	high	LPEPLPQGALTAY	HLA-B*35:08	synthetic	IEDB:no-position(analog/variant)			0.16	Yes

Comment 2)

Moreover, if feasible, it would be interesting to assess how UpTCR performs on recently published datasets such as the autoantigen/HLA-B27 restricted TCRs reported by Wang et al (2026 Nature Biotechnology). Even a brief analysis or discussion of these cases could help contextualize the generalizability across newly characterized antigen-TCR pairs.

Wang, N., Yeh, H., Lai, B., Perera, J., Jude, K. M., Risch, I., ... & Garcia, K. C. (2026). Deep peptide recognition profiling decodes TCR specificity and enables disease-associated antigen discovery. Nature Biotechnology, 1-11.

Response: We thank the reviewer for this valuable suggestion and for pointing us to this important work. We agree that assessing UpTCR on newly characterized antigen-TCR pairs helps contextualize its generalizability, and we have accordingly evaluated UpTCR on the HLA-B*27:05-restricted autoantigen dataset reported by Wang et al. (2026).

Specifically, we used their experimentally measured T-cell activation data as ground truth, comprising 336 TCR-peptide pairs (21 HLA-B*27:05-restricted TCRs from patients with ankylosing spondylitis and acute anterior uveitis, each tested against 16 candidate peptides; 65 activating and 271 non-activating pairs). Applied in a zero-shot setting, UpTCR achieved the highest performance among all competing methods (Supplementary Figure S28), indicating promising generalization ability.

We have added this analysis as Supplementary Figure S28, together with a brief description in the Discussion and the corresponding Methods.

To be clearer, the revised content is presented below.

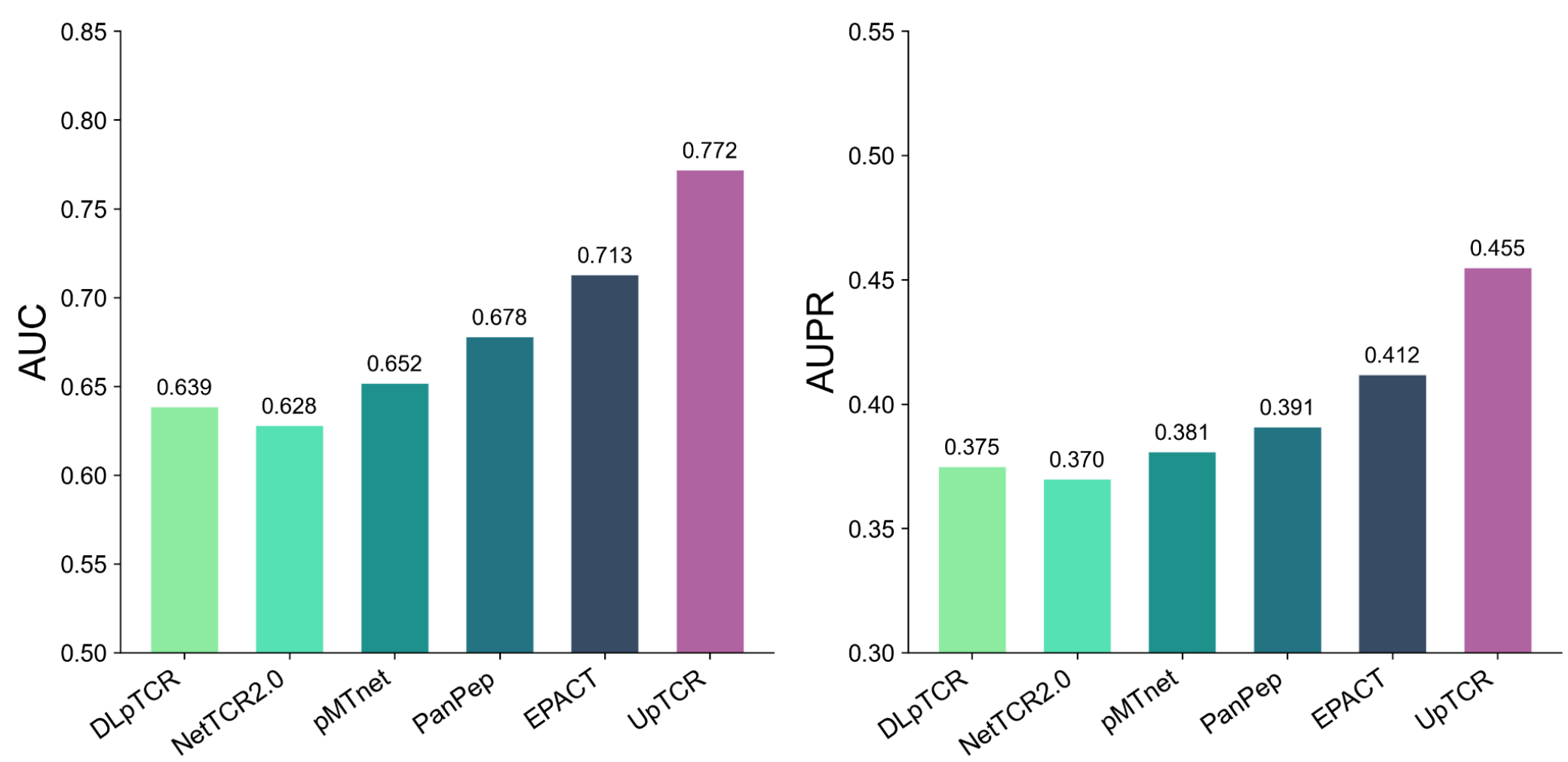

Figure S28. *Performance of UpTCR and baselines on the previously unseen HLA-B*27:05-restricted autoantigen system from Wang et al. UpTCR achieved higher performance than other methods, with an AUC of 0.772 (Left) and an AUPR of 0.455 (Right).*

Discussion (page 35):

…To further probe generalizability, we evaluated UpTCR on an independent HLA-B*27:05-restricted autoantigen dataset reported by Wang et al[1]. In a zero-shot setting, UpTCR achieved the highest performance among competing methods (Supplementary Figure S28), indicating promising generalization ability.

Methods (page 38):

…To further assess the generalizability of UpTCR, we evaluated it on an independent dataset from Wang et al.[1], which characterized HLA-B*27:05-restricted TCRs from patients with ankylosing spondylitis and acute anterior uveitis. We used their experimentally measured T-cell activation data as ground truth, yielding 336 TCR-peptide pairs (21 TCRs, each tested against 16 candidate peptides; 65 activating and 271 non-activating pairs). All models were evaluated in a strict zero-shot setting.

Comment 3)

For comment 7 (Figure 8), it would be helpful to consider a more responsive T cell line. Inclusion of PMA/ionomycin positive control could show signaling competence in the T cell line. In its current setup, the Jurkat line used may have limited sensitivity to distinguish between cognate and non-cognate peptide in a CD69 upregulation readout.

Response: We thank the reviewer for this helpful suggestion. We agree that including a PMA/ionomycin positive control is important to demonstrate the signaling competence of the T-cell line and to rule out the possibility that limited assay sensitivity, rather than genuine differences in TCR recognition, accounts for the weaker or negative responses.

To address this concern, we added a PMA/ionomycin (receptor-bypassing) positive control. Because PMA/ionomycin directly activates downstream signaling independently of TCR engagement, it reports the maximal signaling capacity of the reporter line. As shown in the new Supplementary Figure S29, PMA/ionomycin stimulation induced near-complete CD69 upregulation in the TCR-Jurkat cells (98.8% $CD69^+$), compared with minimal basal expression in unstimulated cells (0.12%). This confirms that the engineered TCR-Jurkat line is fully signaling-competent with a broad activation window, and therefore that the reporter system is able to distinguish cognate from non-cognate

peptides: the graded CD69 responses across the peptide panel reflect true differences in TCR recognition rather than limited assay sensitivity.

We have added these results as Supplementary Figure S29 and updated the Methods and figure legend accordingly. We thank the reviewer for this constructive suggestion, which has strengthened the interpretability of our functional validation.

To be clearer, the revised content is presented below.

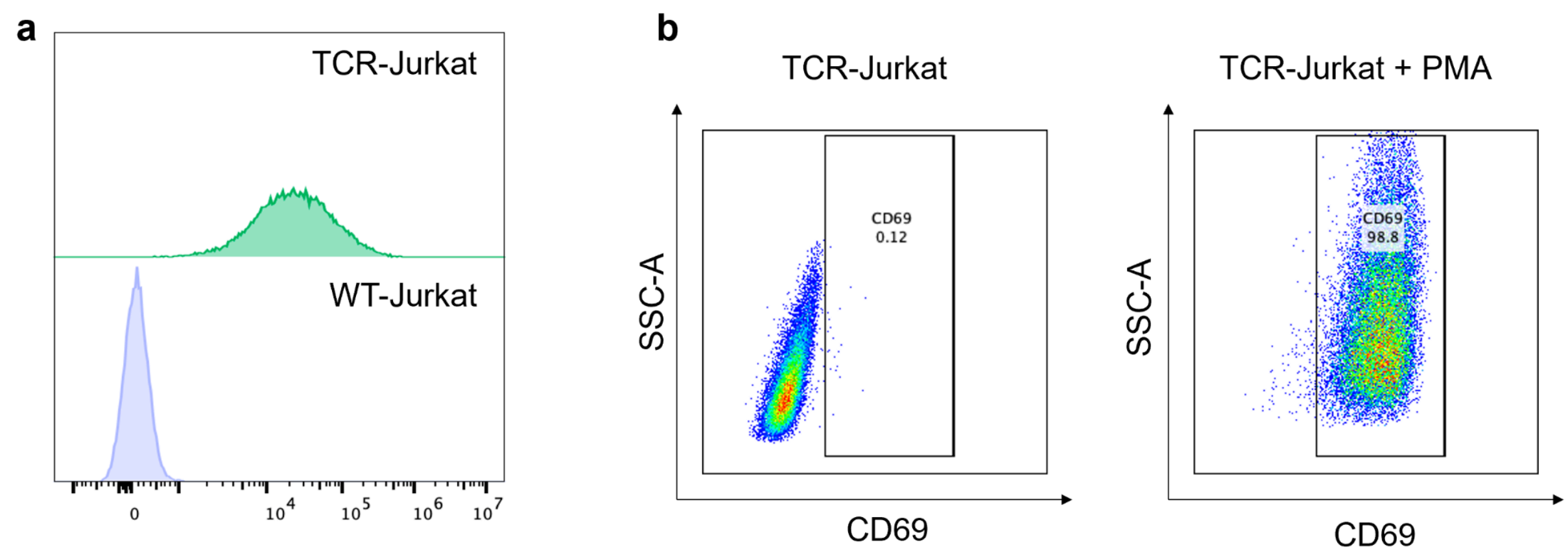

Figure S29. *PMA/ionomycin positive control confirms the signaling competence of the engineered TCR-Jurkat reporter line.* ***a****, Flow cytometry histograms showing TCR expression in engineered TCR*$^+$ *CD8*$^+$ *Jurkat cells versus parental CD8*$^+$ *Jurkat cells, confirming successful TCR transduction.* ***b,*** *Representative flow cytometry plots of CD69 expression without stimulation (left) and after PMA/ionomycin stimulation (right). PMA/ionomycin, which bypasses proximal TCR engagement to directly activate downstream signaling, induced near-complete CD69 upregulation (98.8% CD69*$^+$*), showing that the reporter line is signaling-competent with a broad activation window.*

Methods (page):

PMA/ionomycin (BioLegend, cat. no. 423304) was used as a positive control (Supplementary Figure S29) and DMSO and unrelated peptide as the negative controls.

Reference

1. Wang, N. *et al.* Deep peptide recognition profiling decodes TCR specificity and enables disease-associated antigen discovery. *Nat. Biotechnol.* https://doi.org/10.1038/s41587-026-03128-x (2026) doi:10.1038/s41587-026-03128-x.

Point-by-point response

Manuscript title: "UpTCR: a unified progressive knowledge transfer foundation model for robust T-cell receptor-antigen binding recognition"

Dear Reviewers,

Thank you all for your valuable time, constructive comments, and helpful suggestions, which have significantly improved our manuscript. Here we include a point-by-point response to the reviewers, where *reviewers' comments are presented in italics*, our responses are in blue, and changes made to the manuscript are shown in red (*figure captions are in italics*).

Reviewer #3 (Remarks to the Author):

Summary

The revised manuscript appears to have adequately addressed this reviewer's comments. Thank you for the opportunity to review this manuscript.

Response: We sincerely thank Reviewer #3 for the valuable and constructive comments. Their efforts in reviewing our manuscript have been instrumental in improving our work.

Reviewer #4 (Remarks to the Author):

Summary

I co-reviewed this manuscript with one of the reviewers who provided the listed reports. This is part of the Nature Communications initiative to facilitate training in peer review and to provide appropriate recognition for Early Career Researchers who co-review manuscripts.

Response: We sincerely thank Reviewer #4 for the valuable feedback on our manuscript. We appreciate the time and effort devoted to helping us improve the quality of our work.
